# p21 (CDKN1A) Is the Major Driver of Sulforaphane-Mediated Reduction in SAMHD1 T592 Phosphorylation in Macrophages

**DOI:** 10.3390/biom16081213

**Published:** 2026-08-20

**Authors:** Bianka Nicolle Pena Marcelino, Kiersten Girard, Lauren Letourneau, Andrew Lewin, David Lewin, Anna Presicci, Luke Reistrom, Tyler Williams, H. John Sharifi

**Affiliations:** 1 Department of Biological and Environmental Sciences, Le Moyne College, 1419 Salt Springs Road, Syracuse, NY 13214, USA; 2 Department of Psychology, Le Moyne College, 1419 Salt Springs Road, Syracuse, NY 13214, USA

**Keywords:** sulforaphane, SFN, macrophage, THP-1, hMDM, HIV, NRF2, p21, SAMHD1, T592 phosphorylation

## Abstract

Sulforaphane (SFN), a natural compound found in cruciferous vegetables, mobilizes the transcription factor NRF2 to protect macrophages from HIV-1. SFN/NRF2 exerts this protective effect by promoting the reduced phosphorylation of the antiviral protein SAMHD1. Phosphorylation at threonine 592 (T592) potently inhibits the capacity of SAMHD1 to restrict HIV-1. How SFN, and other NRF2 mobilizers reduce SAMHD1 T592 phosphorylation is unclear. p21 (CDKN1A) is an NRF2-responsive protein that accumulates in primary macrophages after SFN treatment. p21 blocks SAMHD1 T592 phosphorylation through the inhibition of several cyclin-dependent kinases. We therefore hypothesized that SFN acts through p21 to reduce SAMHD1 T592 phosphorylation in macrophages. Here, we use RNAi, CRISPR-Cas9, and pharmacological inhibition to deplete or delete p21 in macrophages and demonstrate that p21 is necessary for SFN to efficiently reduce SAMHD1 T592 phosphorylation and restrict HIV-1 transduction.

## 1. Introduction

The Sterile Alpha Motif and HD domain-containing protein 1 (SAMHD1) is a cellular restriction factor capable of inhibiting early events in HIV-1 replication. The capacity of SAMHD1 to block HIV-1 is regulated by phosphorylation at threonine 592 (T592) of the protein. T592 phosphorylation abrogates the capability of SAMHD1 to restrict HIV-1. In addition to impacting early events in HIV replication, modulation of SAMHD1 T592 phosphorylation contributes to the protein’s roles in HIV latency, non-retrovirus replication, LINE retrotransposon regulation, DNA repair, cell cycle progression, oncogenesis, and immune regulation. The functions of SAMHD1 are extensively reviewed in [1,2,3,4,5,6]. Thus, identifying druggable pathways capable of influencing SAMHD1 T592 phosphorylation represents an attractive therapeutic strategy.

Sulforaphane (SFN) is an isothiocyanate released from cruciferous vegetables upon mechanical damage. In mammals, SFN mobilizes the master antioxidant response transcription factor, nuclear factor erythroid 2-related factor 2 (NRF2) by interfering with constitutive NRF2 turnover mediated by the KEAP1-CUL3 ubiquitin ligase (reviewed in [7]). In human macrophages, SFN upregulates NRF2 to protect these cells from HIV-1 [8,9]. SFN/NRF2 ultimately exerts this protective effect through SAMHD1 via reduced T592 phosphorylation. However, the mechanism by which SFN, and other NRF2 mobilizers, reduce SAMHD1 T592 phosphorylation in macrophages remains unclear [10].

A primary candidate for mediating this effect is p21 (CDKN1A), a member of the Cip/Kip family of cyclin-dependent kinase (CDK) inhibitors. Originally identified as a cell cycle regulator and tumor suppressor, p21 is now recognized as a multifaceted protein capable of influencing a broad array of cellular processes in a context-dependent manner. As such, p21 is heavily regulated by a multitude of intracellular and extracellular stimuli at the transcriptional, translational, and post-translational levels (reviewed in [11]). In macrophages, p21 contributes to cell cycle withdrawal and the initiation of monocyte differentiation [12]. Further, elevated levels of p21 in these cells promote M2 polarization [13], a phenotype associated with tissue repair and the resolution of pro-inflammatory responses (reviewed in [14,15]). One hallmark of M2 macrophages is a high antioxidant capacity and low reactive oxygen species, a state facilitated by NRF2 activity (reviewed in [16]). p21 contributes to NRF2 activation via a reciprocal reinforcement loop wherein NRF2 upregulates p21 transcription, while p21 physically binds to and protects NRF2 from proteasomal degradation (reviewed in [17,18]).

Another hallmark of M2 macrophage polarization is the p21-augmented reduction in pro-inflammatory NF-κB activation [13,19]. Despite this anti-inflammatory role, p21 contributes to HIV-1 restriction in macrophages by helping to maintain SAMHD1 anti-retroviral activity. Specifically, p21 blocks CDK1/2-mediated SAMHD1 T592 phosphorylation [20,21]. Indeed, p21 is upregulated in response to interferon gamma (IFN-γ) [22], a pro-inflammatory cytokine that acts as a potent inhibitor of HIV-1 [23].

Consistent with p21 being an NRF2-responsive protein, SFN relies on NRF2 to promote both p21 upregulation and reduce SAMHD1 T592 phosphorylation in primary macrophages. Furthermore, a systematic screen of candidate cyclins, CDKs, CDK inhibitors, and phosphatases revealed that p21 was uniquely induced by SFN at non-toxic concentrations, whereas other SAMHD1 regulators remained largely unchanged [10]. We therefore hypothesized that SFN acts through p21 to reduce SAMHD1 T592 phosphorylation in macrophages. Here, we use RNAi, CRISPR-Cas9, and pharmacological inhibition to deplete or delete p21 in macrophages and demonstrate that p21 is necessary for SFN to efficiently reduce SAMHD1 T592 phosphorylation and restrict HIV-1 transduction.

## 2. Materials and Methods

### 2.1. Chemical Reagents

Sulforaphane (SFN) (Cat. No. S8044; LKT Laboratories, St. Paul, MN, USA) and UC2288 (Cat. No. 5.32813; Sigma-Aldrich, St. Louis, MO, USA) were dissolved in dimethyl sulfoxide (DMSO) (Cat. No. J66650K2; ThermoFisher Scientific, Waltham, MA, USA). Human IFN-γ (Cat. No. 11500; PBL Assay Science, Piscataway, NJ, USA) was diluted in phosphate-buffered saline (PBS).

### 2.2. Cell Culture

HEK293FT cells were obtained from Invitrogen, Carlsbad, CA, USA, (ThermoFisher Scientific) (Cat. No. R70007) and maintained in Dulbecco’s Modified Eagle Medium (DMEM) supplemented with 10% Fetal Bovine Serum (FBS), penicillin (10,000 IU/mL), streptomycin (10 mg/mL), and amphotericin B (25 μg/mL).

The 786-O adenocarcinoma renal cells were obtained from the American Type Culture Collection (ATCC), Manassas, VA, USA, (Cat. No. CRL-1932) and maintained in Roswell Park Memorial Institute 1640 (RPMI 1640) medium supplemented with 10% FBS, penicillin (10,000 IU/mL), streptomycin (10 mg/mL), and amphotericin B (25 μg/mL).

THP-1 monocytes were obtained from the ATCC (Cat. No. TIB-202) and maintained in RPMI 1640 supplemented with 10% FBS, penicillin (10,000 IU/mL), streptomycin (10 mg/mL), and amphotericin B (25 μg/mL). Stably transduced THP-1 monocytes were cultured similarly in medium supplemented with 10 µg/mL puromycin. THP-1 monocyte cultures were differentiated into a macrophage-like state by incubation in media supplemented with 5 ng/mL phorbol myristate acetate (PMA) for 3 days.

Elutriated human monocytes were obtained from healthy donors at the University of Nebraska Medical Center (Omaha, NE, USA). The monocytes were differentiated into macrophages (hMDM) via plastic adherence with incubation in serum-free DMEM for 2 h, followed by a 7-day incubation in DMEM supplemented with 10% human AB serum (to provide M-CSF and other factors), penicillin (10,000 IU/mL), streptomycin (10 mg/mL), and amphotericin B (25 μg/mL).

All cell cultures were incubated at 37 °C with 5% CO_2_ in a humidified chamber.

### 2.3. siRNA Transfection

Non-targeting siRNA #1 (Cat. No. D-001810-01), non-targeting siRNA #2 (Cat. No. D-001810-02-05), and p21 (CDKN1A) siRNAs #1–4 (Cat. No. LQ-003471-00-0002), were obtained from Horizon Discovery Biosciences Limited, Cambridge, UK. 20 µM siRNA stock was combined with Lipofectamine RNAiMAX Transfection Reagent (Cat. No. 13778100, ThermoFisher Scientific) in Opti-MEM I Reduced Serum Medium (Cat. No. 31985070, ThermoFisher Scientific) in accordance with the protocol provided by ThermoFisher Scientific. The siRNA transfection mix was added to cells to achieve a final concentration of 20 nM siRNA (20 pmol) in Opti-MEM I Reduced Serum Medium. Twelve hours post-transfection, the medium was replaced with normal growth medium (described in Section 2.2). 3.5 days post-transfection the siRNA transfection was repeated and the medium was replaced 12 h post-transfection with downstream experimentation beginning at day 7 post-initial siRNA transfection.

### 2.4. Immunoblotting and Densitometric Analyses

Cells were lysed with Laemmli buffer (50 mM Tris-HCl [pH 6.8], 2% SDS, 10% glycerol, and 0.1% bromophenol blue) and resolved by sodium dodecyl sulfate polyacrylamide gel electrophoresis (SDS-PAGE) using 7.5%, 12%, or 4–15% gradient polyacrylamide gels, depending on the molecular weight of the target protein. Proteins were transferred onto a polyvinylidene difluoride membrane (Cat. No. IPVH00010; Millipore Sigma, Burlington, MA, USA), blocked with 5% bovine serum albumin (BSA), and dissolved in wash buffer (0.1% *v*/*v* Tween 20 in 1× Tris-buffered saline) before three washes in wash buffer. The membranes were then incubated with primary antibody for 12 h at 4 °C on a rocking platform. The primary antibodies used in this work were anti-β-actin (Cat. No. 3700), anti-NRF2 (Cat. No. 12721), anti-p21 (CDKN1A/Waf1/Cip1) (Cat. No. 2947), anti-P-SAMHD1 (phosphorylation at threonine 592) (Cat. No. 89930), and anti-SAMHD1 (Cat. No. 49158) from Cell Signaling Technology, Inc., Danvers, MA, USA, and anti-P-p21 (phosphorylation at threonine 145) (Cat. No. PA5-37519) from ThermoFisher Scientific. All primary antibodies were diluted 1:1000 in wash buffer. After incubation, primary antibody was removed, and membranes were washed three times with wash buffer before incubation with horseradish peroxidase-conjugated secondary antibody. Secondary antibodies were diluted 1:2000 in wash buffer containing 5% *w*/*v* BSA or 5% *w*/*v* non-fat dry milk. The membranes were then incubated overnight at 4 °C on a rocking platform. The secondary antibodies used in this study were anti-mouse (Cat. No. 7076) and anti-rabbit (Cat. No. 7074) from Cell Signaling Technology, Inc. After incubation, the secondary antibody was removed, and the membranes were washed three times with wash buffer before the addition of an enhanced chemiluminescent substrate (Cat. No. 34094, ThermoFisher Scientific) and exposure. Blot imaging was done with a LiCore, Lincoln, NE, USA, C-Digit Scanner imaging system. Densitometric analyses were performed using ImageJ image analysis software [Version 1.52a].

### 2.5. Generation of p21 (CDKN1A) Knockout THP-1 Monocytic Cell Lines

CRISPR-Cas9-mediated disruption of *CDKN1A* in THP-1 monocytes was achieved using the protocol described in [24] with the following exceptions: the vesicular stomatitis virus envelope glycoprotein (VSV-G) and packaging plasmids used were pMD2.G (Addgene plasmid # 12259; http://n2t.net/addgene:12259, accessed on 12 August 2026; RRID:Addgene_12259) and psPAX2 (Addgene plasmid # 12260; http://n2t.net/addgene:12260, accessed on 12 August 2026; RRID:Addgene_12260) respectively. Both plasmids were gifts from Didier Trono. The gRNAs incorporated into the pCLIP-All-hCMV-Puro plasmid were obtained from Skyang Bio LLC (Huntsville, AL, USA): non-targeting gRNA “target” sequence: 5′ GGAGCGCACCATCTTCTTCA 3′, p21 (*CDKN1A*) gRNA #1 target sequence: 5′ GATGTCCGTCAGAACCCATG 3′, and p21 (*CDKN1A*) gRNA #2 target sequence: 5′ CCAAGCTCTACCTTCCCACG 3′.

### 2.6. HIV-1 Reporter Virus Generation and Transduction Analysis

Three million HEK293FT cells were co-transfected with an expression vector encoding the HIV-1 luciferase reporter clone, pNL4-3 *env*(−) *nef*(−) *luc*(+), a gift from Nathaniel Landau, and pMD2.G, encoding VSV-G (see Section 2.5) for pseudotyping HIV. Transfections were carried out using a standard calcium phosphate protocol with a total of 10 µg of DNA at an equimolar ratio of the viral expression vector to the VSV-G expression vector. Twelve hours after transfection, the cells were washed once with PBS and the medium (see Section 2.2) was replaced. 36 h after transfection, the supernatant was collected and cleared of producer cells by centrifugation. The cleared supernatant was then transferred to experimental cell cultures to initiate transduction. Twelve hours post-transduction, the supernatant was replaced with fresh medium. At 72 h post-transduction initiation, the cells were harvested and prepared for luciferase assays by using the Promega, Madison, WI, USA, luciferase assay kit (Cat. No. E501). The culture medium was removed, and cells were washed with PBS. Samples were lysed, and cellular debris was cleared by centrifugation at 18,000× *g* for 1 min. 80 µL of each sample was mixed with 20 µL of the luciferase assay reagent. Luciferase activity, in relative light units (RLU), was measured by photon emission using an Agilent, Santa Clara, CA, USA, BioTek Synergy HTX Multimode Plate Reader.

### 2.7. Subcellular Fractionation

Cytoplasmic and nuclear compartments were isolated using a modified detergent-lysis and sucrose-cushion density protocol. Five million cells were harvested, washed twice with ice-cold PBS, and resuspended in 1 mL of hypotonic Buffer B (10 mM HEPES [pH 7.9], 1.5 mM MgCl2, 10 mM KCl) supplemented with a protease inhibitor cocktail (Cat. No. 11836170001, Roche Diagnostics, Indianapolis, IN, USA). After a 20 min incubation on ice, cell membranes were disrupted by adding 100 μL of 1% NP-40 under continuous vortexing. The lysates were subsequently layered over a 1 mL cushion of 1 M sucrose (prepared in Buffer B) and sedimented at 3100× *g* for 10 min at 4 °C. To capture the cytoplasmic fraction, a 200 μL aliquot of the upper supernatant was collected. The remaining supernatant was aspirated, and the residual nuclear pellet was washed four times with Buffer B before final resuspension in 1 mL of Buffer B; a 200 μL aliquot of this suspension was retained as the nuclear fraction. Both isolated fractions were mixed with an equal volume of 2× Laemmli sample buffer and denatured at 94 °C for 30 min. Isolated fractions were assessed via SDS-PAGE and Western blotting, using anti-GAPDH (Cat. No. 5174) and anti-histone H3 (Cat. No. 9715), from Cell Signaling Technology Inc., to assess the purity of the cytoplasmic and nuclear fractions, respectively.

### 2.8. Cell Viability Analyses

Cell viability was assessed based on metabolic integrity (WST-8 assay) and membrane integrity (Trypan Blue exclusion). Metabolic activity was quantified using a WST-8 assay kit (Cat. No. CK04, Dojindo Molecular Technologies, Inc., Rockville, MD, USA). Macrophages were seeded in 24-well plates and treated with specified concentrations of SFN. 36 h following treatment, WST-8 reagent was added to each well at a 1:10 dilution and incubated at 37 °C for 4 h. After 4 h, 100 μL of media from each well was transferred to a corresponding well in a 96-well plate and absorbance was immediately measured at 450 nm using an Agilent BioTek Synergy HTX Multimode Plate Reader. Absorbance values were normalized to vehicle (DMSO)-treated control wells, which were defined as 100% viable.

Membrane integrity was determined in a parallel set of cultures 36 h after treatment with the specified concentrations of SFN. The medium in each well was aspirated, and macrophages were incubated in PBS mixed 1:1 with 0.4% Trypan Blue solution (Cat. No. 15250061, Gibco, Grand Island, NY, USA). After a 90 s incubation, the cells were washed and then fixed with 10% formalin solution. Cells were visualized by microscopy and images taken using an iPhone 13, Apple Inc., Cupertino, CA, USA, paired with a microscope lens cellphone adapter. Images were analyzed using Google Gemini (Gemini 1.5 Pro version) and confirmed by human analysis. Viable (unstained) and non-viable (blue-stained) cells were counted, and the percentage of dead cells was calculated relative to the total cell count for each condition.

### 2.9. Statistical Analyses

Statistical analyses were performed using GraphPad Prism [Version 11]. Quantitative data are expressed as the mean ± standard deviation (SD) from three independent experiments, or, where indicated, from replicate experiments. For multi-group comparisons evaluating a single independent variable (such as treatment-response assays), data were analyzed using an ordinary One-Way ANOVA (for THP-1 macrophages and 786-O cells) or a Repeated Measures One-Way ANOVA (for primary hMDMs, to account for donor-matched baseline variance), followed by Dunnett’s post hoc test for pairwise comparisons against the vehicle (DMSO) control. For multi-variable analyses involving two independent variables, data were evaluated using an ordinary or Repeated Measures Two-Way ANOVA. To isolate specific differences within these complex datasets, Sidak’s post hoc multiple comparisons test was applied for targeted pairwise dynamics (e.g., cellular compartment-by-treatment tracking), whereas Tukey’s post hoc test was utilized for comprehensive, all-to-all pairwise comparisons among combinatorial treatment groups. Data distribution and normality were evaluated using the Shapiro–Wilk test complemented by visual inspection of quantile-quantile (Q-Q) plots. A *p*-value of <0.05 was considered statistically significant. “*” = *p* < 0.05, “ns” = not significant.

## 3. Results

### 3.1. p21 Protein Levels Correlate with Reduced SAMHD1 T592 Phosphorylation in SFN Treated hMDMs but Not in SFN Treated THP-1-Derived Macrophages

**Figure 1:**
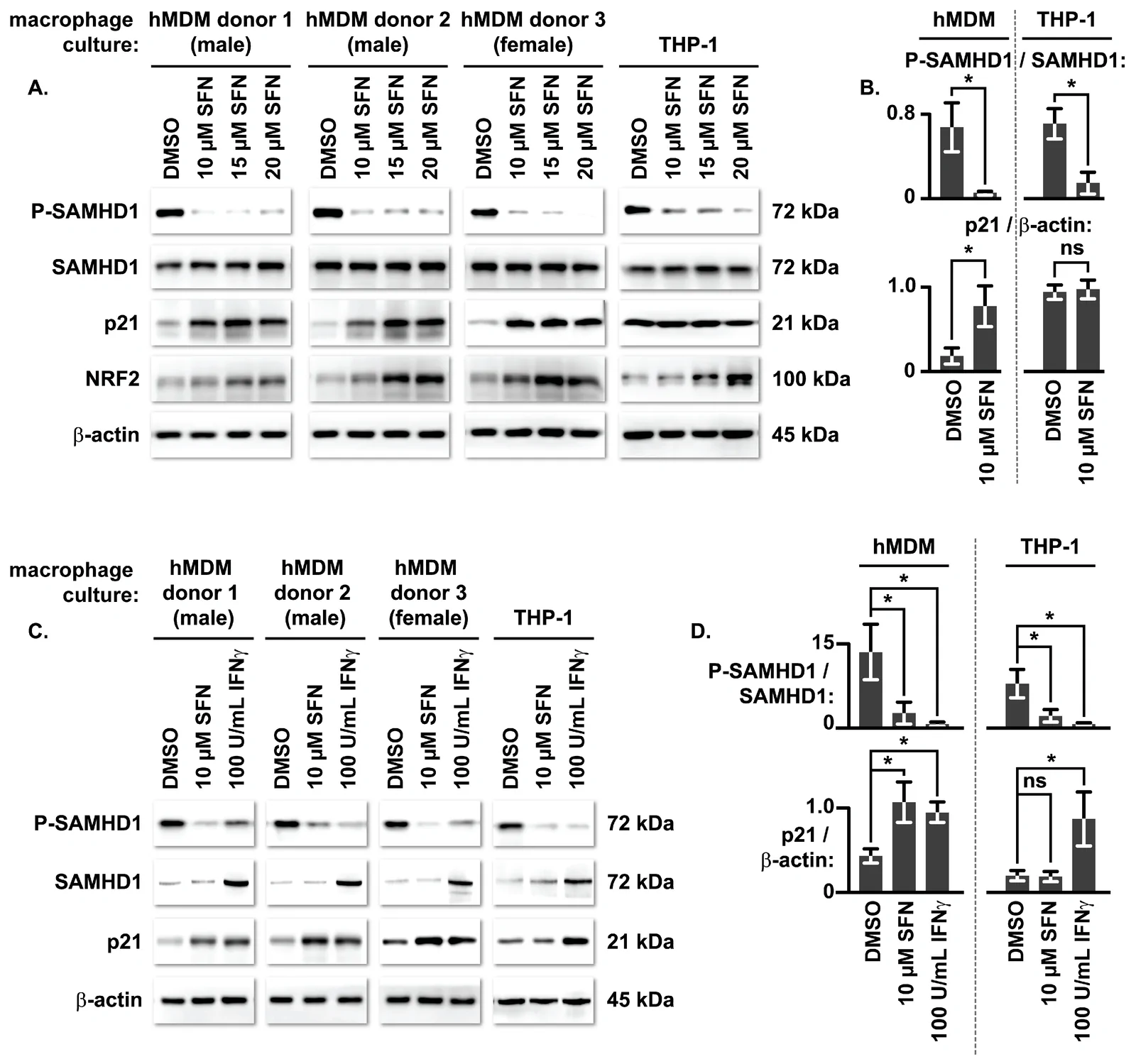
p21 protein levels correlate with reduced SAMHD1 T592 phosphorylation in SFN-treated hMDMs but not in SFN-treated THP-1-derived macrophages. (**A**) Primary hMDMs from three separate donors (two male, one female) and PMA-differentiated THP-1 macrophages were treated with vehicle (DMSO) or increasing concentrations of SFN (10–20 µM) for 24 h. After 24 h, the cells were lysed and proteins denatured. Lysate proteins were resolved via SDS-PAGE and analyzed by Western blot using antibodies specific to the indicated proteins. (**B**) Densitometric analysis representing the average relative ratio of T592 phosphorylated SAMHD1 (P-SAMHD1) to total SAMHD1, and p21 to the β-actin loading control for the data presented in panel (**A**). (**C**) Primary hMDMs and THP-1 macrophages were treated with vehicle (DMSO), 10 µM SFN, or 100 U/mL IFN-γ for 24 h. After 24 h, the cells were harvested and lysate proteins analyzed via Western blot. (**D**) Densitometric analysis of the ratios as described in (**B**) for the IFN-γ treatment setup. Data represents the mean ± SD of three independent experiments (*n* = 3). Statistical significance across multiple treatment groups was determined using a Repeated Measures One-Way ANOVA (for primary hMDMs) or an ordinary One-Way ANOVA (for THP-1 macrophages), followed by Dunnett’s post hoc multiple comparisons test to evaluate pairwise differences against the vehicle control. “*” = *p* < 0.05, “ns” = not significant. All original Western blot images can be found in Supplementary File S1.

SFN treatment led to an increase in p21 protein levels that correlated with reduced SAMHD1 T592 phosphorylation in hMDMs from three distinct donors (two male and one female). Interestingly, no change in p21 levels was observed in similarly treated THP-1 cell line-derived macrophages despite a decrease in SAMHD1 T592 phosphorylation (Figure 1A,B). The lack of p21 upregulation in SFN-treated THP-1 macrophages was not due to a general defect in p21 induction, as IFN-γ treatment upregulated p21 and reduced SAMHD1 T592 phosphorylation in both macrophage models (Figure 1C,D).

### 3.2. SFN Promotes the Accumulation of Cytosolic p21 in Macrophages

Unlike in hMDMs, p21 levels are not upregulated in response to SFN treatment in THP-1 cell line-derived macrophages, yet SFN reduces SAMHD1 T592 phosphorylation in both macrophage cell types. The SFN-mediated increase in p21 levels suggests a role for p21 in contributing to reduced SAMHD1 T592 phosphorylation in hMDMs. However, the lack of a similar increase in p21 in SFN-treated THP-1 macrophages puts into question the necessity of p21 for the effect. This discrepancy may be explained by SFN additionally acting to influence p21 activity in a manner that is independent of modulating total p21 levels. Through several complex molecular interplays, SFN and NRF2 activation are predicted to promote the cytosolic accumulation of p21. For example, SFN/NRF2 can enhance AKT activity [16,25,26], eventually leading to the phosphorylation of p21 at threonine 145 of the protein (T145) [27], a modification that promotes cytosolic retention [28]. We therefore tested whether SFN treatment influences the intracellular localization of p21 in macrophages. To do this, we treated cultures of hMDMs and THP-1 macrophages with 10 µM SFN for 24 h prior to performing fractionations to separate cytoplasmic and nuclear compartments.

**Figure 2:**
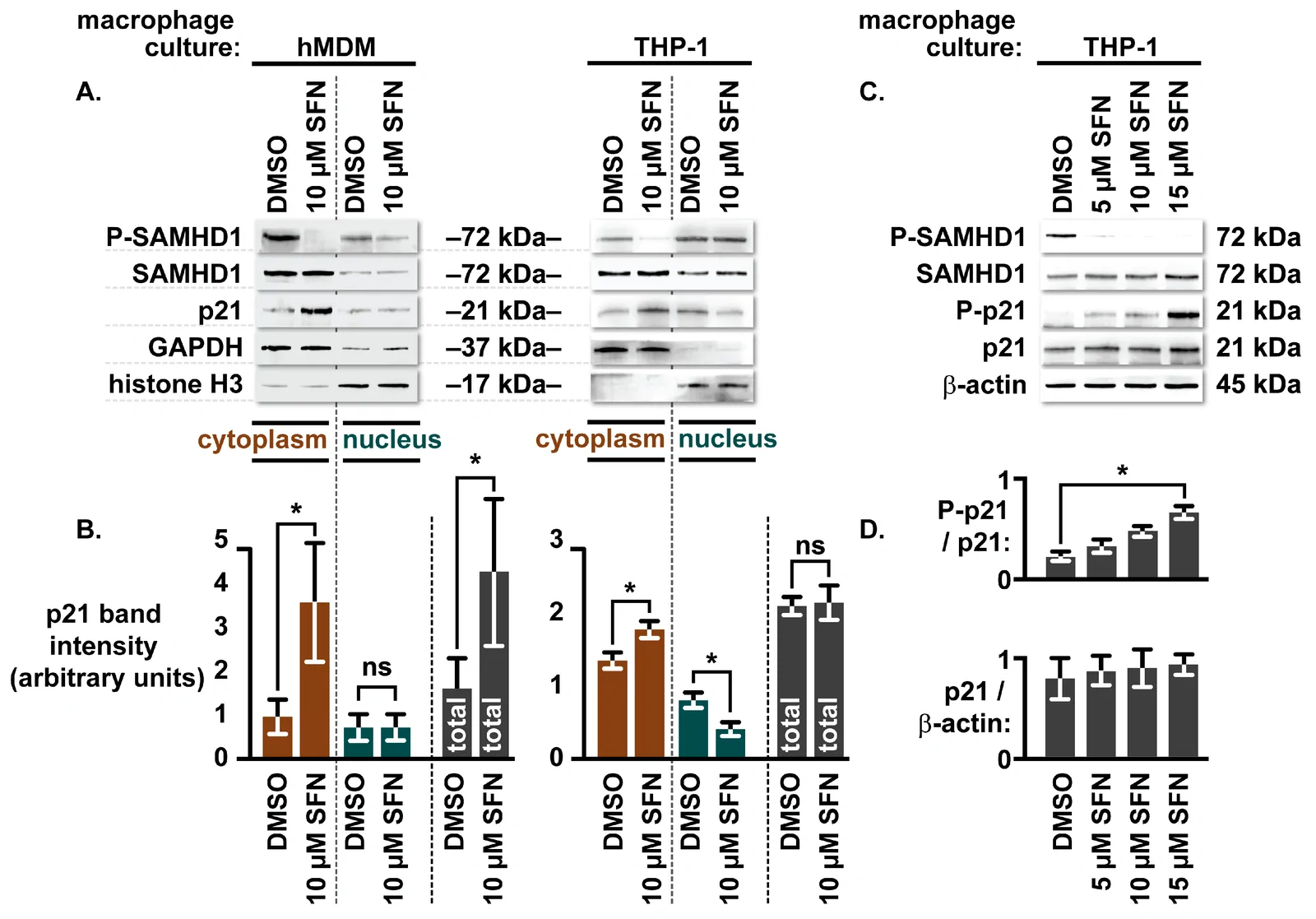
SFN promotes the accumulation of cytosolic p21 in macrophages. (**A**) Representative immunoblots of subcellular fractions from primary hMDMs and PMA-differentiated THP-1 macrophages treated with vehicle (DMSO) or 10 µM SFN for 24 h. Cytoplasmic and nuclear compartments were isolated via biochemical fractionation using a sucrose cushion matrix. Compartmental purity and separation efficiency were assessed by immunoblotted detection of GAPDH (cytosolic marker) and histone H3 (nuclear marker). (**B**) Densitometric quantification of cytosolic, nuclear, or combined total p21 protein signal for replicates of Western blots generated as described in (**A**). (**C**) PMA-differentiated THP-1 macrophages were treated with either vehicle (DMSO) or increasing concentrations of SFN (5–15 µM) for 24 h. After 24 h, the cells were lysed and proteins denatured. Lysate proteins were resolved via SDS-PAGE and analyzed by Western blot using antibodies specific to the indicated proteins. Data represents the mean ± SD of three independent human donors (two male and one female) (hMDMs) or three independent experiments (THP-1). Statistical variance across treatments and compartments was evaluated via a Repeated Measures (hMDM) or an ordinary (THP-1) Two-Way ANOVA followed by Sidak’s post hoc multiple comparisons test for the data presented in (**B**). Statistical significance was determined using a One-Way ANOVA followed by Dunnett’s post hoc multiple comparisons test for the data presented in (**D**). “*” = *p* < 0.05, “ns” = not significant. All original Western blot images can be found in Supplementary File S1.

Consistent with previous reports, endogenous SAMHD1 was readily detected in the cytosolic compartment of both macrophage types [10,29,30], and its distribution remained unchanged following SFN treatment relative to the DMSO control [10]. As expected, the SFN-mediated reduction in SAMHD1 T592 phosphorylation correlated with increased total p21 levels in hMDMs, but not in THP-1 macrophages. Importantly, SFN significantly increased the proportion of p21 within the cytosolic compartment of both cell types (Figure 2A,B). This observation was further supported by an increase in p21 T145 phosphorylation in SFN-treated THP-1 macrophages (Figure 2C,D). Collectively, these data favor a model in which SFN promotes the cytosolic accumulation of p21 to regulate SAMHD1 T592 phosphorylation.

### 3.3. siRNA-Mediated p21 Depletion Diminishes SFN’s Capacity to Reduce SAMHD1 T592 Phosphorylation in hMDMs

**Figure 3:**
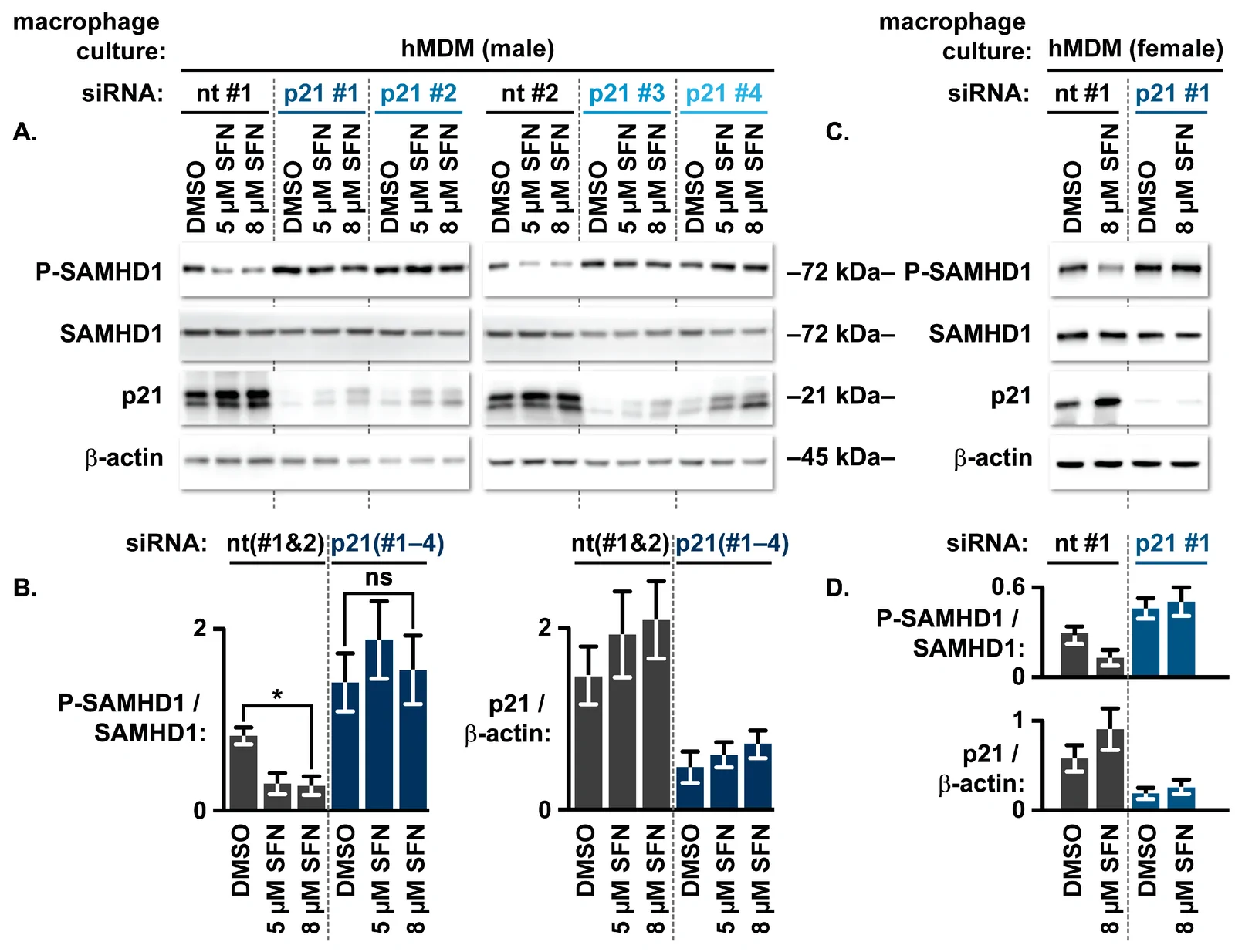
siRNA-mediated p21 depletion diminishes SFN’s capacity to reduce SAMHD1 T592 phosphorylation in hMDMs. (**A**) hMDMs from a male donor were transfected twice with two distinct non-targeting (nt) control siRNAs (#1 and #2) or four independent siRNAs targeting p21 (#’s 1–4). The initial transfection was performed simultaneously with the start of differentiation, and the second transfection was performed 3.5 days later. On day 7 post-initial siRNA transfection, the hMDMs were treated with vehicle (DMSO) or low concentrations of SFN (5 and 8 µM) for 24 h. After 24 h, the cells were lysed and proteins denatured. Lysate proteins were resolved via SDS-PAGE and analyzed by Western blot using antibodies specific to the indicated proteins. (**B**) Densitometric analysis of the Western blot data shown in (**A**). Values from individual siRNA sequences were pooled by target type. Data represents the mean ± SD of the individual siRNA sequences within this donor (*n* = 4 total replicates with 2 distinct sequences (#1 and #2) for nt; *n* = 4 total replicates with 4 distinct sequences (#’s 1–4) for p21). Values reflect the relative ratio of T592 phosphorylated SAMHD1 (P-SAMHD1) to total SAMHD1 protein levels. Statistical significance across the distinct siRNA sequences and drug treatments was evaluated via a Repeated Measures Two-Way ANOVA followed by Dunnett’s post hoc multiple comparisons test relative to the non-targeting control. “*” = *p* < 0.05, “ns” = not significant. (**C**) Western blot data of hMDMs from a second donor (female) transfected as described in (**A**), with a single nt control siRNA (#1) or a single p21-targeting siRNA (#1), treated with DMSO or 8 µM SFN for 24 h. (**D**) Densitometric analysis of the P-SAMHD1 to total SAMHD1 ratio for the second donor (female), included to demonstrate and confirm the biological trend observed in the first donor (male). Data represents the mean ± SD of replicate experiments for this donor (*n* = 2). All original Western blot images can be found in Supplementary File S1.

Given the capacity of SFN to promote both p21 upregulation and cytosolic accumulation in hMDMs, we sought to determine whether p21 is required for SFN to reduce SAMHD1 T592 phosphorylation. To test this, we targeted p21 mRNA using siRNA transfection. Although p21 is robustly expressed during terminal monocyte-to-macrophage differentiation [12], it is not strictly required for the process itself [31]. Therefore, to achieve sustained p21 depletion while minimizing the off-target immune activation associated with high-dose siRNA delivery (reviewed in [32]), we optimized a dual-transfection protocol with the first transfection at the onset of differentiation and a second 3.5 days later. This sequential approach achieved an appreciable reduction in p21 levels across four independent siRNAs. This reduction in p21 levels was associated with an increase in steady-state SAMHD1 T592 phosphorylation. Critically, p21 depletion was sufficient to abrogate the effect of low-dose SFN on reducing SAMHD1 T592 phosphorylation, implicating p21 as a mediator of SFN-induced SAMHD1 regulation in hMDMs (Figure 3).

### 3.4. SFN Fails to Reduce SAMHD1 T592 Phosphorylation and Restrict HIV-1 Transduction in CRISPR-Cas9-Edited THP-1 Macrophages Lacking p21

**Figure 4:**
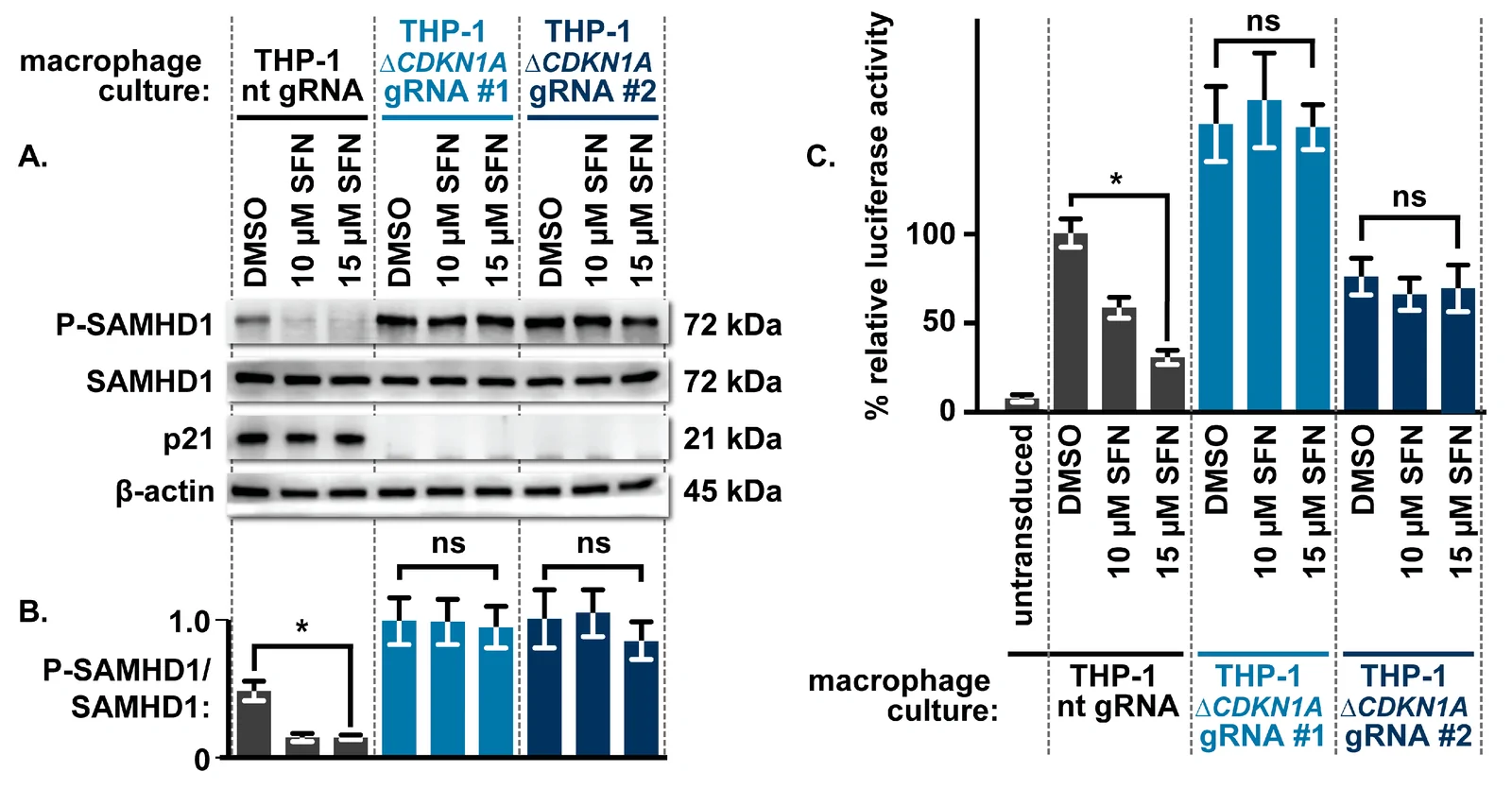
SFN fails to reduce SAMHD1 T592 phosphorylation and restrict HIV-1 transduction in CRISPR-Cas9-edited THP-1 macrophages lacking p21. (**A**) Control THP-1 macrophages (expressing a non-targeting gRNA) and two independent lines of p21-knockout THP-1 macrophages (generated using distinct gRNAs, #1 and #2) (THP-1^Δ*CDKN1A*^) were treated with vehicle (DMSO) or increasing concentrations of SFN (10–15 µM) for 24 h. After 24 h, the cells were lysed and proteins denatured. Lysate proteins were resolved via SDS-PAGE and analyzed by Western blot using antibodies specific to the indicated proteins. (**B**) Densitometric analysis from replicate Western blots generated as described in (**A**) showing the average relative ratio of T592 phosphorylated SAMHD1 (P-SAMHD1) to total SAMHD1. (**C**) Control and THP-1^Δ*CDKN1A*^ macrophages were pre-treated with vehicle (DMSO) or SFN for 24 h, washed to remove residual DMSO/SFN, and transduced with a VSV-G-pseudotyped HIV-1 luciferase reporter virus. Luciferase activity was quantified from cell lysates 72 h post-transduction to assess viral permissiveness. Data represents the mean ± SD of three independent experiments (*n* = 3). Statistical significance was determined using a Two-Way ANOVA followed by Tukey’s post hoc multiple comparisons test. “*” = *p* < 0.05, “ns” = not significant. All original Western blot images can be found in Supplementary File S1.

To best assess the impact of p21 on SFN’s capacity to reduce SAMHD1 T592 phosphorylation in macrophages, we generated THP-1 monocytes wherein the gene encoding p21 (*CDKN1A*) was disrupted via CRISPR-Cas9 editing (hereafter referred to as THP-1^Δ*CDKN1A*^). Two sets of THP-1^Δ*CDKN1A*^ cells were generated, each using a distinct gRNA targeting a different region of the *CDKN1A* locus. p21 protein was not detected in either set of THP-1^Δ*CDKN1A*^-derived macrophages relative to control THP-1 macrophages that were generated similarly but express a non-targeting gRNA. As expected, steady-state levels of T592 phosphorylated SAMHD1 were higher in THP-1^Δ*CDKN1A*^ macrophages relative to control THP-1 macrophages, consistent with a loss of p21 function. Importantly, whereas SFN treatment led to a 3–4-fold decrease in the ratio of T592 phosphorylated SAMHD1 to total SAMHD1 in control THP-1 macrophages, SFN failed to similarly decrease the ratio of T592 phosphorylated SAMHD1 to total SAMHD1 in either set of THP-1^Δ*CDKN1A*^ macrophages (Figure 4A,B).

Having demonstrated that loss of p21 effectively abrogates SFN’s capacity to reduce SAMHD1 T592 phosphorylation in THP-1 macrophages, we next tested whether this outcome would translate to HIV-1 transduction. To test this, control THP-1 macrophages and THP-1^Δ*CDKN1A*^ macrophages were treated with vehicle (DMSO) or SFN for 24 h. After 24 h, the cells were washed to remove residual DMSO/SFN and transduced with a VSV-G-pseudotyped HIV-1 luciferase reporter virus. Seventy-two hours after transduction, luciferase activity was quantified to assess transduction efficiency. Whereas SFN treatment led to a 50–75% decrease in luciferase activity in transduced control THP-1 macrophages, SFN failed to decrease luciferase activity in similarly treated THP-1^Δ*CDKN1A*^ macrophages (Figure 4C). The inability of SFN to impair HIV-1 transduction in THP-1^Δ*CDKN1A*^ macrophages mirrors similar results in macrophages lacking SAMHD1 [10].

At SFN concentrations exceeding 20 µM, we observed a modest reduction in total SAMHD1 levels and a modest decrease in the ratio of T592-phosphorylated SAMHD1 to total SAMHD1 in THP-1^Δ*CDKN1A*^ macrophages. This correlated with a decrease in luciferase activity post-HIV-1 reporter virus transduction, suggesting that higher SFN concentrations may trigger secondary mechanisms to reduce SAMHD1 T592 phosphorylation. In hMDMs, for example, high-dose SFN resulted in decreased Cyclin D3 and CDK4 levels [10]. Alternatively, these observations may reflect confounding cytotoxicity, as SFN concentrations above 20 µM are known to be toxic to monocytic immune lineages [33,34,35]. Indeed, we observed loss of viability in control THP-1 macrophages treated with SFN at concentrations greater than 20 µM, an effect that was exacerbated in the THP-1^Δ*CDKN1A*^ macrophages (Figure S1), potentially due to the loss of p21-mediated anti-apoptotic signaling [22,36]. Nonetheless, our findings demonstrate that p21 is required for SFN to efficiently protect macrophages from HIV-1 transduction through reduced SAMHD1 T592 phosphorylation at physiologically relevant [37,38,39], non-toxic concentrations.

### 3.5. UC2288 Reduces SAMHD1 Phosphorylation and Occludes Additive Effects of SFN in Macrophages

Having established genetically that p21 is necessary for SFN to efficiently reduce SAMHD1 T592 phosphorylation, we next sought to complement these findings using a pharmacological strategy. To do this, we treated hMDMs and THP-1 cell line-derived macrophages with UC2288. UC2288 is described as a selective inhibitor of p21 that predominantly acts to suppress transcription of the *CDKN1A* gene, eventually leading to loss of p21 protein as a consequence of normal turnover [40]. hMDMs and THP-1 macrophages were treated with UC2288 at concentrations ranging from 5 to 15 µM for 24 h prior to harvesting the cells for Western blot analysis. The concentrations used reflect the range established in several studies [40,41,42]. Relative to the control 786-O renal cell adenocarcinoma line [40], we observed a modest decrease in p21 levels in hMDMs and THP-1 macrophages treated with UC2288. Interestingly, UC2288 treatment alone led to a reduction in SAMHD1 T592 phosphorylation (Figure S2A,B), a finding that is counterintuitive, as the loss of p21 or p21 function would be expected to increase steady-state SAMHD1 T592 phosphorylation. This decrease may reflect ERK inhibition [42] or other residual kinase inhibition inherent to UC2288’s parent compound, sorafenib [43]. Notably, SFN could not further reduce SAMHD1 T592 phosphorylation in macrophages pretreated with UC2288 (Figure S2C,D), though it is unclear whether this is related to a potential inhibition of p21 function.

## 4. Discussion

Our findings establish p21 as the major mediator by which SFN promotes reduced SAMHD1 T592 phosphorylation in macrophages. At non-toxic concentrations, the ability of SFN to modulate SAMHD1 is contingent upon p21 abundance, as siRNA-mediated depletion in hMDMs (Figure 3) or CRISPR-Cas9 ablation in THP-1 macrophages (Figure 4) abrogates this effect. This requirement is functionally underscored by the failure of SFN to restrict HIV-1 transduction in p21-deficient THP-1 macrophages (Figure 4C), a result that phenocopies the loss of restriction observed in SAMHD1-knockout macrophages [10].

While SFN induced an increase in p21 levels in hMDMs, such accumulation was absent in THP-1-derived macrophages (Figure 1). We posit that this divergence stems from the p53-null status of the THP-1 cell line, as p53 serves as the primary transcriptional activator of p21 (*CDKN1A*). Although the crosstalk between NRF2, p53, and p21 is complex and context-specific (reviewed in [17,44]), NRF2 activation can modulate p53 activity through multiple mechanisms (reviewed in [44,45]). For example, through downstream targets such as NQO1, NRF2 can stabilize p53 to facilitate p21 induction [46]. Furthermore, p53 contains redox-sensitive cysteine residues that promote DNA binding under the reducing conditions favored by NRF2 activation [47]. Once induced, p21 can sustain the antioxidant response by competing with KEAP1 to prevent NRF2 degradation [48], and further stabilize p53 [49]. Such reciprocal stabilization establishes a cooperative positive feedback loop that likely drives the robust p21 accumulation observed in hMDMs in response to SFN. Our proposed p53-dependent mechanism is supported by our IFN-γ data; IFN-γ, which induces p21 via p53-independent JAK-STAT1 signaling [22,50], successfully upregulated p21 in both macrophage models (Figure 1C,D). The IFN-γ response confirms that the downstream transcriptional machinery for p21 expression remains intact in THP-1 cells, implicating the absence of functional p53 as the bottleneck for SFN-mediated induction.

**Figure 5:**
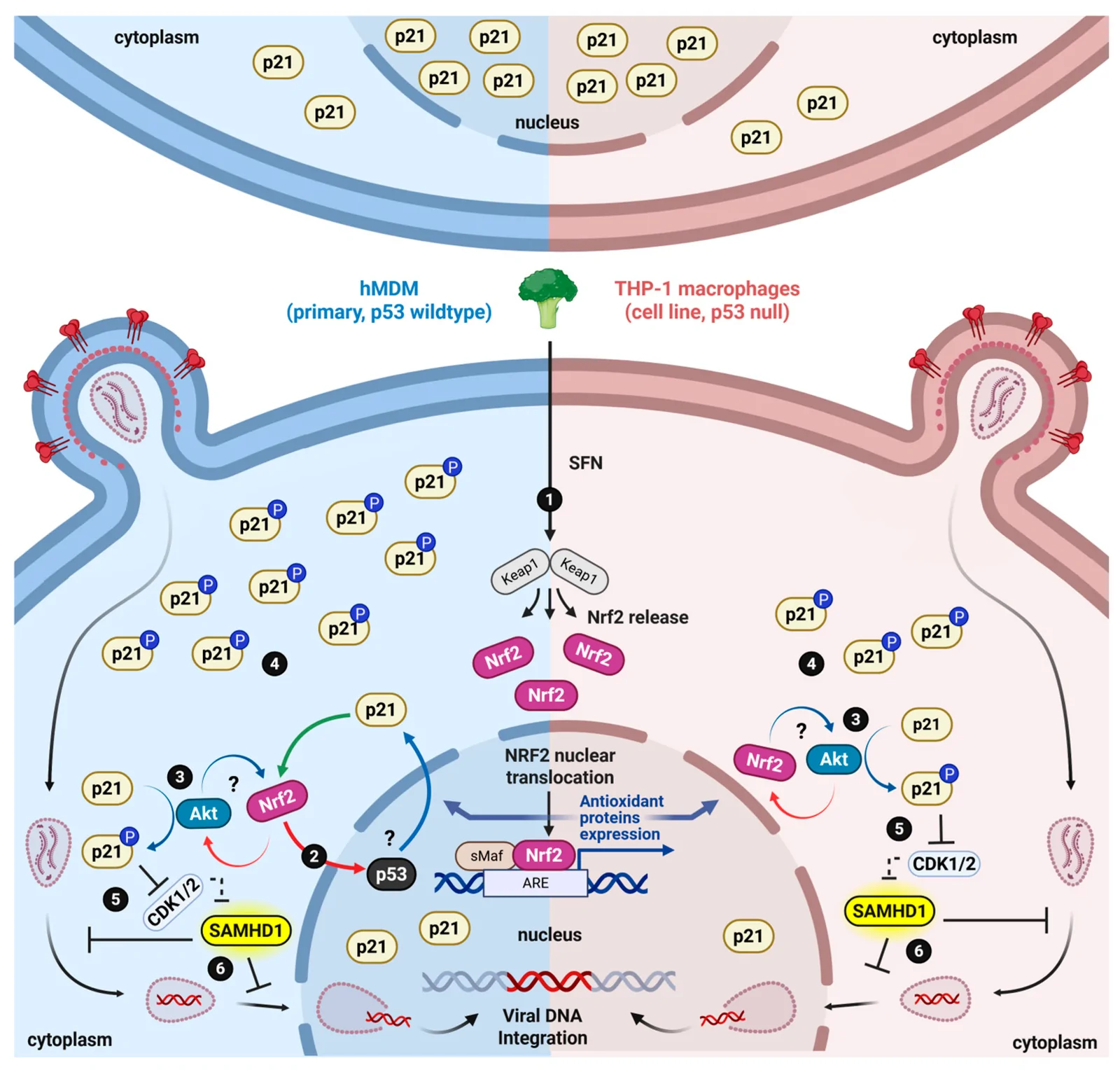
Proposed model of SFN-mediated SAMHD1 regulation via transcriptional and spatial modulation of p21. In primary hMDMs, SFN activates NRF2 (1), which likely coordinates with wild-type p53 to transcriptionally induce p21 expression. This process is reinforced by a cooperative positive feedback loop where induced p21 competes with KEAP1 to further prevent NRF2 degradation, while simultaneously stabilizing p53 (2). Conversely, in THP-1-derived macrophages, the absence of functional p53 creates a bottleneck that hinders SFN-mediated p21 transcriptional upregulation. Despite transcriptional differences, SFN likely stimulates an NRF2-AKT signaling axis in both hMDMs and THP-1 macrophages, triggering the phosphorylation of p21 at T145 (3). This modification drives the spatial redistribution and accumulation of p21 within the cytosolic compartment (4). Enhanced cytosolic enrichment of p21 likely blocks CDK-mediated phosphorylation of endogenous cytosolic SAMHD1 at T592 (5). Maintaining SAMHD1 in its unphosphorylated, “active”, state ultimately restricts HIV-1 transduction (6). “?” indicates mechanisms supported by literature and partially by our data. This figure was created in BioRender. Sharifi, J. (2026) https://BioRender.com/w9wrb29, accessed on 12 August 2026.

Despite the lack of SFN-induced p21 upregulation in THP-1 macrophages, p21 is nonetheless required for SFN to reduce SAMHD1 T592 phosphorylation in these cells (Figure 4A,B). This suggests an additional mechanism by which SFN modulates p21 activity. Indeed, we observed accumulation of p21 in the cytosol of both hMDMs and THP-1 macrophages following SFN treatment (Figure 2A,B). This cytosolic shift is consistent with the promotion of an NRF2-AKT signaling axis [16,25,26], a hypothesis further supported by our observation of increased p21 phosphorylation at T145 [27,28] (Figure 2C,D). The enrichment of p21 within the cytosolic compartment likely facilitates the inhibition of CDK-mediated phosphorylation of cytosolic SAMHD1. Although SAMHD1 is historically categorized as a nuclear protein, endogenous SAMHD1 is readily detected in the cytosol of primary immune cells, including macrophages [10,29,30], and nuclear localization is not a prerequisite for its retroviral restriction activity [51,52]. Consequently, even in p53-null THP-1 macrophages, SFN promotes reduced SAMHD1 T592 phosphorylation by driving the spatial redistribution of p21. More broadly, this highlights the importance of evaluating both primary cells and transformed cell line models, as comparing these systems can unmask complementary mechanisms within a single regulatory axis. Future studies aimed at fully characterizing the molecular details of this proposed model (Figure 5) will be of interest.

Beyond influencing HIV replication, SAMHD1 regulates genome integrity, innate immunity, and chemotherapy responses. At stalled replication forks, SAMHD1 T592 phosphorylation promotes MRE11-dependent processing to restart DNA synthesis [53]. This function prevents the cytosolic accumulation of DNA fragments that trigger cGAS–STING inflammatory signaling [54]. In oncology, SAMHD1 drives chemoresistance by hydrolyzing antimetabolite drugs like cytarabine [55], a dNTPase activity that T592 phosphorylation can suppress in specific contexts [56,57]. Because NRF2 and p21 control CDK activity, modulating this axis may concurrently influence these broader SAMHD1 roles. Importantly, SFN fails to reduce SAMHD1 T592 phosphorylation in primary activated CD4+ T cells [10] or impact HIV replication in dividing cell lines [8,9]. This specificity may reflect lower baseline NRF2 levels, higher steady-state SAMHD1 T592 phosphorylation [9,10] and potentially reduced p21 function in proliferating cells compared to macrophages. Consequently, influencing NRF2-p21 activation to modulate these other SAMHD1 functions may depend on cell lineage and differentiation state.

## 5. Conclusions

The key finding of this work demonstrates that p21 is required for SFN to effectively reduce SAMHD1 T592 phosphorylation and protect macrophages from HIV-1 transduction. The data presented here build upon previous work establishing SAMHD1 as the conduit by which SFN protects macrophages from HIV-1 and provide mechanistic insight into the observed correlation of SFN treatment with reduced SAMHD1 T592 phosphorylation in macrophages.

## Data Availability

The original contributions presented in this study are included in the article/Supplementary Materials. Further inquiries can be directed to the corresponding author.

## Supplementary Materials

The following supporting information can be downloaded at: https://www.mdpi.com/article/10.3390/biom16081213/s1, File S1: Original Western blot Images; File S2: Excel Files; File S3: Statistical Analyses; File S4: Additional Figures and Tables, Figure S1: Effects of high-dose SFN on control and Δ*CDKN1A* THP-1 macrophages; Figure S2: UC2288 reduces SAMHD1 phosphorylation and occludes additive effects of SFN in macrophages.

## Abbreviations

The following abbreviations are used in this manuscript:

ATCC	American Type Culture Collection
BSA	Bovine Serum Albumin
CDK	Cyclin-Dependent Kinase
CDKN1A	Cyclin-Dependent Kinase Inhibitor 1A (p21)
cGAS-STING	Cyclic GMP-AMP Synthase-Stimulator of Interferon Genes
CRISPR	Clustered Regularly Interspaced Short Palindromic Repeats
DMEM	Dulbecco’s Modified Eagle Medium
DMSO	Dimethyl Sulfoxide
DNA	Deoxyribonucleic Acid
ERK	Extracellular Signal-Regulated Kinase
FBS	Fetal Bovine Serum
GAPDH	Glyceraldehyde 3-Phosphate Dehydrogenase
gRNA	guide RNA
HEK293FT	Human Embryonic Kidney 293 Fast-growing, SV40 Large T-antigen transformed
HIV-1	Human Immunodeficiency Virus Type 1
hMDM	human Monocyte-Derived Macrophage
IFN-γ	Interferon Gamma
JAK-STAT1	Janus Kinases and Signal Transducer and Activators of Transcription 1
KEAP1	Kelch-Like ECH-Associated Protein 1
M-CSF	Macrophage Colony-Stimulating Factor
MRE11	Meiotic Recombination Enzyme 11
NRF2	Nuclear Factor Erythroid 2-Related Factor 2
PBS	Phosphate-Buffered Saline
PMA	Phorbol 12-Myristate 13-Acetate
P-SAMHD1	Phosphorylation of SAMHD1 at T592
RNAi	Ribonucleic Acid interference
RPMI 1640	Roswell Park Memorial Institute 1640
SAMHD1	Sterile Alpha Motif Histidine (H) and Aspartate (D) Domain-Containing Protein 1
SDS-PAGE	Sodium Dodecyl Sulfate Polyacrylamide Gel Electrophoresis
SFN	Sulforaphane
siRNA	Small Interfering RNA
T145	Threonine at position 145 of p21
T592	Threonine at position 592 of SAMHD1
VSV-G	Vesicular Stomatitis Virus Glycoprotein
WST-8	Water-Soluble Tetrazolium

## References

[1] Cheung P.-H.H., Yang H., Wu L. (2025). DNTP Depletion and beyond: The Multifaceted Nature of SAMHD1-Mediated Viral Restriction. J. Virol..

[2] Dobransky A., Root M., Hafner N., Marcum M., Sharifi H.J. (2024). CRL4-DCAF1 Ubiquitin Ligase Dependent Functions of HIV Viral Protein R and Viral Protein X. Viruses.

[3] Deutschmann J., Gramberg T. (2021). SAMHD1 … and Viral Ways around It. Viruses.

[4] Coggins S.A., Mahboubi B., Schinazi R.F., Kim B. (2020). SAMHD1 Functions and Human Diseases. Viruses.

[5] Schott K., Majer C., Bulashevska A., Childs L., Schmidt M.H.H., Rajalingam K., Munder M., König R. (2022). SAMHD1 in Cancer: Curse or Cure?. J. Mol. Med..

[6] Chen Z., Hu J., Ying S., Xu A. (2021). Dual Roles of SAMHD1 in Tumor Development and Chemoresistance to Anticancer Drugs. Oncol. Lett..

[7] Dinkova-Kostova A.T., Fahey J.W., Kostov R.V., Kensler T.W. (2017). KEAP1 and Done? Targeting the NRF2 Pathway with Sulforaphane. Trends Food Sci. Technol..

[8] Furuya A.K., Sharifi H.J., Jellinger R.M., Cristofano P., Shi B., de Noronha C.M. (2016). Sulforaphane Inhibits HIV Infection of Macrophages through Nrf2. PLoS Pathog..

[9] Rabinowitz J., Sharifi H.J., Martin H., Marchese A., Robek M., Shi B., Mongin A.A., de Noronha C.M.C. (2021). XCT/SLC7A11 Antiporter Function Inhibits HIV-1 Infection. Virology.

[10] Sharifi H.J., Paine D.N., Fazzari V.A., Tipple A.F., Patterson E., de Noronha C.M.C. (2022). Sulforaphane Reduces SAMHD1 Phosphorylation To Protect Macrophages from HIV-1 Infection. J. Virol..

[11] Kreis N.-N., Louwen F., Yuan J. (2019). The Multifaceted P21 (Cip1/Waf1/CDKN1A) in Cell Differentiation, Migration and Cancer Therapy. Cancers.

[12] Asada M., Yamada T., Ichijo H., Delia D., Miyazono K., Fukumuro K., Mizutani S. (1999). Apoptosis Inhibitory Activity of Cytoplasmic P21(Cip1/WAF1) in Monocytic Differentiation. EMBO J..

[13] Rackov G., Hernández-Jiménez E., Shokri R., Carmona-Rodríguez L., Mañes S., Álvarez-Mon M., López-Collazo E., Martínez-A C., Balomenos D. (2016). P21 Mediates Macrophage Reprogramming through Regulation of P50-P50 NF-ΚB and IFN-β. J. Clin. Investig..

[14] Murray P.J. (2017). Macrophage Polarization. Annu. Rev. Physiol..

[15] Yunna C., Mengru H., Lei W., Weidong C. (2020). Macrophage M1/M2 Polarization. Eur. J. Pharmacol..

[16] Wang L., He C. (2022). Nrf2-Mediated Anti-Inflammatory Polarization of Macrophages as Therapeutic Targets for Osteoarthritis. Front. Immunol..

[17] Villeneuve N.F., Sun Z., Chen W., Zhang D.D. (2009). Nrf2 and P21 Regulate the Fine Balance between Life and Death by Controlling ROS Levels. Cell Cycle.

[18] Yuan H., Xu Y., Luo Y., Wang N.-X., Xiao J.-H. (2021). Role of Nrf2 in Cell Senescence Regulation. Mol. Cell. Biochem..

[19] Trakala M., Arias C.F., García M.I., Moreno-Ortiz M.C., Tsilingiri K., Fernández P.J., Mellado M., Díaz-Meco M.T., Moscat J., Serrano M. (2009). Regulation of Macrophage Activation and Septic Shock Susceptibility via P21(WAF1/CIP1). Eur. J. Immunol..

[20] Pauls E., Ruiz A., Riveira-Munoz E., Permanyer M., Badia R., Clotet B., Keppler O.T., Ballana E., Este J.A. (2014). P21 Regulates the HIV-1 Restriction Factor SAMHD1. Proc. Natl. Acad. Sci. USA.

[21] Valle-Casuso J.C., Allouch A., David A., Lenzi G.M., Studdard L., Barré-Sinoussi F., Müller-Trutwin M., Kim B., Pancino G., Sáez-Cirión A. (2017). P21 Restricts HIV-1 in Monocyte-Derived Dendritic Cells through the Reduction of Deoxynucleoside Triphosphate Biosynthesis and Regulation of SAMHD1 Antiviral Activity. J. Virol..

[22] Hobeika A.C., Etienne W., Torres B.A., Johnson H.M., Subramaniam P.S. (1999). IFN-Gamma Induction of P21(WAF1) Is Required for Cell Cycle Inhibition and Suppression of Apoptosis. J. Interf. Cytokine Res..

[23] Ennen J., Kurth R. (1993). Interferon-Gamma-Activated Monocytes Impair Infectivity of HIV Particles by an Oxygen Metabolite-Dependent Reaction. Immunology.

[24] Deragon M.A., Sharifi H.J., LaRocca T.J. (2025). Generation of a RIP1 Knockout U937 Cell Line Using the CRISPR-Cas9 System. J. Vis. Exp..

[25] Wu M., Gibbons J.G., DeLoid G.M., Bedugnis A.S., Thimmulappa R.K., Biswal S., Kobzik L. (2017). Immunomodulators Targeting MARCO Expression Improve Resistance to Postinfluenza Bacterial Pneumonia. Am. J. Physiol. Lung Cell. Mol. Physiol..

[26] Wu Y., Gao M., Wu J., Hu P., Xu X., Zhang Y., Wang D., Chen Z., Huang C. (2019). Sulforaphane Triggers a Functional Elongation of Microglial Process via the Akt Signal. J. Nutr. Biochem..

[27] Li Y., Dowbenko D., Lasky L.A. (2002). AKT/PKB Phosphorylation of P21Cip/WAF1 Enhances Protein Stability of P21Cip/WAF1 and Promotes Cell Survival. J. Biol. Chem..

[28] Zhou B.P., Liao Y., Xia W., Spohn B., Lee M.H., Hung M.C. (2001). Cytoplasmic Localization of P21Cip1/WAF1 by Akt-Induced Phosphorylation in HER-2/Neu-Overexpressing Cells. Nat. Cell Biol..

[29] Baldauf H.-M., Pan X., Erikson E., Schmidt S., Daddacha W., Burggraf M., Schenkova K., Ambiel I., Wabnitz G., Gramberg T. (2012). SAMHD1 Restricts HIV-1 Infection in Resting CD4(+) T Cells. Nat. Med..

[30] Sharifi H.J., Furuya A.K.M., Jellinger R.M., Nekorchuk M.D., de Noronha C.M.C. (2014). Cullin4A and Cullin4B Are Interchangeable for HIV Vpr and Vpx Action through the CRL4 Ubiquitin Ligase Complex. J. Virol..

[31] Merched A.J., Chan L. (2004). Absence of P21Waf1/Cip1/Sdi1 Modulates Macrophage Differentiation and Inflammatory Response and Protects against Atherosclerosis. Circulation.

[32] Jackson A.L., Linsley P.S. (2010). Recognizing and Avoiding SiRNA Off-Target Effects for Target Identification and Therapeutic Application. Nat. Rev. Drug Discov..

[33] Moon D.-O., Kim M.-O., Kang S.-H., Choi Y.H., Kim G.-Y. (2009). Sulforaphane Suppresses TNF-Alpha-Mediated Activation of NF-KappaB and Induces Apoptosis through Activation of Reactive Oxygen Species-Dependent Caspase-3. Cancer Lett..

[34] Ruhee R.T., Ma S., Suzuki K. (2019). Sulforaphane Protects Cells against Lipopolysaccharide-Stimulated Inflammation in Murine Macrophages. Antioxidants.

[35] Fernandez-Prades L., Brasal-Prieto M., Alba G., Martin V., Montserrat-de la Paz S., Cejudo-Guillen M., Santa-Maria C., Dakhaoui H., Granados B., Sobrino F. (2023). Sulforaphane Reduces the Chronic Inflammatory Immune Response of Human Dendritic Cells. Nutrients.

[36] Blázquez-Prieto J., Huidobro C., López-Alonso I., Amado-Rodriguez L., Martín-Vicente P., López-Martínez C., Crespo I., Pantoja C., Fernandez-Marcos P.J., Serrano M. (2021). Activation of P21 Limits Acute Lung Injury and Induces Early Senescence after Acid Aspiration and Mechanical Ventilation. Transl. Res..

[37] Livingstone T.L., Saha S., Bernuzzi F., Savva G.M., Troncoso-Rey P., Traka M.H., Mills R.D., Ball R.Y., Mithen R.F. (2022). Accumulation of Sulforaphane and Alliin in Human Prostate Tissue. Nutrients.

[38] Saito A., Ishikawa S., Yang K., Sawa A., Ishizuka K. (2025). Sulforaphane as a Potential Therapeutic Agent: A Comprehensive Analysis of Clinical Trials and Mechanistic Insights. J. Nutr. Sci..

[39] Cornblatt B.S., Ye L., Dinkova-Kostova A.T., Erb M., Fahey J.W., Singh N.K., Chen M.-S.A., Stierer T., Garrett-Mayer E., Argani P. (2007). Preclinical and Clinical Evaluation of Sulforaphane for Chemoprevention in the Breast. Carcinogenesis.

[40] Wettersten H.I., Hwang S.H., Li C., Shiu E.Y., Wecksler A.T., Hammock B.D., Weiss R.H. (2013). A Novel P21 Attenuator Which Is Structurally Related to Sorafenib. Cancer Biol. Ther..

[41] Gupta R., Dong Y., Solomon P.D., Wettersten H.I., Cheng C.J., Min J.-N., Henson J., Dogra S.K., Hwang S.H., Hammock B.D. (2014). Synergistic Tumor Suppression by Combined Inhibition of Telomerase and CDKN1A. Proc. Natl. Acad. Sci. USA.

[42] Liang R., Zhu X. (2021). UC2288 Induces Cell Apoptosis of Nasopharyngeal Carcinoma Cells via Inhibiting EGFR/ERK Pathway. J. Cancer.

[43] Wilhelm S.M., Carter C., Tang L., Wilkie D., McNabola A., Rong H., Chen C., Zhang X., Vincent P., McHugh M. (2004). BAY 43-9006 Exhibits Broad Spectrum Oral Antitumor Activity and Targets the RAF/MEK/ERK Pathway and Receptor Tyrosine Kinases Involved in Tumor Progression and Angiogenesis. Cancer Res..

[44] Rotblat B., Melino G., Knight R.A. (2012). NRF2 and P53: Januses in Cancer?. Oncotarget.

[45] Wakabayashi N., Slocum S.L., Skoko J.J., Shin S., Kensler T.W. (2010). When NRF2 Talks, Who’s Listening?. Antioxid. Redox Signal..

[46] Asher G., Tsvetkov P., Kahana C., Shaul Y. (2005). A Mechanism of Ubiquitin-Independent Proteasomal Degradation of the Tumor Suppressors P53 and P73. Genes Dev..

[47] Buzek J., Latonen L., Kurki S., Peltonen K., Laiho M. (2002). Redox State of Tumor Suppressor P53 Regulates Its Sequence-Specific DNA Binding in DNA-Damaged Cells by Cysteine 277. Nucleic Acids Res..

[48] Chen W., Sun Z., Wang X.-J., Jiang T., Huang Z., Fang D., Zhang D.D. (2009). Direct Interaction between Nrf2 and P21(Cip1/WAF1) Upregulates the Nrf2-Mediated Antioxidant Response. Mol. Cell.

[49] Pang L.Y., Scott M., Hayward R.L., Mohammed H., Whitelaw C.B.A., Smith G.C.M., Hupp T.R. (2011). P21(WAF1) Is Component of a Positive Feedback Loop That Maintains the P53 Transcriptional Program. Cell Cycle.

[50] Chin Y.E., Kitagawa M., Su W.C., You Z.H., Iwamoto Y., Fu X.Y. (1996). Cell Growth Arrest and Induction of Cyclin-Dependent Kinase Inhibitor P21 WAF1/CIP1 Mediated by STAT1. Science.

[51] Brandariz-Nuñez A., Valle-Casuso J.C., White T.E., Laguette N., Benkirane M., Brojatsch J., Diaz-Griffero F. (2012). Role of SAMHD1 Nuclear Localization in Restriction of HIV-1 and SIVmac. Retrovirology.

[52] Wang C., Meng L., Wang J., Zhang K., Duan S., Ren P., Wei Y., Fu X., Yu B., Wu J. (2021). Role of Intracellular Distribution of Feline and Bovine SAMHD1 Proteins in Lentiviral Restriction. Virol. Sin..

[53] Daddacha W., Koyen A.E., Bastien A.J., Head P.E., Dhere V.R., Nabeta G.N., Connolly E.C., Werner E., Madden M.Z., Daly M.B. (2017). SAMHD1 Promotes DNA End Resection to Facilitate DNA Repair by Homologous Recombination. Cell Rep..

[54] Coquel F., Silva M.-J., Técher H., Zadorozhny K., Sharma S., Nieminuszczy J., Mettling C., Dardillac E., Barthe A., Schmitz A.-L. (2018). SAMHD1 Acts at Stalled Replication Forks to Prevent Interferon Induction. Nature.

[55] Herold N., Rudd S.G., Sanjiv K., Kutzner J., Bladh J., Paulin C.B.J., Helleday T., Henter J.-I., Schaller T. (2017). SAMHD1 Protects Cancer Cells from Various Nucleoside-Based Antimetabolites. Cell Cycle.

[56] Arnold L.H., Groom H.C.T., Kunzelmann S., Schwefel D., Caswell S.J., Ordonez P., Mann M.C., Rueschenbaum S., Goldstone D.C., Pennell S. (2015). Phospho-Dependent Regulation of SAMHD1 Oligomerisation Couples Catalysis and Restriction. PLoS Pathog..

[57] Orris B., Huynh K.W., Ammirati M., Han S., Bolaños B., Carmody J., Petroski M.D., Bosbach B., Shields D.J., Stivers J.T. (2022). Phosphorylation of SAMHD1 Thr592 Increases C-Terminal Domain Dynamics, Tetramer Dissociation and SsDNA Binding Kinetics. Nucleic Acids Res..

