# Supplementary material for: p21 (CDKN1A) Is the Major Driver of Sulforaphane-Mediated Reduction in SAMHD1 T592 Phosphorylation in Macrophages
Source: Biomolecules. 2026 Aug 20;16(8):1213. doi: 10.3390/biom16081213 (PMC13511314; doi:10.3390/biom16081213)
Supplement: Supplementary file 1 [file biomolecules-16-01213-s001.zip › Figure S1 and S2.pdf]

biomolecules

MDPI

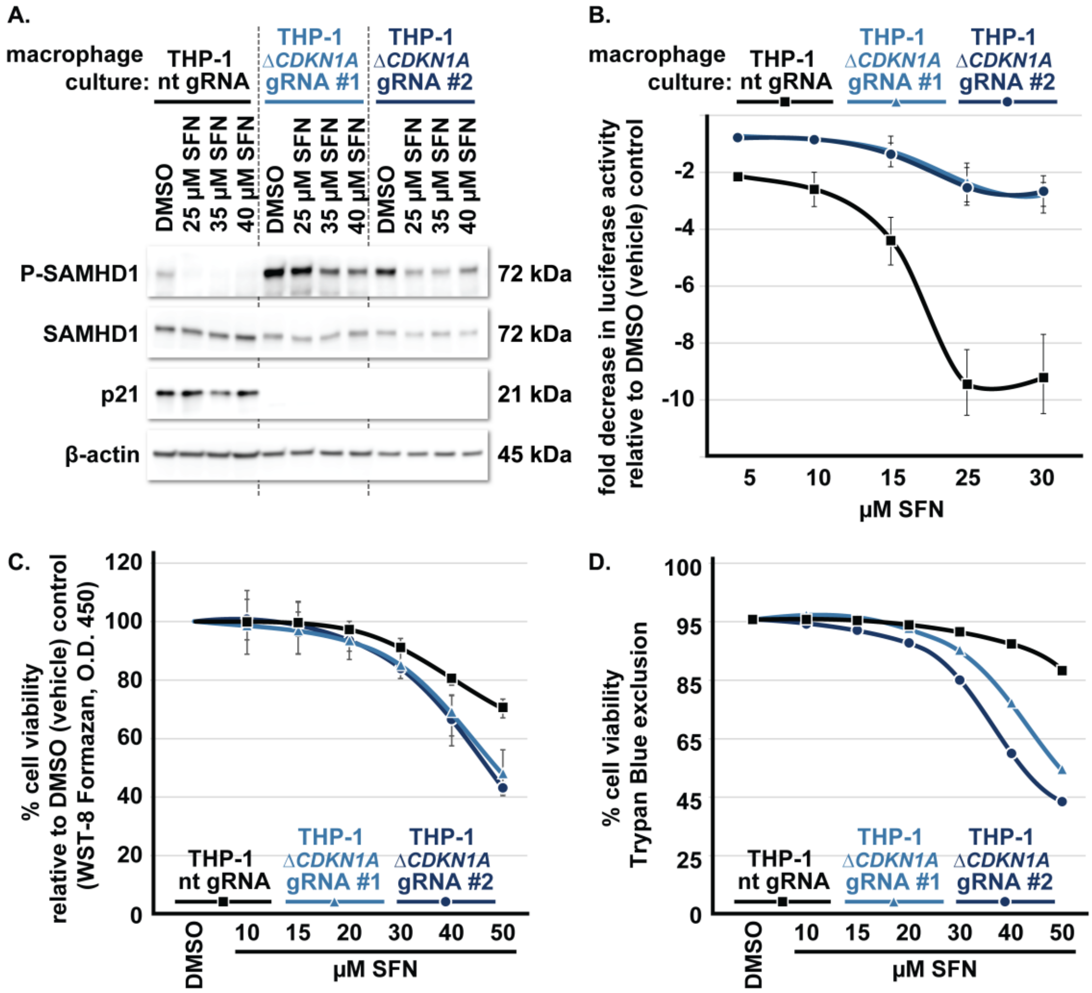

Figure S1. Effects of high-dose SFN on control and Δ*CDKN1A* THP-1 macrophages. **(A)** Control and THP-1$^{\Delta CDKN1A}$ macrophages were treated with vehicle (DMSO) or high concentrations of SFN (25–40 µM) for 24 h. After 24 h, the cells were lysed and proteins denatured. Lysate proteins were resolved via SDS-PAGE and analyzed by western blot using antibodies specific to the indicated proteins. **(B)** Control and THP-1$^{\Delta CDKN1A}$ macrophages were pre-treated with vehicle (DMSO) or increasing concentrations of SFN (5–30 µM) for 24 h, washed to remove residual DMSO/SFN, and transduced with a VSV-G-pseudotyped HIV-1 luciferase reporter virus. Luciferase activity was quantified from cell lysates 72 h post-transduction. The fold decrease in luciferase activity relative to the vehicle (DMSO) control was determined. Data represents the mean ± SD of three independent experiments (n = 3). **(C) and (D)** Control and THP-1$^{\Delta CDKN1A}$ macrophages were treated with vehicle (DMSO) or increasing concentrations of SFN (10–50 µM) for 36 h. After 36 h cell viability was evaluated as follows: (c) Relative metabolic activity was quantified via water-soluble tetrazolium salt reduction (WST-8 assay). Absorbance values (450 nm) were normalized against vehicle controls (DMSO) to define 100% viability. Data represents the mean ± SD of triplicates (n = 3). (d) Percentage of viable macrophages calculated via in situ Trypan Blue exclusion staining following 10% formalin fixation. Cellular quantification was performed via computer-assisted vision analysis (Google Gemini 1.5 Pro) and cross-verified by manual human blinding (n = 1).

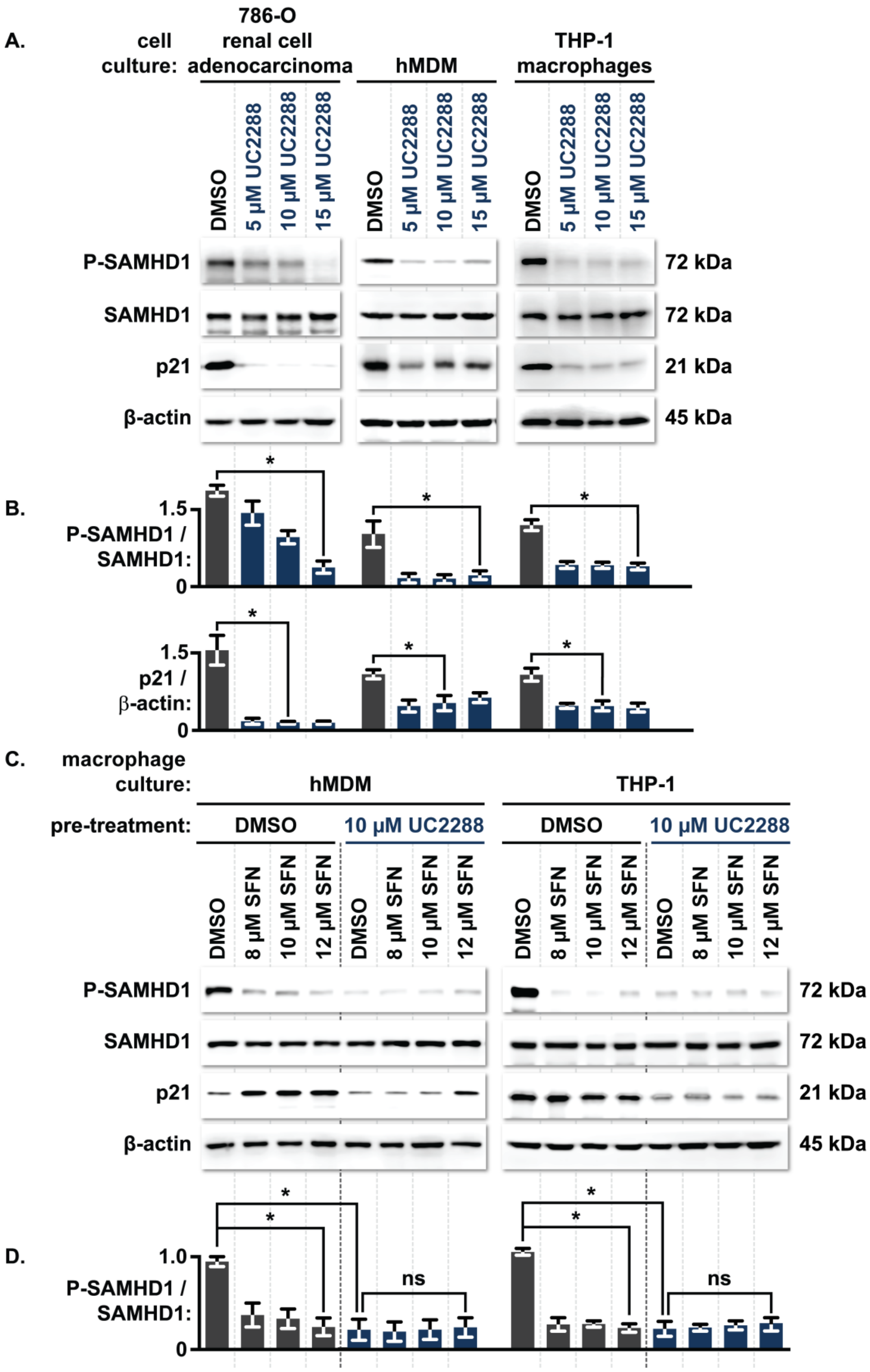

Figure S2. UC2288 reduces SAMHD1 phosphorylation and occludes additive effects of SFN in macrophages. (**A**) 786-O renal adenocarcinoma cells, primary hMDMs, and PMA-differentiated THP-1 macrophages were treated with vehicle (DMSO) or increasing concentrations of UC2288 (5–15 µM) for 24 h. After 24 h, the cells were lysed and proteins denatured. Lysate proteins were resolved via

https://doi.org/10.3390/xxxxx

SDS-PAGE and analyzed by Western blot using antibodies specific to the indicated proteins. (**B**) Densitometric analysis representing the average relative ratio of T592 phosphorylated SAMHD1 (P-SAMHD1) to total SAMHD1, and p21 to the β-actin loading control for the data presented in panel (**A**). (**C**) primary hMDMs and PMA-differentiated THP-1 macrophages were pre-treated with vehicle (DMSO) or 10 μM UC2288 for 12 h. After 12 h, the cells were treated with vehicle (DMSO) or increasing concentrations of SFN (8–12 μM) for an additional 12 h. Twelve hours after SFN treatment (24 h post-UC2288 treatment), the cells were harvested and lysate proteins were analyzed via Western blot using antibodies specific to the indicated proteins. (**D**) Densitometric analysis data representing the average relative ratio of P-SAMHD1 to total SAMHD1 for the data presented in panel (**C**). Statistical significance across treatment groups was determined using a Repeated Measures One-Way ANOVA (for primary hMDMs from two male and one female donor) or an ordinary One-Way ANOVA (for THP-1 macrophages and 786-O cells), followed by Dunnett's post hoc test to evaluate pairwise differences against the vehicle control for data presented in (**B**). Statistical significance for combinatorial treatments in panel (**D**) was evaluated using a Repeated Measures (hMDM) or an ordinary (THP-1) Two-Way ANOVA followed by Tukey's post hoc multiple comparisons test to compare all treatment condition means against one another. "*" = $p < 0.05$, "ns" = not significant. All original Western blot images can be found in Supplementary File S1.
