# Supplementary material for: p21 (CDKN1A) Is the Major Driver of Sulforaphane-Mediated Reduction in SAMHD1 T592 Phosphorylation in Macrophages
Source: Biomolecules. 2026 Aug 20;16(8):1213. doi: 10.3390/biom16081213 (PMC13511314; doi:10.3390/biom16081213)
Supplement: Supplementary file 1 [file biomolecules-16-01213-s001.zip › Supplementary File S1 Original Western Blot Images/Original Western Blot Images.pdf]

Figure 1A. hMDM. P-SAMHD1 from Donors 1&2

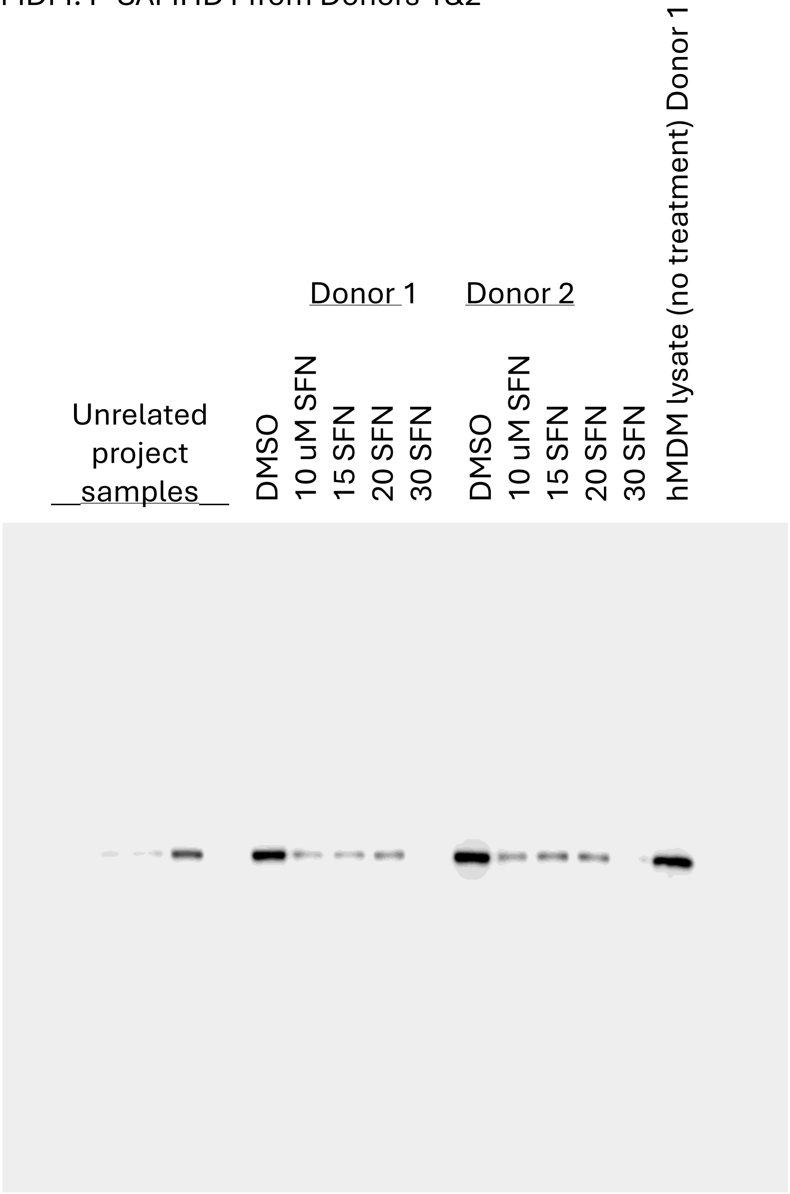

For all representative western blots, equal loading was confirmed using corresponding housekeeping controls (e.g., beta-actin), with all target proteins for a given series of blots resolved from the exact same sample lysates.

Figure 1A. hMDM. P-SAMHD1 from Donor 3

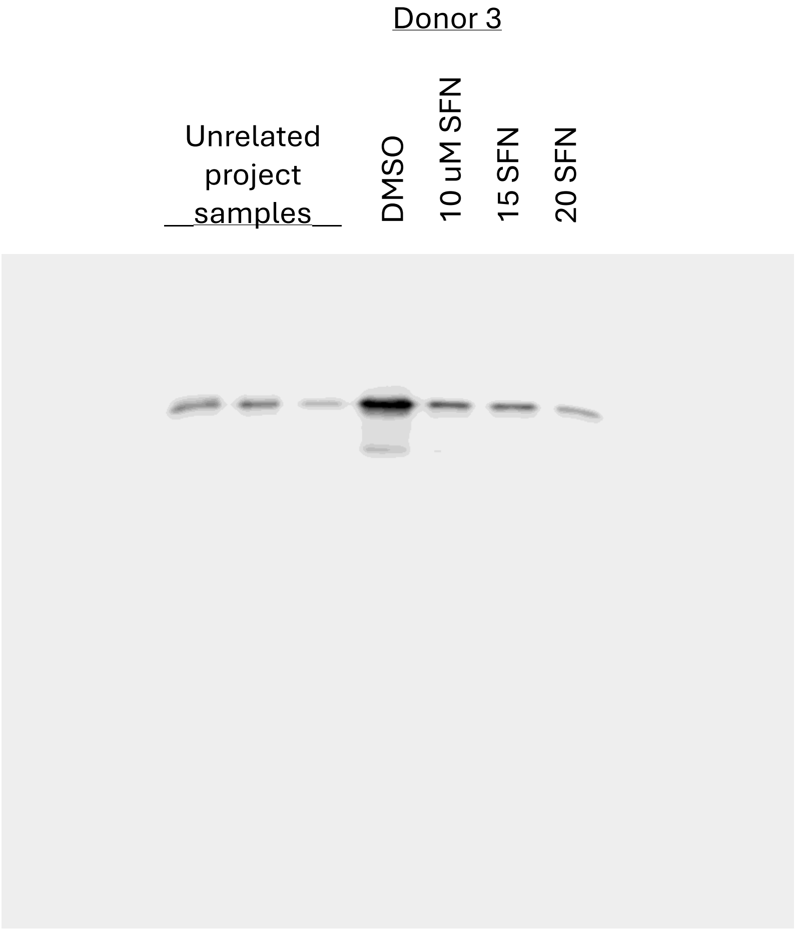

For all representative western blots, equal loading was confirmed using corresponding housekeeping controls (e.g., beta-actin), with all target proteins for a given series of blots resolved from the exact same sample lysates.

Figure 1A. hMDM.
Total SAMHD1

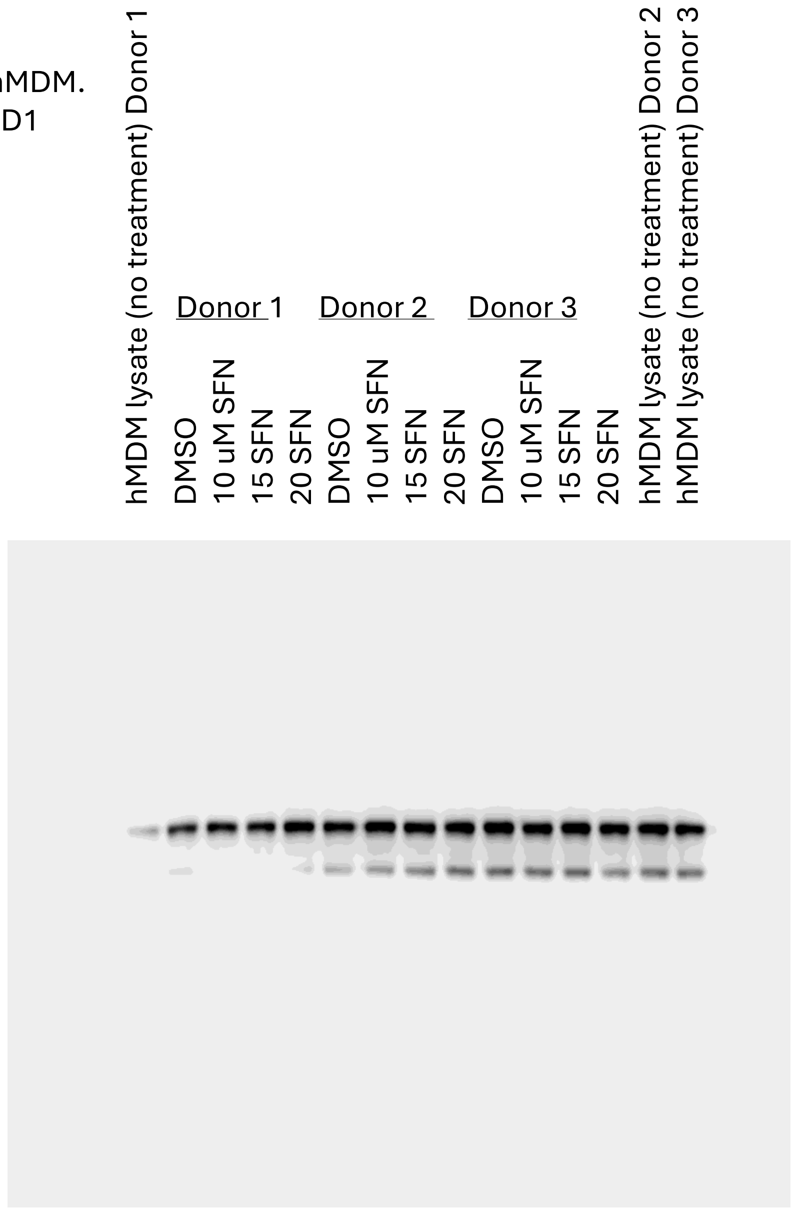

For all representative western blots, equal loading was confirmed using corresponding housekeeping controls (e.g., beta-actin), with all target proteins for a given series of blots resolved from the exact same sample lysates.

Figure 1A. hMDM. P21. Donors 1 and 2.

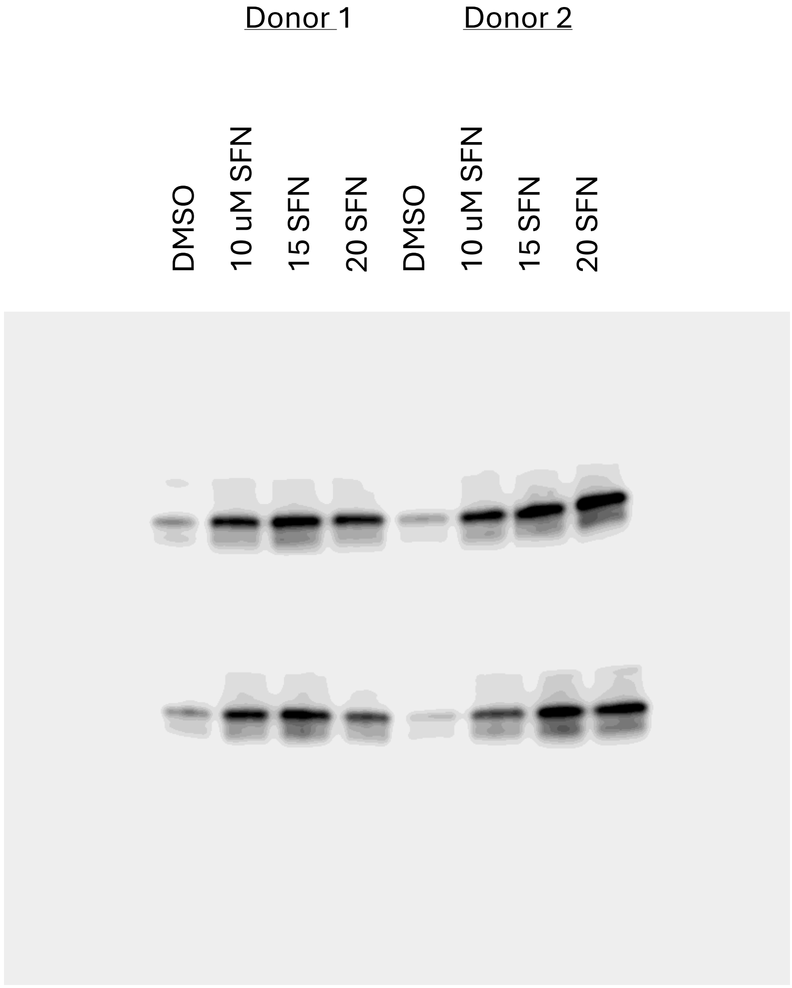

^Same samples (ran in parallel)^

For all representative western blots, equal loading was confirmed using corresponding housekeeping controls (e.g., beta-actin), with all target proteins for a given series of blots resolved from the exact same sample lysates.

Figure 1A. hMDM. P21. Donor 3.

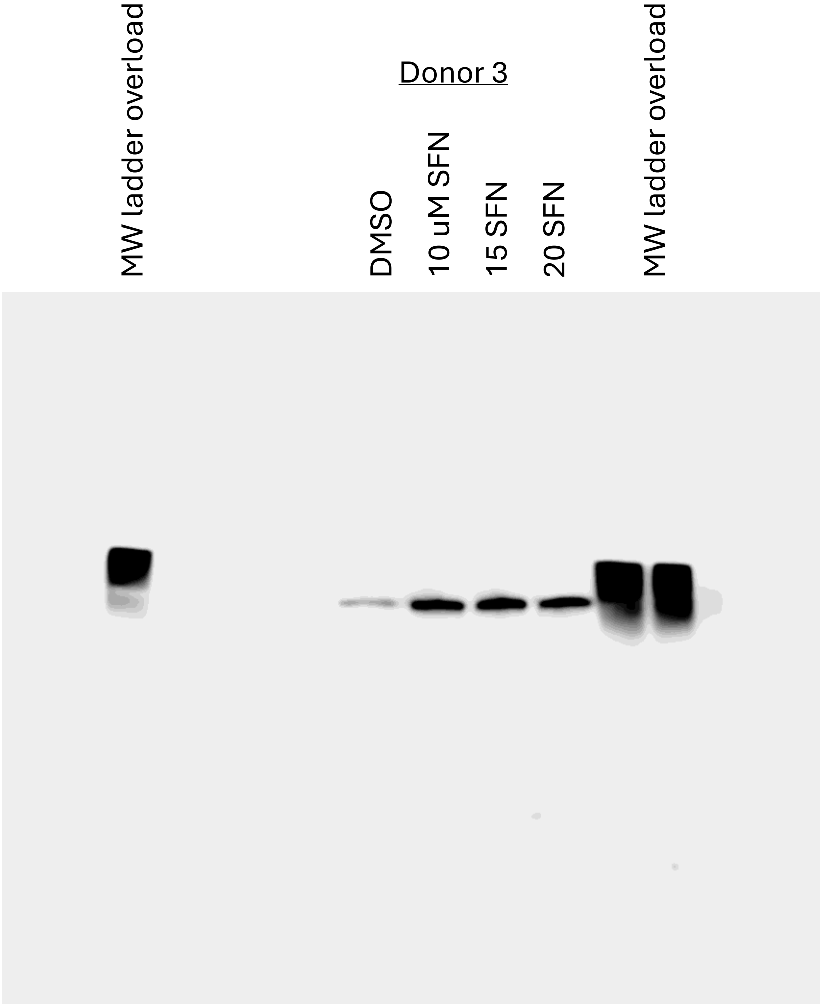

For all representative western blots, equal loading was confirmed using corresponding housekeeping controls (e.g., beta-actin), with all target proteins for a given series of blots resolved from the exact same sample lysates.

Figure 1A. hMDM. Nrf2.

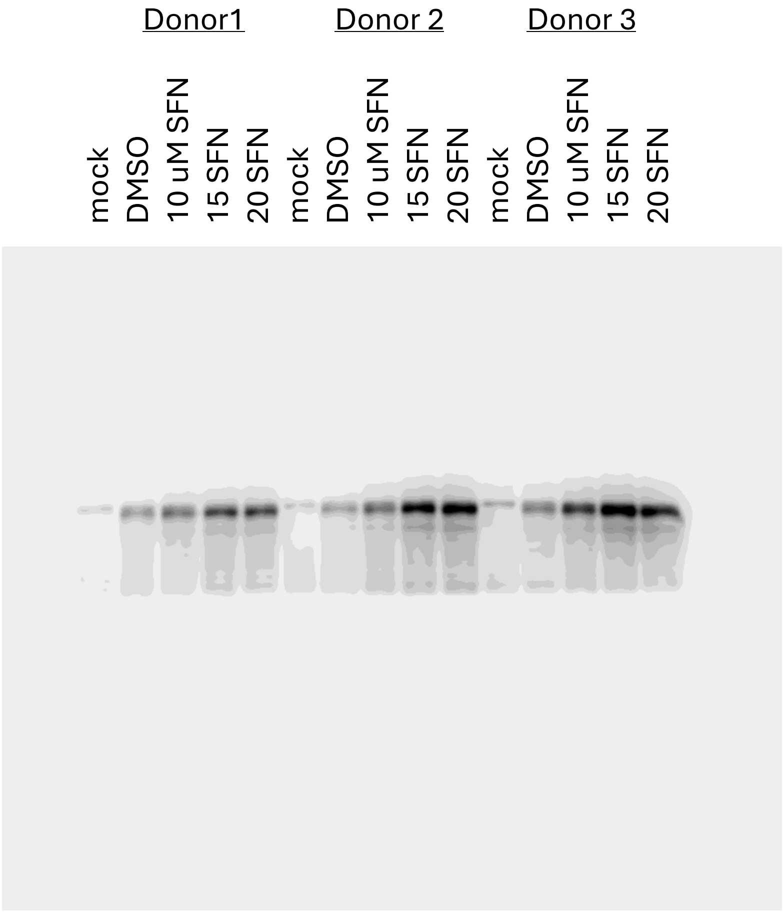

For all representative western blots, equal loading was confirmed using corresponding housekeeping controls (e.g., beta-actin), with all target proteins for a given series of blots resolved from the exact same sample lysates.

Figure 1A.
hMDM. B-actin.

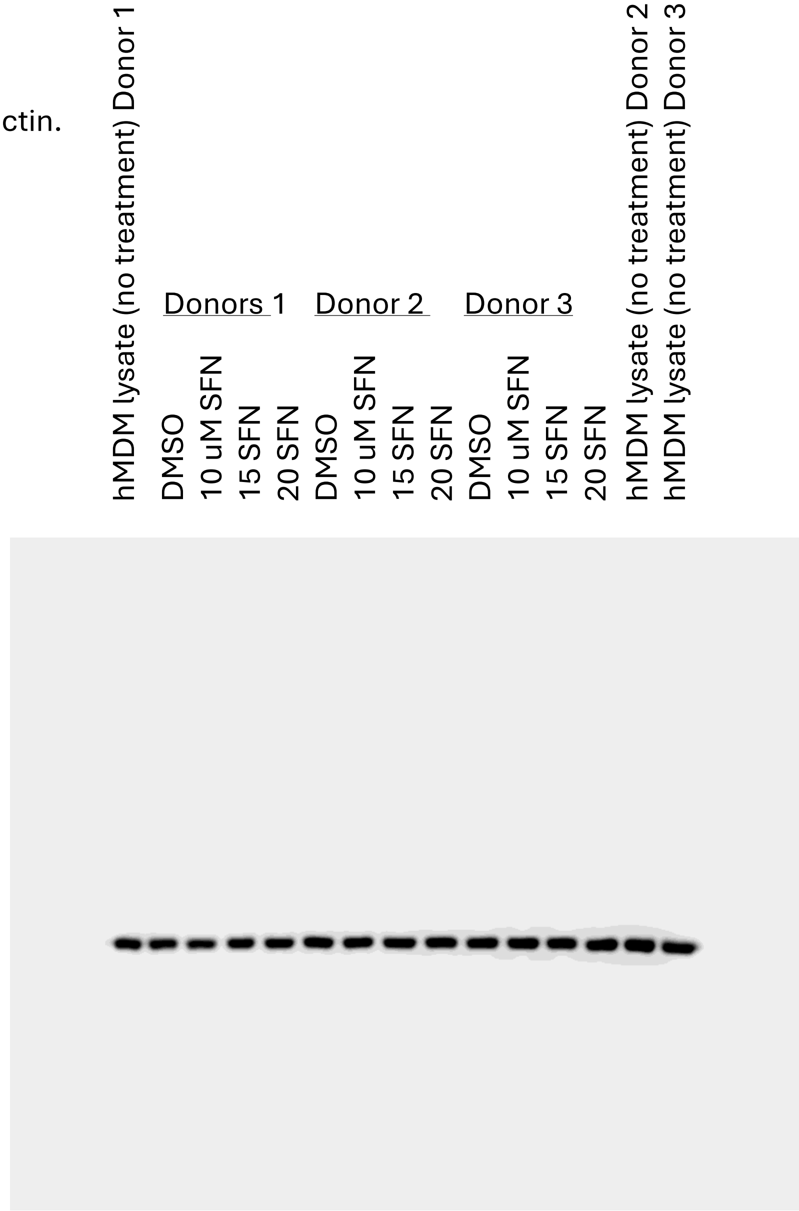

For all representative western blots, equal loading was confirmed using corresponding housekeeping controls (e.g., beta-actin), with all target proteins for a given series of blots resolved from the exact same sample lysates.

Figure 1A. THP-1. P-SAMHD1.

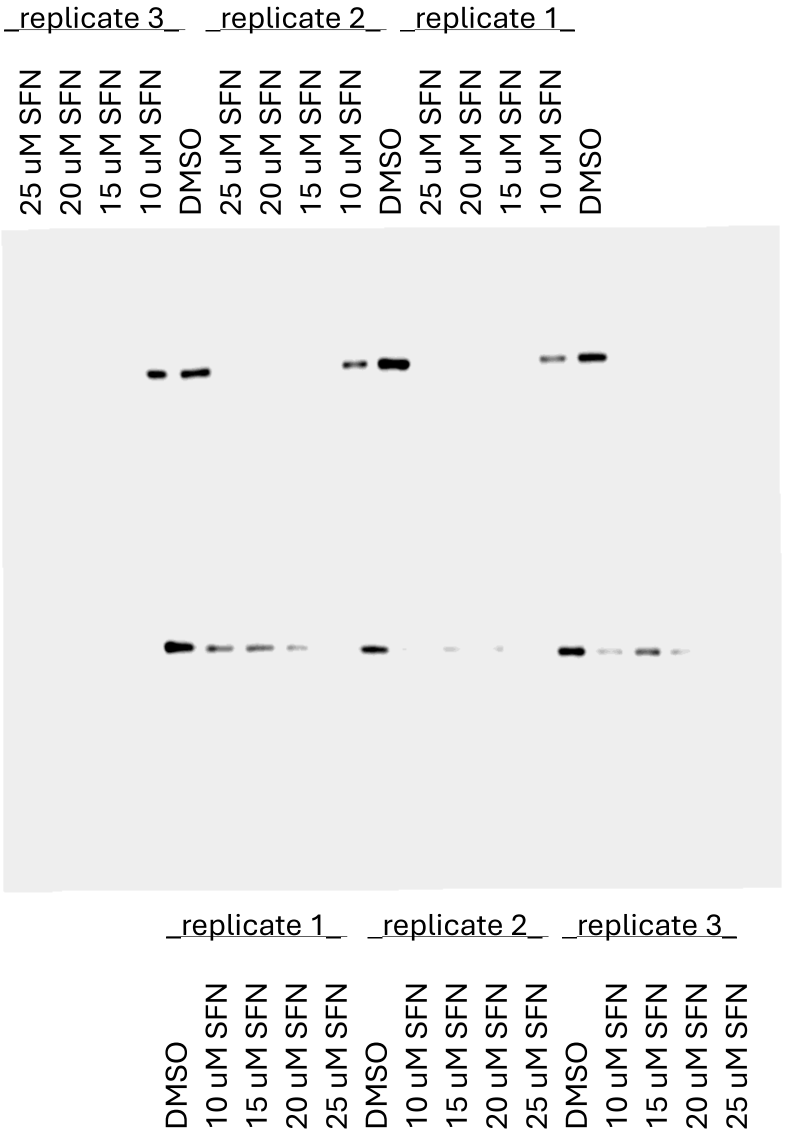

^Same samples (ran in parallel, opposite orientation)^

For all representative western blots, equal loading was confirmed using corresponding housekeeping controls (e.g., beta-actin), with all target proteins for a given series of blots resolved from the exact same sample lysates.

Figure 1A. THP-1. total SAMHD1.

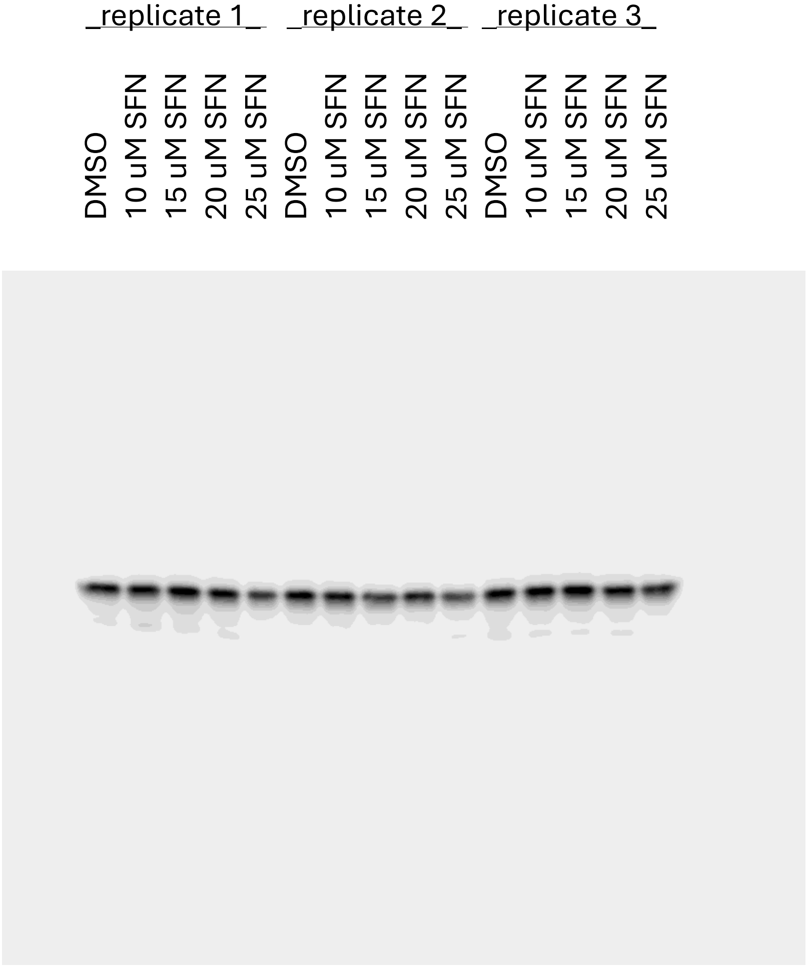

For all representative western blots, equal loading was confirmed using corresponding housekeeping controls (e.g., beta-actin), with all target proteins for a given series of blots resolved from the exact same sample lysates.

Figure 1A. THP-1. p21.

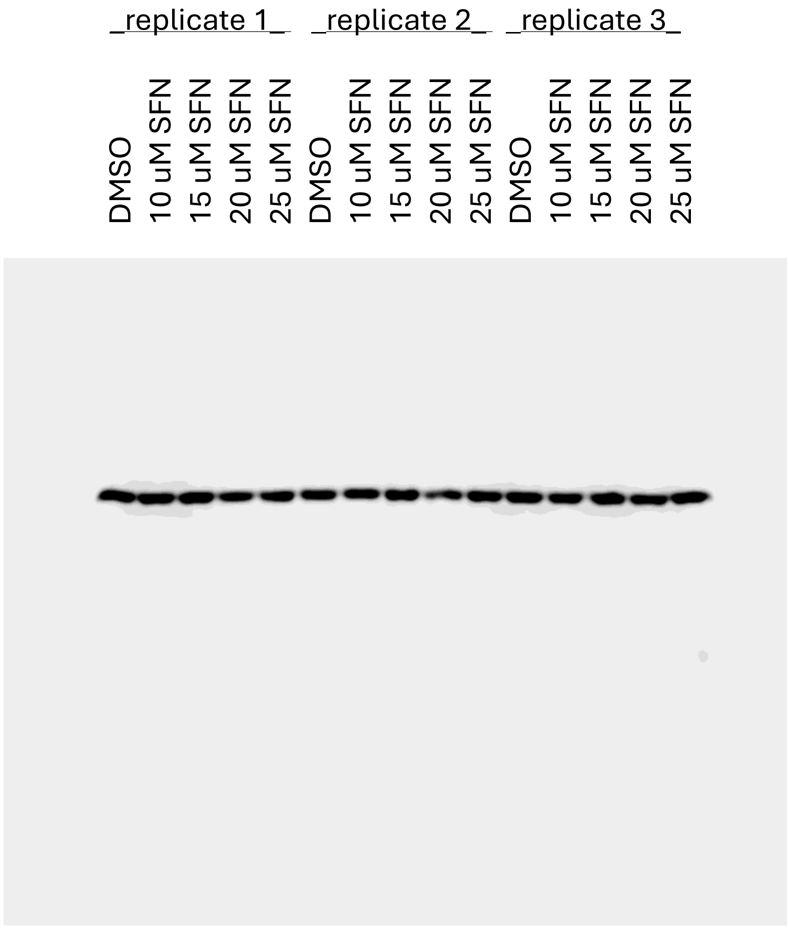

For all representative western blots, equal loading was confirmed using corresponding housekeeping controls (e.g., beta-actin), with all target proteins for a given series of blots resolved from the exact same sample lysates.

Figure 1A. THP-1. NRF2.

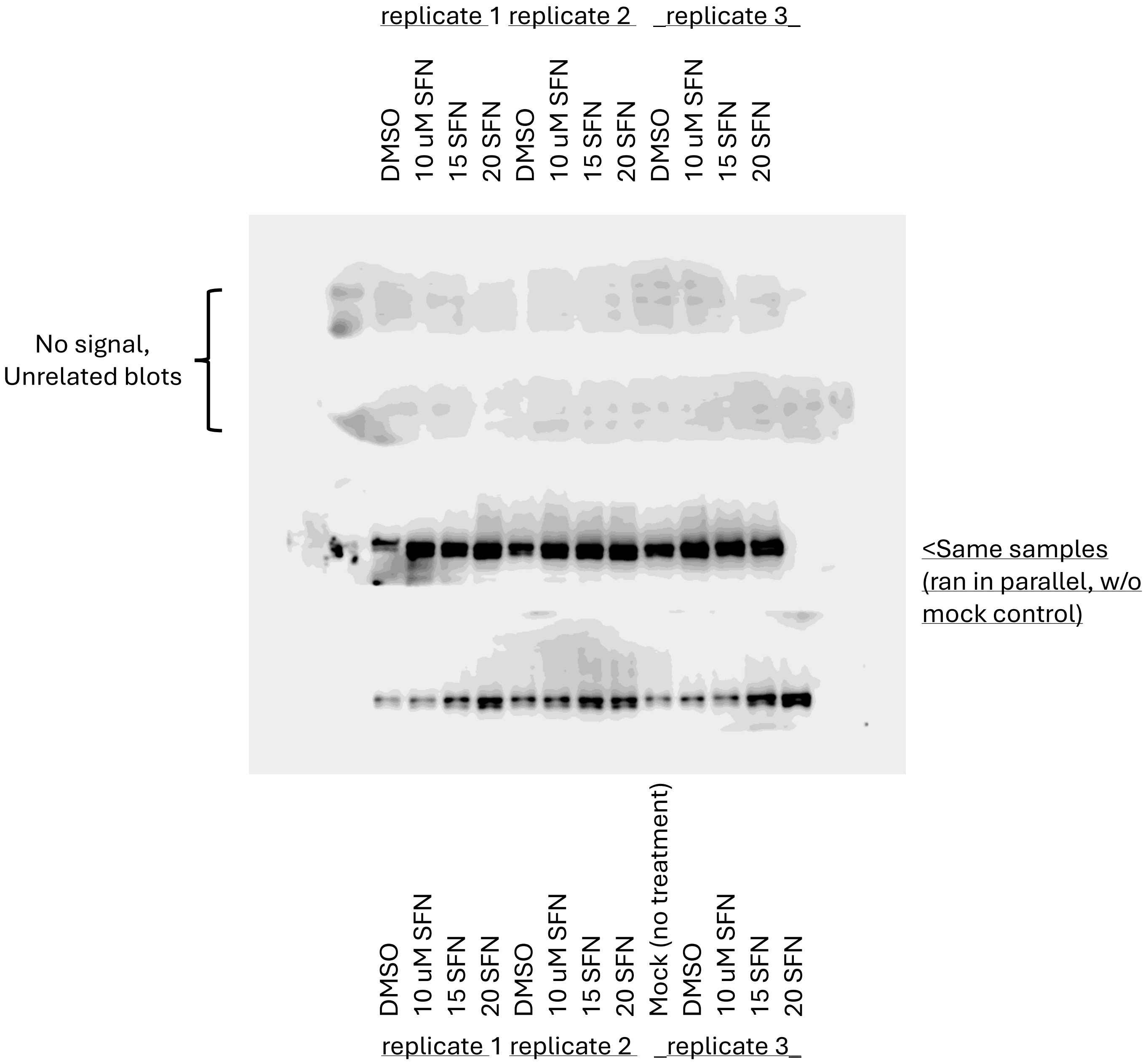

For all representative western blots, equal loading was confirmed using corresponding housekeeping controls (e.g., beta-actin), with all target proteins for a given series of blots resolved from the exact same sample lysates.

Figure 1A. THP-1. B-actin.

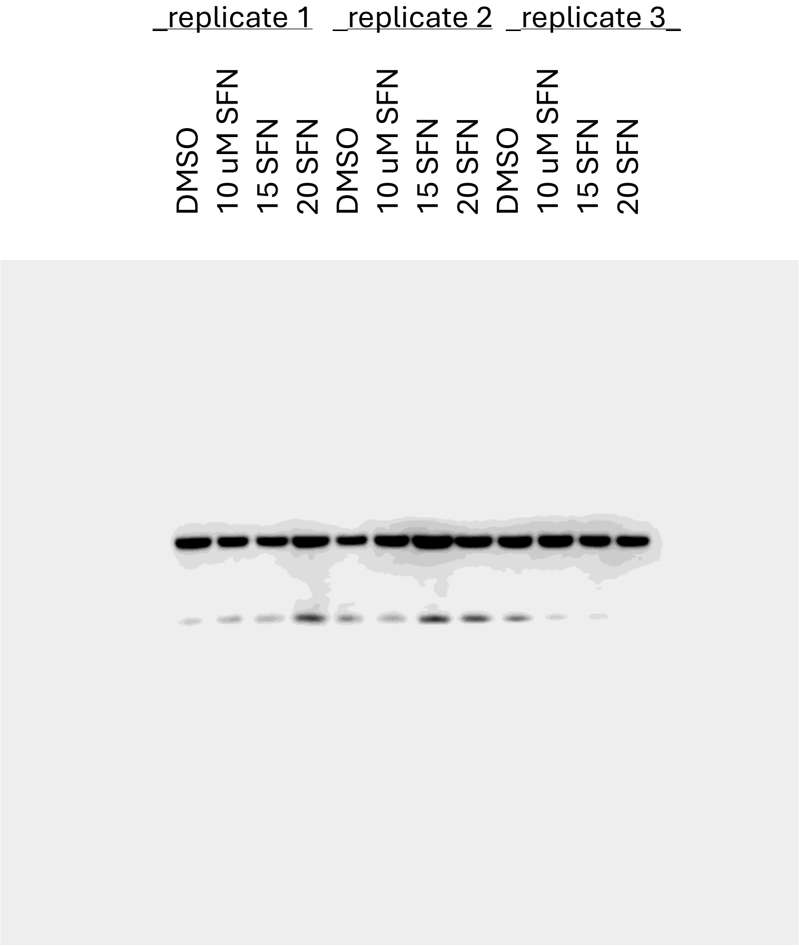

For all representative western blots, equal loading was confirmed using corresponding housekeeping controls (e.g., beta-actin), with all target proteins for a given series of blots resolved from the exact same sample lysates.

Figure 1B. hMDM and THP1. P-SAMHD1.

THP-1 Donor 3 Donor 2 Donor 1

100 U IFNy
10 uM SFN
DMSO
100 U IFNy
10 uM SFN
DMSO
100 U IFNy
10 uM SFN
DMSO
100 U IFNy
10 uM SFN
DMSO

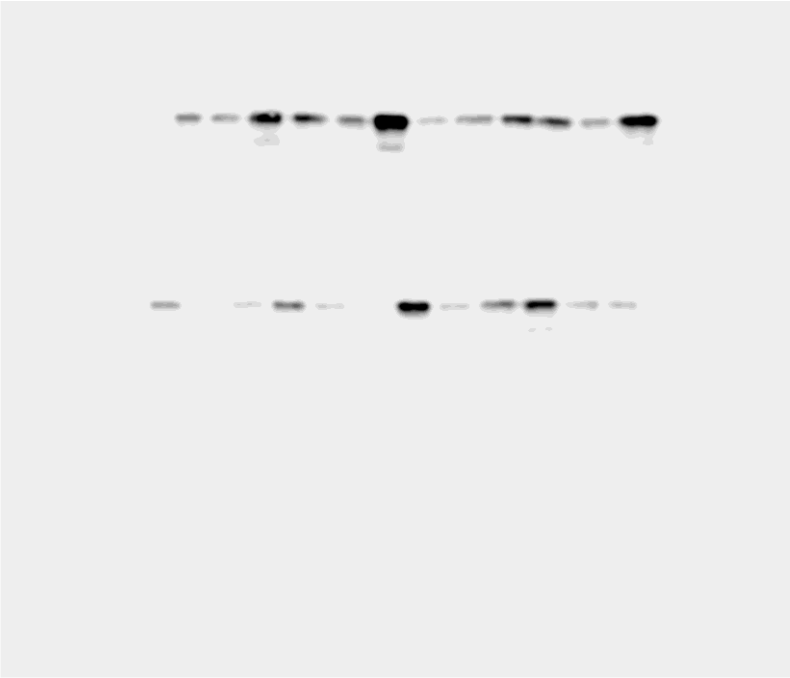

Donor 1 Donor 2 Donor 3 THP-1

DMSO
10 uM SFN
100 U IFNy
DMSO
10 uM SFN
100 U IFNy
DMSO
10 uM SFN
100 U IFNy
DMSO
10 uM SFN
100 U IFNy

^Same samples (ran in parallel, opposite orientation)^

For all representative western blots, equal loading was confirmed using corresponding housekeeping controls (e.g., beta-actin), with all target proteins for a given series of blots resolved from the exact same sample lysates.

Figure 1B. hMDM. Total SAMHD1 from Donors 1&2.

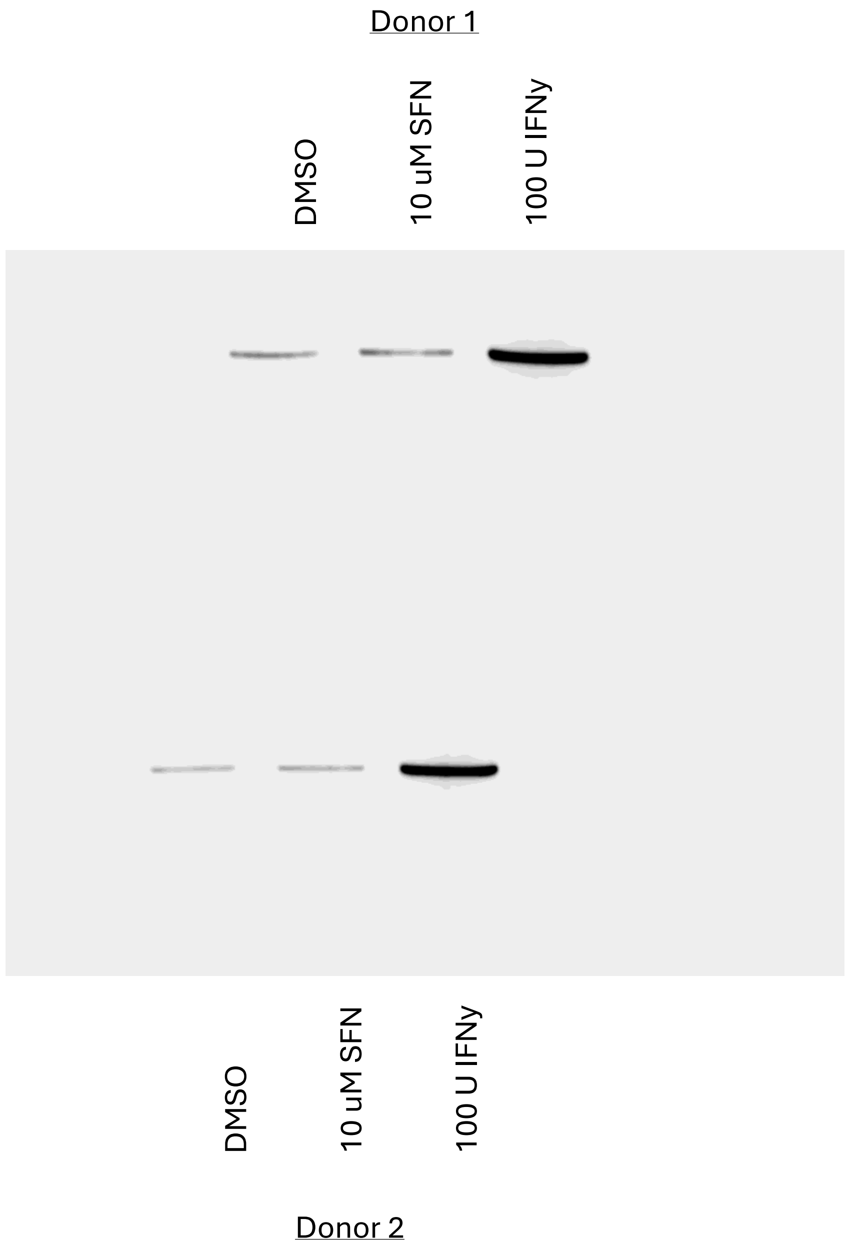

For all representative western blots, equal loading was confirmed using corresponding housekeeping controls (e.g., beta-actin), with all target proteins for a given series of blots resolved from the exact same sample lysates.

Figure 1B. hMDM. Total SAMHD1 from Donor 3.

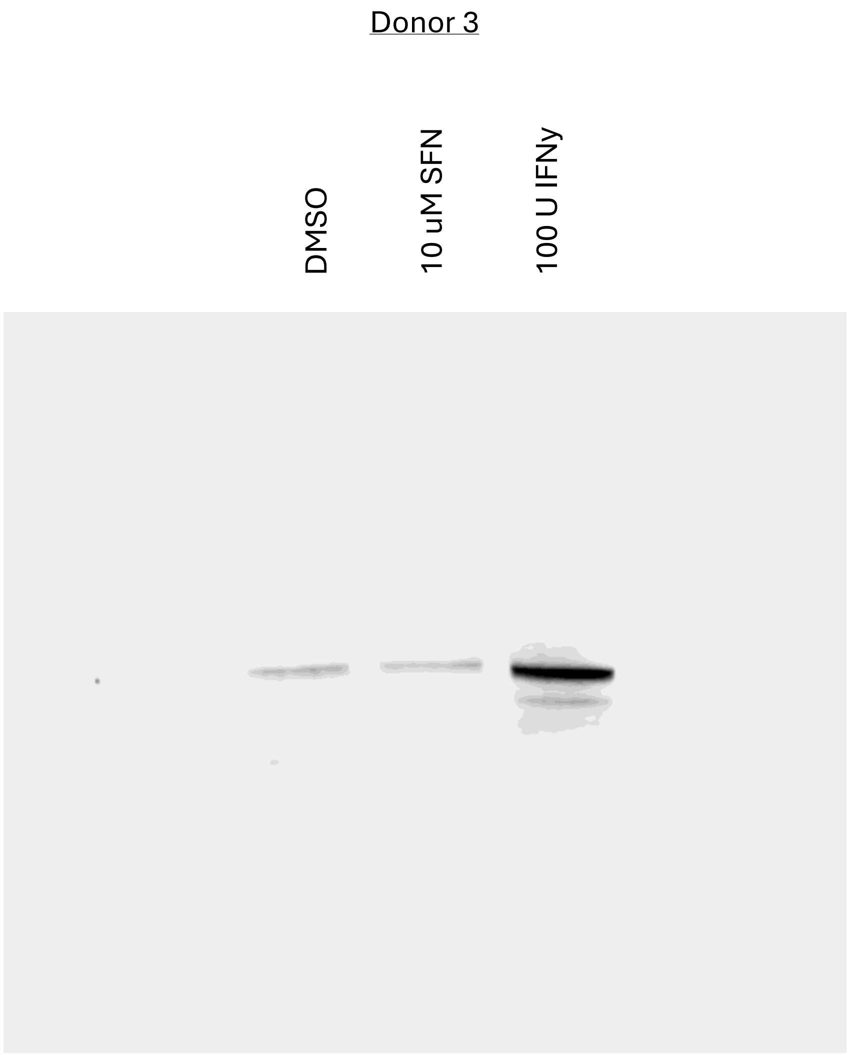

For all representative western blots, equal loading was confirmed using corresponding housekeeping controls (e.g., beta-actin), with all target proteins for a given series of blots resolved from the exact same sample lysates.

Figure 1B. hMDM.
p21.

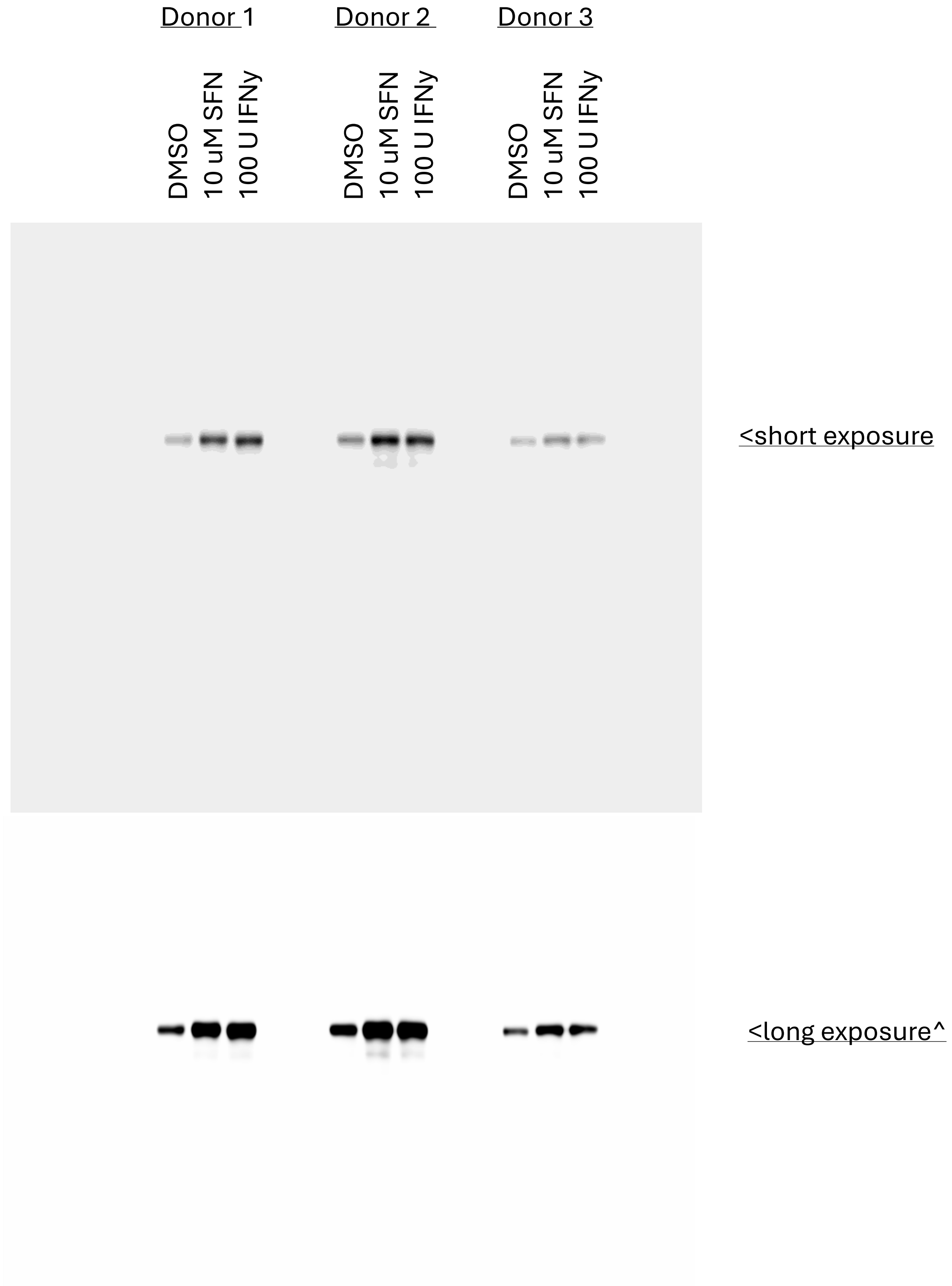

For all representative western blots, equal loading was confirmed using corresponding housekeeping controls (e.g., beta-actin), with all target proteins for a given series of blots resolved from the exact same sample lysates.

Figure 1B. hMDM.
B-actin

Donor1 Donor2 Donor3

hMDM lysate (no treatment) Donor 1
DMSO
10 uM SFN
100 U IFNy
DMSO
10 uM SFN
100 U IFNy
DMSO
10 uM SFN
100 U IFNy
hMDM lysate (no treatment) Donor 2
hMDM lysate (no treatment) Donor 3

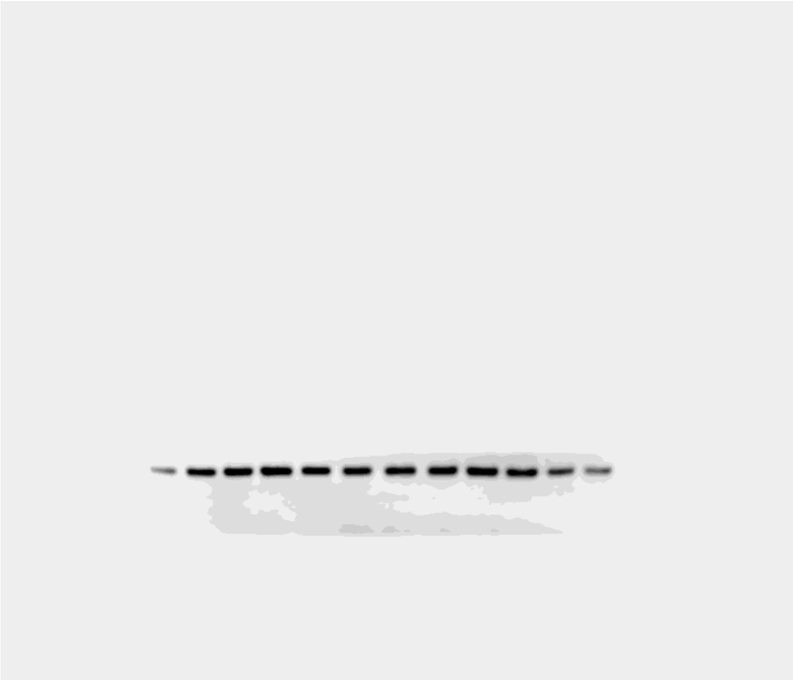

For all representative western blots, equal loading was confirmed using corresponding housekeeping controls (e.g., beta-actin), with all target proteins for a given series of blots resolved from the exact same sample lysates.

Figure 1B. THP-1. total SAMHD1.

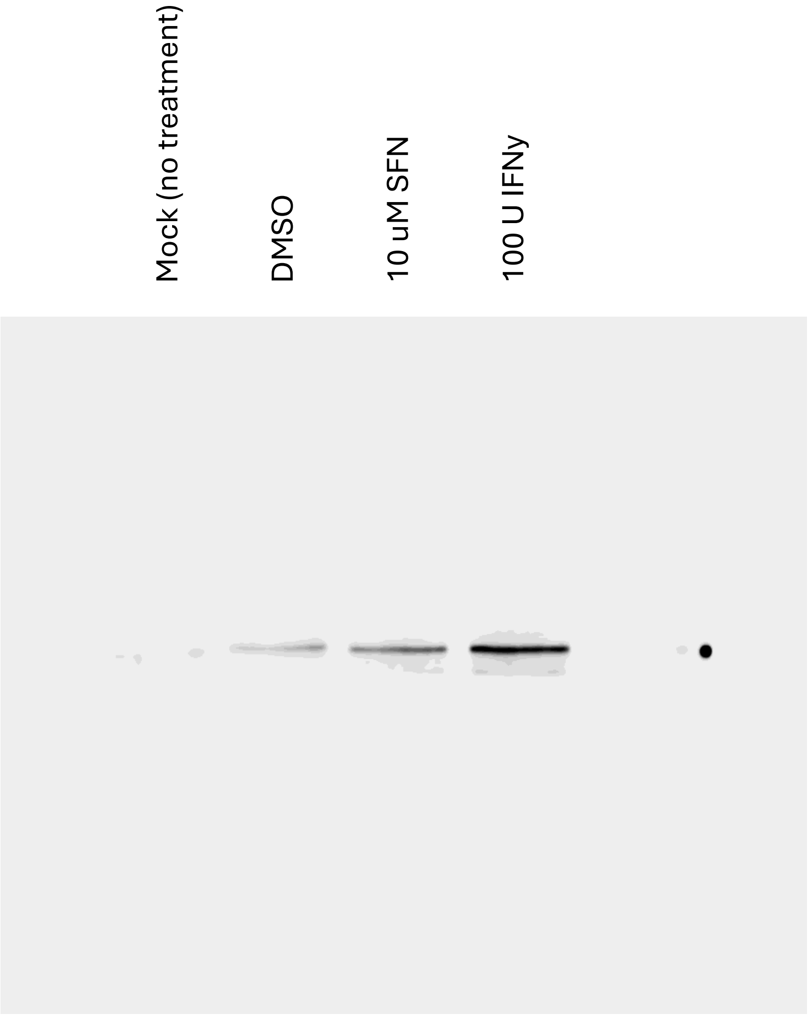

For all representative western blots, equal loading was confirmed using corresponding housekeeping controls (e.g., beta-actin), with all target proteins for a given series of blots resolved from the exact same sample lysates.

Figure 1B. THP-1. B-actin

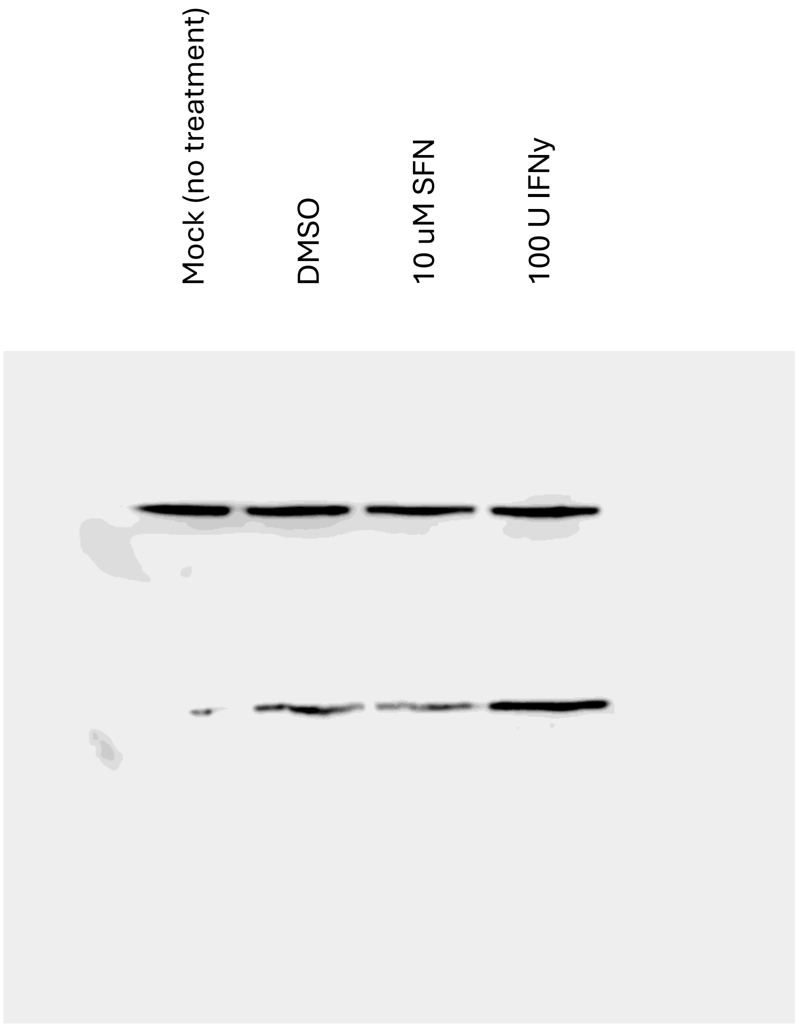

< Bottom membrane cut too close. Image not used.

For all representative western blots, equal loading was confirmed using corresponding housekeeping controls (e.g., beta-actin), with all target proteins for a given series of blots resolved from the exact same sample lysates.

Figure 1B. THP-1. p21.

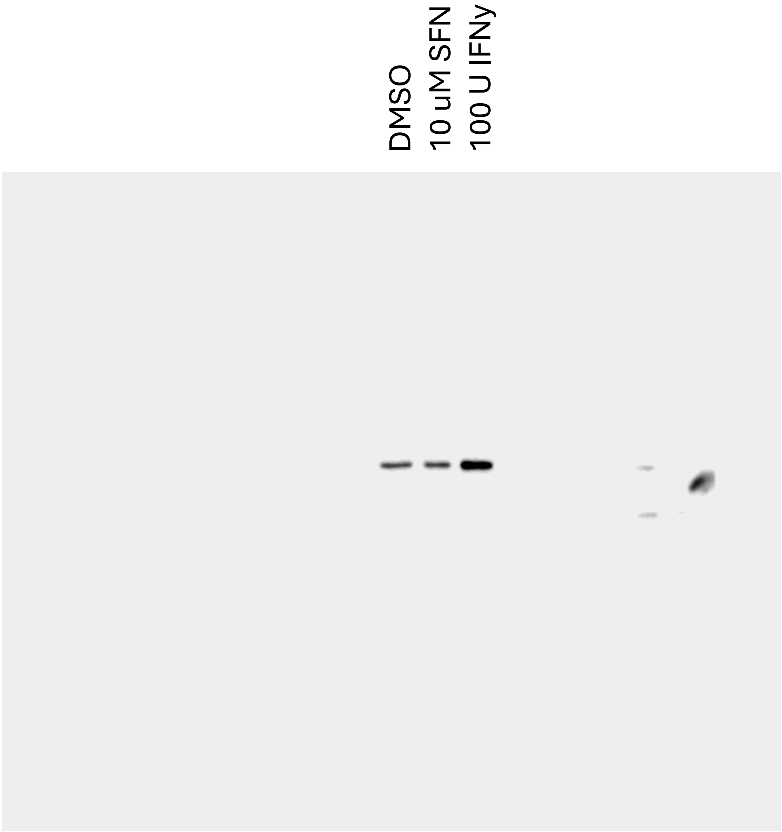

For all representative western blots, equal loading was confirmed using corresponding housekeeping controls (e.g., beta-actin), with all target proteins for a given series of blots resolved from the exact same sample lysates.

Figure 2A. hMDM. P-SAMHD1.

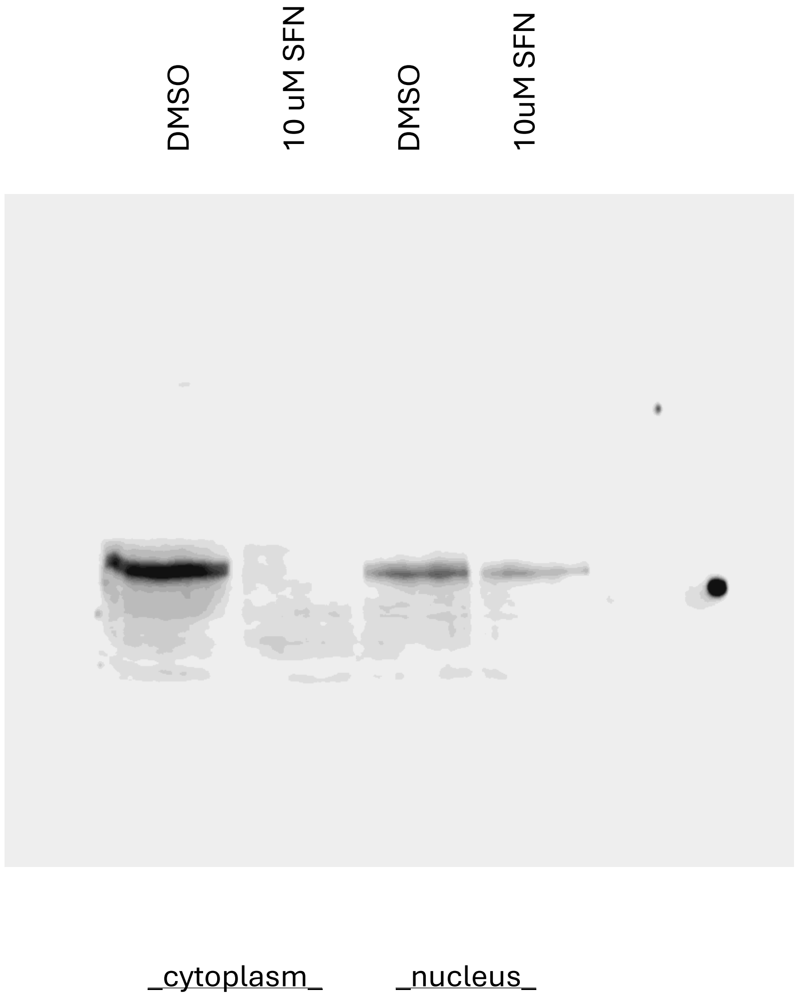

For all representative western blots, equal loading was confirmed using corresponding housekeeping controls (e.g., beta-actin), with all target proteins for a given series of blots resolved from the exact same sample lysates.

Figure 2A. hMDM. total -SAMHD1.

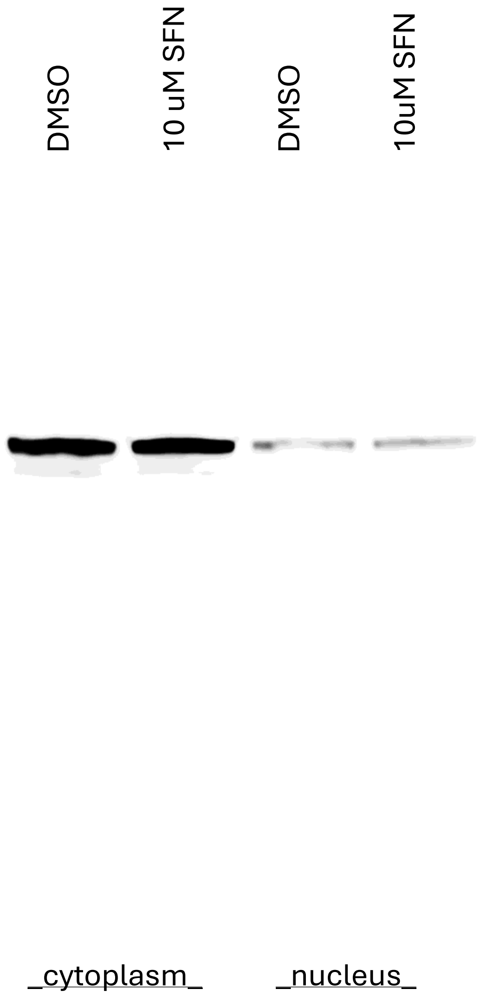

For all representative western blots, equal loading was confirmed using corresponding housekeeping controls (e.g., beta-actin), with all target proteins for a given series of blots resolved from the exact same sample lysates.

Figure 2A. hMDM. p21.

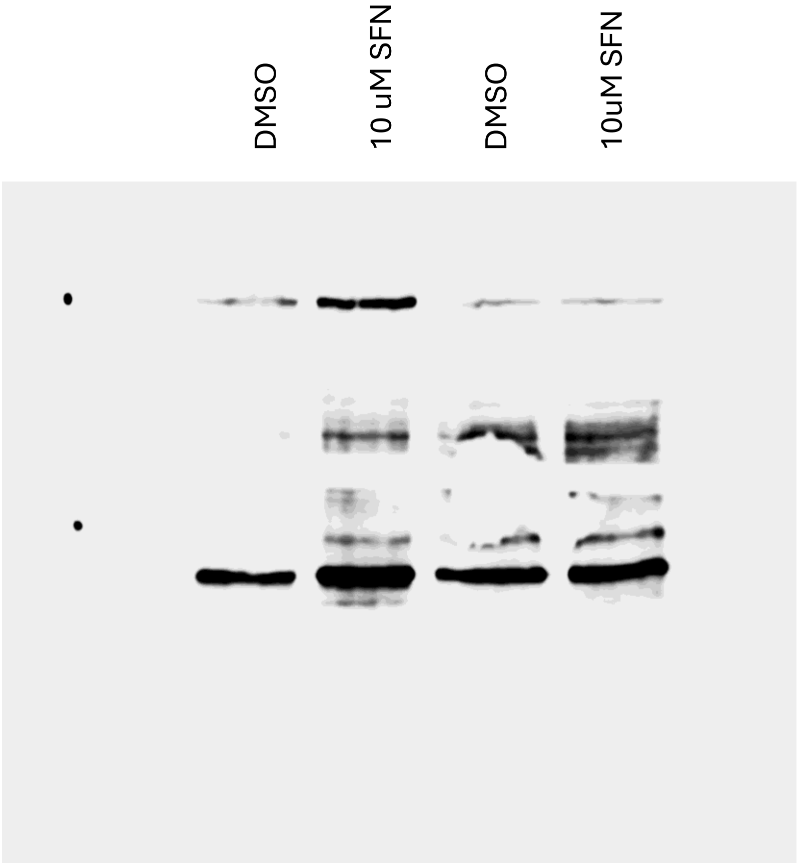

For all representative western blots, equal loading was confirmed using corresponding housekeeping controls (e.g., beta-actin), with all target proteins for a given series of blots resolved from the exact same sample lysates.

Figure 2A. hMDM. GAPDH.

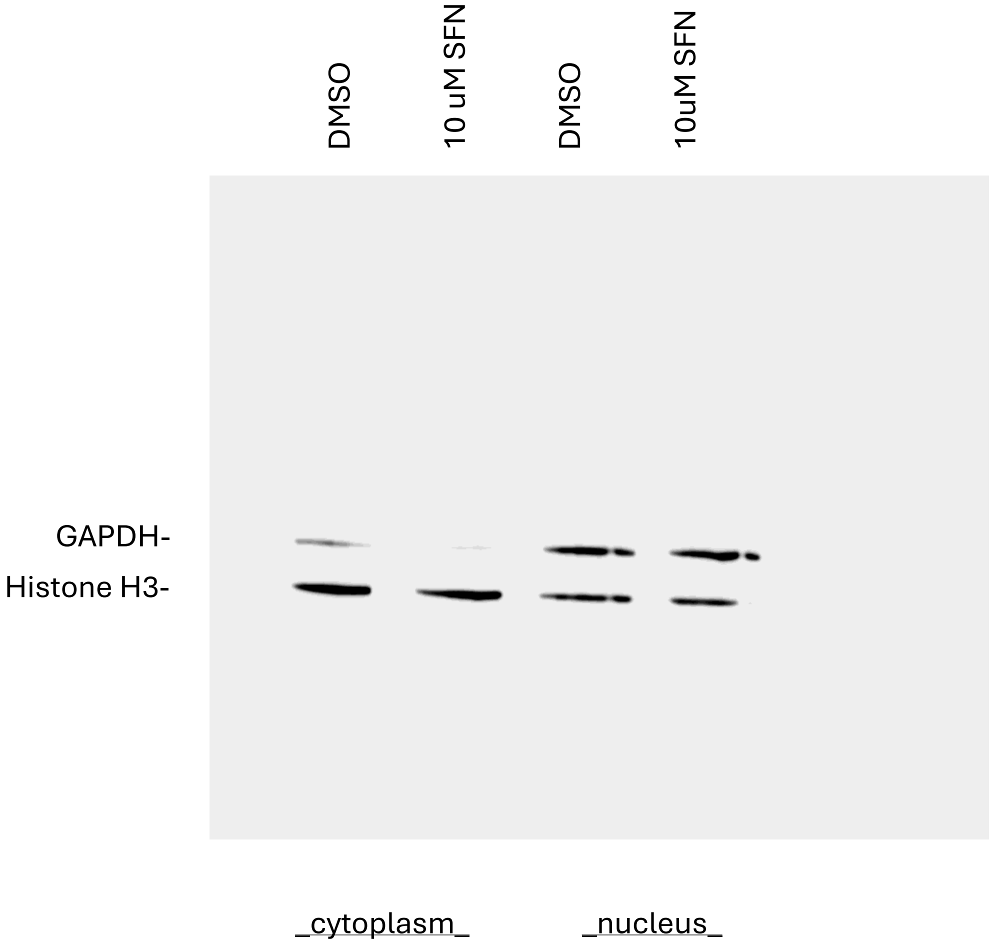

For all representative western blots, equal loading was confirmed using corresponding housekeeping controls (e.g., beta-actin), with all target proteins for a given series of blots resolved from the exact same sample lysates.

Figure 2A. hMDM. Histone H3.

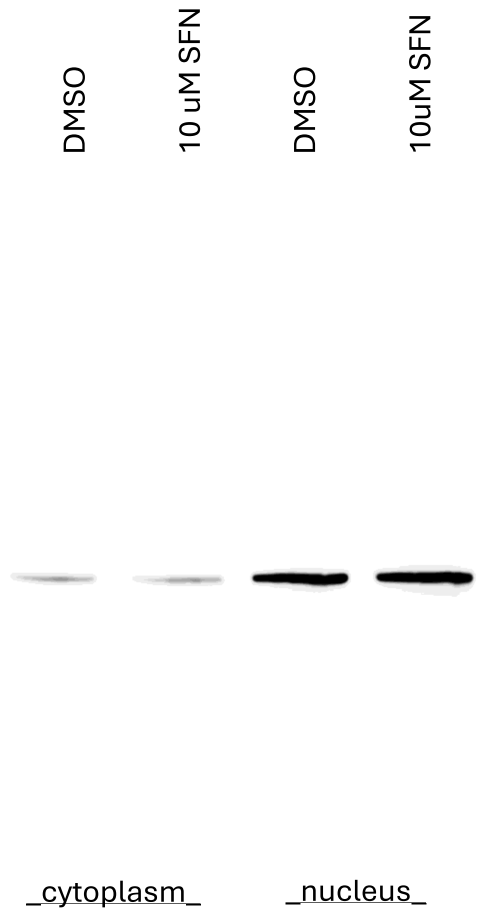

For all representative western blots, equal loading was confirmed using corresponding housekeeping controls (e.g., beta-actin), with all target proteins for a given series of blots resolved from the exact same sample lysates.

Figure 2A. THP1. P-SAMHD1.

Top membrane cut too close (cut off). Image not used.

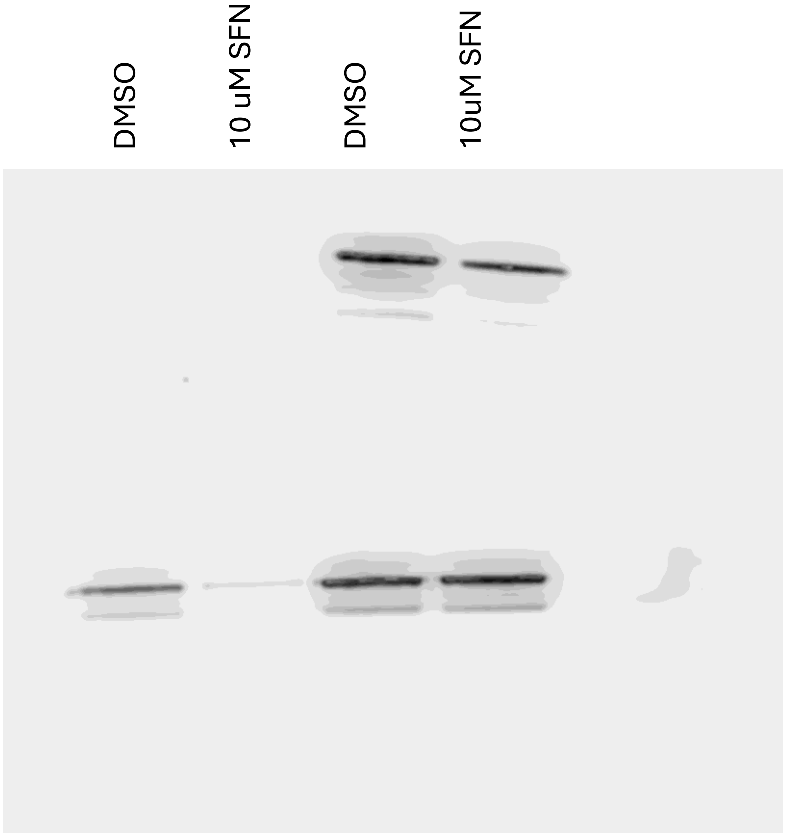

P-SAMHD1-

cytoplasm _nucleus_

For all representative western blots, equal loading was confirmed using corresponding housekeeping controls (e.g., beta-actin), with all target proteins for a given series of blots resolved from the exact same sample lysates.

Figure 2A. THP1. total-SAMHD1.

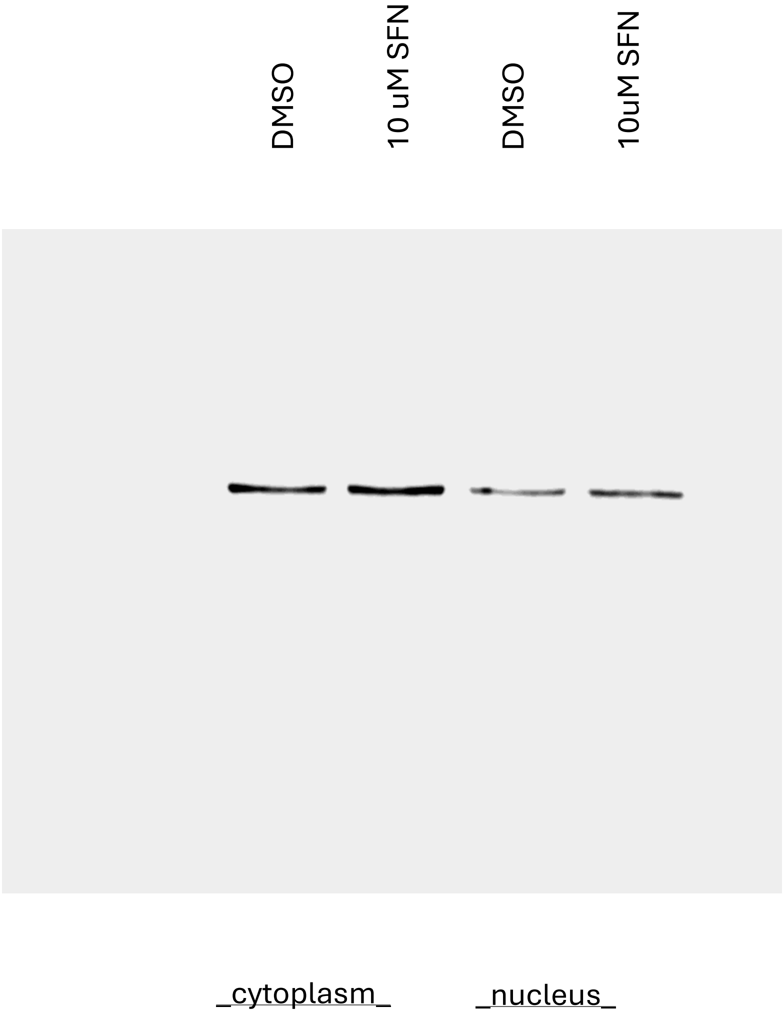

For all representative western blots, equal loading was confirmed using corresponding housekeeping controls (e.g., beta-actin), with all target proteins for a given series of blots resolved from the exact same sample lysates.

Figure 2A. THP1. p21.

cytoplasm _nucleus_

DMSO 10 uM SFN DMSO 10uM SFN

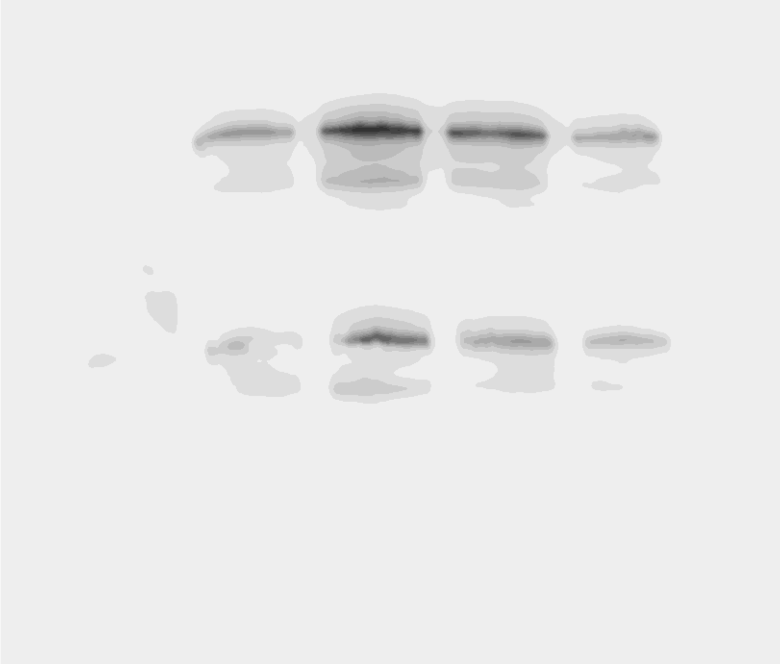

<Same samples (ran in parallel)

For all representative western blots, equal loading was confirmed using corresponding housekeeping controls (e.g., beta-actin), with all target proteins for a given series of blots resolved from the exact same sample lysates.

Figure 2A. THP1. GAPDH and histone H3.

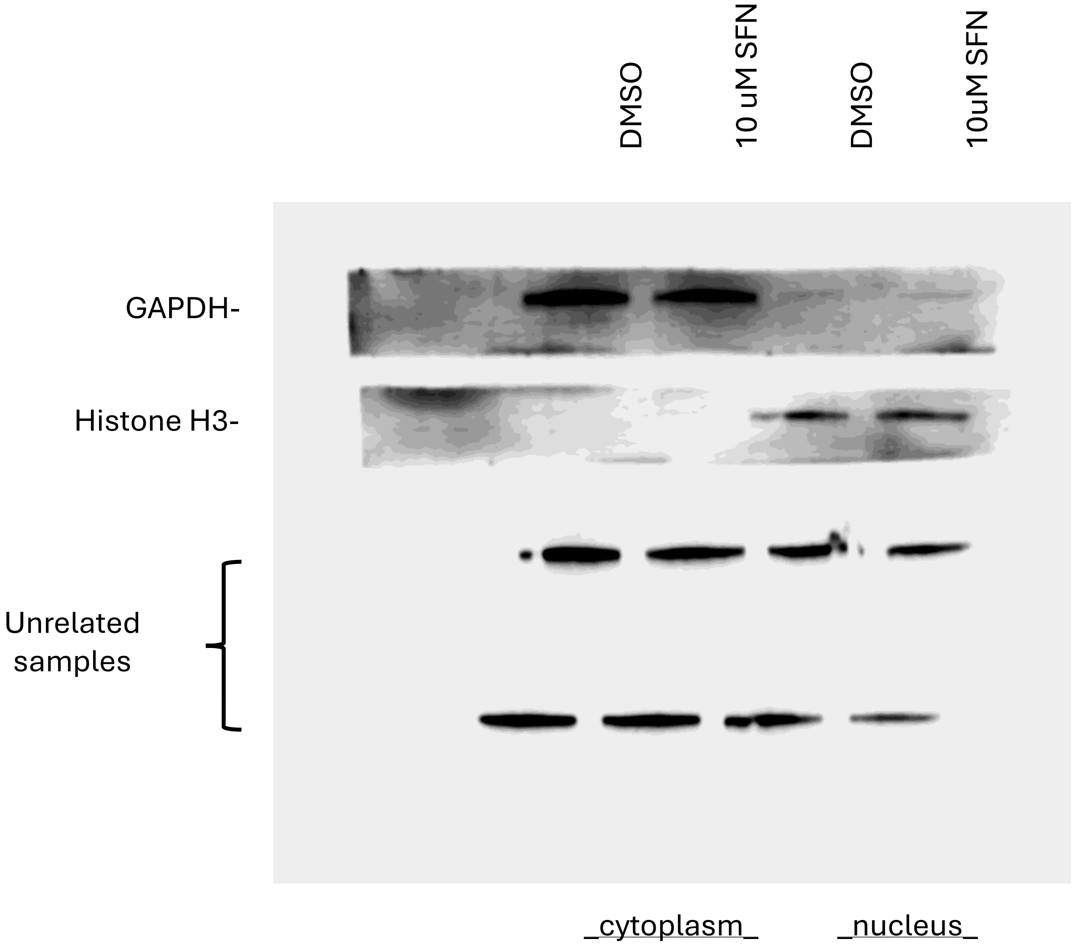

For all representative western blots, equal loading was confirmed using corresponding housekeeping controls (e.g., beta-actin), with all target proteins for a given series of blots resolved from the exact same sample lysates.

Figure 2C. THP1. P-SAMHD1.

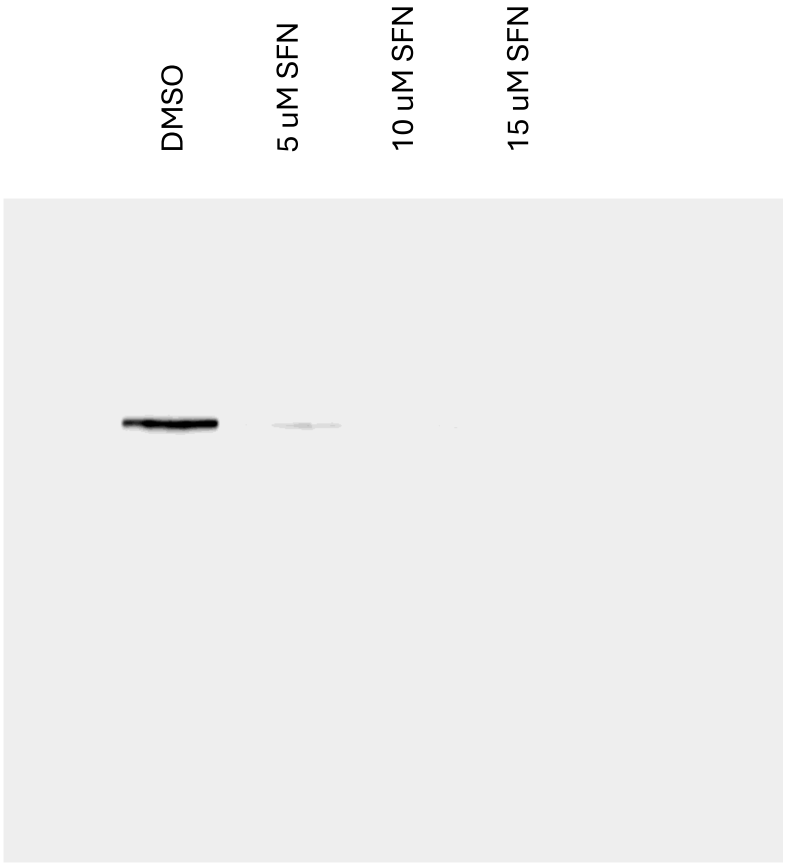

For all representative western blots, equal loading was confirmed using corresponding housekeeping controls (e.g., beta-actin), with all target proteins for a given series of blots resolved from the exact same sample lysates.

Figure 2C. THP1. total SAMHD1 and B-actin.

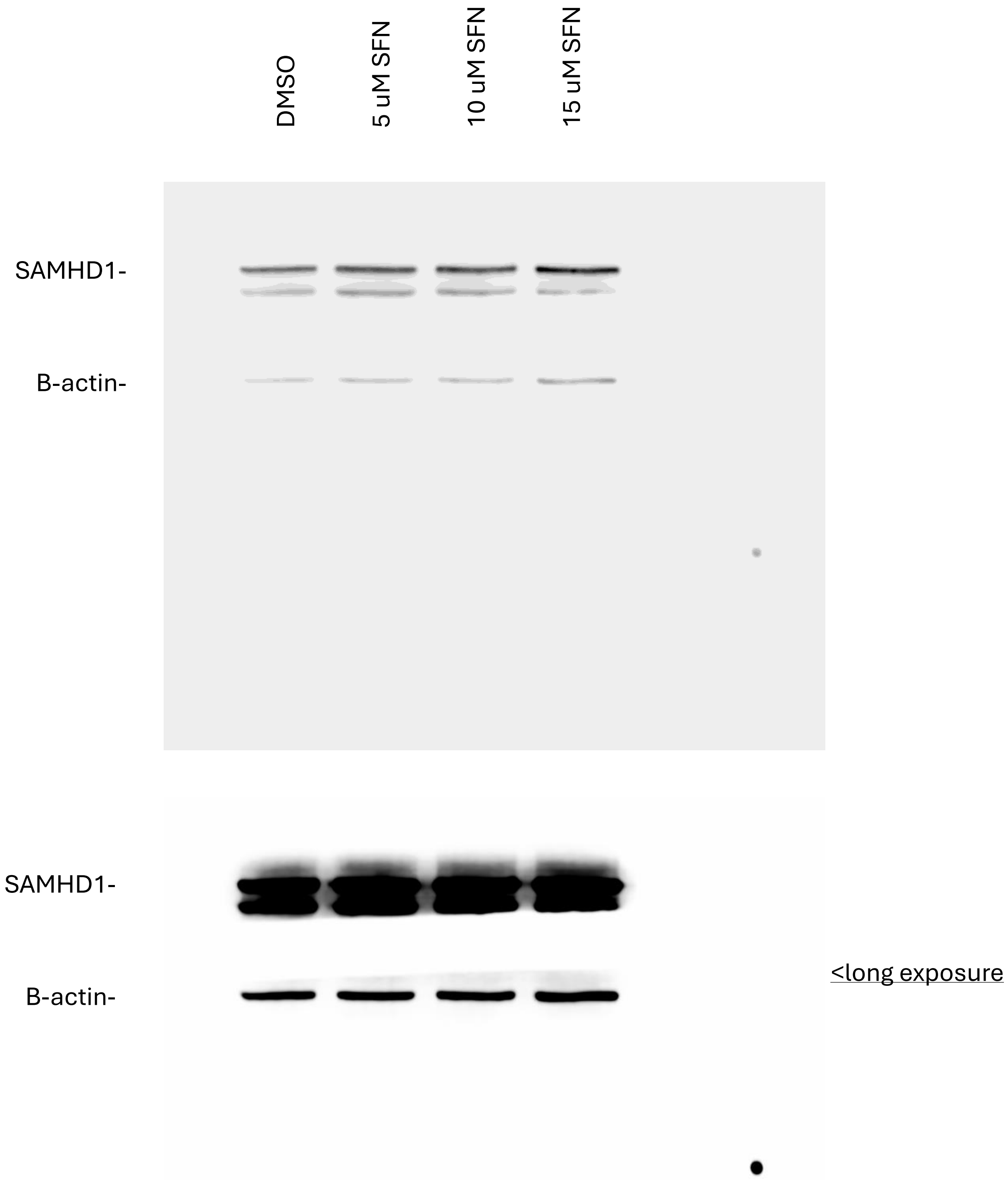

For all representative western blots, equal loading was confirmed using corresponding housekeeping controls (e.g., beta-actin), with all target proteins for a given series of blots resolved from the exact same sample lysates.

Figure 2C. THP1. P-p21.

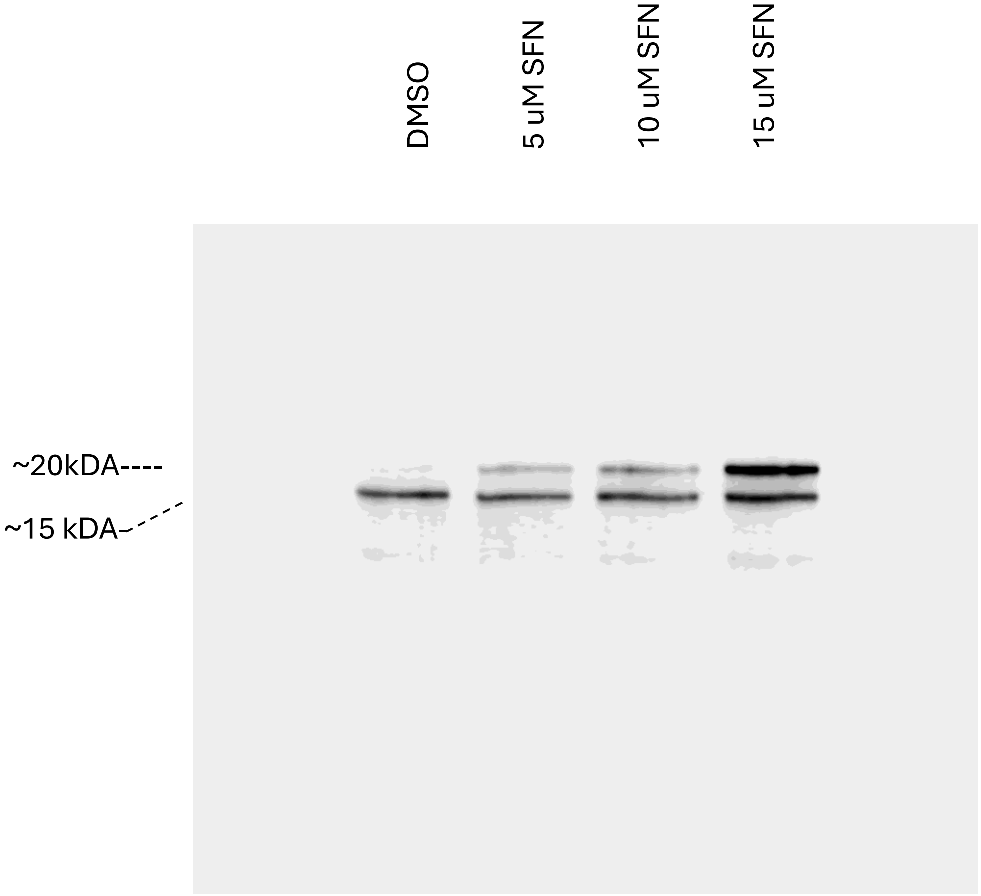

For all representative western blots, equal loading was confirmed using corresponding housekeeping controls (e.g., beta-actin), with all target proteins for a given series of blots resolved from the exact same sample lysates.

Figure 2C. THP1. total p21.

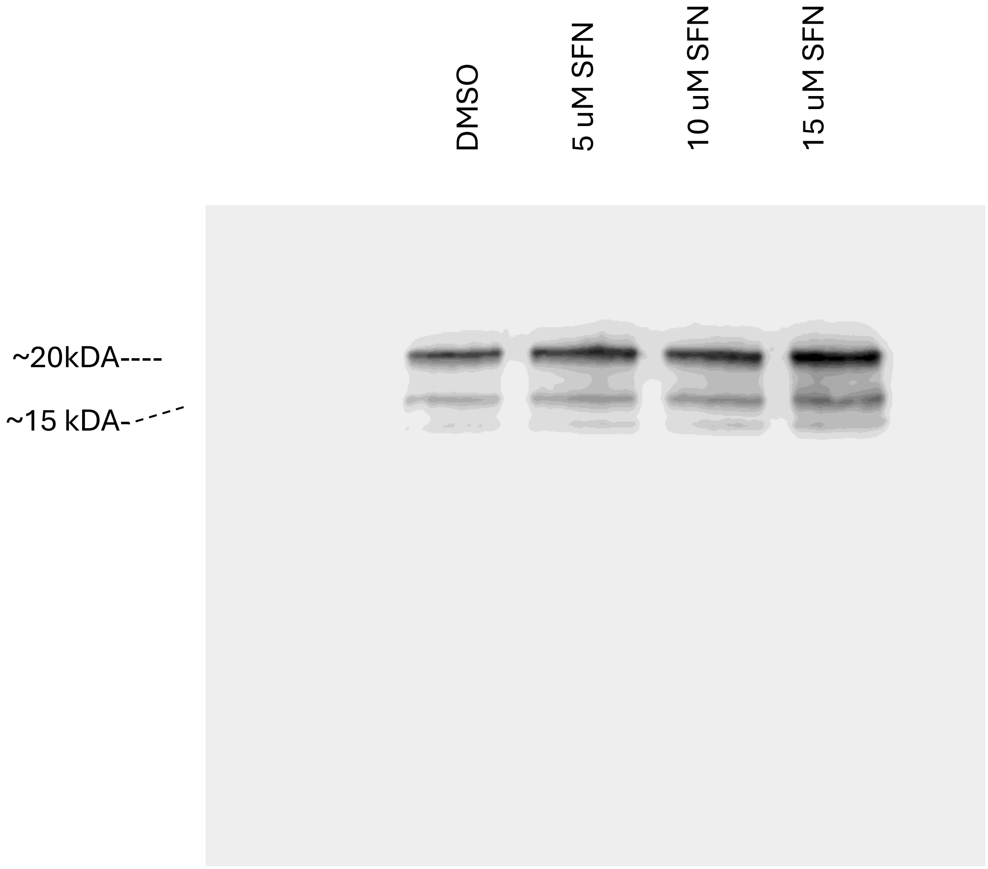

For all representative western blots, equal loading was confirmed using corresponding housekeeping controls (e.g., beta-actin), with all target proteins for a given series of blots resolved from the exact same sample lysates.

Figure 3A. hMDM male donor. P-SAMHD1.

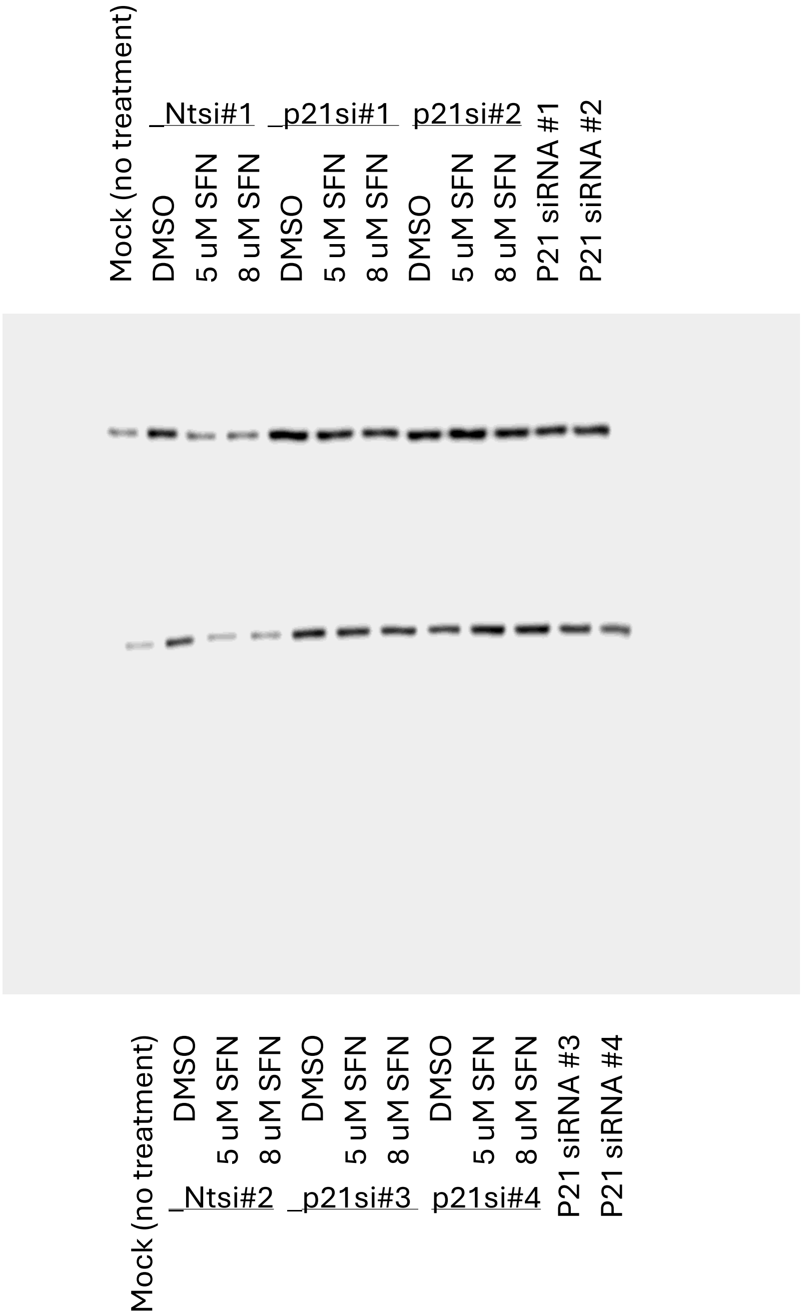

For all representative western blots, equal loading was confirmed using corresponding housekeeping controls (e.g., beta-actin), with all target proteins for a given series of blots resolved from the exact same sample lysates.

Figure 3A. hMDM male donor. B-actin.

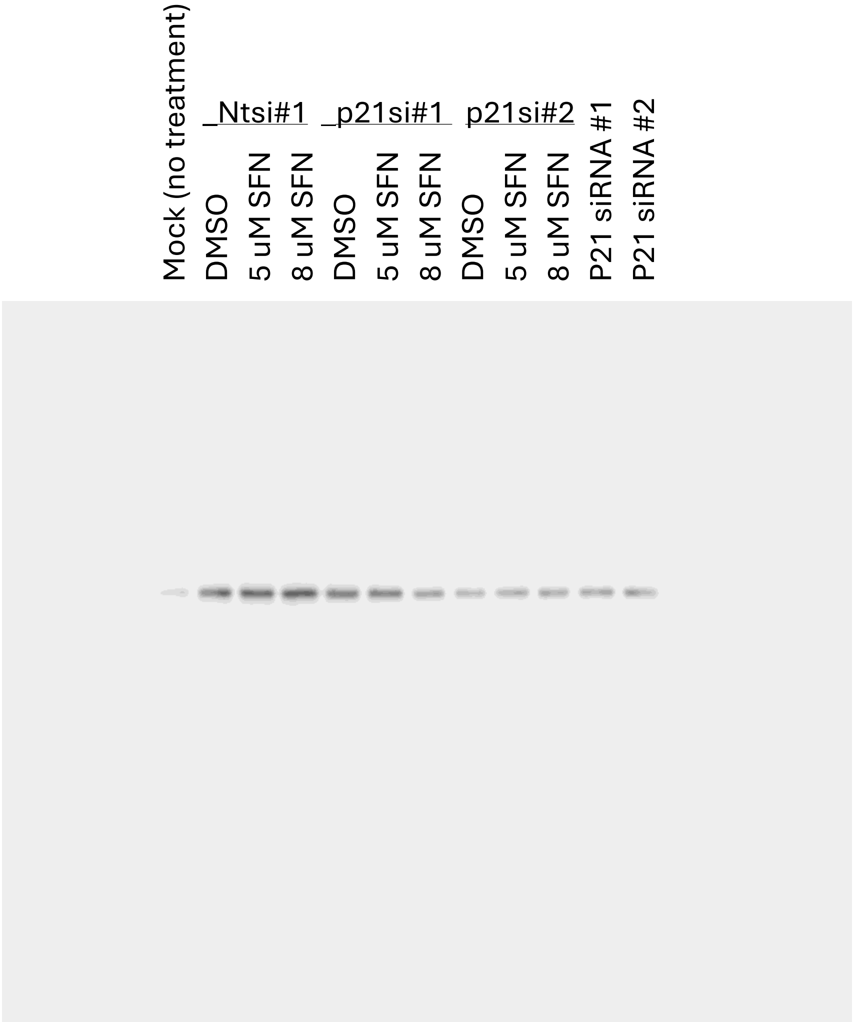

For all representative western blots, equal loading was confirmed using corresponding housekeeping controls (e.g., beta-actin), with all target proteins for a given series of blots resolved from the exact same sample lysates.

Figure 3A. hMDM male donor. B-actin. (siRNA set 2)

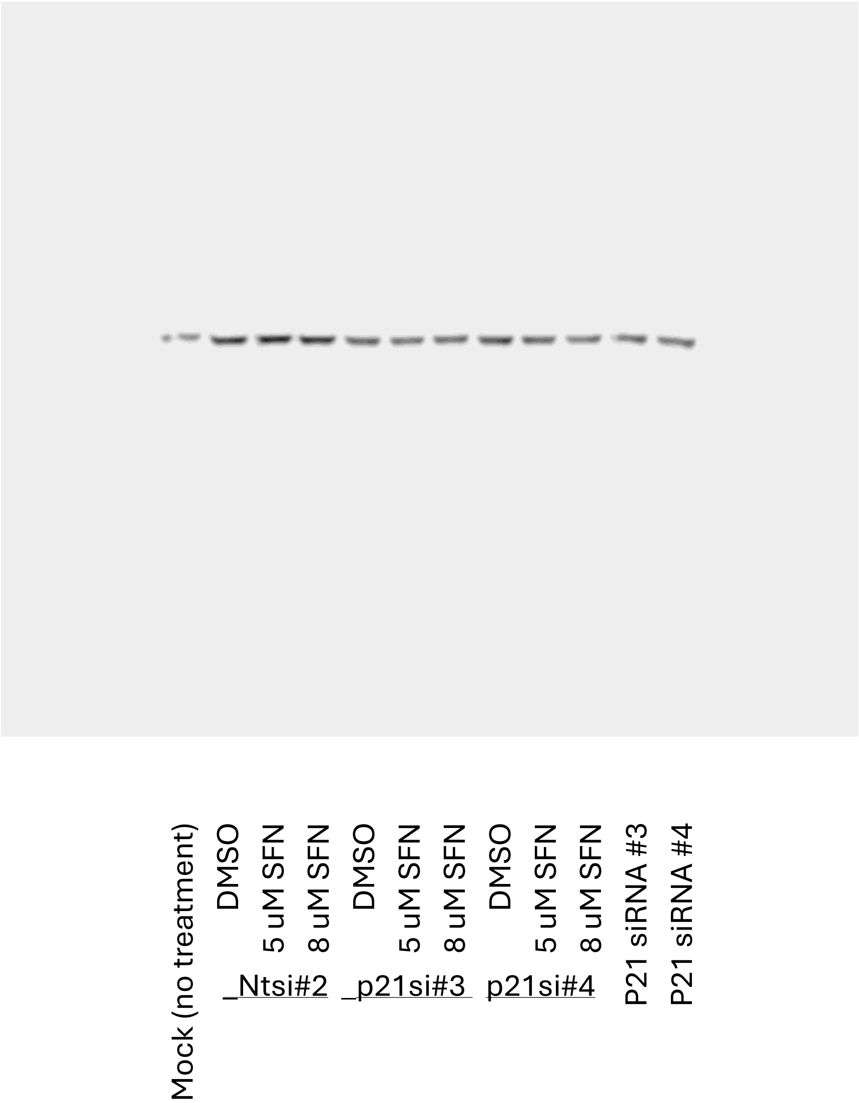

For all representative western blots, equal loading was confirmed using corresponding housekeeping controls (e.g., beta-actin), with all target proteins for a given series of blots resolved from the exact same sample lysates.

Figure 3A. hMDM male donor. p21

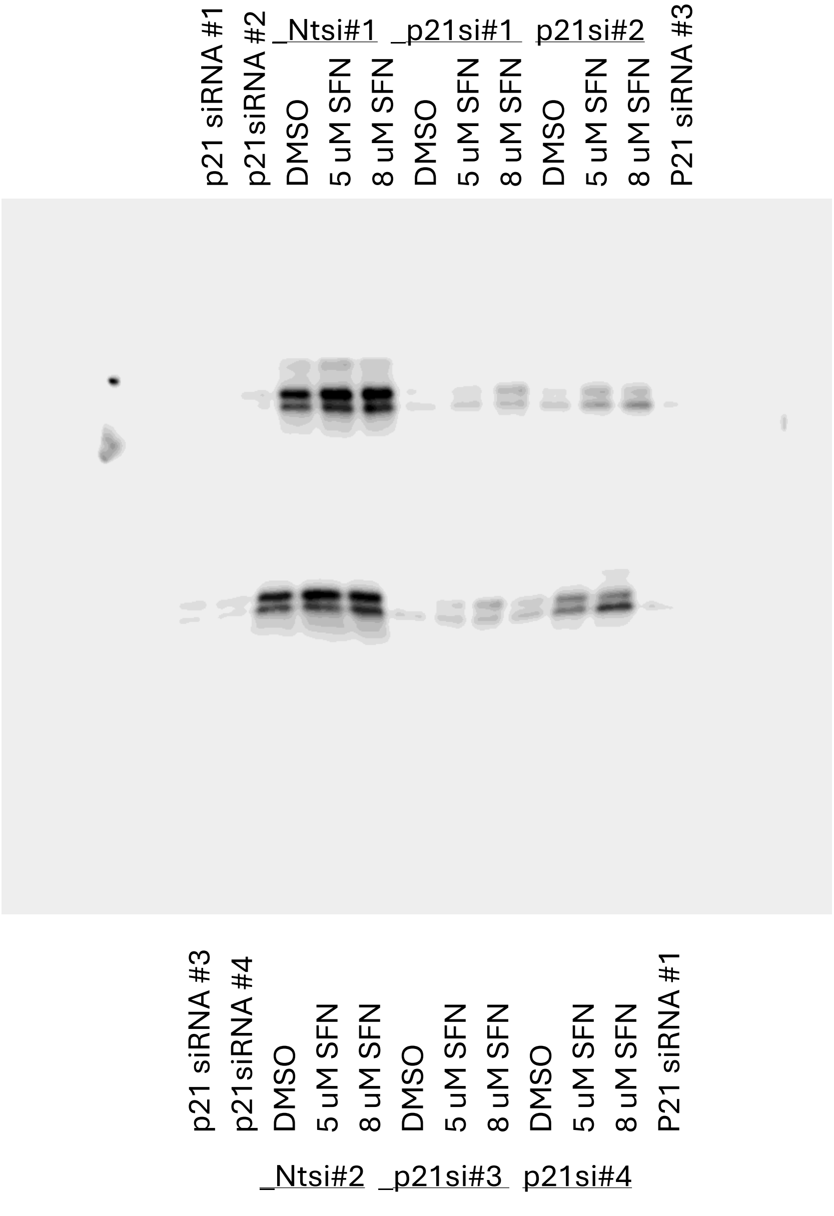

For all representative western blots, equal loading was confirmed using corresponding housekeeping controls (e.g., beta-actin), with all target proteins for a given series of blots resolved from the exact same sample lysates.

Figure 3A. hMDM male donor. Total SAMHD1.

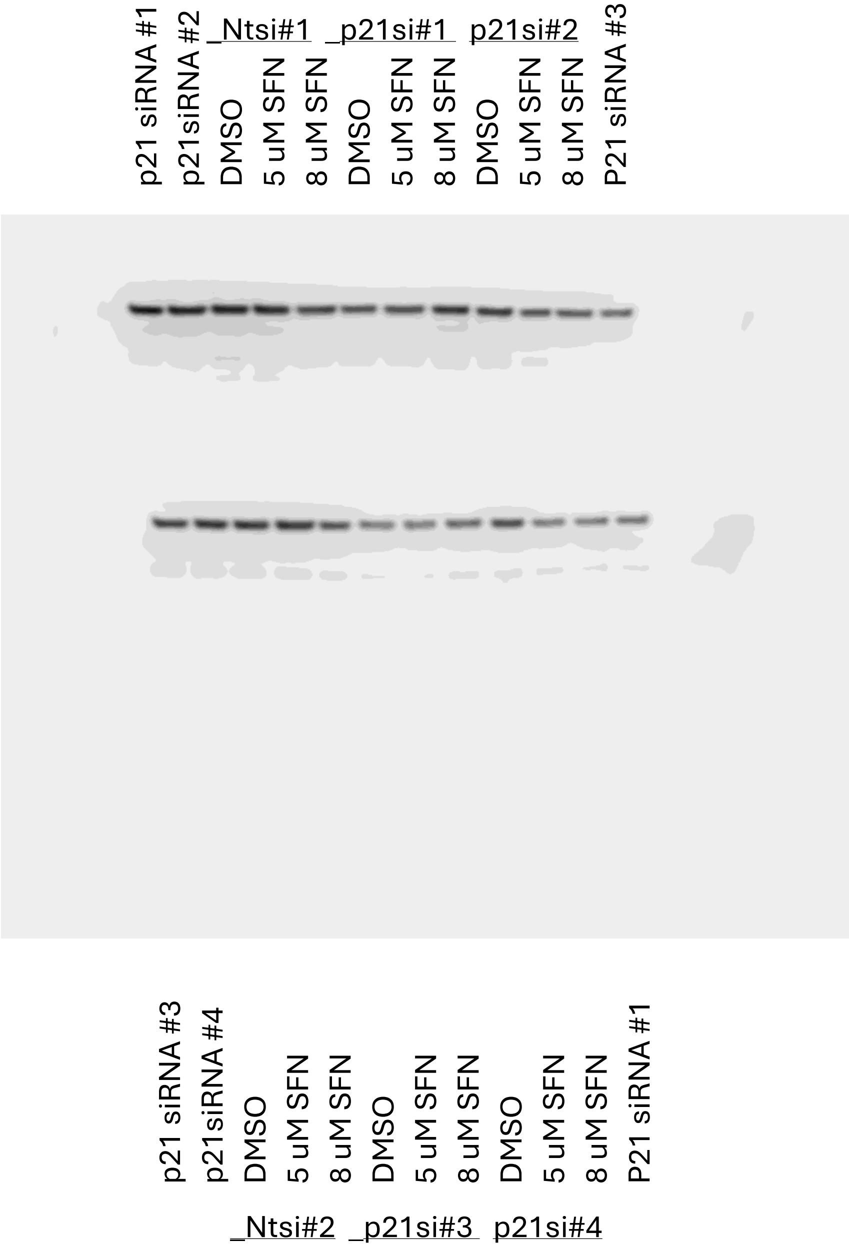

For all representative western blots, equal loading was confirmed using corresponding housekeeping controls (e.g., beta-actin), with all target proteins for a given series of blots resolved from the exact same sample lysates.

Figure 3C. hMDM other (female) donor. P-SAMHD1.

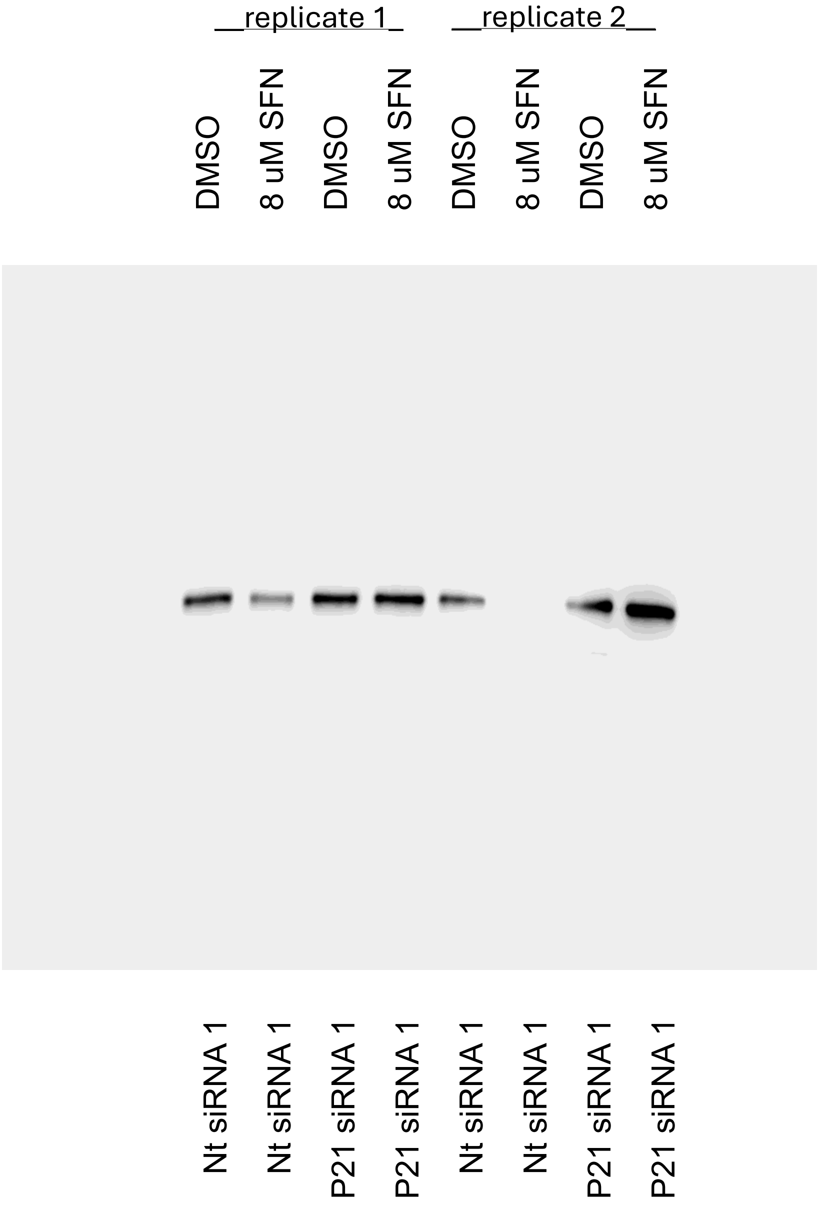

For all representative western blots, equal loading was confirmed using corresponding housekeeping controls (e.g., beta-actin), with all target proteins for a given series of blots resolved from the exact same sample lysates.

Figure 3C. hMDM other (female) donor. total SAMHD1.

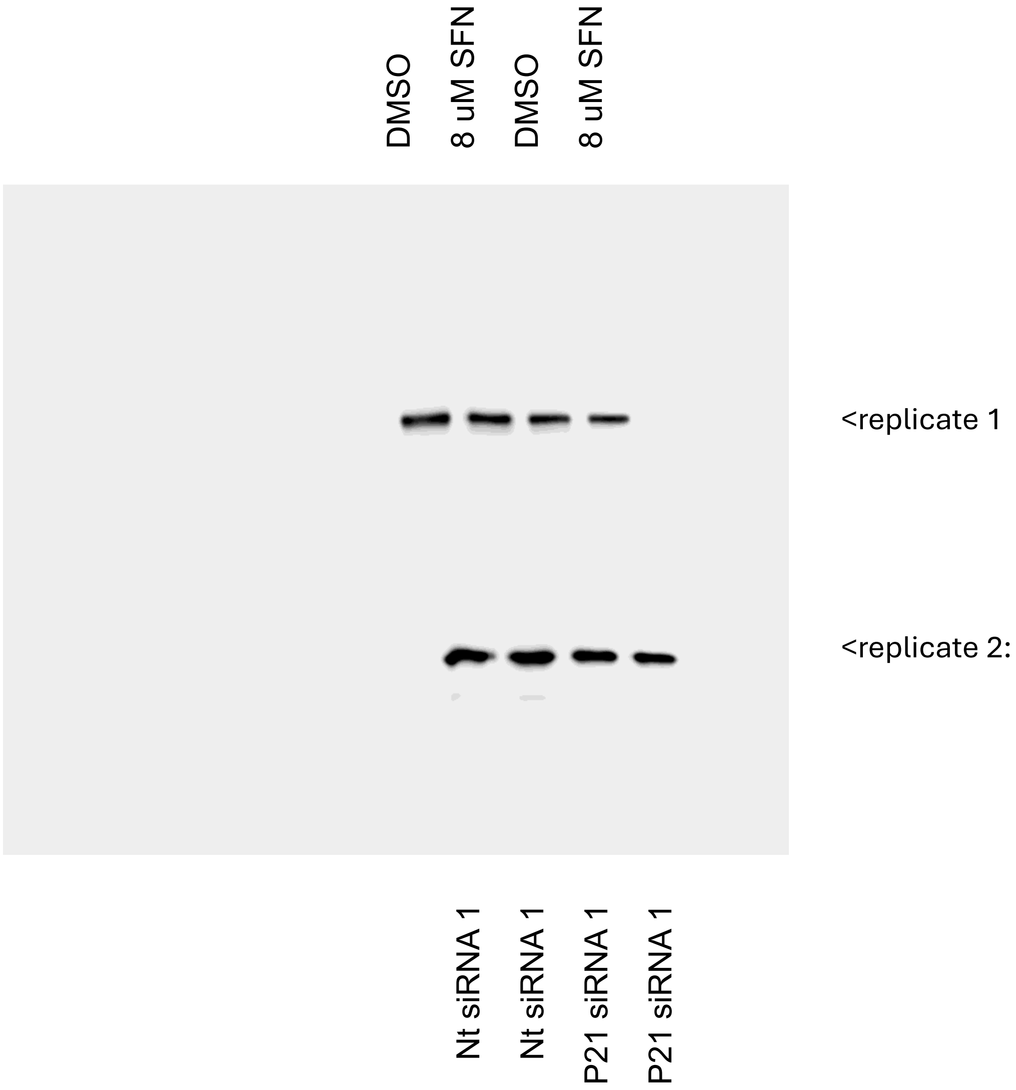

For all representative western blots, equal loading was confirmed using corresponding housekeeping controls (e.g., beta-actin), with all target proteins for a given series of blots resolved from the exact same sample lysates.

Figure 3C. hMDM other (female) donor. p21.

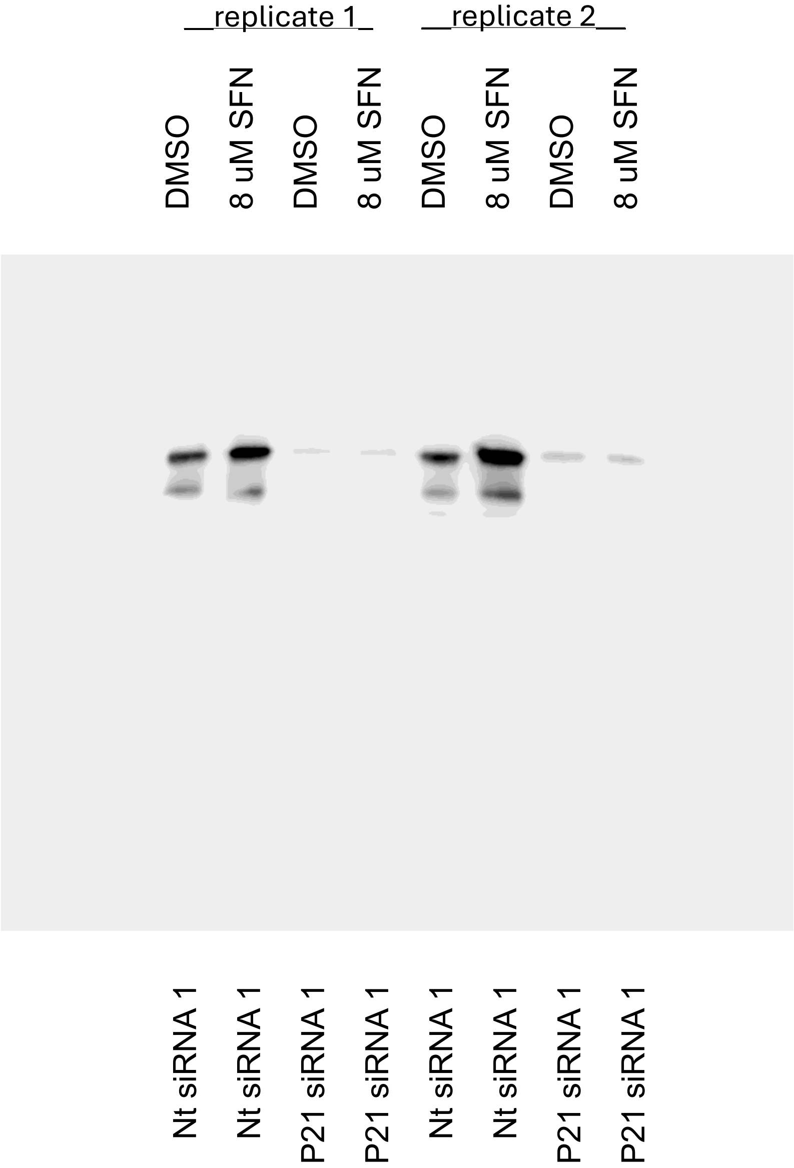

For all representative western blots, equal loading was confirmed using corresponding housekeeping controls (e.g., beta-actin), with all target proteins for a given series of blots resolved from the exact same sample lysates.

Figure 3C. hMDM other (female) donor. B-actin.

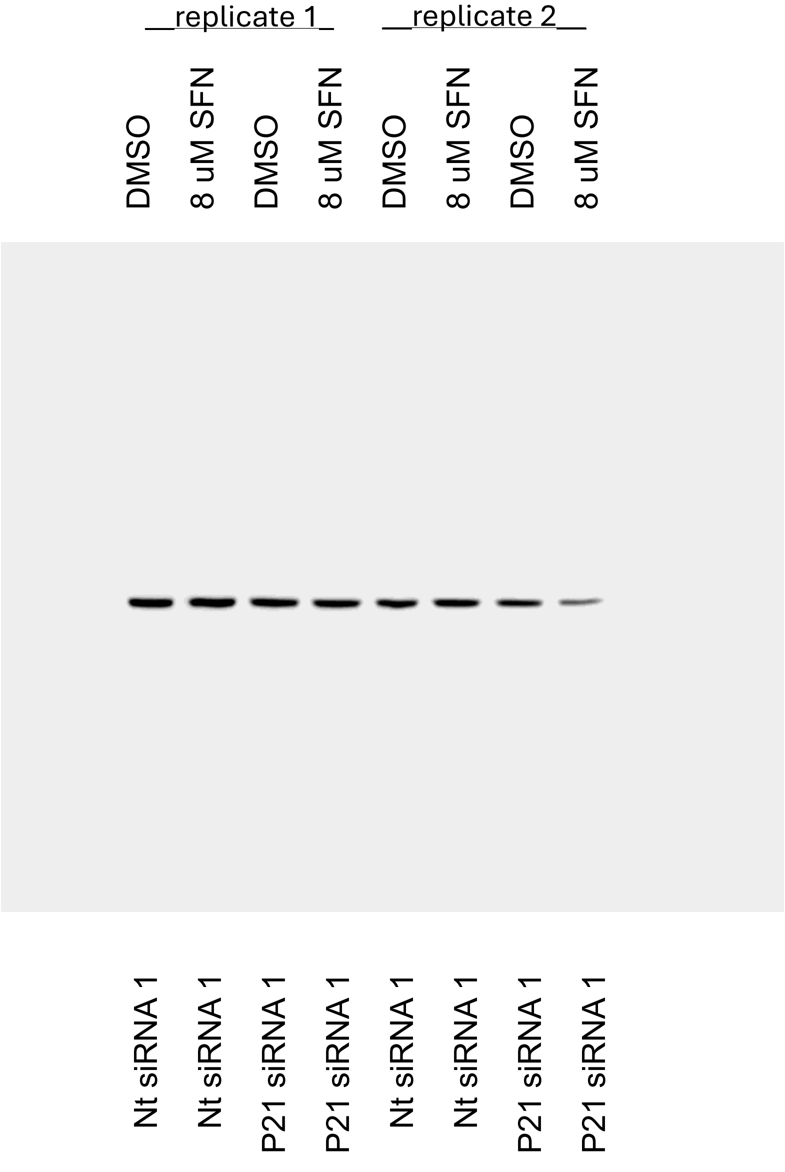

For all representative western blots, equal loading was confirmed using corresponding housekeeping controls (e.g., beta-actin), with all target proteins for a given series of blots resolved from the exact same sample lysates.

Figure 4A. CRISPR-Edited THP-1. P-SAMHD1.

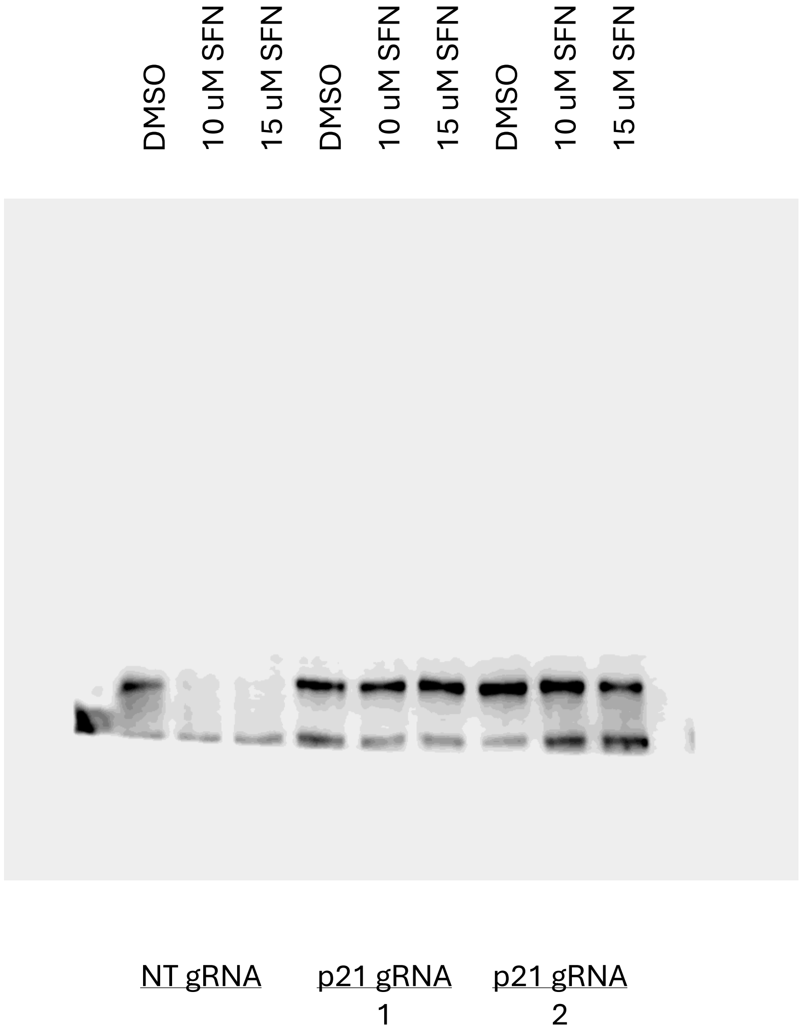

For all representative western blots, equal loading was confirmed using corresponding housekeeping controls (e.g., beta-actin), with all target proteins for a given series of blots resolved from the exact same sample lysates.

Figure 4A. CRISPR-Edited THP-1. total SAMHD1.

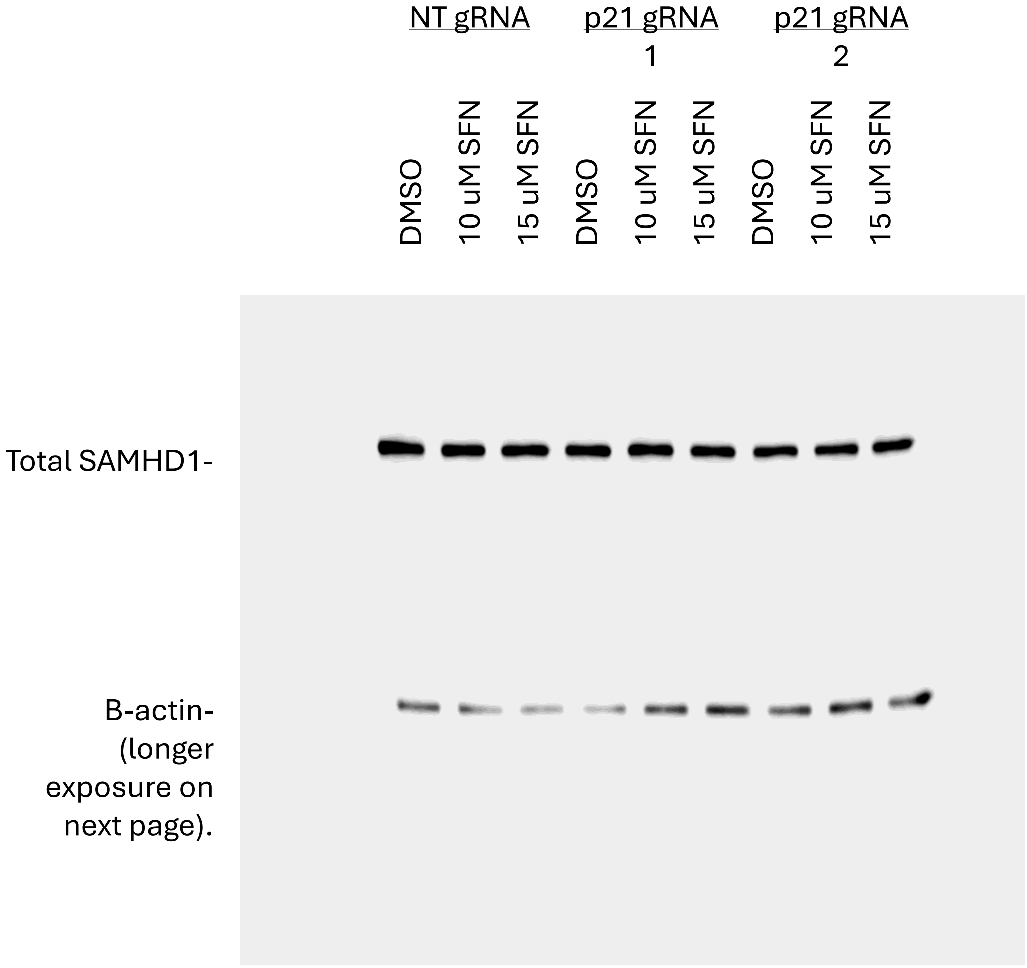

For all representative western blots, equal loading was confirmed using corresponding housekeeping controls (e.g., beta-actin), with all target proteins for a given series of blots resolved from the exact same sample lysates.

Figure 4A. CRISPR-Edited THP-1. B-actin.

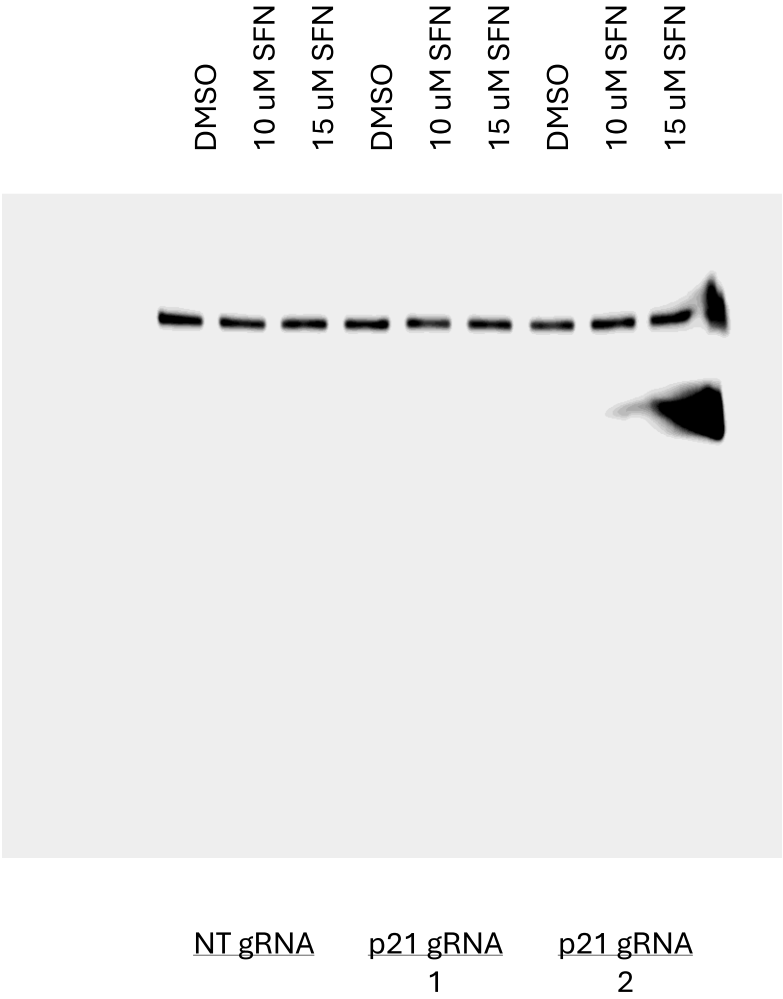

For all representative western blots, equal loading was confirmed using corresponding housekeeping controls (e.g., beta-actin), with all target proteins for a given series of blots resolved from the exact same sample lysates.

Figure 4A. CRISPR-Edited THP-1. p21

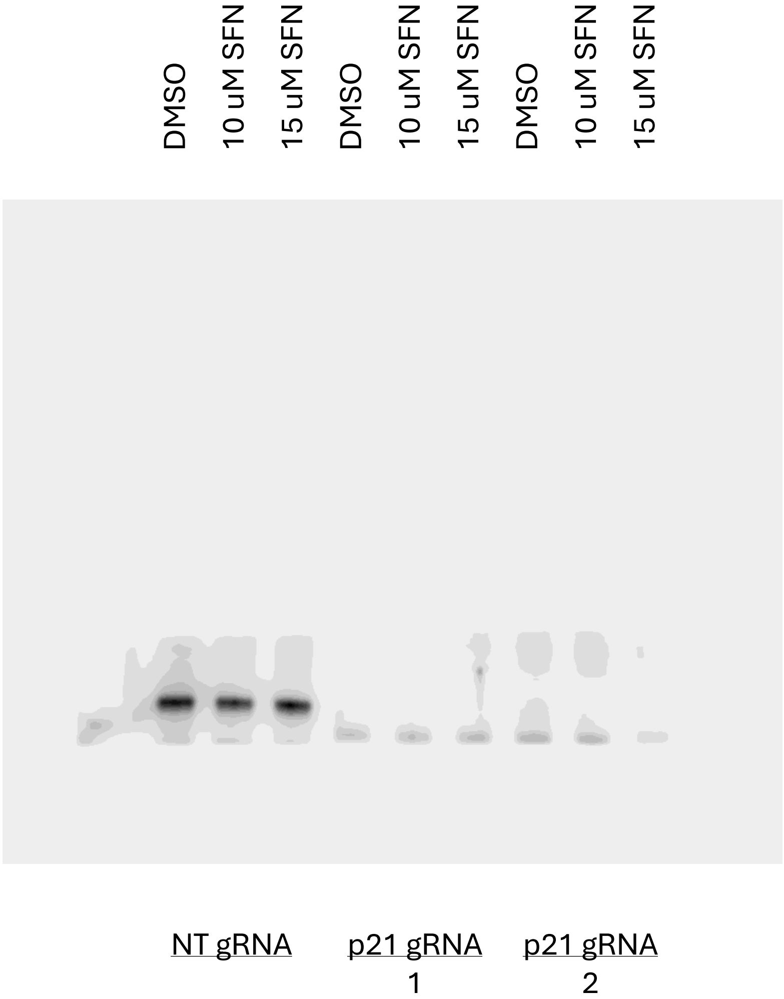

For all representative western blots, equal loading was confirmed using corresponding housekeeping controls (e.g., beta-actin), with all target proteins for a given series of blots resolved from the exact same sample lysates.

Figure S1A. CRISPR-Edited THP-1. P-SAMHD1.

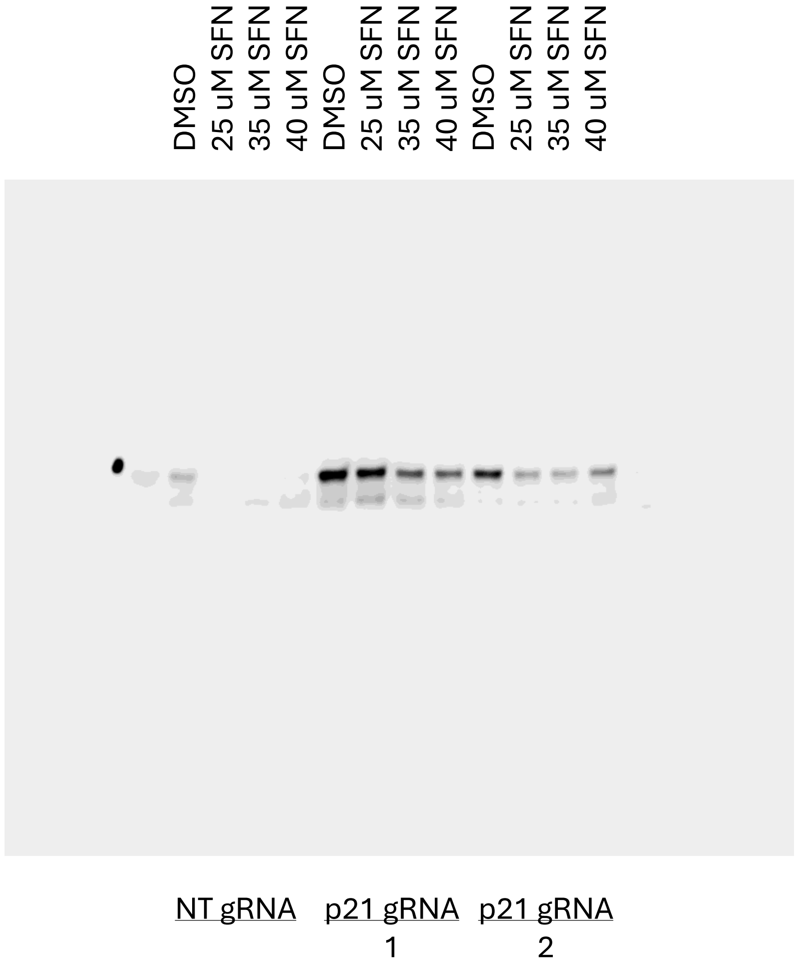

For all representative western blots, equal loading was confirmed using corresponding housekeeping controls (e.g., beta-actin), with all target proteins for a given series of blots resolved from the exact same sample lysates.

Figure S1A. CRISPR-Edited THP-1. total SAMHD 1.

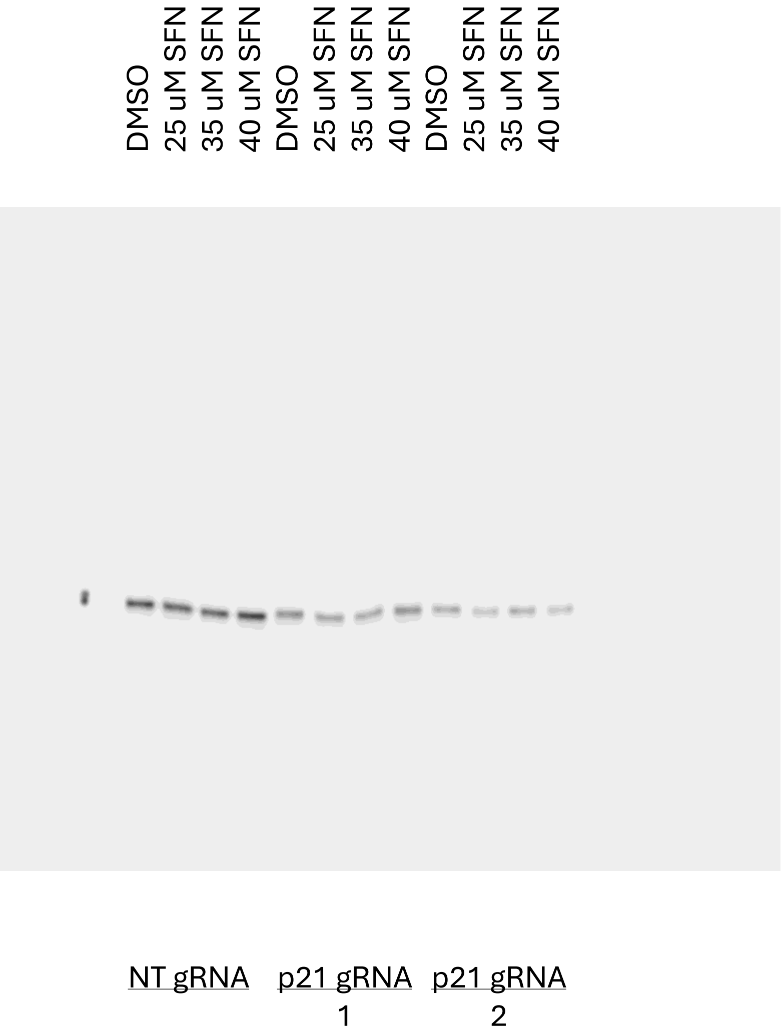

For all representative western blots, equal loading was confirmed using corresponding housekeeping controls (e.g., beta-actin), with all target proteins for a given series of blots resolved from the exact same sample lysates.

Figure S1A. CRISPR-Edited THP-1. total SAMHD 1.

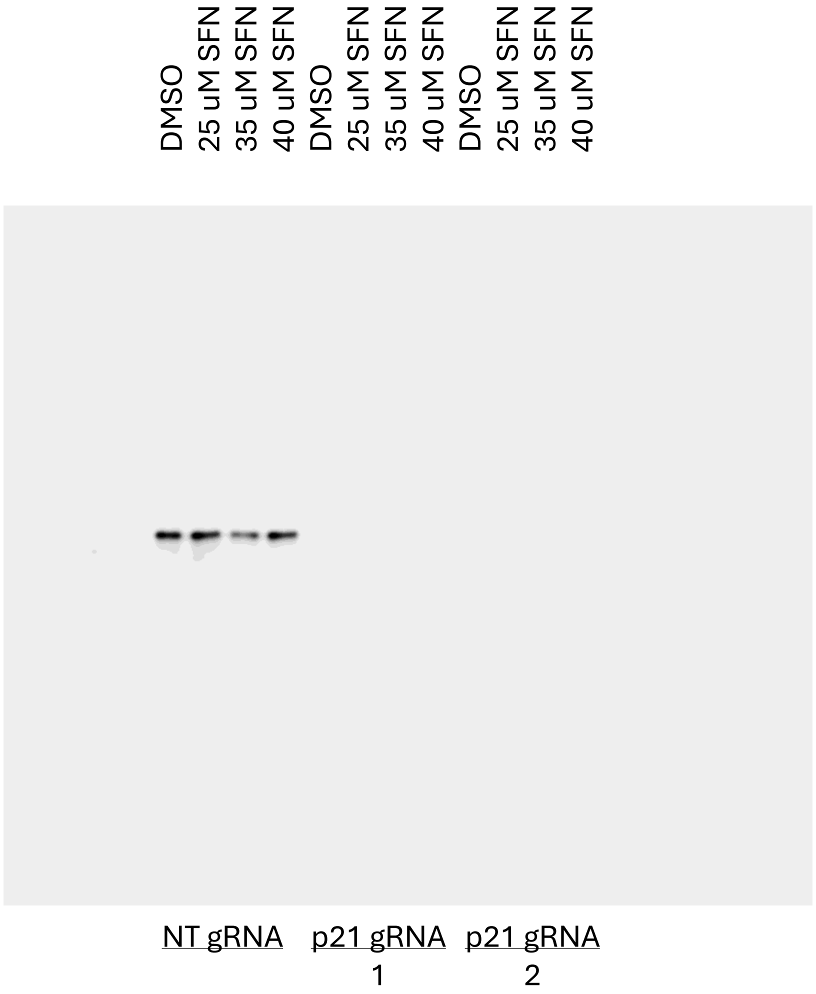

For all representative western blots, equal loading was confirmed using corresponding housekeeping controls (e.g., beta-actin), with all target proteins for a given series of blots resolved from the exact same sample lysates.

Figure S1A. CRISPR-Edited THP-1. total SAMHD 1.

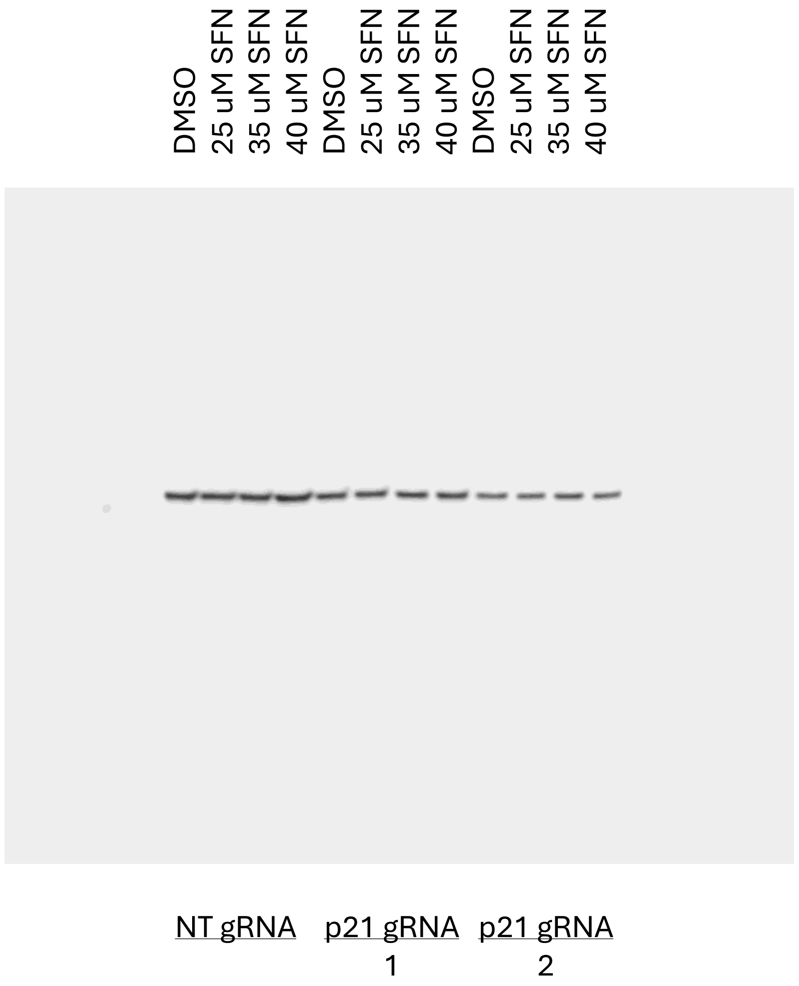

For all representative western blots, equal loading was confirmed using corresponding housekeeping controls (e.g., beta-actin), with all target proteins for a given series of blots resolved from the exact same sample lysates.

Figure S2A. 786-O. P-SAMHD1.

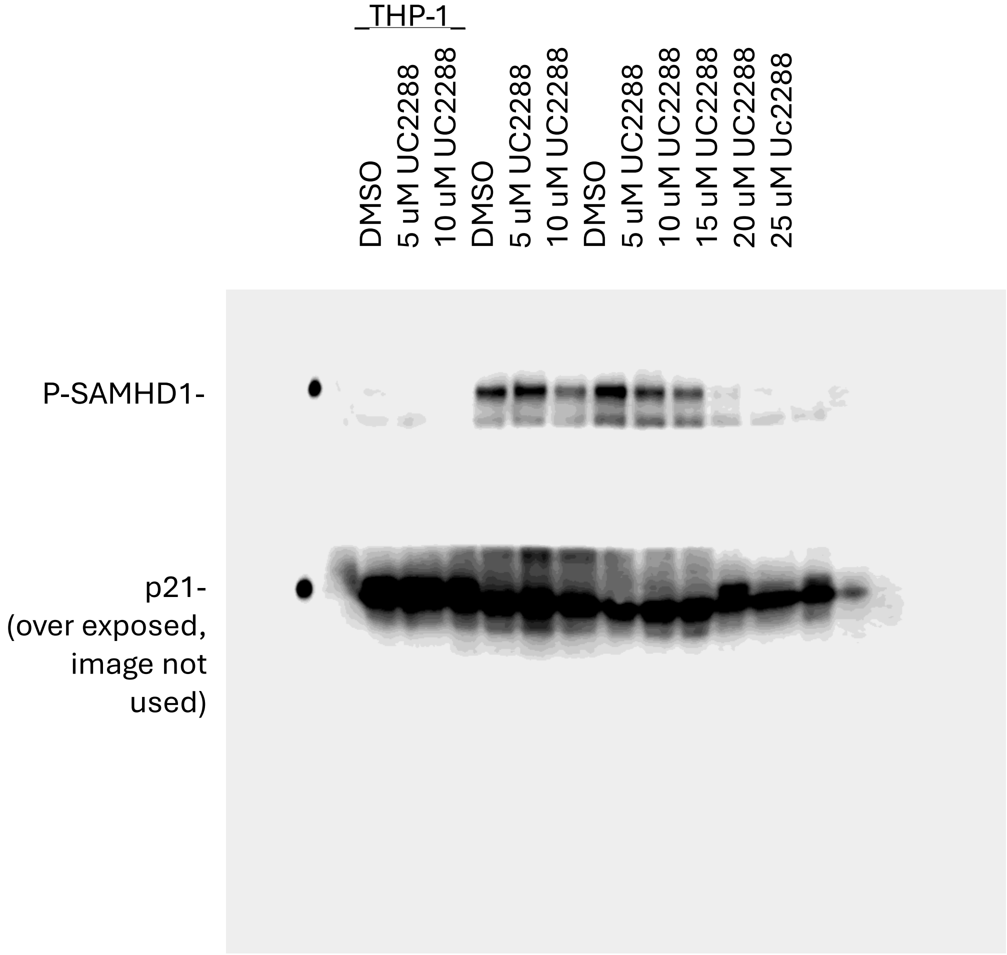

For all representative western blots, equal loading was confirmed using corresponding housekeeping controls (e.g., beta-actin), with all target proteins for a given series of blots resolved from the exact same sample lysates.

Figure S2A. 786-O. total SAMHD1.

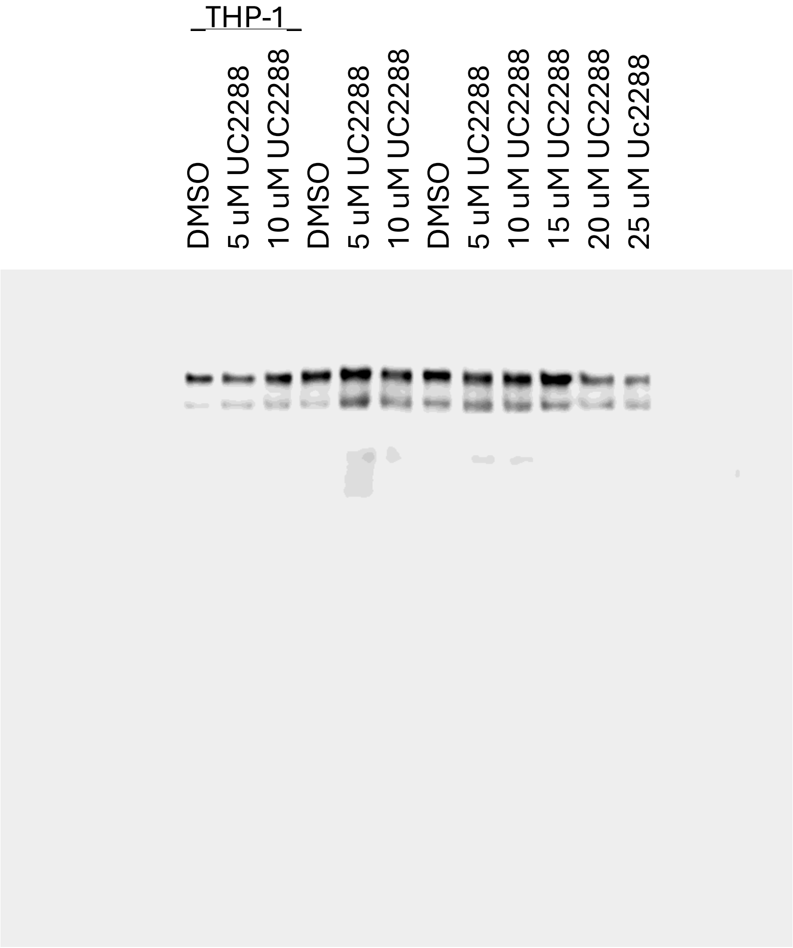

For all representative western blots, equal loading was confirmed using corresponding housekeeping controls (e.g., beta-actin), with all target proteins for a given series of blots resolved from the exact same sample lysates.

Figure S2A. 786-O. p21.

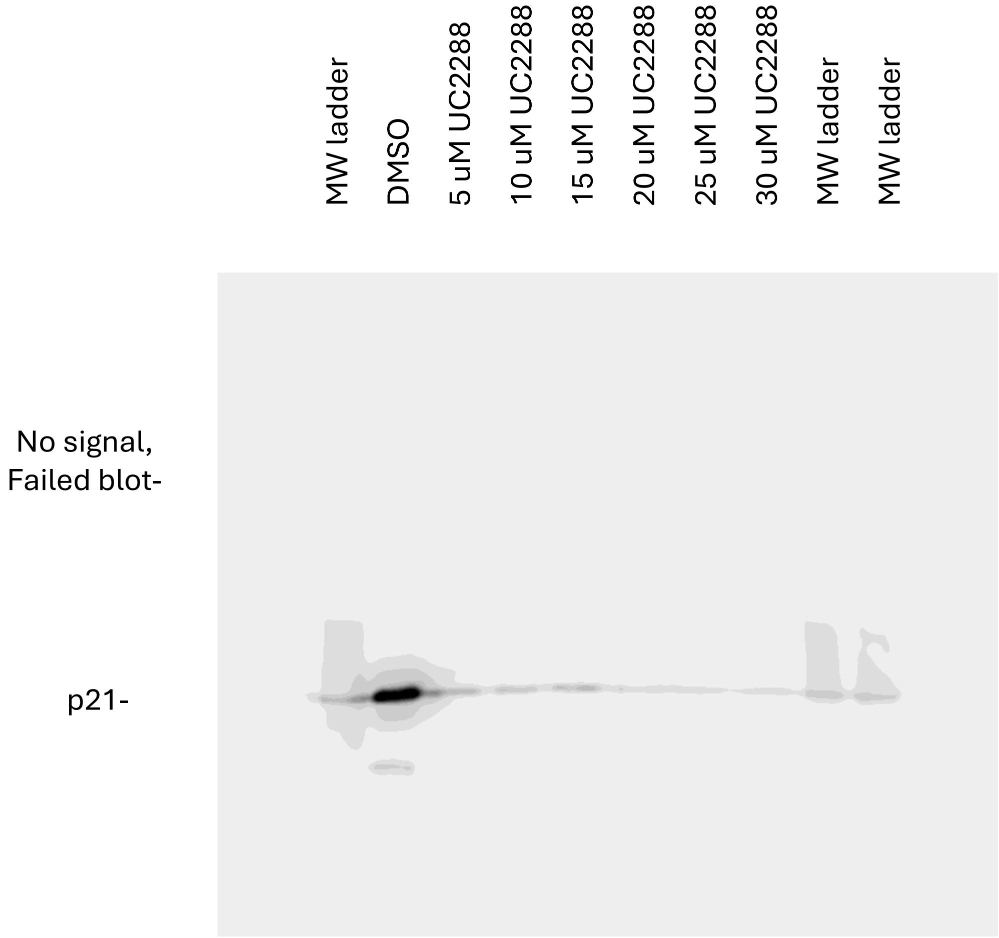

For all representative western blots, equal loading was confirmed using corresponding housekeeping controls (e.g., beta-actin), with all target proteins for a given series of blots resolved from the exact same sample lysates.

Figure S2A. 786-O. B-actin.

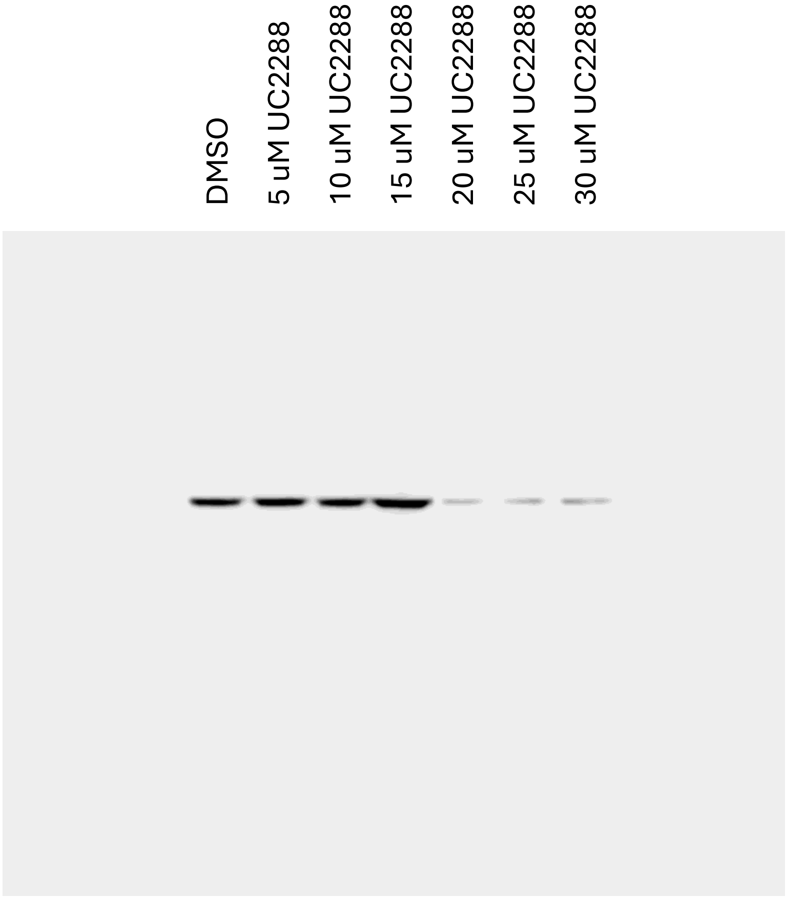

For all representative western blots, equal loading was confirmed using corresponding housekeeping controls (e.g., beta-actin), with all target proteins for a given series of blots resolved from the exact same sample lysates.

Figure S2A. hMDM. P-SAMHD1 (also see next page).

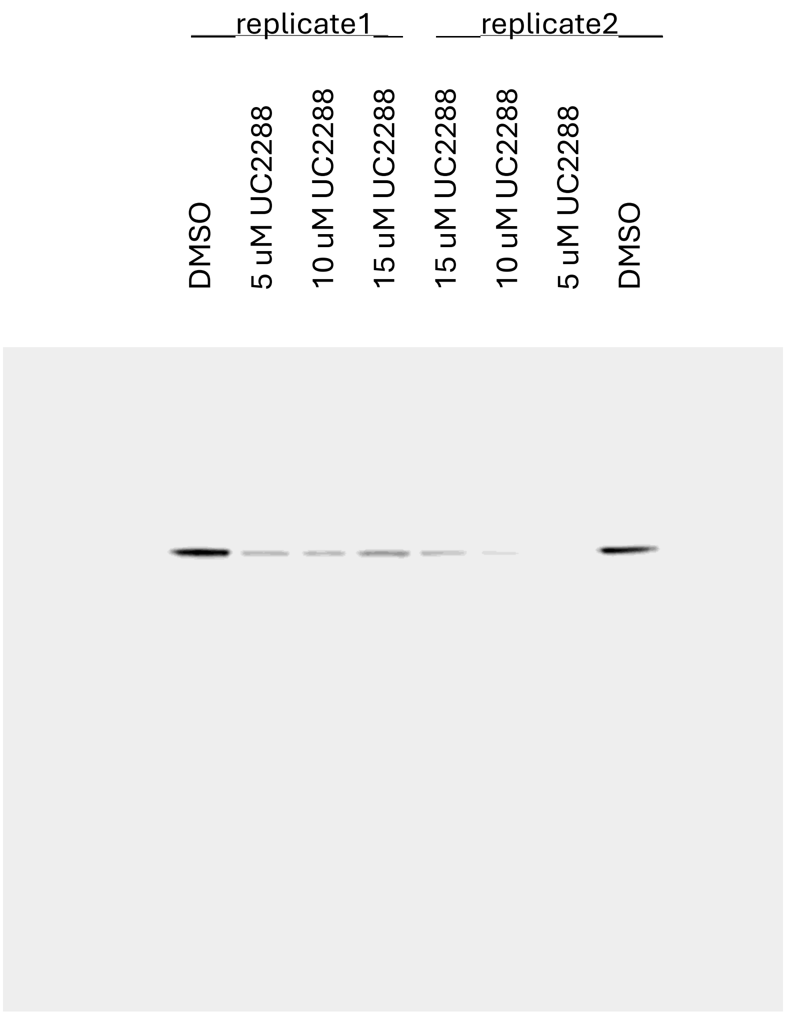

For all representative western blots, equal loading was confirmed using corresponding housekeeping controls (e.g., beta-actin), with all target proteins for a given series of blots resolved from the exact same sample lysates.

Figure S2A. hMDM.

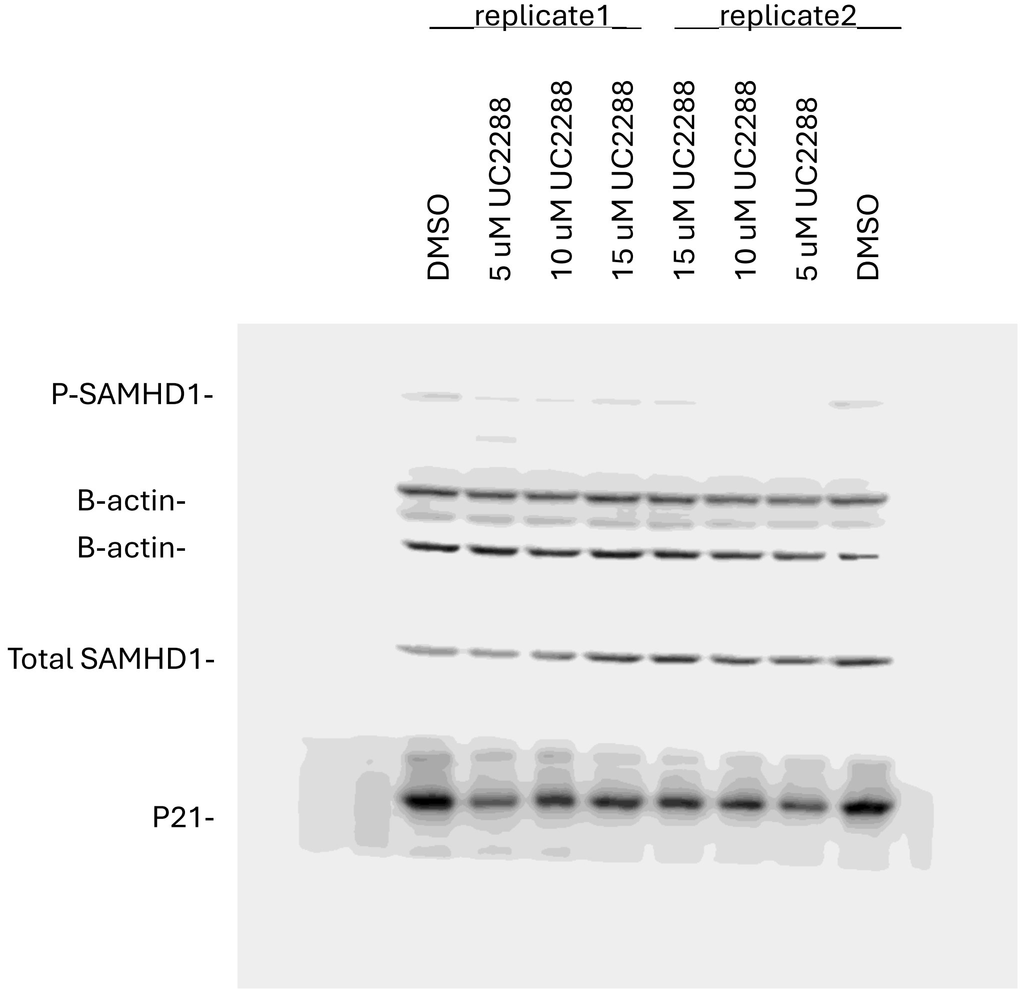

For all representative western blots, equal loading was confirmed using corresponding housekeeping controls (e.g., beta-actin), with all target proteins for a given series of blots resolved from the exact same sample lysates.

Figure S2A. THP-1. P-SAMHD1.

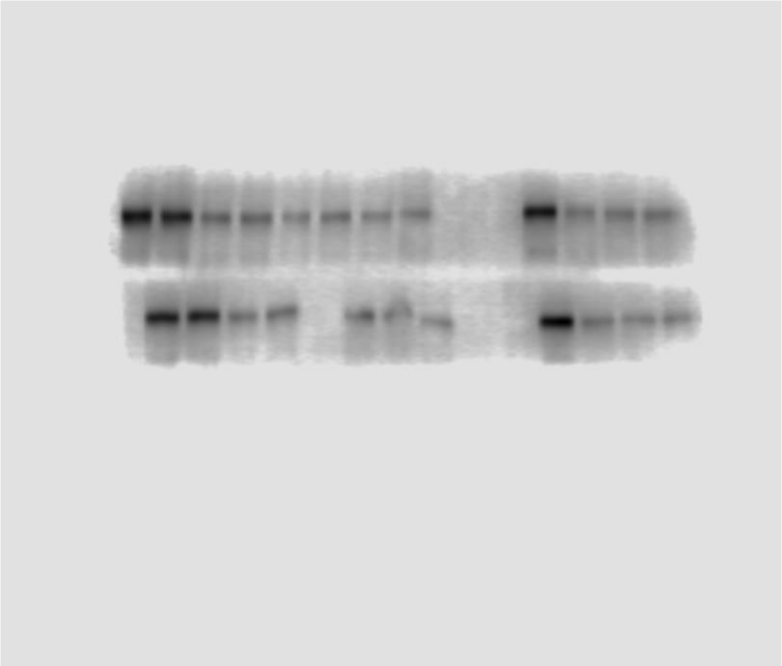

For all representative western blots, equal loading was confirmed using corresponding housekeeping controls (e.g., beta-actin), with all target proteins for a given series of blots resolved from the exact same sample lysates.

Figure S2A. THP-1. total SAMHD1.

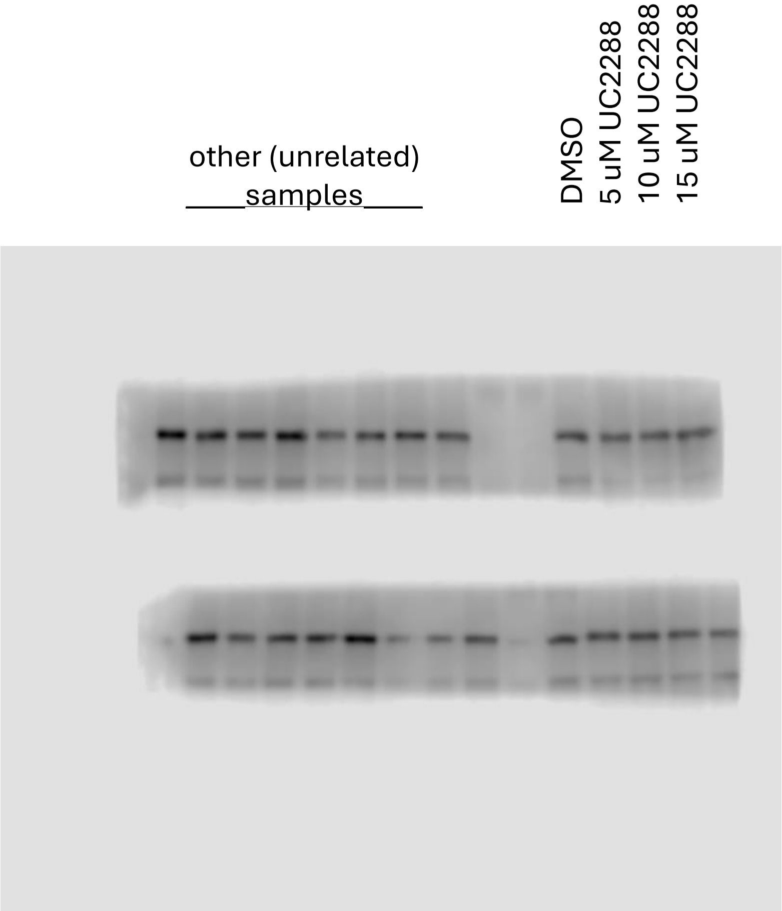

-Replicate bottom blot loading mismatch. Bottom membrane not used.

For all representative western blots, equal loading was confirmed using corresponding housekeeping controls (e.g., beta-actin), with all target proteins for a given series of blots resolved from the exact same sample lysates.

Figure S2A. THP-1. p21.

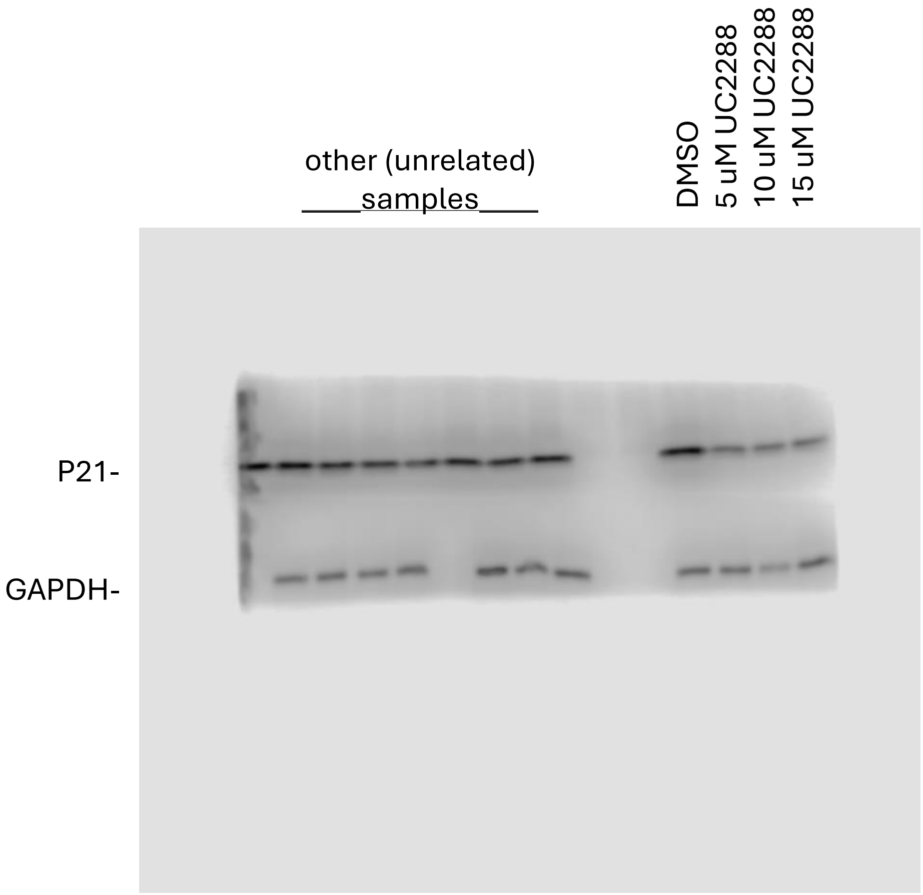

For all representative western blots, equal loading was confirmed using corresponding housekeeping controls (e.g., beta-actin), with all target proteins for a given series of blots resolved from the exact same sample lysates.

Figure S2A. THP-1. B-actin.

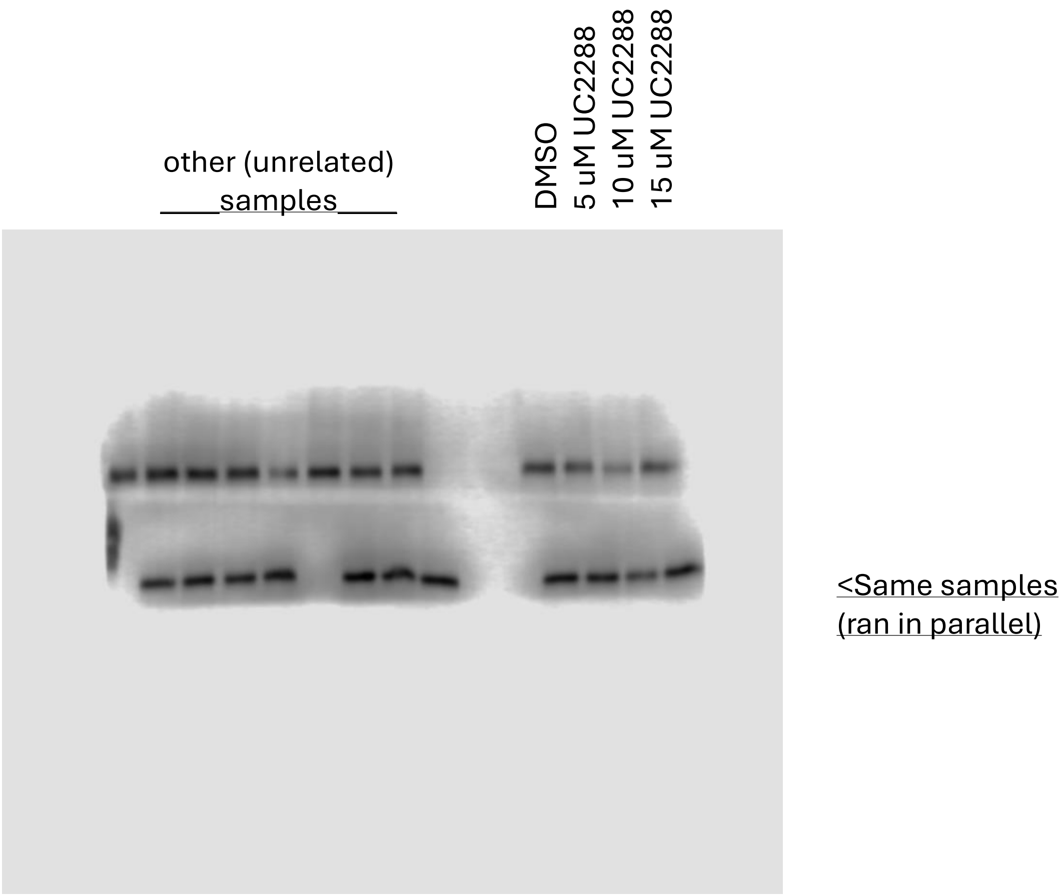

For all representative western blots, equal loading was confirmed using corresponding housekeeping controls (e.g., beta-actin), with all target proteins for a given series of blots resolved from the exact same sample lysates.

Figure S2C. hMDM. P-SAMHD1 + total SAMHD1

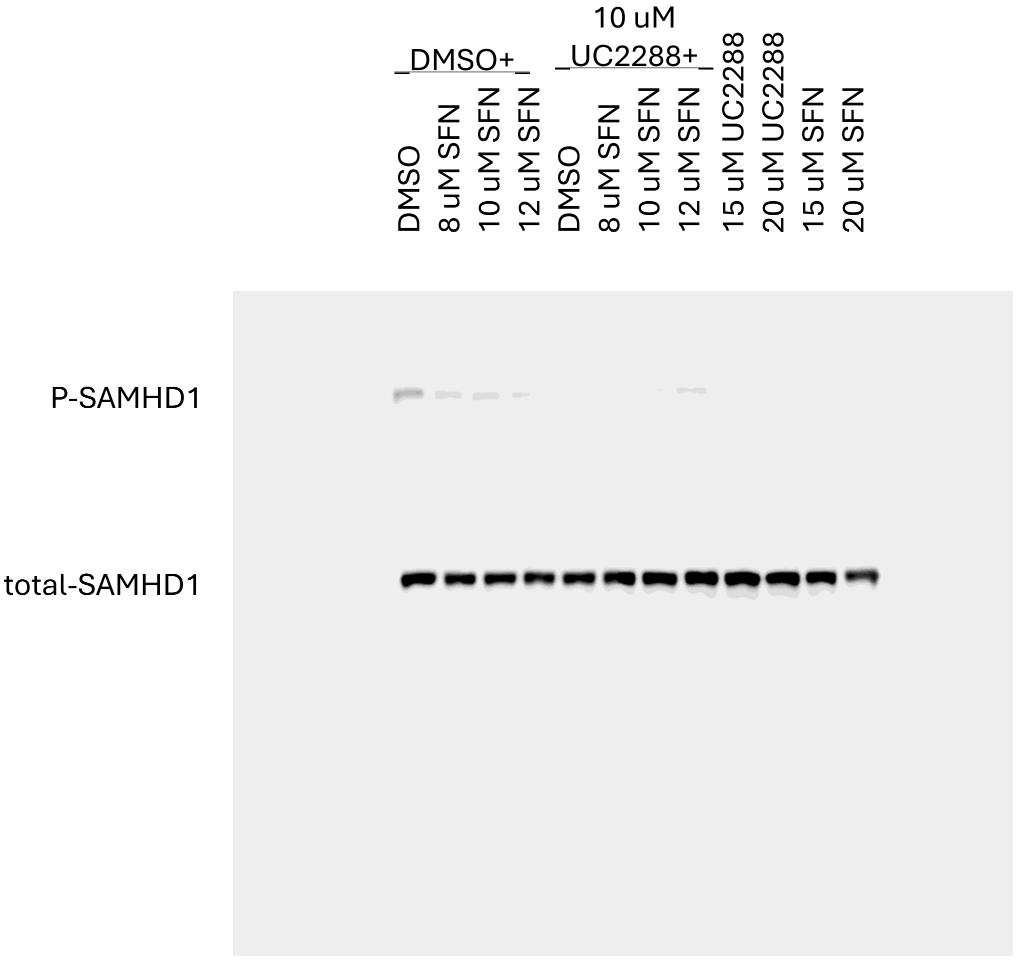

For all representative western blots, equal loading was confirmed using corresponding housekeeping controls (e.g., beta-actin), with all target proteins for a given series of blots resolved from the exact same sample lysates.

Figure S2C. hMDM. P21.

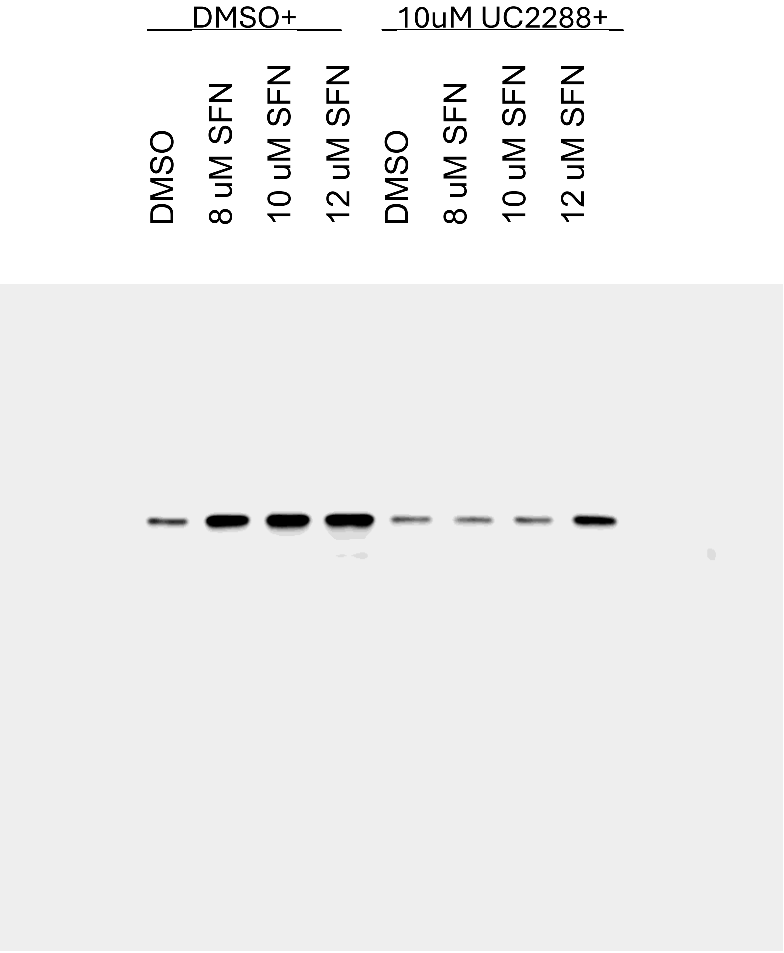

For all representative western blots, equal loading was confirmed using corresponding housekeeping controls (e.g., beta-actin), with all target proteins for a given series of blots resolved from the exact same sample lysates.

Figure S2C. hMDM. B-actin.

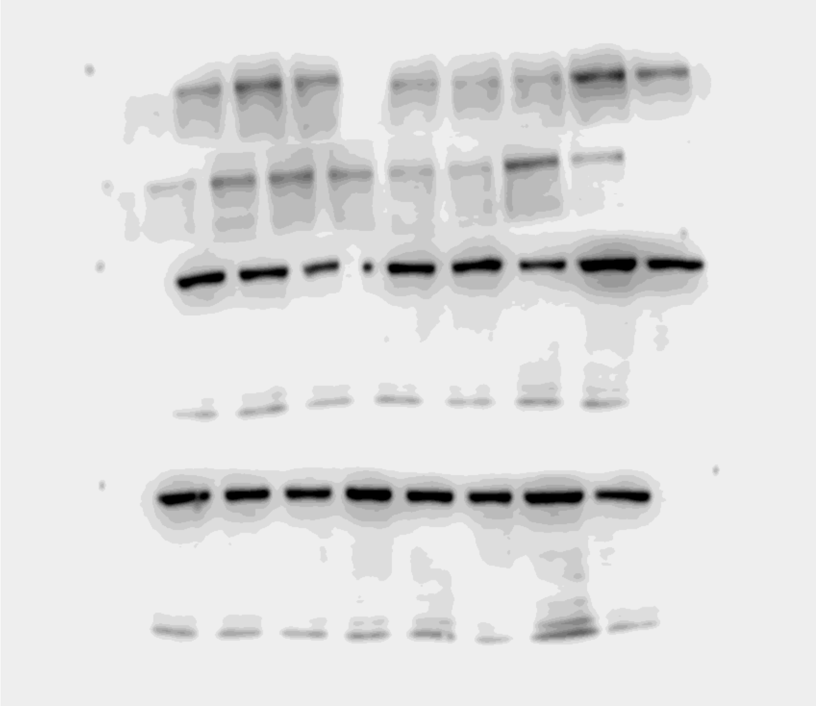

For all representative western blots, equal loading was confirmed using corresponding housekeeping controls (e.g., beta-actin), with all target proteins for a given series of blots resolved from the exact same sample lysates.

Figure S2C. THP-1. P-SAMHD1.

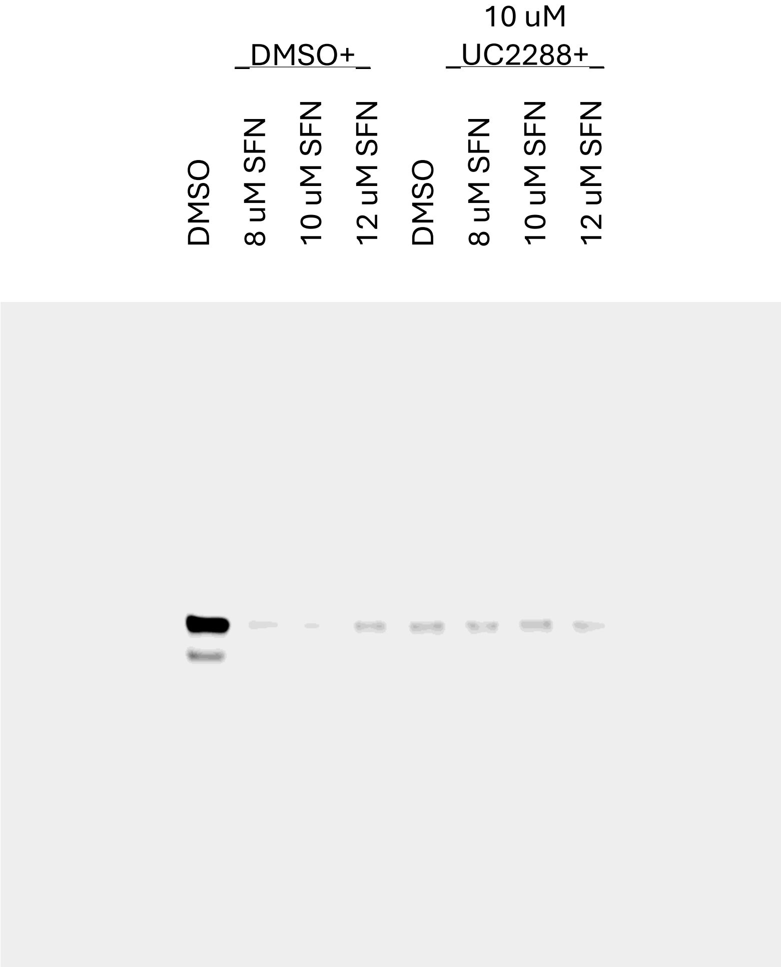

For all representative western blots, equal loading was confirmed using corresponding housekeeping controls (e.g., beta-actin), with all target proteins for a given series of blots resolved from the exact same sample lysates.

Figure S2C. THP1. total SAMHD1, B-actin, p21.

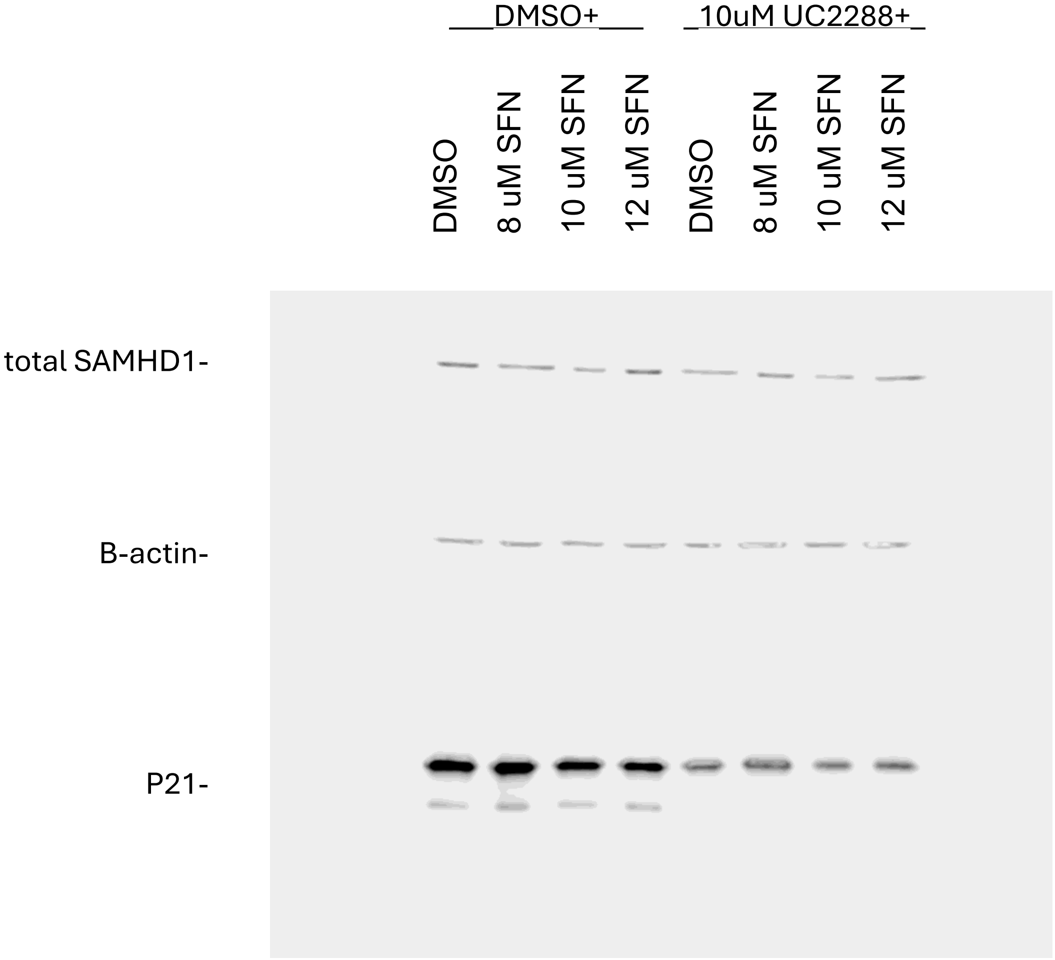

For all representative western blots, equal loading was confirmed using corresponding housekeeping controls (e.g., beta-actin), with all target proteins for a given series of blots resolved from the exact same sample lysates.
