# Supplementary material for: p21 (CDKN1A) Is the Major Driver of Sulforaphane-Mediated Reduction in SAMHD1 T592 Phosphorylation in Macrophages
Source: Biomolecules. 2026 Aug 20;16(8):1213. doi: 10.3390/biom16081213 (PMC13511314; doi:10.3390/biom16081213)
Supplement: Supplementary file 1 [file biomolecules-16-01213-s001.zip › Supplementary File S3 Statistical Analyses/Statistical Analyses.pdf]

Table format: **Column**		Group A	Group B	Group C	Group D
		DMSO	10 uM SFN	15 uM SFN	20 uM SFN
1	hMDM d1	0.9195221	0.036778	0.026482	0.061230
2	hMDM d2	0.6688220	0.053716	0.090916	0.072639
3	hMDM d3	0.4497310	0.048238	0.029192	0.000622

Table format: **Column**		Group A	Group B	Group C	Group D
		DMSO	10 uM SFN	15 uM SFN	20 uM SFN
1	hMDM d1	0.2850659	1.032369	1.290213	0.913505
2	hMDM d2	0.1679730	0.729732	1.109989	1.081734
3	hMDM d3	0.0998420	0.539219	0.494332	0.480915

	Group A	Group B	Group C	Group D
	DMSO	10 uM SFN	15 uM SFN	20 μM SFN
1	0.859794	0.276602	0.250281	0.092446
2	0.515434	0.008006	0.051152	0.033094
3	0.656202	0.073090	0.257644	0.053293

	Group A	Group B	Group C	Group D
	DMSO	10 uM SFN	15 uM SFN	20 µM SFN
1	0.961617	0.844337	0.931876	1.152799
2	1.028033	1.013229	0.810190	1.042840
3	0.866050	1.065358	0.994893	0.954393

Table format: **Column**		Group A	Group B	Group C
		DMSO	10 uM SFN	100 U/mL IFNy
1	hMDM d1	10.08864	1.620327	0.784972129
2	hMDM d2	20.31630	5.250392	0.307692000
3	hMDM d3	11.64525	0.997440	0.445424814

Table format: **Column**		Group A	Group B	Group C
		DMSO	10 uM SFN	100 U/mL IFNy
1	hMDM d1	0.326192	0.919609	0.929934433
2	hMDM d2	0.480610	1.346305	1.069844000
3	hMDM d3	0.474656	0.923583	0.813959279

	Group A	Group B	Group C
	DMSO	10 uM SFN	100 U/mL IFNy
1	10.926860	2.862598	0.563892
2	5.863484	2.679854	0.532041
3	5.779813	0.862654	0.137655

	Group A	Group B	Group C
	DMSO	10 uM SFN	100 U/mL IFNy
1	0.247186	0.233747	0.791612
2	0.093479	0.104571	0.516025
3	0.180426	0.167537	1.190924

	Group A	Group B	Group C	Group D
	DMSO	5uM UC2288	10uM UC2288	15uM UC2288
1	1.778655	1.671296	0.718579	0.264460
2	1.941634	1.333037	0.950494	0.175322
3	1.803566	1.230116	0.966438	0.356772

	Group A	Group B	Group C	Group D
	DMSO	5uM UC2288	10uM UC2288	15uM UC2288
1	1.896897	0.111865	0.106666	0.091581
2	1.352213	0.130591	0.096158	0.096748
3	1.356152	0.128441	0.094707	0.103425

Table format: **Column**		Group A	Group B	Group C	Group D
		DMSO	5uM UC2288	10uM UC2288	15uM UC2288
1	hMDM d1	1.2564250	0.213570	0.162735	0.263325
2	hMDM d2	0.7928130	0.011770	0.044234	0.102928
3	hMDM d3	0.9085110	0.155102	0.129726	0.125283

Table format: **Column**	Group A	Group B	Group C	Group D
	DMSO	5uM UC2288	10uM UC2288	15uM UC2288
1 hMDM d1	0.9038380	0.509825	0.562971	0.554382
2 hMDM d2	0.8111800	0.425156	0.611548	0.572436
3 hMDM d3	0.9075190	0.444335	0.419075	0.602639

	Group A	Group B	Group C	Group D
	DMSO	5uM UC2288	10uM UC2288	15uM UC2288
1	1.305124	0.397522	0.374919	0.308495
2	1.215041	0.373586	0.352304	0.360802
3	1.060964	0.372495	0.372128	0.345672

	Group A	Group B	Group C	Group D
	DMSO	5uM UC2288	10uM UC2288	15uM UC2288
1	0.964741	0.456166	0.533841	0.374901
2	0.970173	0.458150	0.389319	0.476847
3	0.827653	0.386923	0.406560	0.323337

Table format: **Grouped**		Group A DMSO+			Group B 10uM UC2288+		
		A:1	A:2	A:3	B:1	B:2	B:3
1	DMSO	0.906825	0.966187	0.947838	0.053900	0.323873	0.060230
2	8 uM SFN	0.242412	0.476087	0.251546	0.042169	0.304814	0.049944
3	10 uM SFN	0.236935	0.399207	0.251719	0.053069	0.334506	0.058640
4	12 uM SFN	0.110739	0.347406	0.118053	0.113510	0.329900	0.089072

Table format: **Grouped**		Group A DMSO+			Group B 10uM UC2288+		
		A:1	A:2	A:3	B:1	B:2	B:3
1	DMSO	0.347181	0.347019	0.281410	0.152418	0.285649	0.155616
2	8 uM SFN	0.986575	0.657748	1.004441	0.197905	0.311890	0.197890
3	10 uM SFN	1.211103	0.859848	1.165855	0.215305	0.349269	0.199549
4	12 uM SFN	0.974143	0.914858	0.938770	0.564686	0.432330	0.275043

Table format: **Grouped**		Group A DMSO+			Group B 10uM UC2288+		
		A:1	A:2	A:3	B:1	B:2	B:3
1	DMSO	1.070055	1.017519	1.051623	0.146414	0.172819	0.260790
2	8 uM SFN	0.266269	0.204353	0.321036	0.227037	0.173031	0.210509
3	10 uM SFN	0.271619	0.280190	0.252169	0.243722	0.183257	0.211047
4	12 uM SFN	0.193297	0.190482	0.263803	0.247596	0.169173	0.258388

Table format: **Grouped**	Group A DMSO+			Group B 10uM UC2288+		
	A:1	A:2	A:3	B:1	B:2	B:3
1 DMSO	0.938100	0.848095	0.882155	0.355919	0.330959	0.430349
2 8 uM SFN	1.044398	0.855938	0.861675	0.386299	0.403878	0.303149
3 10 uM SFN	0.879842	1.059227	0.871227	0.281778	0.366996	0.348829
4 12 uM SFN	0.757760	1.045795	0.871227	0.241101	0.356148	0.399590

Table format: **Grouped**		Group A nt siRNA (pooled)				Group B p21 (pooled)			
		A:1	A:2	A:3	A:4	B:1	B:2	B:3	B:4
1	DMSO	0.743976	0.7730940	0.89213300	0.890461000	1.804800	1.246515	1.597855	0.928762
2	5 uM SFN	0.223036	0.1796030	0.42537900	0.370726000	1.152819	2.299729	1.576340	2.065833
3	8 uM SFN	0.410800	0.3590630	0.18020860	0.133025626	1.011749	1.626622	1.261764	1.975965

Table format: **Grouped**	Group A nt siRNA (pooled)				Group B p21 (pooled)			
	A:1	A:2	A:3	A:4	B:1	B:2	B:3	B:4
1 DMSO	1.3763660	2.0402430	1.73616500	0.991217000	0.441304000	0.483694	0.597807	0.402225
2 5 uM SFN	1.5732480	2.5171010	2.00241000	1.028311000	0.596275000	0.541238	0.772821	0.642664
3 8 uM SFN	2.1335624	2.3234500	2.10893650	1.075431574	0.665147173	0.712787	0.851674	0.841100

Table format: **Grouped**	Group A nt gRNA			Group B p21 gRNA 1			Group C p21 gRNA 2		
	A:1	A:2	A:3	B:1	B:2	B:3	C:1	C:2	C:3
1 DMSO	0.515705	0.532249	0.516480	1.274846	0.739673	0.984675	1.331158	0.871312	0.776543
2 10 uM SFN	0.108063	0.124049	0.155325	1.223486	0.726567	1.034090	1.326898	0.855349	0.871427
3 15 uM SFN	0.121475	0.108028	0.122292	1.213350	0.747277	0.935908	0.830792	0.723777	0.948307

Table format: **Grouped**		Group A nt gRNA			Group B p21 gRNA 1			Group C p21 gRNA 2		
		A:1	A:2	A:3	B:1	B:2	B:3	C:1	C:2	C:3
1	DMSO	1292310	1420933	1154209	2049187	2385003	1810241	977959	1139574	844301
2	10 uM SFN	743056	838271	692159	2212802	2612132	1897788	826101	989635	713146
3	15 uM SFN	443218	461883	358839	2041460	2226169	1879517	883287	1088696	770581

Table format: **Grouped**		Group A DMSO			Group B 10uM SFN		
		A:1	A:2	A:3	B:1	B:2	B:3
1	Cytoplasm	28769.39	26009.02	27325.65	34092.100	36470.090	35828.550
2	Nucleus	14650.70	15284.82	16009.51	7846.577	8766.045	9005.752
3	Total Cell	43420.09	41293.84	43335.16	41938.670	45236.130	44834.300

Table format: **Grouped**		Group A DMSO			Group B 10uM SFN		
		A:1	A:2	A:3	B:1	B:2	B:3
1	Cytoplasm	9648.681	12125.980	21815.82	35276.920	49535.620	81617.920
2	Nucleus	5365.004	8659.163	15849.26	5623.175	8466.237	16222.670
3	Total Cell	15013.690	20785.150	37665.08	40900.100	58001.860	97840.600

	Group A	Group B	Group C	Group D
	DMSO	5 uM SFN	10 uM SFN	15 uM SFN
1	0.209487	0.251555	0.422127	0.618180
2	0.197301	0.273421	0.428923	0.555392
3	0.255567	0.367988	0.480383	0.589378

	Group A	Group B	Group C	Group D
	DMSO	5 uM SFN	10 uM SFN	15 uM SFN
1	0.577234	0.720492	0.771594	0.889494
2	0.923229	0.967562	0.903795	1.038596
3	0.899315	0.943630	1.080294	0.929341

RM one-way ANOVA<br>ANOVA results							
1	Table Analyze	hMDM SFN P					
2	Distribution as	Normal (Gaus					
3							
4	**Repeated me**						
5	Assume sphe	Yes					
6	F	22.15					
7	P value	0.0012					
8	P value sum	**					
9	Statistically si	Yes					
10	R squared	0.9172					
11							
12	**Was the mat**						
13	F	1.290					
14	P value	0.3419					
15	P value sum	ns					
16	Is there signi	No					
17	R squared	0.03440					
18							
19	**ANOVA table**	**SS**	**DF**	**MS**	**F (DFn, DFd)**	**P value**	
20	Treatment (b	0.9008	3	0.3003	F (3, 6) = 22.1	P=0.0012	
21	Individual (be	0.03498	2	0.01749	F (2, 6) = 1.29	P=0.3419	
22	Residual (ra	0.08134	6	0.01356			
23	Total	1.017	11				
24							
25	**Data summa**						
26	Number of tr	4					
27	Number of su	3					
28	Number of m	0					

RM one-way ANOVA Multiple comparisons								
1	Number of families	1						
2	Number of comparisons per family	3						
3	Alpha	0.05						
4								
5	**Dunnett's multiple comparisons test**	**Mean diff.**	**95.00% CI of diff.**	**Below threshold?**	**Summary**	**Adjusted P Value**	**A-?**	
6	DMSO vs. 10 uM SFN	0.6331	0.3385 to 0.9278	Yes	**	0.0014	B	10 uM SFN
7	DMSO vs. 15 uM SFN	0.6305	0.3359 to 0.9251	Yes	**	0.0014	C	15 uM SFN
8	DMSO vs. 20 uM SFN	0.6345	0.3399 to 0.9292	Yes	**	0.0014	D	20 uM SFN
9								
10	**Test details**	**Mean 1**	**Mean 2**	**Mean diff.**	**SE of diff.**	**n1**	**n2**	**q**
11	DMSO vs. 10 uM SFN	0.6794	0.04624	0.6331	0.09506	3	3	6.660
12	DMSO vs. 15 uM SFN	0.6794	0.04886	0.6305	0.09506	3	3	6.632
13	DMSO vs. 20 uM SFN	0.6794	0.04483	0.6345	0.09506	3	3	6.675

1	
2	
3	
4	
5	
6	
7	
8	
9	
10	**DF**
11	6
12	6
13	6

RM one-way ANOVA<br>ANOVA results							
1	Table Analyze	hMDM SFN p					
2	Distribution as	Normal (Gaus					
3							
4	**Repeated me**						
5	Assume sph	Yes					
6	F	11.73					
7	P value	0.0064					
8	P value sumi	**					
9	Statistically si	Yes					
10	R squared	0.8543					
11							
12	**Was the mat**						
13	F	8.247					
14	P value	0.0190					
15	P value sumi	*					
16	Is there signi	Yes					
17	R squared	0.2859					
18							
19	**ANOVA table**	**SS**	**DF**	**MS**	**F (DFn, DFd)**	**P value**	
20	Treatment (b	1.066	3	0.3555	F (3, 6) = 11.7	P=0.0064	
21	Individual (be	0.4999	2	0.2499	F (2, 6) = 8.24	P=0.0190	
22	Residual (rai	0.1818	6	0.03031			
23	Total	1.748	11				
24							
25	**Data summa**						
26	Number of tr	4					
27	Number of su	3					
28	Number of m	0					

RM one-way ANOVA
Multiple comparisons

1	Number of fa	1							
2	Number of co	3							
3	Alpha	0.05							
4									
5	**Dunnett's mu**	**Mean diff.**	**95.00% CI of**	**Below thresh**	**Summary**	**Adjusted P Va**	**A-?**		
6	DMSO vs. 1(	-0.5828	-1.023 to -0.1	Yes	*	0.0155	B	10 uM SFN	
7	DMSO vs. 1!	-0.7806	-1.221 to -0.3	Yes	**	0.0038	C	15 uM SFN	
8	DMSO vs. 2(	-0.6411	-1.082 to -0.2	Yes	**	0.0100	D	20 uM SFN	
9									
10	**Test details**	**Mean 1**	**Mean 2**	**Mean diff.**	**SE of diff.**	**n1**	**n2**	**q**	**DF**
11	DMSO vs. 1(	0.1843	0.7671	-0.5828	0.1421	3	3	4.100	6
12	DMSO vs. 1!	0.1843	0.9648	-0.7806	0.1421	3	3	5.491	6
13	DMSO vs. 2(	0.1843	0.8254	-0.6411	0.1421	3	3	4.510	6

1	Table Analyze	THP1 SFN PS				
2	Data sets ana	A-D				
3	Distribution as	Normal (Gaus				
4						
5	**ANOVA sumr**					
6	F	14.91				
7	P value	0.0012				
8	P value sumi	**				
9	Significant di	Yes				
10	R squared	0.8483				
11						
12	**Brown-Forsy**					
13	F (DFn, DFd	0.5596 (3, 8)				
14	P value	0.6564				
15	P value sumi	ns				
16	Are SDs sigr	No				
17						
18	**Bartlett's tes**					
19	Bartlett's stat					
20	P value					
21	P value sumi					
22	Are SDs sigr					
23						
24	**ANOVA table**	**SS**	**DF**	**MS**	**F (DFn, DFd)**	**P value**
25	Treatment (b	0.7182	3	0.2394	F (3, 8) = 14.9	P=0.0012
26	Residual (wi	0.1285	8	0.01606		
27	Total	0.8467	11			
28						
29	**Data summa**					
30	Number of tr	4				
31	Number of v	12				

1	Number of far	1							
2	Number of co	3							
3	Alpha	0.05							
4									
5	**Dunnett's mu**	**Mean diff.**	**95.00% CI of**	**Below thresh**	**Summary**	**Adjusted P Va**	**A-?**		
6	DMSO vs. 1(	0.5579	0.2599 to 0.8!	Yes	**	0.0017	B	10 uM SFN	
7	DMSO vs. 1!	0.4908	0.1928 to 0.7!	Yes	**	0.0038	C	15 uM SFN	
8	DMSO vs. 2(	0.6175	0.3196 to 0.9'	Yes	***	0.0009	D	20 µM SFN	
9									
10	**Test details**	**Mean 1**	**Mean 2**	**Mean diff.**	**SE of diff.**	**n1**	**n2**	**q**	**DF**
11	DMSO vs. 1(	0.6771	0.1192	0.5579	0.1035	3	3	5.392	8
12	DMSO vs. 1!	0.6771	0.1864	0.4908	0.1035	3	3	4.743	8
13	DMSO vs. 2(	0.6771	0.05961	0.6175	0.1035	3	3	5.968	8

1	Table Analyze	THP1 SFN p2				
2	Data sets ana	A-D				
3	Distribution as	Normal (Gaus				
4						
5	**ANOVA sumr**					
6	F	1.040				
7	P value	0.4257				
8	P value sumi	ns				
9	Significant di	No				
10	R squared	0.2806				
11						
12	**Brown-Forsy**					
13	F (DFn, DFd)	0.04808 (3, 8				
14	P value	0.9850				
15	P value sumi	ns				
16	Are SDs sigr	No				
17						
18	**Bartlett's tes**					
19	Bartlett's stat					
20	P value					
21	P value sumi					
22	Are SDs sigr					
23						
24	**ANOVA table**	**SS**	**DF**	**MS**	**F (DFn, DFd)**	**P value**
25	Treatment (b	0.03017	3	0.01006	F (3, 8) = 1.04	P=0.4257
26	Residual (wit	0.07735	8	0.009669		
27	Total	0.1075	11			
28						
29	**Data summa**					
30	Number of tr	4				
31	Number of v	12				

1	Number of far	1						
2	Number of co	3						
3	Alpha	0.05						
4								
5	**Dunnett's mu**	**Mean diff.**	**95.00% CI of**	**Below thresh**	**Summary**	**Adjusted P Va**	**A-?**		
6	DMSO vs. 1(	-0.02241	-0.2536 to 0.2	No	ns	0.9843	B	10 uM SFN	
7	DMSO vs. 1!	0.03958	-0.1916 to 0.2	No	ns	0.9256	C	15 uM SFN	
8	DMSO vs. 2(	-0.09811	-0.3293 to 0.1	No	ns	0.5088	D	20 µM SFN	
9									
10	**Test details**	**Mean 1**	**Mean 2**	**Mean diff.**	**SE of diff.**	**n1**	**n2**	**q**	**DF**
11	DMSO vs. 1(	0.9519	0.9743	-0.02241	0.08029	3	3	0.2791	8
12	DMSO vs. 1!	0.9519	0.9123	0.03958	0.08029	3	3	0.4930	8
13	DMSO vs. 2(	0.9519	1.050	-0.09811	0.08029	3	3	1.222	8

RM one-way ANOVA
ANOVA results

1	Table Analyze	hMDM IFN PS					
2	Distribution as	Normal (Gaus					
3							
4	**Repeated me**						
5	Assume sph	Yes					
6	F	19.01					
7	P value	0.0091					
8	P value sum	**					
9	Statistically si	Yes					
10	R squared	0.9048					
11							
12	**Was the mat**						
13	F	2.287					
14	P value	0.2176					
15	P value sum	ns					
16	Is there signi	No					
17	R squared	0.09818					
18							
19	**ANOVA table**	**SS**	**DF**	**MS**	**F (DFn, DFd)**	**P value**	
20	Treatment (b	316.6	2	158.3	F (2, 4) = 19.0	P=0.0091	
21	Individual (be	38.10	2	19.05	F (2, 4) = 2.28	P=0.2176	
22	Residual (ra	33.31	4	8.328			
23	Total	388.0	8				
24							
25	**Data summa**						
26	Number of tr	3					
27	Number of s	3					
28	Number of m	0					

RM one-way ANOVA
Multiple comparisons

1	Number of far	1							
2	Number of co	2							
3	Alpha	0.05							
4									
5	**Dunnett's mu**	**Mean diff.**	**95.00% CI of**	**Below thresh**	**Summary**	**Adjusted P Va**	**A-?**		
6	DMSO vs. 1(	11.39	3.594 to 19.1(	Yes	*	0.0144	B	10 uM SFN	
7	DMSO vs. 1(	13.50	5.704 to 21.3(	Yes	**	0.0079	C	100 U/mL IFN	
8									
9	**Test details**	**Mean 1**	**Mean 2**	**Mean diff.**	**SE of diff.**	**n1**	**n2**	**q**	**DF**
10	DMSO vs. 1(	14.02	2.623	11.39	2.356	3	3	4.836	4
11	DMSO vs. 1(	14.02	0.5127	13.50	2.356	3	3	5.731	4

**RM one-way ANOVA** ANOVA results							
1	Table Analyze	hMDM IFN p2					
2	Distribution as	Normal (Gaus					
3							
4	**Repeated me**						
5	Assume sphe	Yes					
6	F	23.32					
7	P value	0.0062					
8	P value sumi	**					
9	Statistically si	Yes					
10	R squared	0.9210					
11							
12	**Was the matc**						
13	F	3.765					
14	P value	0.1203					
15	P value sumi	ns					
16	Is there signi	No					
17	R squared	0.1295					
18							
19	**ANOVA table**	**SS**	**DF**	**MS**	**F (DFn, DFd)**	**P value**	
20	Treatment (b	0.6811	2	0.3405	F (2, 4) = 23.3	P=0.0062	
21	Individual (be	0.1100	2	0.05499	F (2, 4) = 3.76	P=0.1203	
22	Residual (rai	0.05842	4	0.01461			
23	Total	0.8495	8				
24							
25	**Data summa**						
26	Number of tr	3					
27	Number of su	3					
28	Number of m	0					

RM one-way ANOVA
Multiple comparisons

1	Number of far	1							
2	Number of co	2							
3	Alpha	0.05							
4									
5	**Dunnett's mu**	**Mean diff.**	**95.00% CI of**	**Below thresh**	**Summary**	**Adjusted P Va**	**A-?**		
6	DMSO vs. 1(	-0.6360	-0.9627 to -0.	Yes	**	0.0051	B	10 uM SFN	
7	DMSO vs. 1(	-0.5108	-0.8374 to -0.	Yes	*	0.0114	C	100 U/mL IFN	
8									
9	**Test details**	**Mean 1**	**Mean 2**	**Mean diff.**	**SE of diff.**	**n1**	**n2**	**q**	**DF**
10	DMSO vs. 1(	0.4272	1.063	-0.6360	0.09867	3	3	6.446	4
11	DMSO vs. 1(	0.4272	0.9379	-0.5108	0.09867	3	3	5.176	4

1	Table Analyze	THP1 IFN PS/				
2	Data sets ana	A-C				
3	Distribution as	Normal (Gaus				
4						
5	**ANOVA sumr**					
6	F	12.43				
7	P value	0.0074				
8	P value sumi	**				
9	Significant di	Yes				
10	R squared	0.8055				
11						
12	**Brown-Forsy**					
13	F (DFn, DFd)	0.6110 (2, 6)				
14	P value	0.5734				
15	P value sumi	ns				
16	Are SDs sigr	No				
17						
18	**Bartlett's tes**					
19	Bartlett's stat					
20	P value					
21	P value sumi					
22	Are SDs sigr					
23						
24	**ANOVA table**	**SS**	**DF**	**MS**	**F (DFn, DFd)**	**P value**
25	Treatment (b	82.59	2	41.29	F (2, 6) = 12.4	P=0.0074
26	Residual (wit	19.94	6	3.323		
27	Total	102.5	8			
28						
29	**Data summa**					
30	Number of tr	3				
31	Number of v	9				

1	Number of far	1							
2	Number of co	2							
3	Alpha	0.05							
4									
5	**Dunnett's mu**	**Mean diff.**	**95.00% CI of**	**Below thresh**	**Summary**	**Adjusted P Va**	**A-?**		
6	DMSO vs. 1(	5.388	1.127 to 9.65(	Yes	*	0.0196	B	10 uM SFN	
7	DMSO vs. 1(	7.112	2.851 to 11.37	Yes	**	0.0055	C	100 U/mL IFN	
8									
9	**Test details**	**Mean 1**	**Mean 2**	**Mean diff.**	**SE of diff.**	**n1**	**n2**	**q**	**DF**
10	DMSO vs. 1(	7.523	2.135	5.388	1.488	3	3	3.620	6
11	DMSO vs. 1(	7.523	0.4112	7.112	1.488	3	3	4.779	6

1	Table Analyze	THP1 IFN p2'				
2	Data sets ana	A-C				
3	Distribution as	Normal (Gaus				
4						
5	**ANOVA sumr**					
6	F	10.49				
7	P value	0.0110				
8	P value sum	*				
9	Significant di	Yes				
10	R squared	0.7776				
11						
12	**Brown-Forsy**					
13	F (DFn, DFd	2.100 (2, 6)				
14	P value	0.2036				
15	P value sum	ns				
16	Are SDs sign	No				
17						
18	**Bartlett's tes**					
19	Bartlett's stat					
20	P value					
21	P value sum					
22	Are SDs sign					
23						
24	**ANOVA table**	**SS**	**DF**	**MS**	**F (DFn, DFd)**	**P value**
25	Treatment (b	0.8757	2	0.4379	F (2, 6) = 10.4	P=0.0110
26	Residual (wit	0.2505	6	0.04175		
27	Total	1.126	8			
28						
29	**Data summa**					
30	Number of tr	3				
31	Number of va	9				

1	Number of far	1							
2	Number of co	2							
3	Alpha	0.05							
4									
5	**Dunnett's mu**	**Mean diff.**	**95.00% CI of**	**Below thresh**	**Summary**	**Adjusted P Va**	**A-?**		
6	DMSO vs. 1(	0.005079	-0.4726 to 0.4	No	ns	0.9993	B	10 uM SFN	
7	DMSO vs. 1(	-0.6592	-1.137 to -0.1	Yes	*	0.0134	C	100 U/mL IFN	
8									
9	**Test details**	**Mean 1**	**Mean 2**	**Mean diff.**	**SE of diff.**	**n1**	**n2**	**q**	**DF**
10	DMSO vs. 1(	0.1737	0.1686	0.005079	0.1668	3	3	0.03044	6
11	DMSO vs. 1(	0.1737	0.8329	-0.6592	0.1668	3	3	3.951	6

1	Table Analyze	786O UC228				
2	Data sets ana	A-D				
3	Distribution as	Normal (Gaus				
4						
5	**ANOVA sumr**					
6	F	62.94				
7	P value	<0.0001				
8	P value sumi	****				
9	Significant di	Yes				
10	R squared	0.9594				
11						
12	**Brown-Forsy**					
13	F (DFn, DFd	0.3922 (3, 8)				
14	P value	0.7621				
15	P value sumi	ns				
16	Are SDs sigr	No				
17						
18	**Bartlett's tes**					
19	Bartlett's stat					
20	P value					
21	P value sumi					
22	Are SDs sigr					
23						
24	**ANOVA table**	**SS**	**DF**	**MS**	**F (DFn, DFd)**	**P value**
25	Treatment (b	4.176	3	1.392	F (3, 8) = 62.9	P<0.0001
26	Residual (wit	0.1769	8	0.02212		
27	Total	4.353	11			
28						
29	**Data summa**					
30	Number of tr	4				
31	Number of v	12				

1	Number of far	1						
2	Number of co	3						
3	Alpha	0.05						
4								
5	**Dunnett's mu**	**Mean diff.**	**95.00% CI of**	**Below thresh**	**Summary**	**Adjusted P Va**	**A-?**		
6	DMSO vs. 5u	0.4298	0.08014 to 0.7	Yes	*	0.0191	B	5uM UC2288	
7	DMSO vs. 10	0.9628	0.6131 to 1.3	Yes	***	0.0001	C	10uM UC228	
8	DMSO vs. 15	1.576	1.226 to 1.92	Yes	****	<0.0001	D	15uM UC228	
9									
10	**Test details**	**Mean 1**	**Mean 2**	**Mean diff.**	**SE of diff.**	**n1**	**n2**	**q**	**DF**
11	DMSO vs. 5u	1.841	1.411	0.4298	0.1214	3	3	3.540	8
12	DMSO vs. 10	1.841	0.8785	0.9628	0.1214	3	3	7.929	8
13	DMSO vs. 15	1.841	0.2655	1.576	0.1214	3	3	12.98	8

1	Table Analyze	786O UC228				
2	Data sets ana	A-D				
3	Distribution as	Normal (Gaus				
4						
5	**ANOVA sumr**					
6	F	62.24				
7	P value	<0.0001				
8	P value sum	****				
9	Significant di	Yes				
10	R squared	0.9589				
11						
12	**Brown-Forsy**					
13	F (DFn, DFd	0.9684 (3, 8)				
14	P value	0.4537				
15	P value sum	ns				
16	Are SDs sign	No				
17						
18	**Bartlett's tes**					
19	Bartlett's stat					
20	P value					
21	P value sum					
22	Are SDs sign					
23						
24	**ANOVA table**	**SS**	**DF**	**MS**	**F (DFn, DFd)**	**P value**
25	Treatment (b	4.592	3	1.531	F (3, 8) = 62.2	P<0.0001
26	Residual (wit	0.1967	8	0.02459		
27	Total	4.789	11			
28						
29	**Data summa**					
30	Number of tr	4				
31	Number of v	12				

1	Number of far	1							
2	Number of co	3							
3	Alpha	0.05							
4									
5	**Dunnett's mu**	**Mean diff.**	**95.00% CI of**	**Below thresh**	**Summary**	**Adjusted P Va**	**A-?**		
6	DMSO vs. 5u	1.411	1.043 to 1.78	Yes	****	<0.0001	B	5uM UC2288	
7	DMSO vs. 10	1.436	1.067 to 1.80	Yes	****	<0.0001	C	10uM UC228	
8	DMSO vs. 15	1.438	1.069 to 1.80	Yes	****	<0.0001	D	15uM UC228	
9									
10	**Test details**	**Mean 1**	**Mean 2**	**Mean diff.**	**SE of diff.**	**n1**	**n2**	**q**	**DF**
11	DMSO vs. 5u	1.535	0.1236	1.411	0.1280	3	3	11.02	8
12	DMSO vs. 10	1.535	0.09918	1.436	0.1280	3	3	11.21	8
13	DMSO vs. 15	1.535	0.09725	1.438	0.1280	3	3	11.23	8

RM one-way ANOVA<br>ANOVA results							
1	Table Analyze	hMDM UC228					
2	Distribution as	Normal (Gaus					
3							
4	**Repeated me**						
5	Assume sph	Yes					
6	F	69.21					
7	P value	<0.0001					
8	P value sum	****					
9	Statistically si	Yes					
10	R squared	0.9719					
11							
12	**Was the mat**						
13	F	7.190					
14	P value	0.0255					
15	P value sum	*					
16	Is there signi	Yes					
17	R squared	0.06307					
18							
19	**ANOVA table**	**SS**	**DF**	**MS**	**F (DFn, DFd)**	**P value**	
20	Treatment (b	1.636	3	0.5454	F (3, 6) = 69.2	P<0.0001	
21	Individual (be	0.1133	2	0.05666	F (2, 6) = 7.19	P=0.0255	
22	Residual (ra	0.04728	6	0.007880			
23	Total	1.797	11				
24							
25	**Data summa**						
26	Number of tr	4					
27	Number of s	3					
28	Number of m	0					

RM one-way ANOVA
Multiple comparisons

1	Number of far	1							
2	Number of co	3							
3	Alpha	0.05							
4									
5	**Dunnett's mu**	**Mean diff.**	**95.00% CI of**	**Below thresh**	**Summary**	**Adjusted P Va**	**A-?**		
6	DMSO vs. 5u	0.8591	0.6345 to 1.08	Yes	****	<0.0001	B	5uM UC2288	
7	DMSO vs. 10	0.8737	0.6490 to 1.09	Yes	****	<0.0001	C	10uM UC228	
8	DMSO vs. 15	0.8221	0.5974 to 1.04	Yes	****	<0.0001	D	15uM UC228	
9									
10	**Test details**	**Mean 1**	**Mean 2**	**Mean diff.**	**SE of diff.**	**n1**	**n2**	**q**	**DF**
11	DMSO vs. 5u	0.9859	0.1268	0.8591	0.07248	3	3	11.85	6
12	DMSO vs. 10	0.9859	0.1122	0.8737	0.07248	3	3	12.05	6
13	DMSO vs. 15	0.9859	0.1638	0.8221	0.07248	3	3	11.34	6

RM one-way ANOVA
ANOVA results

1	Table Analyze	hMDM UC228					
2	Distribution as	Normal (Gaus					
3							
4	**Repeated me**						
5	Assume sphe	Yes					
6	F	21.47					
7	P value	0.0013					
8	P value sumi	**					
9	Statistically si	Yes					
10	R squared	0.9148					
11							
12	**Was the mat**						
13	F	0.3520					
14	P value	0.7169					
15	P value sumi	ns					
16	Is there signi	No					
17	R squared	0.009900					
18							
19	**ANOVA table**	**SS**	**DF**	**MS**	**F (DFn, DFd)**	**P value**	
20	Treatment (b	0.2991	3	0.09969	F (3, 6) = 21.4	P=0.0013	
21	Individual (be	0.003269	2	0.001635	F (2, 6) = 0.35	P=0.7169	
22	Residual (rai	0.02786	6	0.004643			
23	Total	0.3302	11				
24							
25	**Data summa**						
26	Number of tr	4					
27	Number of su	3					
28	Number of m	0					

RM one-way ANOVA
Multiple comparisons

1	Number of far	1							
2	Number of co	3							
3	Alpha	0.05							
4									
5	**Dunnett's mu**	**Mean diff.**	**95.00% CI of**	**Below thresh**	**Summary**	**Adjusted P Va**	**A-?**		
6	DMSO vs. 5u	0.4144	0.2420 to 0.58	Yes	***	0.0008	B	5uM UC2288	
7	DMSO vs. 10	0.3430	0.1705 to 0.51	Yes	**	0.0021	C	10uM UC228	
8	DMSO vs. 15	0.2977	0.1253 to 0.47	Yes	**	0.0043	D	15uM UC228	
9									
10	**Test details**	**Mean 1**	**Mean 2**	**Mean diff.**	**SE of diff.**	**n1**	**n2**	**q**	**DF**
11	DMSO vs. 5u	0.8742	0.4598	0.4144	0.05564	3	3	7.448	6
12	DMSO vs. 10	0.8742	0.5312	0.3430	0.05564	3	3	6.165	6
13	DMSO vs. 15	0.8742	0.5765	0.2977	0.05564	3	3	5.351	6

1	Table Analyze	THP1 UC228				
2	Data sets ana	A-D				
3	Distribution as	Normal (Gaus				
4						
5	**ANOVA sumr**					
6	F	127.4				
7	P value	<0.0001				
8	P value sum	****				
9	Significant di	Yes				
10	R squared	0.9795				
11						
12	**Brown-Forsy**					
13	F (DFn, DFd	2.268 (3, 8)				
14	P value	0.1576				
15	P value sum	ns				
16	Are SDs sign	No				
17						
18	**Bartlett's tes**					
19	Bartlett's stat					
20	P value					
21	P value sum					
22	Are SDs sign					
23						
24	**ANOVA table**	**SS**	**DF**	**MS**	**F (DFn, DFd)**	**P value**
25	Treatment (b	1.559	3	0.5198	F (3, 8) = 127	P<0.0001
26	Residual (wit	0.03264	8	0.004080		
27	Total	1.592	11			
28						
29	**Data summa**					
30	Number of tr	4				
31	Number of va	12				

1	Number of far	1						
2	Number of co	3						
3	Alpha	0.05						
4								
5	**Dunnett's mu**	**Mean diff.**	**95.00% CI of**	**Below thresh**	**Summary**	**Adjusted P Va**	**A-?**	
6	DMSO vs. 5u	0.8125	0.6623 to 0.96	Yes	****	<0.0001	B	5uM UC2288
7	DMSO vs. 10	0.8273	0.6771 to 0.97	Yes	****	<0.0001	C	10uM UC228
8	DMSO vs. 15	0.8554	0.7052 to 1.00	Yes	****	<0.0001	D	15uM UC228
9								
10	**Test details**	**Mean 1**	**Mean 2**	**Mean diff.**	**SE of diff.**	**n1**	**n2**	**q**	**DF**
11	DMSO vs. 5u	1.194	0.3812	0.8125	0.05216	3	3	15.58	8
12	DMSO vs. 10	1.194	0.3665	0.8273	0.05216	3	3	15.86	8
13	DMSO vs. 15	1.194	0.3383	0.8554	0.05216	3	3	16.40	8

1	Table Analyze	THP1 UC228				
2	Data sets ana	A-D				
3	Distribution as	Normal (Gaus				
4						
5	**ANOVA sumr**					
6	F	127.4				
7	P value	<0.0001				
8	P value sumi	****				
9	Significant di	Yes				
10	R squared	0.9795				
11						
12	**Brown-Forsy**					
13	F (DFn, DFd	2.268 (3, 8)				
14	P value	0.1576				
15	P value sumi	ns				
16	Are SDs sign	No				
17						
18	**Bartlett's tes**					
19	Bartlett's stat					
20	P value					
21	P value sumi					
22	Are SDs sign					
23						
24	**ANOVA table**	**SS**	**DF**	**MS**	**F (DFn, DFd)**	**P value**
25	Treatment (b	1.559	3	0.5198	F (3, 8) = 127	P<0.0001
26	Residual (wit	0.03264	8	0.004080		
27	Total	1.592	11			
28						
29	**Data summa**					
30	Number of tr	4				
31	Number of v	12				

1	Number of far	1							
2	Number of co	3							
3	Alpha	0.05							
4									
5	**Dunnett's mu**	**Mean diff.**	**95.00% CI of**	**Below thresh**	**Summary**	**Adjusted P Va**	**A-?**		
6	DMSO vs. 5u	0.8125	0.6623 to 0.96	Yes	****	<0.0001	B	5uM UC2288	
7	DMSO vs. 10	0.8273	0.6771 to 0.97	Yes	****	<0.0001	C	10uM UC228	
8	DMSO vs. 15	0.8554	0.7052 to 1.00	Yes	****	<0.0001	D	15uM UC228	
9									
10	**Test details**	**Mean 1**	**Mean 2**	**Mean diff.**	**SE of diff.**	**n1**	**n2**	**q**	**DF**
11	DMSO vs. 5u	1.194	0.3812	0.8125	0.05216	3	3	15.58	8
12	DMSO vs. 10	1.194	0.3665	0.8273	0.05216	3	3	15.86	8
13	DMSO vs. 15	1.194	0.3383	0.8554	0.05216	3	3	16.40	8

2way ANOVA<br>ANOVA results									
1	Table Analyzed	hMDM SFN v UC2288 PSAM/SA							
2									
3	**Two-way RM ANOVA**	Matching: Across row							
4	Assume sphericity?	Yes							
5	Alpha	0.05							
6									
7	**Source of Variation**	**% of total variation**	**P value**	**P value summary**	**Significant?**				
8	Row Factor x Column Factor	29.97	<0.0001	****	Yes				
9	Row Factor	27.79	0.0245	*	Yes				
10	Column Factor	27.36	<0.0001	****	Yes				
11	Subject	13.58	0.0016	**	Yes				
12									
13	**ANOVA table**	**SS**	**DF**	**MS**	**F (DFn, DFd)**	**P value**			
14	Row Factor x Column Factor	0.5405	3	0.1802	F (3, 8) = 61.85	P<0.0001			
15	Row Factor	0.5011	3	0.1670	F (3, 8) = 5.455	P=0.0245			
16	Column Factor	0.4934	1	0.4934	F (1, 8) = 169.4	P<0.0001			
17	Subject	0.2450	8	0.03062	F (8, 8) = 10.51	P=0.0016			
18	Residual	0.02330	8	0.002913					
19									
20	**Difference between column means**								
21	Mean of DMSO+	0.4379							
22	Mean of 10uM UC2288+	0.1511							
23	Difference between means	0.2868							
24	SE of difference	0.02203							
25	95% CI of difference	0.2360 to 0.3376							
26									
27	**Data summary**								
28	Number of columns (Column Factor)	2							
29	Number of rows (Row Factor)	4							
30	Number of subjects (Subject)	12							
31	Number of missing values	0							

	2way ANOVA<br>Multiple comparisons					
1	Within each column, compare rows (simple effects within columns)					
2						
3	Number of families	2				
4	Number of comparisons per family	3				
5	Alpha	0.05				
6						
7	**Dunnett's multiple comparisons test**	**Mean diff.**	**95.00% CI of diff.**	**Below threshold?**	**Summary**	**Adjusted P Value**
8						
9	DMSO+					
10	DMSO vs. 8 uM SFN	0.6169	0.3428 to 0.8910	Yes	****	<0.0001
11	DMSO vs. 10 uM SFN	0.6443	0.3702 to 0.9184	Yes	****	<0.0001
12	DMSO vs. 12 uM SFN	0.7482	0.4741 to 1.022	Yes	****	<0.0001
13						
14	10uM UC2288+					
15	DMSO vs. 8 uM SFN	0.01369	-0.2604 to 0.2878	No	ns	0.9984
16	DMSO vs. 10 uM SFN	-0.002737	-0.2768 to 0.2714	No	ns	>0.9999
17	DMSO vs. 12 uM SFN	-0.03149	-0.3056 to 0.2426	No	ns	0.9816
18						
19						
20	**Test details**	**Mean 1**	**Mean 2**	**Mean diff.**	**SE of diff.**	**N1**
21						
22	DMSO+					
23	DMSO vs. 8 uM SFN	0.9403	0.3233	0.6169	0.1057	3
24	DMSO vs. 10 uM SFN	0.9403	0.2960	0.6443	0.1057	3
25	DMSO vs. 12 uM SFN	0.9403	0.1921	0.7482	0.1057	3
26						
27	10uM UC2288+					
28	DMSO vs. 8 uM SFN	0.1460	0.1323	0.01369	0.1057	3
29	DMSO vs. 10 uM SFN	0.1460	0.1487	-0.002737	0.1057	3
30	DMSO vs. 12 uM SFN	0.1460	0.1775	-0.03149	0.1057	3

1			
2			
3			
4			
5			
6			
7			
8			
9			
10			
11			
12			
13			
14			
15			
16			
17			
18			
19			
20	**N2**	**q**	**DF**
21			
22			
23	3	5.835	16.00
24	3	6.094	16.00
25	3	7.077	16.00
26			
27			
28	3	0.1295	16.00
29	3	0.02589	16.00
30	3	0.2979	16.00

2way ANOVA
ANOVA results

1	Table Analyzed	hMDM SFN v UC2288 PSAM/SA							
2									
3	**Two-way RM ANOVA**	Matching: Across row							
4	Assume sphericity?	Yes							
5	Alpha	0.05							
6									
7	**Source of Variation**	**% of total variation**	**P value**	**P value summary**	**Significant?**				
8	Row Factor x Column Factor	29.97	<0.0001	****	Yes				
9	Row Factor	27.79	0.0245	*	Yes				
10	Column Factor	27.36	<0.0001	****	Yes				
11	Subject	13.58	0.0016	**	Yes				
12									
13	**ANOVA table**	**SS**	**DF**	**MS**	**F (DFn, DFd)**	**P value**			
14	Row Factor x Column Factor	0.5405	3	0.1802	F (3, 8) = 61.85	P<0.0001			
15	Row Factor	0.5011	3	0.1670	F (3, 8) = 5.455	P=0.0245			
16	Column Factor	0.4934	1	0.4934	F (1, 8) = 169.4	P<0.0001			
17	Subject	0.2450	8	0.03062	F (8, 8) = 10.51	P=0.0016			
18	Residual	0.02330	8	0.002913					
19									
20	**Difference between column means**								
21	Mean of DMSO+	0.4379							
22	Mean of 10uM UC2288+	0.1511							
23	Difference between means	0.2868							
24	SE of difference	0.02203							
25	95% CI of difference	0.2360 to 0.3376							
26									
27	**Data summary**								
28	Number of columns (Column Factor)	2							
29	Number of rows (Row Factor)	4							
30	Number of subjects (Subject)	12							
31	Number of missing values	0							

	2way ANOVA<br>Multiple comparisons					
1	Within each column, compare rows (simple effects within columns)					
2						
3	Number of families	2				
4	Number of comparisons per family	3				
5	Alpha	0.05				
6						
7	**Šídák's multiple comparisons test**	**Mean diff.**	**95.00% CI of diff.**	**Below threshold?**	**Summary**	**Adjusted P Value**
8						
9	DMSO+					
10	DMSO vs. 8 uM SFN	0.6169	0.3352 to 0.8987	Yes	****	<0.0001
11	DMSO vs. 10 uM SFN	0.6443	0.3626 to 0.9261	Yes	****	<0.0001
12	DMSO vs. 12 uM SFN	0.7482	0.4665 to 1.030	Yes	****	<0.0001
13						
14	10uM UC2288+					
15	DMSO vs. 8 uM SFN	0.01369	-0.2680 to 0.2954	No	ns	0.9990
16	DMSO vs. 10 uM SFN	-0.002737	-0.2845 to 0.2790	No	ns	>0.9999
17	DMSO vs. 12 uM SFN	-0.03149	-0.3132 to 0.2502	No	ns	0.9878
18						
19						
20	**Test details**	**Mean 1**	**Mean 2**	**Mean diff.**	**SE of diff.**	**N1**
21						
22	DMSO+					
23	DMSO vs. 8 uM SFN	0.9403	0.3233	0.6169	0.1057	3
24	DMSO vs. 10 uM SFN	0.9403	0.2960	0.6443	0.1057	3
25	DMSO vs. 12 uM SFN	0.9403	0.1921	0.7482	0.1057	3
26						
27	10uM UC2288+					
28	DMSO vs. 8 uM SFN	0.1460	0.1323	0.01369	0.1057	3
29	DMSO vs. 10 uM SFN	0.1460	0.1487	-0.002737	0.1057	3
30	DMSO vs. 12 uM SFN	0.1460	0.1775	-0.03149	0.1057	3

1			
2			
3			
4			
5			
6			
7			
8			
9			
10			
11			
12			
13			
14			
15			
16			
17			
18			
19			
20	**N2**	**t**	**DF**
21			
22			
23	3	5.835	16.00
24	3	6.094	16.00
25	3	7.077	16.00
26			
27			
28	3	0.1295	16.00
29	3	0.02589	16.00
30	3	0.2979	16.00

2way ANOVA
ANOVA results

1	Table Analyzed	hMDM SFN v UC2288 PSAM/SAI				
2						
3	**Two-way RM ANOVA**	Matching: Across row				
4	Assume sphericity?	Yes				
5	Alpha	0.05				
6						
7	**Source of Variation**	**% of total variation**	**P value**	**P value summary**	**Significant?**	
8	Row Factor x Column Factor	29.97	<0.0001	****	Yes	
9	Row Factor	27.79	0.0245	*	Yes	
10	Column Factor	27.36	<0.0001	****	Yes	
11	Subject	13.58	0.0016	**	Yes	
12						
13	**ANOVA table**	**SS**	**DF**	**MS**	**F (DFn, DFd)**	**P value**
14	Row Factor x Column Factor	0.5405	3	0.1802	F (3, 8) = 61.85	P<0.0001
15	Row Factor	0.5011	3	0.1670	F (3, 8) = 5.455	P=0.0245
16	Column Factor	0.4934	1	0.4934	F (1, 8) = 169.4	P<0.0001
17	Subject	0.2450	8	0.03062	F (8, 8) = 10.51	P=0.0016
18	Residual	0.02330	8	0.002913		
19						
20	**Difference between column means**					
21	Mean of DMSO+	0.4379				
22	Mean of 10uM UC2288+	0.1511				
23	Difference between means	0.2868				
24	SE of difference	0.02203				
25	95% CI of difference	0.2360 to 0.3376				
26						
27	**Data summary**					
28	Number of columns (Column Factor)	2				
29	Number of rows (Row Factor)	4				
30	Number of subjects (Subject)	12				
31	Number of missing values	0				

2way ANOVA<br>Multiple comparisons						
1	Within each column, compare rows (simple effects within columns)					
2						
3	Number of families	2				
4	Number of comparisons per family	6				
5	Alpha	0.05				
6						
7	**Tukey's multiple comparisons test**	**Mean diff.**	**95.00% CI of diff.**	**Below threshold?**	**Summary**	**Adjusted P Value**
8						
9	DMSO+					
10	DMSO vs. 8 uM SFN	0.6169	0.3145 to 0.9194	Yes	***	0.0001
11	DMSO vs. 10 uM SFN	0.6443	0.3418 to 0.9468	Yes	****	<0.0001
12	DMSO vs. 12 uM SFN	0.7482	0.4457 to 1.051	Yes	****	<0.0001
13	8 uM SFN vs. 10 uM SFN	0.02739	-0.2751 to 0.3299	No	ns	0.9937
14	8 uM SFN vs. 12 uM SFN	0.1313	-0.1712 to 0.4338	No	ns	0.6107
15	10 uM SFN vs. 12 uM SFN	0.1039	-0.1986 to 0.4064	No	ns	0.7612
16						
17	10uM UC2288+					
18	DMSO vs. 8 uM SFN	0.01369	-0.2888 to 0.3162	No	ns	0.9992
19	DMSO vs. 10 uM SFN	-0.002737	-0.3052 to 0.2997	No	ns	>0.9999
20	DMSO vs. 12 uM SFN	-0.03149	-0.3340 to 0.2710	No	ns	0.9905
21	8 uM SFN vs. 10 uM SFN	-0.01643	-0.3189 to 0.2861	No	ns	0.9986
22	8 uM SFN vs. 12 uM SFN	-0.04519	-0.3477 to 0.2573	No	ns	0.9729
23	10 uM SFN vs. 12 uM SFN	-0.02876	-0.3312 to 0.2737	No	ns	0.9927
24						
25						
26	**Test details**	**Mean 1**	**Mean 2**	**Mean diff.**	**SE of diff.**	**N1**
27						
28	DMSO+					
29	DMSO vs. 8 uM SFN	0.9403	0.3233	0.6169	0.1057	3
30	DMSO vs. 10 uM SFN	0.9403	0.2960	0.6443	0.1057	3
31	DMSO vs. 12 uM SFN	0.9403	0.1921	0.7482	0.1057	3
32	8 uM SFN vs. 10 uM SFN	0.3233	0.2960	0.02739	0.1057	3
33	8 uM SFN vs. 12 uM SFN	0.3233	0.1921	0.1313	0.1057	3
34	10 uM SFN vs. 12 uM SFN	0.2960	0.1921	0.1039	0.1057	3
35						
36	10uM UC2288+					
37	DMSO vs. 8 uM SFN	0.1460	0.1323	0.01369	0.1057	3
38	DMSO vs. 10 uM SFN	0.1460	0.1487	-0.002737	0.1057	3
39	DMSO vs. 12 uM SFN	0.1460	0.1775	-0.03149	0.1057	3
40	8 uM SFN vs. 10 uM SFN	0.1323	0.1487	-0.01643	0.1057	3
41	8 uM SFN vs. 12 uM SFN	0.1323	0.1775	-0.04519	0.1057	3
42	10 uM SFN vs. 12 uM SFN	0.1487	0.1775	-0.02876	0.1057	3

1			
2			
3			
4			
5			
6			
7			
8			
9			
10			
11			
12			
13			
14			
15			
16			
17			
18			
19			
20			
21			
22			
23			
24			
25			
26	**N2**	**q**	**DF**
27			
28			
29	3	8.252	16.00
30	3	8.619	16.00
31	3	10.01	16.00
32	3	0.3664	16.00
33	3	1.756	16.00
34	3	1.390	16.00
35			
36			
37	3	0.1831	16.00
38	3	0.03662	16.00
39	3	0.4213	16.00
40	3	0.2198	16.00
41	3	0.6044	16.00
42	3	0.3846	16.00

2way ANOVA<br>ANOVA results					
Table Analyzed	hMDM SFN v UC2288 p21/bactin				
**Two-way RM ANOVA**	Matching: Across row				
Assume sphericity?	Yes				
Alpha	0.05				
**Source of Variation**	**% of total variation**	**P value**	**P value summary**	**Significant?**	
Row Factor x Column Factor	13.17	0.0160	*	Yes	
Row Factor	22.97	0.0001	***	Yes	
Column Factor	56.19	<0.0001	****	Yes	
Subject	2.207	0.8898	ns	No	
**ANOVA table**	**SS**	**DF**	**MS**	**F (DFn, DFd)**	**P value**
Row Factor x Column Factor	0.3938	3	0.1313	F (3, 8) = 6.416	P=0.0160
Row Factor	0.6870	3	0.2290	F (3, 8) = 27.76	P=0.0001
Column Factor	1.681	1	1.681	F (1, 8) = 82.15	P<0.0001
Subject	0.06601	8	0.008251	F (8, 8) = 0.4032	P=0.8898
Residual	0.1637	8	0.02046		
**Difference between column means**					
Mean of DMSO+	0.8074				
Mean of 10uM UC2288+	0.2781				
Difference between means	0.5293				
SE of difference	0.05840				
95% CI of difference	0.3946 to 0.6639				
**Data summary**					
Number of columns (Column Factor)	2				
Number of rows (Row Factor)	4				
Number of subjects (Subject)	12				
Number of missing values	0				

2way ANOVA<br>Multiple comparisons						
1	Within each column, compare rows (simple effects within columns)					
2						
3	Number of families	2				
4	Number of comparisons per family	3				
5	Alpha	0.05				
6						
7	**Dunnett's multiple comparisons test**	**Mean diff.**	**95.00% CI of diff.**	**Below threshold?**	**Summary**	**Adjusted P Value**
8						
9	DMSO+					
10	DMSO vs. 8 uM SFN	-0.5577	-0.8114 to -0.3041	Yes	****	<0.0001
11	DMSO vs. 10 uM SFN	-0.7537	-1.007 to -0.5001	Yes	****	<0.0001
12	DMSO vs. 12 uM SFN	-0.6174	-0.8710 to -0.3638	Yes	****	<0.0001
13						
14	10uM UC2288+					
15	DMSO vs. 8 uM SFN	-0.03800	-0.2916 to 0.2156	No	ns	0.9613
16	DMSO vs. 10 uM SFN	-0.05681	-0.3104 to 0.1968	No	ns	0.8885
17	DMSO vs. 12 uM SFN	-0.2261	-0.4798 to 0.02751	No	ns	0.0854
18						
19						
20	**Test details**	**Mean 1**	**Mean 2**	**Mean diff.**	**SE of diff.**	**N1**
21						
22	DMSO+					
23	DMSO vs. 8 uM SFN	0.3252	0.8829	-0.5577	0.09783	3
24	DMSO vs. 10 uM SFN	0.3252	1.079	-0.7537	0.09783	3
25	DMSO vs. 12 uM SFN	0.3252	0.9426	-0.6174	0.09783	3
26						
27	10uM UC2288+					
28	DMSO vs. 8 uM SFN	0.1979	0.2359	-0.03800	0.09783	3
29	DMSO vs. 10 uM SFN	0.1979	0.2547	-0.05681	0.09783	3
30	DMSO vs. 12 uM SFN	0.1979	0.4240	-0.2261	0.09783	3

1			
2			
3			
4			
5			
6			
7			
8			
9			
10			
11			
12			
13			
14			
15			
16			
17			
18			
19			
20	**N2**	**q**	**DF**
21			
22			
23	3	5.701	16.00
24	3	7.704	16.00
25	3	6.311	16.00
26			
27			
28	3	0.3884	16.00
29	3	0.5807	16.00
30	3	2.311	16.00

2way ANOVA<br>ANOVA results								
1	Table Analyzed	THP1 SFN v UC2288 PSAM/SAM						
2								
3	**Two-way ANOVA**	Ordinary						
4	Alpha	0.05						
5								
6	**Source of Variation**	**% of total variation**	**P value**	**P value summary**	**Significant?**			
7	Interaction	41.81	<0.0001	****	Yes			
8	Row Factor	37.59	<0.0001	****	Yes			
9	Column Factor	19.09	<0.0001	****	Yes			
10								
11	**ANOVA table**	**SS**	**DF**	**MS**	**F (DFn, DFd)**	**P value**		
12	Interaction	0.7565	3	0.2522	F (3, 16) = 147.4	P<0.0001		
13	Row Factor	0.6800	3	0.2267	F (3, 16) = 132.5	P<0.0001		
14	Column Factor	0.3453	1	0.3453	F (1, 16) = 201.8	P<0.0001		
15	Residual	0.02738	16	0.001711				
16	Total	1.809	23					
17								
18	**Difference between column means**							
19	Mean of DMSO+	0.4485						
20	Mean of 10uM UC2288+	0.2086						
21	Difference between means	0.2399						
22	SE of difference	0.01689						
23	95% CI of difference	0.2041 to 0.2757						
24								
25	**Data summary**							
26	Number of columns (Column Factor)	2						
27	Number of rows (Row Factor)	4						
28	Number of values	24						

2way ANOVA
Multiple comparisons

1	Within each column, compare rows (simple effects within columns)					
2						
3	Number of families	2				
4	Number of comparisons per family	3				
5	Alpha	0.05				
6						
7	**Dunnett's multiple comparisons test**	**Mean diff.**	**95.00% CI of diff.**	**Below threshold?**	**Summary**	**Adjusted P Value**
8						
9	DMSO+					
10	DMSO vs. 8 uM SFN	0.7825	0.6949 to 0.8701	Yes	****	<0.0001
11	DMSO vs. 10 uM SFN	0.7784	0.6908 to 0.8660	Yes	****	<0.0001
12	DMSO vs. 12 uM SFN	0.8305	0.7430 to 0.9181	Yes	****	<0.0001
13						
14	10uM UC2288+					
15	DMSO vs. 8 uM SFN	-0.01018	-0.09775 to 0.07738	No	ns	0.9810
16	DMSO vs. 10 uM SFN	-0.01933	-0.1069 to 0.06823	No	ns	0.8924
17	DMSO vs. 12 uM SFN	-0.03171	-0.1193 to 0.05585	No	ns	0.6752
18						
19						
20	**Test details**	**Mean 1**	**Mean 2**	**Mean diff.**	**SE of diff.**	**N1**
21						
22	DMSO+					
23	DMSO vs. 8 uM SFN	1.046	0.2639	0.7825	0.03378	3
24	DMSO vs. 10 uM SFN	1.046	0.2680	0.7784	0.03378	3
25	DMSO vs. 12 uM SFN	1.046	0.2159	0.8305	0.03378	3
26						
27	10uM UC2288+					
28	DMSO vs. 8 uM SFN	0.1933	0.2035	-0.01018	0.03378	3
29	DMSO vs. 10 uM SFN	0.1933	0.2127	-0.01933	0.03378	3
30	DMSO vs. 12 uM SFN	0.1933	0.2251	-0.03171	0.03378	3

1			
2			
3			
4			
5			
6			
7			
8			
9			
10			
11			
12			
13			
14			
15			
16			
17			
18			
19			
20	**N2**	**q**	**DF**
21			
22			
23	3	23.17	16.00
24	3	23.05	16.00
25	3	24.59	16.00
26			
27			
28	3	0.3015	16.00
29	3	0.5724	16.00
30	3	0.9389	16.00

2way ANOVA<br>ANOVA results								
1	Table Analyzed	THP1 SFN v UC2288 PSAM/SAM						
2								
3	**Two-way ANOVA**	Ordinary						
4	Alpha	0.05						
5								
6	**Source of Variation**	**% of total variation**	**P value**	**P value summary**	**Significant?**			
7	Interaction	41.81	<0.0001	****	Yes			
8	Row Factor	37.59	<0.0001	****	Yes			
9	Column Factor	19.09	<0.0001	****	Yes			
10								
11	**ANOVA table**	**SS**	**DF**	**MS**	**F (DFn, DFd)**	**P value**		
12	Interaction	0.7565	3	0.2522	F (3, 16) = 147.4	P<0.0001		
13	Row Factor	0.6800	3	0.2267	F (3, 16) = 132.5	P<0.0001		
14	Column Factor	0.3453	1	0.3453	F (1, 16) = 201.8	P<0.0001		
15	Residual	0.02738	16	0.001711				
16	Total	1.809	23					
17								
18	**Difference between column means**							
19	Mean of DMSO+	0.4485						
20	Mean of 10uM UC2288+	0.2086						
21	Difference between means	0.2399						
22	SE of difference	0.01689						
23	95% CI of difference	0.2041 to 0.2757						
24								
25	**Data summary**							
26	Number of columns (Column Factor)	2						
27	Number of rows (Row Factor)	4						
28	Number of values	24						

2way ANOVA
Multiple comparisons

1	Within each column, compare rows (simple effects within columns)					
2						
3	Number of families	2				
4	Number of comparisons per family	6				
5	Alpha	0.05				
6						
7	**Tukey's multiple comparisons test**	**Mean diff.**	**95.00% CI of diff.**	**Below threshold?**	**Summary**	**Adjusted P Value**
8						
9	DMSO+					
10	DMSO vs. 8 uM SFN	0.7825	0.6859 to 0.8791	Yes	****	<0.0001
11	DMSO vs. 10 uM SFN	0.7784	0.6818 to 0.8750	Yes	****	<0.0001
12	DMSO vs. 12 uM SFN	0.8305	0.7339 to 0.9272	Yes	****	<0.0001
13	8 uM SFN vs. 10 uM SFN	-0.004107	-0.1007 to 0.09253	No	ns	0.9993
14	8 uM SFN vs. 12 uM SFN	0.04803	-0.04861 to 0.1447	No	ns	0.5045
15	10 uM SFN vs. 12 uM SFN	0.05213	-0.04450 to 0.1488	No	ns	0.4364
16						
17	10uM UC2288+					
18	DMSO vs. 8 uM SFN	-0.01018	-0.1068 to 0.08645	No	ns	0.9901
19	DMSO vs. 10 uM SFN	-0.01933	-0.1160 to 0.07730	No	ns	0.9389
20	DMSO vs. 12 uM SFN	-0.03171	-0.1283 to 0.06492	No	ns	0.7847
21	8 uM SFN vs. 10 uM SFN	-0.009150	-0.1058 to 0.08748	No	ns	0.9928
22	8 uM SFN vs. 12 uM SFN	-0.02153	-0.1182 to 0.07511	No	ns	0.9184
23	10 uM SFN vs. 12 uM SFN	-0.01238	-0.1090 to 0.08426	No	ns	0.9826
24						
25						
26	**Test details**	**Mean 1**	**Mean 2**	**Mean diff.**	**SE of diff.**	**N1**
27						
28	DMSO+					
29	DMSO vs. 8 uM SFN	1.046	0.2639	0.7825	0.03378	3
30	DMSO vs. 10 uM SFN	1.046	0.2680	0.7784	0.03378	3
31	DMSO vs. 12 uM SFN	1.046	0.2159	0.8305	0.03378	3
32	8 uM SFN vs. 10 uM SFN	0.2639	0.2680	-0.004107	0.03378	3
33	8 uM SFN vs. 12 uM SFN	0.2639	0.2159	0.04803	0.03378	3
34	10 uM SFN vs. 12 uM SFN	0.2680	0.2159	0.05213	0.03378	3
35						
36	10uM UC2288+					
37	DMSO vs. 8 uM SFN	0.1933	0.2035	-0.01018	0.03378	3
38	DMSO vs. 10 uM SFN	0.1933	0.2127	-0.01933	0.03378	3
39	DMSO vs. 12 uM SFN	0.1933	0.2251	-0.03171	0.03378	3
40	8 uM SFN vs. 10 uM SFN	0.2035	0.2127	-0.009150	0.03378	3
41	8 uM SFN vs. 12 uM SFN	0.2035	0.2251	-0.02153	0.03378	3
42	10 uM SFN vs. 12 uM SFN	0.2127	0.2251	-0.01238	0.03378	3

1			
2			
3			
4			
5			
6			
7			
8			
9			
10			
11			
12			
13			
14			
15			
16			
17			
18			
19			
20			
21			
22			
23			
24			
25			
26	**N2**	**q**	**DF**
27			
28			
29	3	32.76	16.00
30	3	32.59	16.00
31	3	34.78	16.00
32	3	0.1719	16.00
33	3	2.011	16.00
34	3	2.183	16.00
35			
36			
37	3	0.4264	16.00
38	3	0.8095	16.00
39	3	1.328	16.00
40	3	0.3831	16.00
41	3	0.9013	16.00
42	3	0.5182	16.00

2way ANOVA
ANOVA results

1	Table Analyzed	THP1 SFN v UC2288 p21/bactin						
2								
3	**Two-way ANOVA**	Ordinary						
4	Alpha	0.05						
5								
6	**Source of Variation**	**% of total variation**	**P value**	**P value summary**	**Significant?**			
7	Interaction	0.2853	0.8576	ns	No			
8	Row Factor	0.1515	0.9379	ns	No			
9	Column Factor	93.56	<0.0001	****	Yes			
10								
11	**ANOVA table**	**SS**	**DF**	**MS**	**F (DFn, DFd)**	**P value**		
12	Interaction	0.005721	3	0.001907	F (3, 16) = 0.2536	P=0.8576		
13	Row Factor	0.003039	3	0.001013	F (3, 16) = 0.1347	P=0.9379		
14	Column Factor	1.876	1	1.876	F (1, 16) = 249.5	P<0.0001		
15	Residual	0.1203	16	0.007520				
16	Total	2.005	23					
17								
18	**Difference between column means**							
19	Mean of DMSO+	0.9096						
20	Mean of 10uM UC2288+	0.3504						
21	Difference between means	0.5592						
22	SE of difference	0.03540						
23	95% CI of difference	0.4842 to 0.6343						
24								
25	**Data summary**							
26	Number of columns (Column Factor)	2						
27	Number of rows (Row Factor)	4						
28	Number of values	24						

2way ANOVA<br>Multiple comparisons						
1	Within each column, compare rows (simple effects within columns)					
2						
3	Number of families	2				
4	Number of comparisons per family	3				
5	Alpha	0.05				
6						
7	**Dunnett's multiple comparisons test**	**Mean diff.**	**95.00% CI of diff.**	**Below threshold?**	**Summary**	**Adjusted P Value**
8						
9	DMSO+					
10	DMSO vs. 8 uM SFN	-0.03122	-0.2148 to 0.1524	No	ns	0.9454
11	DMSO vs. 10 uM SFN	-0.04732	-0.2309 to 0.1363	No	ns	0.8434
12	DMSO vs. 12 uM SFN	-0.002144	-0.1857 to 0.1814	No	ns	>0.9999
13						
14	10uM UC2288+					
15	DMSO vs. 8 uM SFN	0.007967	-0.1756 to 0.1915	No	ns	0.9989
16	DMSO vs. 10 uM SFN	0.03987	-0.1437 to 0.2234	No	ns	0.8967
17	DMSO vs. 12 uM SFN	0.04013	-0.1434 to 0.2237	No	ns	0.8951
18						
19						
20	**Test details**	**Mean 1**	**Mean 2**	**Mean diff.**	**SE of diff.**	**N1**
21						
22	DMSO+					
23	DMSO vs. 8 uM SFN	0.8895	0.9207	-0.03122	0.07081	3
24	DMSO vs. 10 uM SFN	0.8895	0.9368	-0.04732	0.07081	3
25	DMSO vs. 12 uM SFN	0.8895	0.8916	-0.002144	0.07081	3
26						
27	10uM UC2288+					
28	DMSO vs. 8 uM SFN	0.3724	0.3644	0.007967	0.07081	3
29	DMSO vs. 10 uM SFN	0.3724	0.3325	0.03987	0.07081	3
30	DMSO vs. 12 uM SFN	0.3724	0.3323	0.04013	0.07081	3

1			
2			
3			
4			
5			
6			
7			
8			
9			
10			
11			
12			
13			
14			
15			
16			
17			
18			
19			
20	**N2**	**q**	**DF**
21			
22			
23	3	0.4409	16.00
24	3	0.6682	16.00
25	3	0.03028	16.00
26			
27			
28	3	0.1125	16.00
29	3	0.5631	16.00
30	3	0.5667	16.00

2way ANOVA<br>ANOVA results									
1	Table Analyze	hMDMp21siR							
2									
3	**Two-way RM**	Matching: Acr							
4	Assume spl	Yes							
5	Alpha	0.05							
6									
7	**Source of Va**	**% of total var**	**P value**	**P value sumr**	**Significant?**				
8	Row Factor	8.604	0.0750	ns	No				
9	Row Factor	2.420	0.2960	ns	No				
10	Time	70.13	<0.0001	****	Yes				
11	Subject	7.789	0.6949	ns	No				
12									
13	**ANOVA table**	**SS**	**DF**	**MS**	**F (DFn, DFd)**	**P value**			
14	Row Factor	0.8595	2	0.4298	F (2, 9) = 3.50	P=0.0750			
15	Row Factor	0.2417	2	0.1209	F (2, 9) = 1.39	P=0.2960			
16	Time	7.006	1	7.006	F (1, 9) = 57.0	P<0.0001			
17	Subject	0.7781	9	0.08646	F (9, 9) = 0.70	P=0.6949			
18	Residual	1.105	9	0.1227					
19									
20	**Difference be**								
21	Mean of nt	0.4651							
22	Mean of p2	1.546							
23	Difference b	-1.081							
24	SE of differe	0.1430							
25	95% CI of c	-1.404 to -0.7							
26									
27	**Data summa**								
28	Number of	2							
29	Number of	3							
30	Number of	12							
31	Number of	0							

2way ANOVA<br>Multiple comparisons									
1	Within each c								
2									
3	Number of far	2							
4	Number of co	2							
5	Alpha	0.05							
6									
7	**Dunnett's mu**	**Mean diff.**	**95.00% CI of**	**Below thresh**	**Summary**	**Adjusted P Va**			
8									
9	nt siRNA (po								
10	DMSO vs. 5	0.5252	-0.02331 to 1	No	ns	0.0612			
11	DMSO vs. 8	0.5541	0.005601 to 1	Yes	*	0.0476			
12									
13	p21 (pooled)								
14	DMSO vs. 5	-0.3792	-0.9277 to 0.1	No	ns	0.1967			
15	DMSO vs. 8	-0.07454	-0.6231 to 0.4	No	ns	0.9258			
16									
17									
18	**Test details**	**Mean 1**	**Mean 2**	**Mean diff.**	**SE of diff.**	**N1**	**N2**	**q**	**DF**
19									
20	nt siRNA (po								
21	DMSO vs. 5	0.8249	0.2997	0.5252	0.2287	4	4	2.297	18.00
22	DMSO vs. 8	0.8249	0.2708	0.5541	0.2287	4	4	2.423	18.00
23									
24	p21 (pooled)								
25	DMSO vs. 5	1.394	1.774	-0.3792	0.2287	4	4	1.658	18.00
26	DMSO vs. 8	1.394	1.469	-0.07454	0.2287	4	4	0.3260	18.00

2way ANOVA<br>ANOVA results									
1	Table Analyze	hMDMp21siR							
2									
3	**Two-way RM**	Matching: Acr							
4	Assume spl	Yes							
5	Alpha	0.05							
6									
7	**Source of Va**	**% of total var**	**P value**	**P value sumr**	**Significant?**				
8	Row Factor	0.09529	0.9694	ns	No				
9	Row Factor	4.128	0.2839	ns	No				
10	Time	69.23	<0.0001	****	Yes				
11	Subject	12.79	0.5427	ns	No				
12									
13	**ANOVA table**	**SS**	**DF**	**MS**	**F (DFn, DFd)**	**P value**			
14	Row Factor	0.01023	2	0.005116	F (2, 9) = 0.03	P=0.9694			
15	Row Factor	0.4433	2	0.2217	F (2, 9) = 1.45	P=0.2839			
16	Time	7.435	1	7.435	F (1, 9) = 45.2	P<0.0001			
17	Subject	1.373	9	0.1526	F (9, 9) = 0.92	P=0.5427			
18	Residual	1.478	9	0.1642					
19									
20	**Difference be**								
21	Mean of nt :	1.742							
22	Mean of p2	0.6291							
23	Difference b	1.113							
24	SE of differe	0.1654							
25	95% CI of c	0.7389 to 1.4[illegible]							
26									
27	**Data summa**								
28	Number of (	2							
29	Number of r	3							
30	Number of :	12							
31	Number of r	0							

2way ANOVA<br>Multiple comparisons									
1	Within each c								
2									
3	Number of far	2							
4	Number of co	2							
5	Alpha	0.05							
6									
7	**Dunnett's mu**	**Mean diff.**	**95.00% CI of**	**Below thresh**	**Summary**	**Adjusted P Va**			
8									
9	nt siRNA (po								
10	DMSO vs. 5	-0.2443	-0.9193 to 0.4	No	ns	0.6000			
11	DMSO vs. 8	-0.3743	-1.049 to 0.30	No	ns	0.3299			
12									
13	p21 (pooled)								
14	DMSO vs. 5	-0.1570	-0.8320 to 0.5	No	ns	0.8021			
15	DMSO vs. 8	-0.2864	-0.9614 to 0.3	No	ns	0.5039			
16									
17									
18	**Test details**	**Mean 1**	**Mean 2**	**Mean diff.**	**SE of diff.**	**N1**	**N2**	**q**	**DF**
19									
20	nt siRNA (po								
21	DMSO vs. 5	1.536	1.780	-0.2443	0.2814	4	4	0.8680	18.00
22	DMSO vs. 8	1.536	1.910	-0.3743	0.2814	4	4	1.330	18.00
23									
24	p21 (pooled)								
25	DMSO vs. 5	0.4813	0.6382	-0.1570	0.2814	4	4	0.5579	18.00
26	DMSO vs. 8	0.4813	0.7677	-0.2864	0.2814	4	4	1.018	18.00

2way ANOVA<br>ANOVA results								
1	Table Analyze	THP1p21KOS						
2								
3	**Two-way AN(**	Ordinary						
4	Alpha	0.05						
5								
6	**Source of Va**	**% of total var**	**P value**	**P value sumr**	**Significant?**			
7	Interaction	4.750	0.3360	ns	No			
8	Row Factor	4.385	0.1334	ns	No			
9	Column Fa	73.38	<0.0001	****	Yes			
10								
11	**ANOVA table**	**SS**	**DF**	**MS**	**F (DFn, DFd)**	**P value**		
12	Interaction	0.1971	4	0.04927	F (4, 18) = 1.2	P=0.3360		
13	Row Factor	0.1819	2	0.09097	F (2, 18) = 2.2	P=0.1334		
14	Column Fa	3.045	2	1.522	F (2, 18) = 37	P<0.0001		
15	Residual	0.7253	18	0.04030				
16	Total	4.149	26					
17								
18	**Data summa**							
19	Number of	3						
20	Number of	3						
21	Number of	27						

2way ANOVA<br>Multiple comparisons									
1	Within each c								
2									
3	Number of far	3							
4	Number of co	2							
5	Alpha	0.05							
6									
7	**Dunnett's mu**	**Mean diff.**	**95.00% CI of**	**Below thresh**	**Summary**	**Adjusted P Va**			
8									
9	nt gRNA								
10	DMSO vs.	0.3923	-0.0008155 to	No	ns	0.0505			
11	DMSO vs.	0.4042	0.01107 to 0.7	Yes	*	0.0437			
12									
13	p21 gRNA 1								
14	DMSO vs.	0.005017	-0.3881 to 0.3	No	ns	0.9993			
15	DMSO vs.	0.03422	-0.3589 to 0.4	No	ns	0.9686			
16									
17	p21 gRNA 2								
18	DMSO vs.	-0.02489	-0.4180 to 0.3	No	ns	0.9832			
19	DMSO vs.	0.1587	-0.2344 to 0.5	No	ns	0.5350			
20									
21									
22	**Test details**	**Mean 1**	**Mean 2**	**Mean diff.**	**SE of diff.**	**N1**	**N2**	**q**	**DF**
23									
24	nt gRNA								
25	DMSO vs.	0.5215	0.1291	0.3923	0.1639	3	3	2.394	18.00
26	DMSO vs.	0.5215	0.1173	0.4042	0.1639	3	3	2.466	18.00
27									
28	p21 gRNA 1								
29	DMSO vs.	0.9997	0.9947	0.005017	0.1639	3	3	0.03061	18.00
30	DMSO vs.	0.9997	0.9655	0.03422	0.1639	3	3	0.2088	18.00
31									
32	p21 gRNA 2								
33	DMSO vs.	0.9930	1.018	-0.02489	0.1639	3	3	0.1518	18.00
34	DMSO vs.	0.9930	0.8343	0.1587	0.1639	3	3	0.9683	18.00

2way ANOVA
ANOVA results

1	Table Analyzed	THP1p21KOSFNTransduction						
2								
3	**Two-way ANOVA**	Ordinary						
4	Alpha	0.05						
5								
6	**Source of Variation**	**% of total variation**	**P value**	**P value summary**	**Significant?**			
7	Interaction	6.744	0.0060	**	Yes			
8	Row Factor	4.160	0.0081	**	Yes			
9	Column Factor	83.22	<0.0001	****	Yes			
10								
11	**ANOVA table**	**SS**	**DF**	**MS**	**F (DFn, DFd)**	**P value**		
12	Interaction	768917604039	4	192229401010	F (4, 18) = 5.162	P=0.0060		
13	Row Factor	474292020119	2	237146010060	F (2, 18) = 6.369	P=0.0081		
14	Column Factor	9488643129897	2	4744321564948	F (2, 18) = 127.4	P<0.0001		
15	Residual	670258327510	18	37236573751				
16	Total	11402111081565	26					
17								
18	**Data summary**							
19	Number of columns (Column Factor)	3						
20	Number of rows (Row Factor)	3						
21	Number of values	27						

2way ANOVA<br>Multiple comparisons									
1	Within each c								
2									
3	Number of far	3							
4	Number of co	3							
5	Alpha	0.05							
6									
7	**Tukey's multi**	**Mean diff.**	**95.00% CI of**	**Below thresh**	**Summary**	**Adjusted P Va**			
8									
9	nt gRNA								
10	DMSO vs. 1	531322	129209 to 93	Yes	**	0.0090			
11	DMSO vs. 1	867837	465725 to 12	Yes	****	<0.0001			
12	10 uM SFN	336515	-65597 to 738	No	ns	0.1106			
13									
14	p21 gRNA 1								
15	DMSO vs. 1	-159430	-561543 to 24	No	ns	0.5791			
16	DMSO vs. 1	32428	-369684 to 43	No	ns	0.9769			
17	10 uM SFN	191859	-210254 to 59	No	ns	0.4583			
18									
19	p21 gRNA 2								
20	DMSO vs. 1	144317	-257795 to 54	No	ns	0.6375			
21	DMSO vs. 1	73090	-329023 to 47	No	ns	0.8889			
22	10 uM SFN	-71227	-473340 to 33	No	ns	0.8941			
23									
24									
25	**Test details**	**Mean 1**	**Mean 2**	**Mean diff.**	**SE of diff.**	**N1**	**N2**	**q**	**DF**
26									
27	nt gRNA								
28	DMSO vs. 1	1289151	757829	531322	157558	3	3	4.769	18.00
29	DMSO vs. 1	1289151	421313	867837	157558	3	3	7.790	18.00
30	10 uM SFN	757829	421313	336515	157558	3	3	3.021	18.00
31									
32	p21 gRNA 1								
33	DMSO vs. 1	2081477	2240907	-159430	157558	3	3	1.431	18.00
34	DMSO vs. 1	2081477	2049049	32428	157558	3	3	0.2911	18.00
35	10 uM SFN	2240907	2049049	191859	157558	3	3	1.722	18.00
36									
37	p21 gRNA 2								
38	DMSO vs. 1	987278	842961	144317	157558	3	3	1.295	18.00
39	DMSO vs. 1	987278	914188	73090	157558	3	3	0.6560	18.00
40	10 uM SFN	842961	914188	-71227	157558	3	3	0.6393	18.00

		elow threshold	P value	Mean of DMSO	ean of 10uM SI	Difference	SE of difference	t ratio	df	djusted P Valu
1	Cytoplasm	Yes	0.001623	27368	35464	-8096	1068	7.582	4.000	0.003242
2	Nucleus	Yes	0.000213	15315	8539	6776	528.1	12.83	4.000	0.000638
3	Total Cell	No	0.350446	42683	44003	-1320	1250	1.056	4.000	0.350446

		P value	Mean of DMSO	ean of 10uM SI	Difference	SE of difference	t ratio	df	djusted P Valu
1	Nucleus	0.000213	15315	8539	6776	528.1	12.83	4.000	0.000638
2	Cytoplasm	0.001623	27368	35464	-8096	1068	7.582	4.000	0.003242

1	Table Analyze	THP1fractiona
2		
3	Column A	DMSO
4	vs.	vs.
5	Column B	10uM SFN
6		
7	**Test details**	
8	Test name	Unpaired t tes
9	Variance assu	Individual vari
10	Multiple comp	Set P value th
11	Method	Holm-Šídák m
12	Alpha	0.05
13		
14	Number of tes	3
15	Number of rov	0

2way ANOVA<br>ANOVA results								
1	Table Analyze	THP1fractiona						
2								
3	**Two-way AN(**	Ordinary						
4	Alpha	0.05						
5								
6	**Source of Va**	**% of total var**	**P value**	**P value sumr**	**Significant?**			
7	Interaction	5.188	<0.0001	****	Yes			
8	Row Factor	94.15	<0.0001	****	Yes			
9	Column Fa	0.1087	0.1522	ns	No			
10								
11	**ANOVA table**	**SS**	**DF**	**MS**	**F (DFn, DFd)**	**P value**		
12	Interaction	166298078	2	83149039	F (2, 12) = 55	P<0.0001		
13	Row Factor	3018030806	2	1509015403	F (2, 12) = 10	P<0.0001		
14	Column Fa	3484830	1	3484830	F (1, 12) = 2.3	P=0.1522		
15	Residual	17884650	12	1490387				
16	Total	3205698364	17					
17								
18	**Difference b**							
19	Mean of DN	28455						
20	Mean of 10	29335						
21	Difference b	-880.0						
22	SE of differe	575.5						
23	95% CI of d	-2134 to 373.						
24								
25	**Data summa**							
26	Number of	2						
27	Number of	3						
28	Number of	18						

**2way ANOVA** Multiple comparisons									
1	Compare eac								
2									
3	Number of far	1							
4	Number of co	3							
5	Alpha	0.05							
6									
7	**Šídák's multi**	**Mean diff.**	**95.00% CI of**	**Below thresh**	**Summary**	**Adjusted P Va**			
8									
9	DMSO - 10u								
10	Cytoplasm	-8096	-10857 to -53	Yes	****	<0.0001			
11	Nucleus	6776	4014 to 9537	Yes	****	<0.0001			
12	Total Cell	-1320	-4081 to 1441	No	ns	0.5071			
13									
14									
15	**Test details**	**Mean 1**	**Mean 2**	**Mean diff.**	**SE of diff.**	**N1**	**N2**	**t**	**DF**
16									
17	DMSO - 10u								
18	Cytoplasm	27368	35464	-8096	996.8	3	3	8.122	12.00
19	Nucleus	15315	8539	6776	996.8	3	3	6.797	12.00
20	Total Cell	42683	44003	-1320	996.8	3	3	1.324	12.00

		elow threshold	P value	Mean of DMSO	ean of 10uM SI	Difference	SE of difference	t ratio	df	djusted P Valu
1	Cytoplasm	No	0.044827	14530	55477	-40947	14197	2.884	4.000	0.128543
2	Nucleus	No	0.975244	9958	10104	-146.2	4429	0.03302	4.000	0.975244
3	Total Cell	No	0.086720	24488	65581	-41093	18186	2.260	4.000	0.165919

		P value	Mean of DMSC	ean of 10uM SI	Difference	SE of difference	t ratio	df	djusted P Valu

1	Table Analyze	hMDMfraction
2		
3	Column A	DMSO
4	vs.	vs.
5	Column B	10uM SFN
6		
7	**Test details**	
8	Test name	Unpaired t tes
9	Variance assu	Individual vari
10	Multiple comp	Set P value th
11	Method	Holm-Šídák m
12	Alpha	0.05
13		
14	Number of tes	3
15	Number of rov	0

2way ANOVA<br>ANOVA results									
1	Table Analyze	hMDMfraction							
2									
3	**Two-way RM**	Matching: Acr							
4	Assume spl	Yes							
5	Alpha	0.05							
6									
7	**Source of Va**	**% of total var**	**P value**	**P value summ**	**Significant?**				
8	Row Factor	13.63	0.0189	*	Yes				
9	Row Factor	31.81	0.0687	ns	No				
10	Column Fa	27.55	0.0012	**	Yes				
11	Subject	22.07	0.0458	*	Yes				
12									
13	**ANOVA table**	**SS**	**DF**	**MS**	**F (DFn, DFd)**	**P value**			
14	Row Factor	1670663248	2	835331624	F (2, 6) = 8.26	P=0.0189			
15	Row Factor	3898986892	2	1949493446	F (2, 6) = 4.32	P=0.0687			
16	Column Fa	3377249409	1	3377249409	F (1, 6) = 33.4	P=0.0012			
17	Subject	2704809524	6	450801587	F (6, 6) = 4.45	P=0.0458			
18	Residual	606639946	6	101106658					
19									
20	**Difference be**								
21	Mean of DM	16325							
22	Mean of 10	43721							
23	Difference b	-27395							
24	SE of differe	4740							
25	95% CI of d	-38994 to -15							
26									
27	**Data summa**								
28	Number of	2							
29	Number of	3							
30	Number of	9							
31	Number of	0							

2way ANOVA
Multiple comparisons

1	Compare eac								
2									
3	Number of far	1							
4	Number of co	3							
5	Alpha	0.05							
6									
7	**Šídák's multi**	**Mean diff.**	**95.00% CI of**	**Below thresh**	**Summary**	**Adjusted P Va**			
8									
9	DMSO - 10u								
10	Cytoplasm	-40947	-67825 to -14	Yes	**	0.0074			
11	Nucleus	-146.2	-27025 to 267	No	ns	>0.9999			
12	Total Cell	-41093	-67971 to -14	Yes	**	0.0073			
13									
14									
15	**Test details**	**Mean 1**	**Mean 2**	**Mean diff.**	**SE of diff.**	**N1**	**N2**	**t**	**DF**
16									
17	DMSO - 10u								
18	Cytoplasm	14530	55477	-40947	8210	3	3	4.987	6.000
19	Nucleus	9958	10104	-146.2	8210	3	3	0.01781	6.000
20	Total Cell	24488	65581	-41093	8210	3	3	5.005	6.000

1	Table Analyze	T145P				
2	Data sets ana	A-D				
3	Distribution as	Normal (Gaus				
4						
5	**ANOVA sumr**					
6	F	46.69				
7	P value	<0.0001				
8	P value sum	****				
9	Significant di	Yes				
10	R squared	0.9460				
11						
12	**Brown-Forsy**					
13	F (DFn, DFd	0.2601 (3, 8)				
14	P value	0.8522				
15	P value sum	ns				
16	Are SDs sign	No				
17						
18	**Bartlett's tes**					
19	Bartlett's stat					
20	P value					
21	P value sum					
22	Are SDs sign					
23						
24	**ANOVA table**	**SS**	**DF**	**MS**	**F (DFn, DFd)**	**P value**
25	Treatment (b	0.2373	3	0.07910	F (3, 8) = 46.6	P<0.0001
26	Residual (wit	0.01355	8	0.001694		
27	Total	0.2508	11			
28						
29	**Data summa**					
30	Number of tr	4				
31	Number of v	12				

1	Number of far	1							
2	Number of co	3							
3	Alpha	0.05							
4									
5	**Dunnett's mu**	**Mean diff.**	**95.00% CI of**	**Below thresh**	**Summary**	**Adjusted P Va**	**A-?**		
6	DMSO vs. 5	-0.07687	-0.1736 to 0.0	No	ns	0.1204	B	5 uM SFN	
7	DMSO vs. 10	-0.2230	-0.3198 to -0.	Yes	***	0.0004	C	10 uM SFN	
8	DMSO vs. 15	-0.3669	-0.4636 to -0.	Yes	****	<0.0001	D	15 uM SFN	
9									
10	**Test details**	**Mean 1**	**Mean 2**	**Mean diff.**	**SE of diff.**	**n1**	**n2**	**q**	**DF**
11	DMSO vs. 5	0.2208	0.2977	-0.07687	0.03361	3	3	2.287	8
12	DMSO vs. 10	0.2208	0.4438	-0.2230	0.03361	3	3	6.636	8
13	DMSO vs. 15	0.2208	0.5877	-0.3669	0.03361	3	3	10.92	8

1	Table Analyze	T145P p21/ba				
2	Data sets ana	A-D				
3	Distribution as	Normal (Gaus				
4						
5	**ANOVA sumr**					
6	F	0.6039				
7	P value	0.6307				
8	P value sum	ns				
9	Significant di	No				
10	R squared	0.1846				
11						
12	**Brown-Forsy**					
13	F (DFn, DFd	0.1681 (3, 8)				
14	P value	0.9149				
15	P value sum	ns				
16	Are SDs sign	No				
17						
18	**Bartlett's tes**					
19	Bartlett's stat					
20	P value					
21	P value sum					
22	Are SDs sign					
23						
24	**ANOVA table**	**SS**	**DF**	**MS**	**F (DFn, DFd)**	**P value**
25	Treatment (b	0.03888	3	0.01296	F (3, 8) = 0.60	P=0.6307
26	Residual (wit	0.1717	8	0.02146		
27	Total	0.2106	11			
28						
29	**Data summa**					
30	Number of tr	4				
31	Number of v	12				

1	Number of far	1						
2	Number of co	3						
3	Alpha	0.05						
4								
5	**Dunnett's mu**	**Mean diff.**	**95.00% CI of**	**Below thresh**	**Summary**	**Adjusted P Va**	**A-?**		
6	DMSO vs. 5	-0.07730	-0.4218 to 0.2	No	ns	0.8552	B	5 uM SFN	
7	DMSO vs. 1(	-0.1186	-0.4631 to 0.2	No	ns	0.6489	C	10 uM SFN	
8	DMSO vs. 1!	-0.1526	-0.4970 to 0.1	No	ns	0.4784	D	15 uM SFN	
9									
10	**Test details**	**Mean 1**	**Mean 2**	**Mean diff.**	**SE of diff.**	**n1**	**n2**	**q**	**DF**
11	DMSO vs. 5	0.7999	0.8772	-0.07730	0.1196	3	3	0.6462	8
12	DMSO vs. 1(	0.7999	0.9186	-0.1186	0.1196	3	3	0.9918	8
13	DMSO vs. 1!	0.7999	0.9525	-0.1526	0.1196	3	3	1.275	8

		A	B	C	D
		DMSO	10 uM SFN	15 uM SFN	20 uM SFN
1	**Test for norm**				
2	**Shapiro-Wil**				
3	W	0.9985	0.9601	0.7815	0.8654
4	P value	0.9258	0.6158	0.0710	0.2825
5	Passed nor	Yes	Yes	Yes	Yes
6	P value sur	ns	ns	ns	ns
7					
8	**Number of va**	3	3	3	3

		A	B	C	D
		DMSO	10 uM SFN	15 uM SFN	20 uM SFN
1	**Test for norm**				
2	**Shapiro-Wil**				
3	W	0.9772	0.9831	0.9093	0.9394
4	P value	0.7108	0.7507	0.4157	0.5249
5	Passed nor	Yes	Yes	Yes	Yes
6	P value sun	ns	ns	ns	ns
7					
8	**Number of va**	3	3	3	3

		A	B	C	D
		DMSO	10 uM SFN	15 uM SFN	20 µM SFN
1	**Test for norm**				
2	**Shapiro-Wil**				
3	W	0.9890	0.9187	0.7767	0.9671
4	P value	0.7996	0.4476	0.0600	0.6518
5	Passed nor	Yes	Yes	Yes	Yes
6	P value sun	ns	ns	ns	ns
7					
8	**Number of va**	3	3	3	3

		A	B	C	D
		DMSO	10 uM SFN	15 uM SFN	20 µM SFN
1	**Test for norm**				
2	**Shapiro-Wil**				
3	W	0.9893	0.9149	0.9675	0.9961
4	P value	0.8023	0.4346	0.6536	0.8806
5	Passed nor	Yes	Yes	Yes	Yes
6	P value sun	ns	ns	ns	ns
7					
8	**Number of va**	3	3	3	3

		A	B	C
		DMSO	10 uM SFN	100 U/mL IFNy
1	**Test for norm**			
2	**Shapiro-Wil**			
3	W	0.8611	0.8572	0.9438
4	P value	0.2706	0.2598	0.5427
5	Passed nor	Yes	Yes	Yes
6	P value sun	ns	ns	ns
7				
8	**Number of va**	3	3	3

		A	B	C
		DMSO	10 uM SFN	100 U/mL IFNy
1	**Test for norm**			
2	**Shapiro-Wil**			
3	W	0.7789	0.7570	0.9971
4	P value	0.0650	0.0155	0.8970
5	Passed nor	Yes	No	Yes
6	P value sun	ns	*	ns
7				
8	**Number of va**	3	3	3

		A	B	C
		DMSO	10 uM SFN	100 U/mL IFNy
1	**Test for norm**			
2	**Shapiro-Wil**			
3	W	0.7622	0.8179	0.8057
4	P value	0.0271	0.1580	0.1282
5	Passed nor	No	Yes	Yes
6	P value sun	*	ns	ns
7				
8	**Number of va**	3	3	3

		A	B	C
		DMSO	10 uM SFN	100 U/mL IFNy
1	**Test for norm**			
2	**Shapiro-Will**			
3	W	0.9943	0.9998	0.9889
4	P value	0.8555	0.9723	0.7986
5	Passed nor	Yes	Yes	Yes
6	P value sun	ns	ns	ns
7				
8	**Number of va**	3	3	3

		A	B	C	D
		DMSO	5uM UC2288	10uM UC2288	15uM UC2288
1	**Test for norm**				
2	**Shapiro-Wil**				
3	W	0.8616	0.9134	0.7980	0.9999
4	P value	0.2719	0.4294	0.1098	0.9807
5	Passed nor	Yes	Yes	Yes	Yes
6	P value sun	ns	ns	ns	ns
7					
8	**Number of va**	3	3	3	3

		A	B	C	D
		DMSO	5uM UC2288	10uM UC2288	15uM UC2288
1	**Test for norm**				
2	**Shapiro-Wil**				
3	W	0.7554	0.8348	0.8395	0.9946
4	P value	0.0120	0.2007	0.2128	0.8597
5	Passed nor	No	Yes	Yes	Yes
6	P value sun	*	ns	ns	ns
7					
8	**Number of va**	3	3	3	3

		A	B	C	D
		DMSO	5uM UC2288	10uM UC2288	15uM UC2288
1	**Test for norm**				
2	**Shapiro-Wil**				
3	W	0.9228	0.9443	0.9386	0.8522
4	P value	0.4624	0.5451	0.5219	0.2464
5	Passed nor	Yes	Yes	Yes	Yes
6	P value sun	ns	ns	ns	ns
7					
8	**Number of va**	3	3	3	3

		A	B	C	D
		DMSO	5uM UC2288	10uM UC2288	15uM UC2288
1	**Test for norm**				
2	**Shapiro-Will**				
3	W	0.7786	0.9093	0.9244	0.9793
4	P value	0.0644	0.4158	0.4681	0.7243
5	Passed nor	Yes	Yes	Yes	Yes
6	P value sun	ns	ns	ns	ns
7					
8	**Number of va**	3	3	3	3

		A	B	C	D
		DMSO	5uM UC2288	10uM UC2288	15uM UC2288
1	**Test for norm**				
2	**Shapiro-Wil**				
3	W	0.9776	0.7826	0.8410	0.9441
4	P value	0.7132	0.0737	0.2166	0.5441
5	Passed nor	Yes	Yes	Yes	Yes
6	P value sun	ns	ns	ns	ns
7					
8	**Number of va**	3	3	3	3

		A	B	C	D
		DMSO	5uM UC2288	10uM UC2288	15uM UC2288
1	**Test for norm**				
2	**Shapiro-Wil**				
3	W	0.7785	0.7709	0.8380	0.9653
4	P value	0.0642	0.0467	0.2090	0.6423
5	Passed nor	Yes	No	Yes	Yes
6	P value sun	ns	*	ns	ns
7					
8	**Number of va**	3	3	3	3

	A	B
	DMSO+	0uM UC2288+
1 Test for norm		
2 Shapiro-Will		
3 W	0.7776	0.9182
4 P value	0.0678	0.5268
5 Passed nor	Yes	Yes
6 P value sun	ns	ns
7		
8 Number of va	4	4

		A	B
		DMSO+	0uM UC2288+
1	**Test for norm**		
2	**Shapiro-Wil**		
3	W	0.8440	0.8363
4	P value	0.2074	0.1848
5	Passed nor	Yes	Yes
6	P value sun	ns	ns
7			
8	**Number of va**	4	4

		A	B
		DMSO+	0uM UC2288+
1	**Test for norm**		
2	**Shapiro-Wil**		
3	W	0.6829	0.9959
4	P value	0.0072	0.9854
5	Passed nor	No	Yes
6	P value sun	**	ns
7			
8	**Number of va**	4	4

		A	B
		DMSO+	0uM UC2288
1	Test for norm		
2	Shapiro-Wil		
3	W	0.8772	0.8137
4	P value	0.3267	0.1292
5	Passed nor	Yes	Yes
6	P value sun	ns	ns
7			
8	Number of va	4	4

		A	B
		t siRNA (pooled	p21 (pooled)
1	**Test for norm**		
2	**Shapiro-Will**		
3	W	0.7890	0.8907
4	P value	0.0885	0.3564
5	Passed nor	Yes	Yes
6	P value sun	ns	ns
7			
8	**Number of va**	3	3

		A	B
		t siRNA (pooled	p21 (pooled)
1	**Test for norm**		
2	**Shapiro-Wil**		
3	W	0.9699	0.9969
4	P value	0.6671	0.8940
5	Passed nor	Yes	Yes
6	P value sun	ns	ns
7			
8	**Number of va**	3	3

		A	B	C
		nt gRNA	p21 gRNA 1	p21 gRNA 2
1	**Test for norm**			
2	**Shapiro-Wil**			
3	W	0.7720	0.8573	0.8495
4	P value	0.0493	0.2601	0.2392
5	Passed nor	No	Yes	Yes
6	P value sun	*	ns	ns
7				
8	**Number of va**	3	3	3

		A	B	C
		nt gRNA	p21 gRNA 1	p21 gRNA 2
1	**Test for norm**			
2	**Shapiro-Wil**			
3	W	0.9835	0.8726	0.9999
4	P value	0.7539	0.3028	0.9858
5	Passed nor	Yes	Yes	Yes
6	P value sun	ns	ns	ns
7				
8	**Number of va**	3	3	3

		A	B
		DMSO	10uM SFN
1	**Test for norm**		
2	**Shapiro-Wil**		
3	W	0.9953	0.9178
4	P value	0.8688	0.4446
5	Passed nor	Yes	Yes
6	P value sun	ns	ns
7			
8	**Number of va**	3	3

		A	B
		DMSO	10uM SFN
1	**Test for norm**		
2	**Shapiro-Wil**		
3	W	0.9562	0.8813
4	P value	0.5974	0.3282
5	Passed nor	Yes	Yes
6	P value sun	ns	ns
7			
8	**Number of va**	3	3

		A	B	C	D
		DMSO	5 uM SFN	10 uM SFN	15 uM SFN
1	**Test for norm**				
2	**Shapiro-Wil**				
3	W	0.8986	0.8850	0.8362	0.9977
4	P value	0.3812	0.3392	0.2041	0.9090
5	Passed nor	Yes	Yes	Yes	Yes
6	P value sun	ns	ns	ns	ns
7					
8	**Number of va**	3	3	3	3

	A	B	C	D
	DMSO	5 uM SFN	10 uM SFN	15 uM SFN
1 Test for norm				
2 Shapiro-Wil				
3 W	0.8016	0.8219	0.9932	0.9326
4 P value	0.1183	0.1679	0.8421	0.4986
5 Passed nor	Yes	Yes	Yes	Yes
6 P value sun	ns	ns	ns	ns
7				
8 Number of va	3	3	3	3

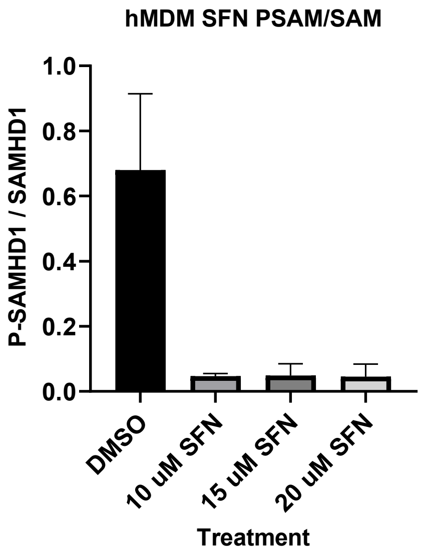
hMDM SFN PSAM/SAM
P-SAMHD1 / SAMHD1
1.0
0.8
0.6
0.4
0.2
0.0
DMSO
10 uM SFN
15 uM SFN
20 uM SFN
Treatment

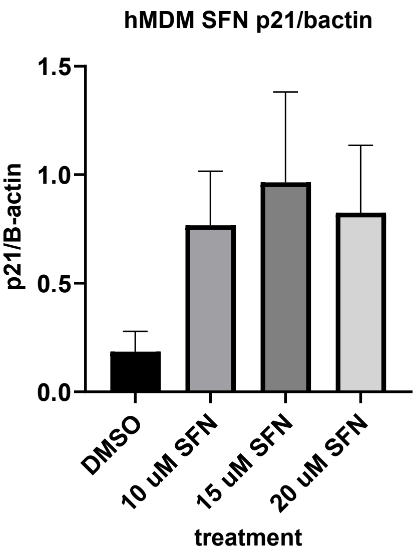
hMDM SFN p21/bactin
1.5
1.0
0.5
0.0
p21/B-actin
DMSO
10 uM SFN
15 uM SFN
20 uM SFN
treatment

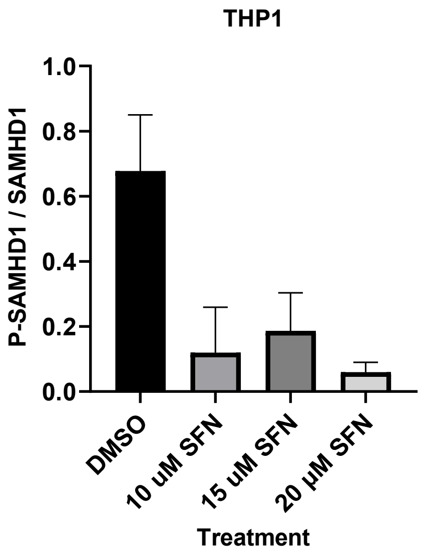
THP1
P-SAMHD1 / SAMHD1
1.0
0.8
0.6
0.4
0.2
0.0
DMSO
10 uM SFN
15 uM SFN
20 μM SFN
Treatment

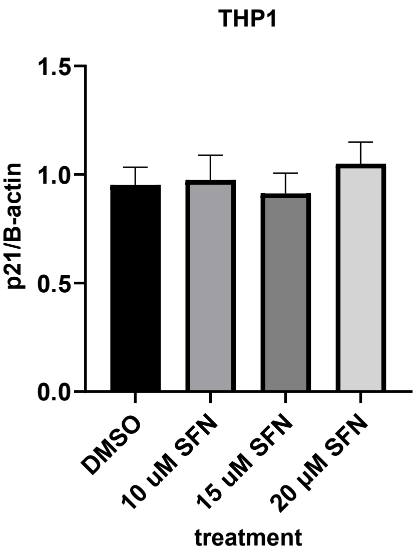
THP1
p21/B-actin
1.5
1.0
0.5
0.0
DMSO
10 uM SFN
15 uM SFN
20 µM SFN
treatment

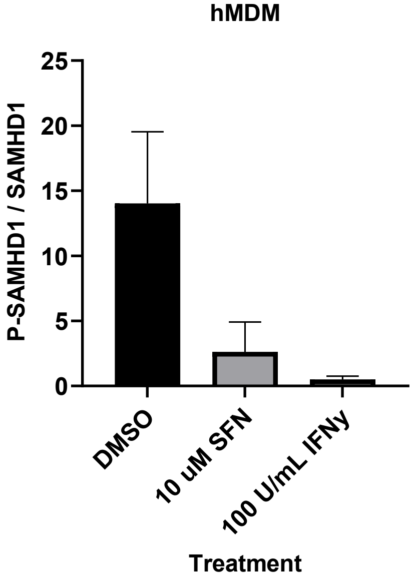
hMDM
P-SAMHD1 / SAMHD1
25
20
15
10
5
0
DMSO
10 uM SFN
100 U/mL IFNy
Treatment

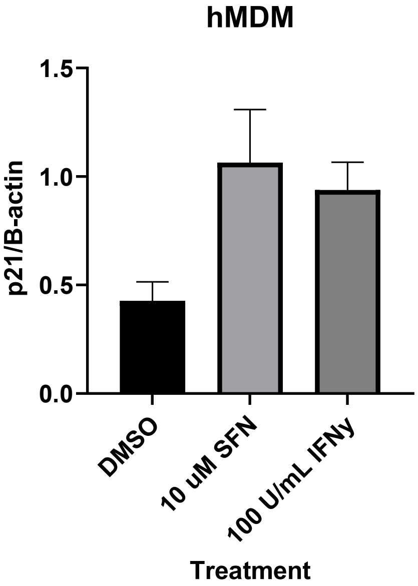
hMDM
p21/B-actin
1.5
1.0
0.5
0.0
DMSO
10 uM SFN
100 U/mL IFNy
Treatment

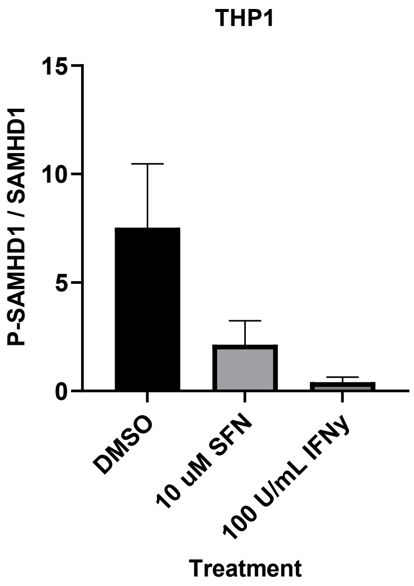
THP1
P-SAMHD1 / SAMHD1
15
10
5
0
DMSO
10 uM SFN
100 U/mL IFNy
Treatment

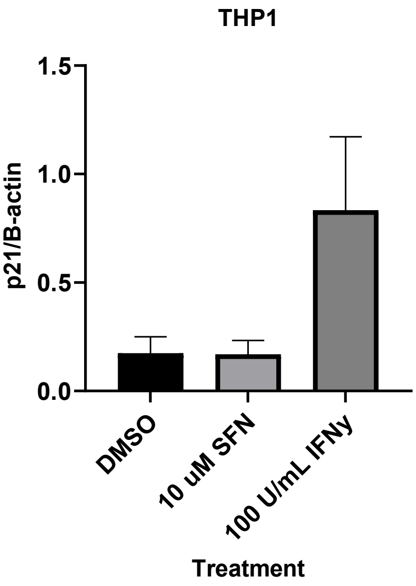
THP1
1.5
1.0
0.5
0.0
p21/B-actin
DMSO
10 uM SFN
100 U/mL IFNy
Treatment

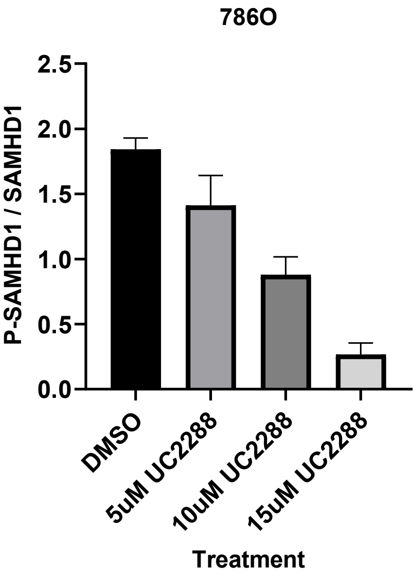

786O
P-SAMHD1 / SAMHD1
2.5
2.0
1.5
1.0
0.5
0.0
DMSO
5uM UC2288
10uM UC2288
15uM UC2288
Treatment

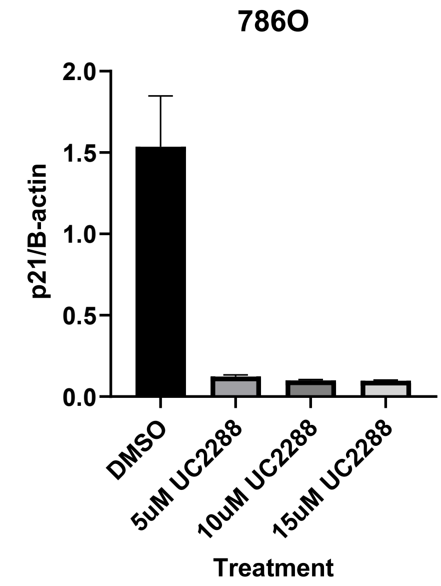

786O
2.0
1.5
1.0
0.5
0.0
p21/B-actin
DMSO
5uM UC2288
10uM UC2288
15uM UC2288
Treatment

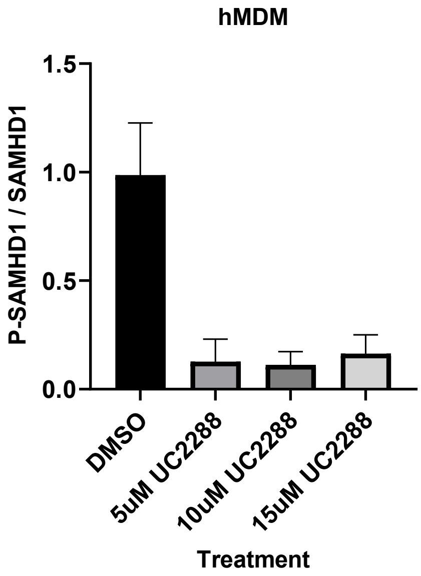
hMDM
P-SAMHD1 / SAMHD1
1.5
1.0
0.5
0.0
DMSO
5uM UC2288
10uM UC2288
15uM UC2288
Treatment

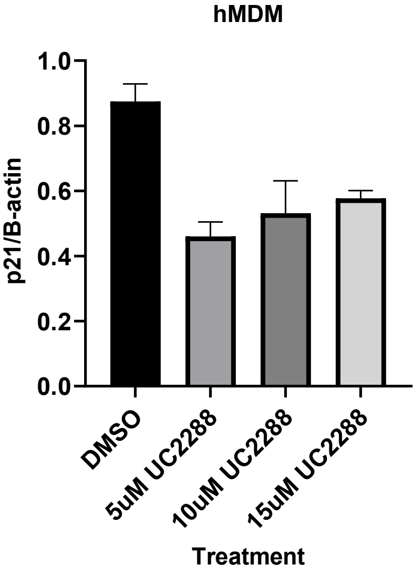
hMDM
1.0
0.8
0.6
0.4
0.2
0.0
p21/B-actin
DMSO
5uM UC2288
10uM UC2288
15uM UC2288
Treatment

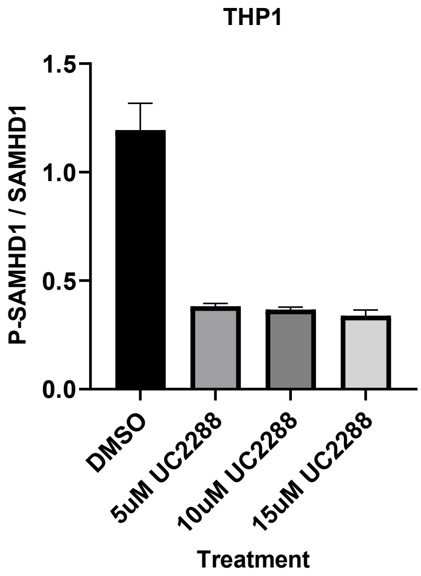
THP1
P-SAMHD1 / SAMHD1
1.5
1.0
0.5
0.0
DMSO
5uM UC2288
10uM UC2288
15uM UC2288
Treatment

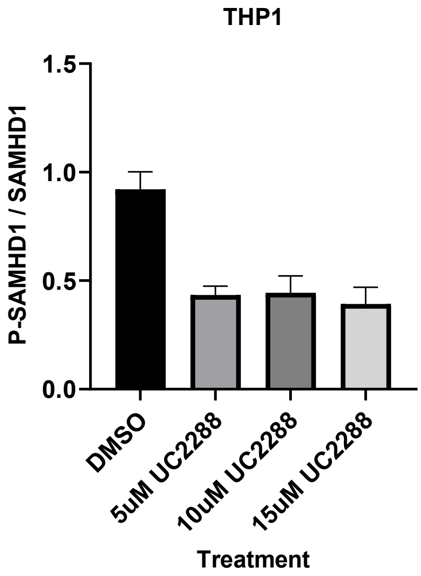
THP1
P-SAMHD1 / SAMHD1
1.5
1.0
0.5
0.0
DMSO
5uM UC2288
10uM UC2288
15uM UC2288
Treatment

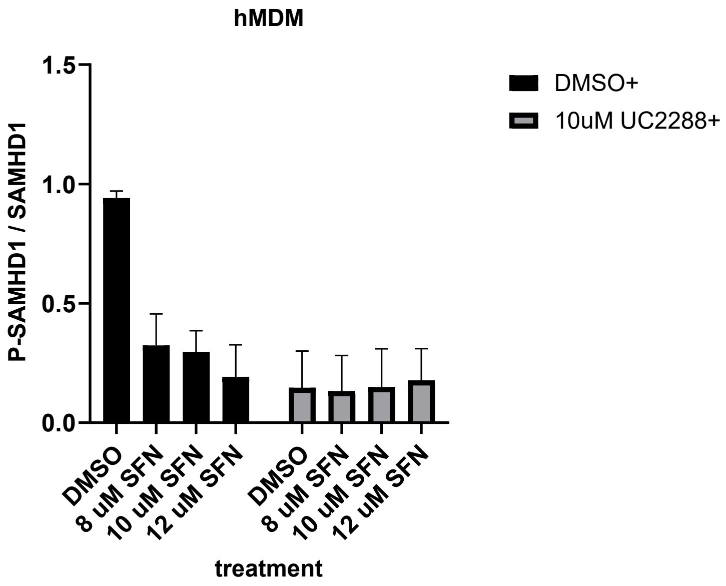

hMDM
1.5
1.0
0.5
0.0
P-SAMHD1 / SAMHD1
DMSO
8 uM SFN
10 uM SFN
12 uM SFN
DMSO
8 uM SFN
10 uM SFN
12 uM SFN
treatment
DMSO+
10uM UC2288+

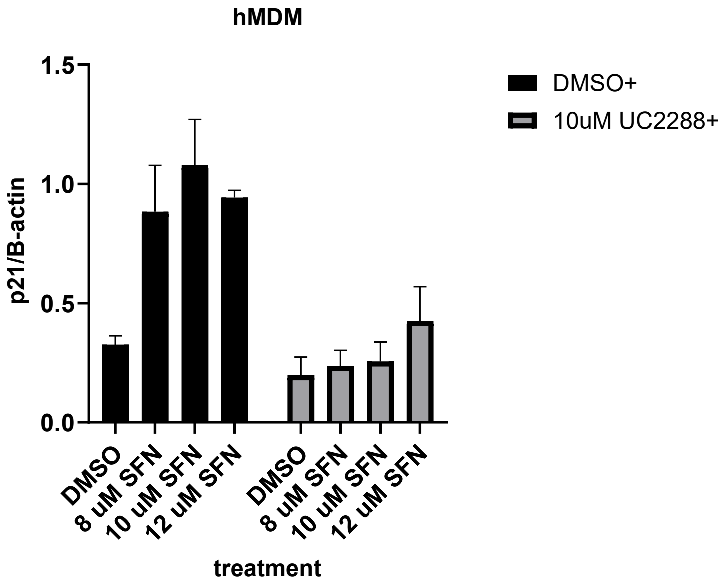
hMDM
1.5
1.0
0.5
0.0
p21/B-actin
DMSO
8 uM SFN
10 uM SFN
12 uM SFN
DMSO
8 uM SFN
10 uM SFN
12 uM SFN
treatment
DMSO+
10uM UC2288+

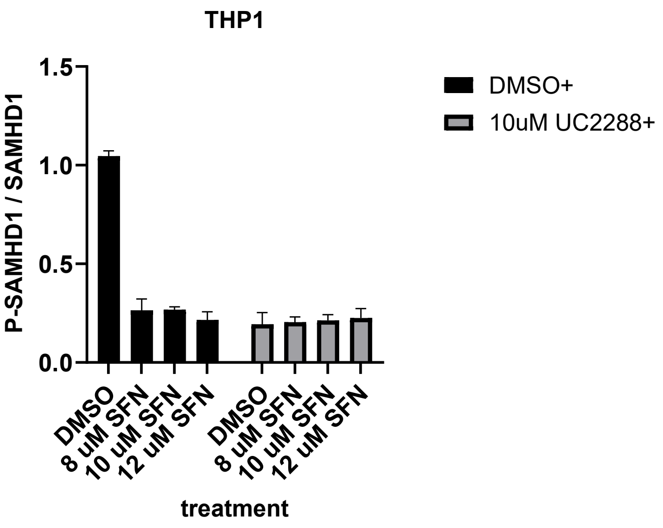
THP1
P-SAMHD1 / SAMHD1
1.5
1.0
0.5
0.0
DMSO
8 uM SFN
10 uM SFN
12 uM SFN
DMSO
8 uM SFN
10 uM SFN
12 uM SFN
treatment
DMSO+
10uM UC2288+

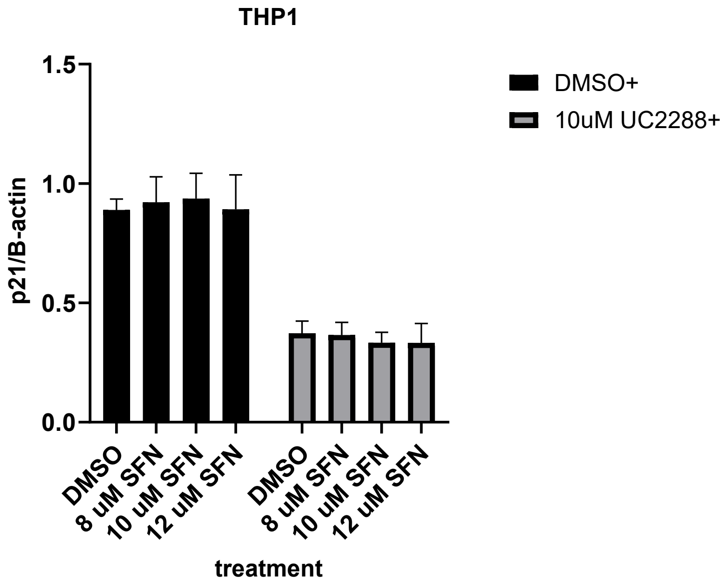
THP1
1.5
1.0
0.5
0.0
p21/B-actin
DMSO+
10uM UC2288+
DMSO
8 uM SFN
10 uM SFN
12 uM SFN
DMSO
8 uM SFN
10 uM SFN
12 uM SFN
treatment

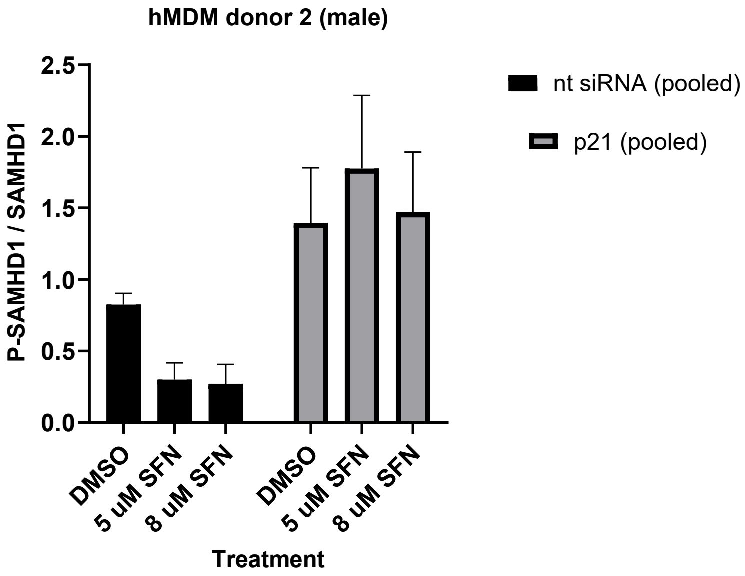

hMDM donor 2 (male)
P-SAMHD1 / SAMHD1
2.5
2.0
1.5
1.0
0.5
0.0
DMSO
5 uM SFN
8 uM SFN
DMSO
5 uM SFN
8 uM SFN
Treatment
nt siRNA (pooled)
p21 (pooled)

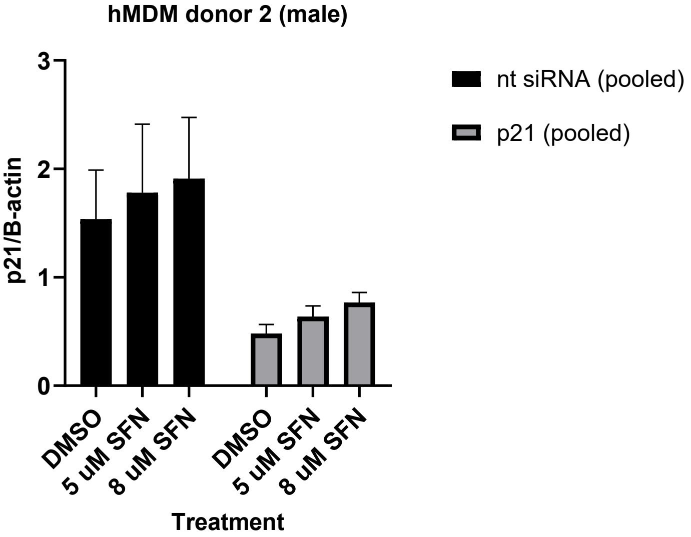
hMDM donor 2 (male)
p21/B-actin
3
2
1
0
DMSO
5 uM SFN
8 uM SFN
DMSO
5 uM SFN
8 uM SFN
Treatment
nt siRNA (pooled)
p21 (pooled)

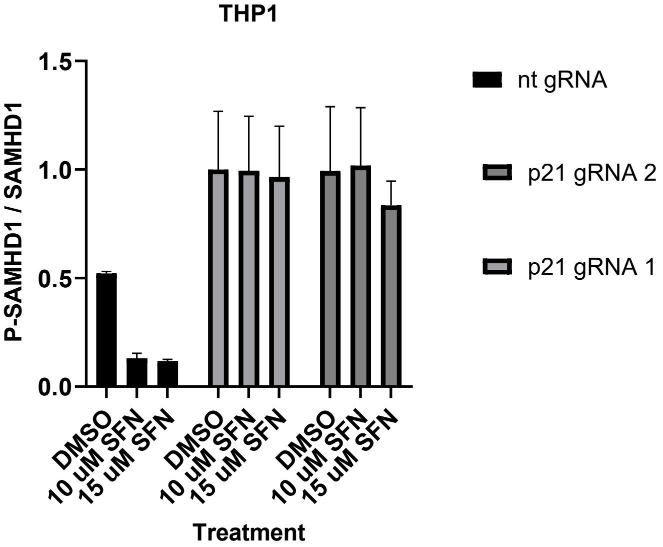
THP1
P-SAMHD1 / SAMHD1
1.5
1.0
0.5
0.0
DMSO
10 uM SFN
15 uM SFN
DMSO
10 uM SFN
15 uM SFN
DMSO
10 uM SFN
15 uM SFN
Treatment
nt gRNA
p21 gRNA 2
p21 gRNA 1

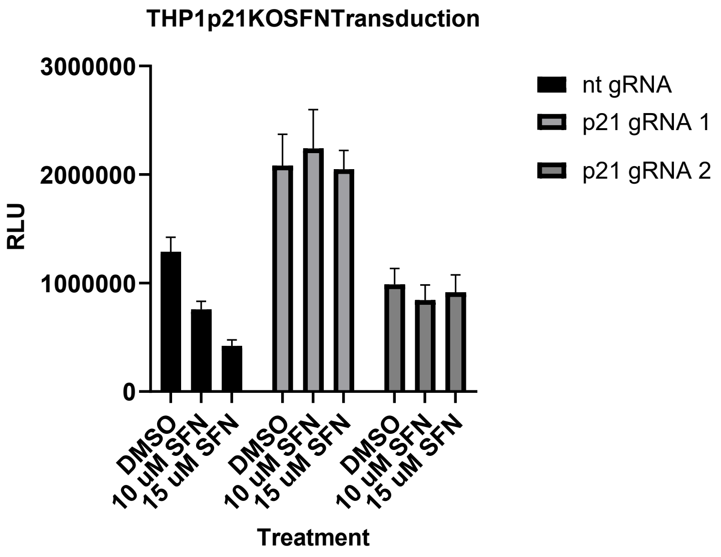
THP1p21KOSFNTransduction
3000000
2000000
1000000
0
RLU
nt gRNA
p21 gRNA 1
p21 gRNA 2
DMSO
10 uM SFN
15 uM SFN
DMSO
10 uM SFN
15 uM SFN
DMSO
10 uM SFN
15 uM SFN
Treatment

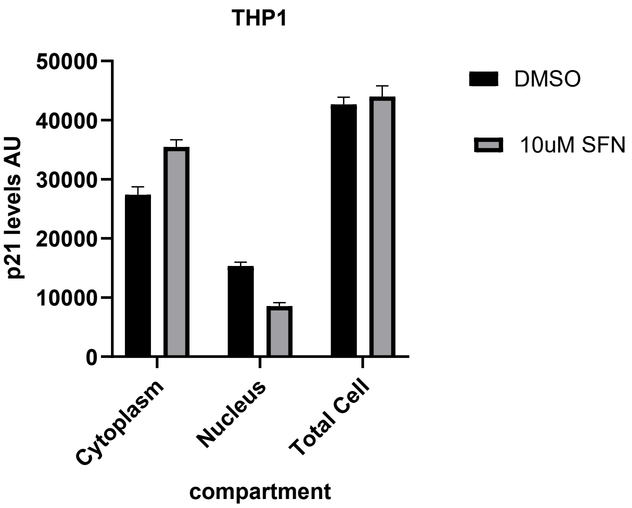
THP1
50000
40000
30000
20000
10000
0
p21 levels AU
Cytoplasm
Nucleus
Total Cell
compartment
DMSO
10uM SFN

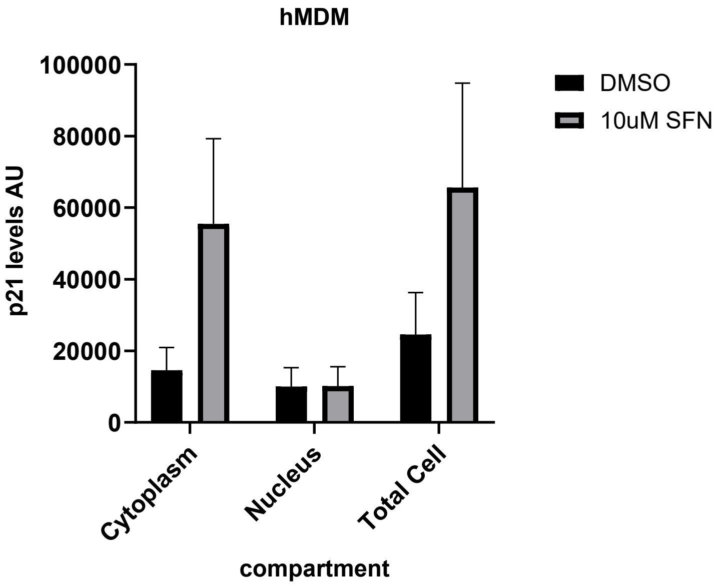
hMDM
p21 levels AU
100000
80000
60000
40000
20000
0
Cytoplasm
Nucleus
Total Cell
compartment
DMSO
10uM SFN

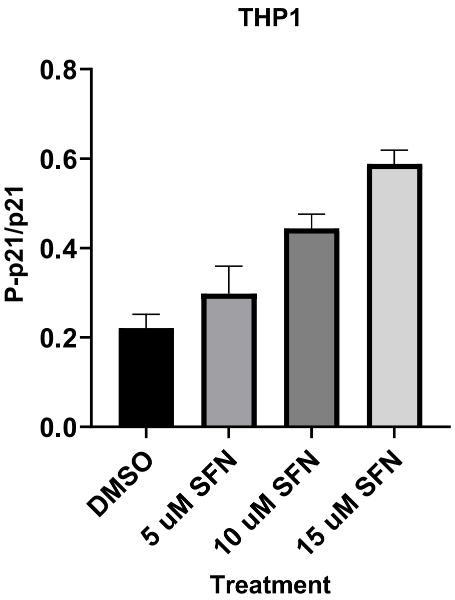
THP1
0.8
0.6
0.4
0.2
0.0
P-p21/p21
DMSO
5 uM SFN
10 uM SFN
15 uM SFN
Treatment

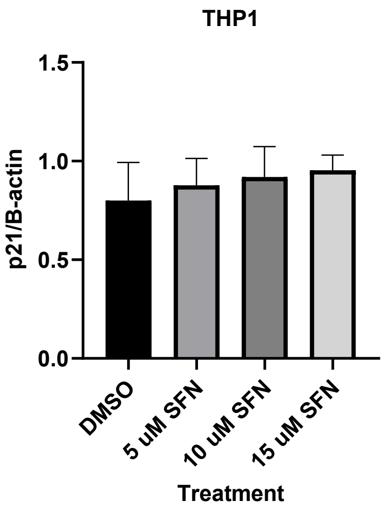
THP1
1.5
1.0
0.5
0.0
p21/B-actin
DMSO
5 uM SFN
10 uM SFN
15 uM SFN
Treatment

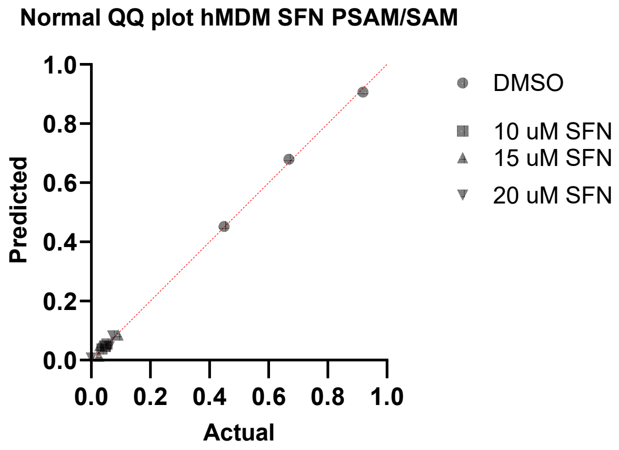

Normal QQ plot hMDM SFN PSAM/SAM
Predicted
Actual
0.0
0.2
0.4
0.6
0.8
1.0
DMSO
10 uM SFN
15 uM SFN
20 uM SFN

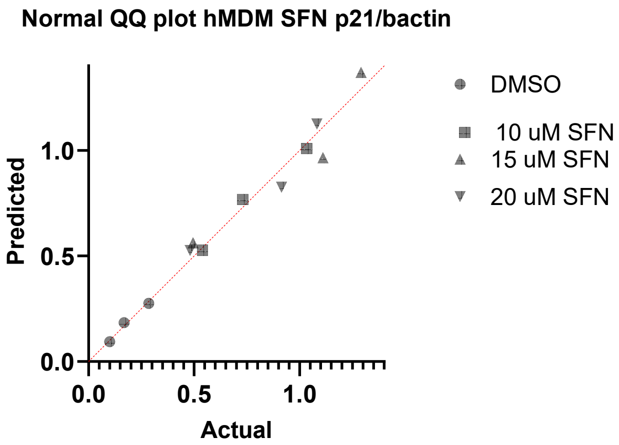
Normal QQ plot hMDM SFN p21/bactin
Predicted
Actual
0.0
0.5
1.0
DMSO
10 uM SFN
15 uM SFN
20 uM SFN

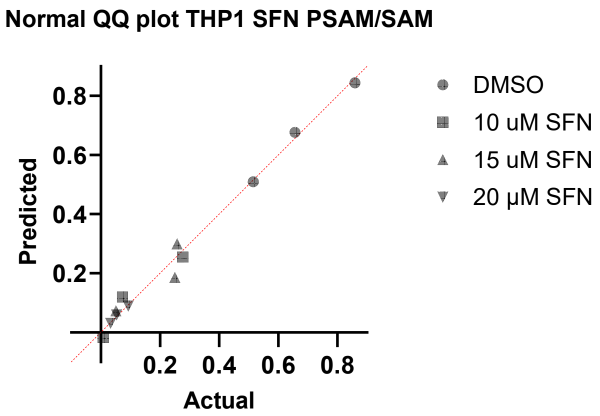

Normal QQ plot THP1 SFN PSAM/SAM
0.8
0.6
0.4
0.2
Predicted
0.2
0.4
0.6
0.8
Actual
DMSO
10 uM SFN
15 uM SFN
20 μM SFN

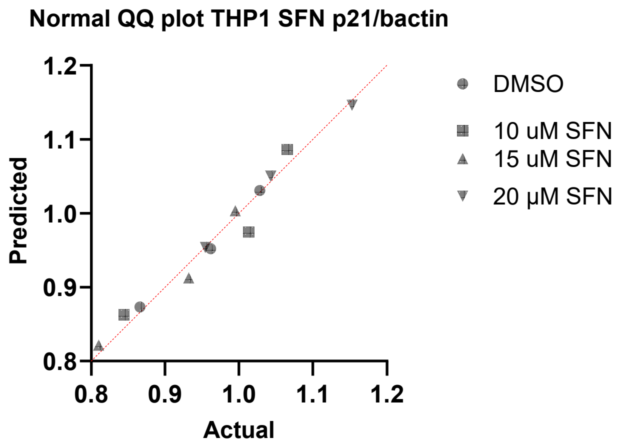

Normal QQ plot THP1 SFN p21/bactin
Predicted
1.2
1.1
1.0
0.9
0.8
0.8
0.9
1.0
1.1
1.2
Actual
DMSO
10 uM SFN
15 uM SFN
20 µM SFN

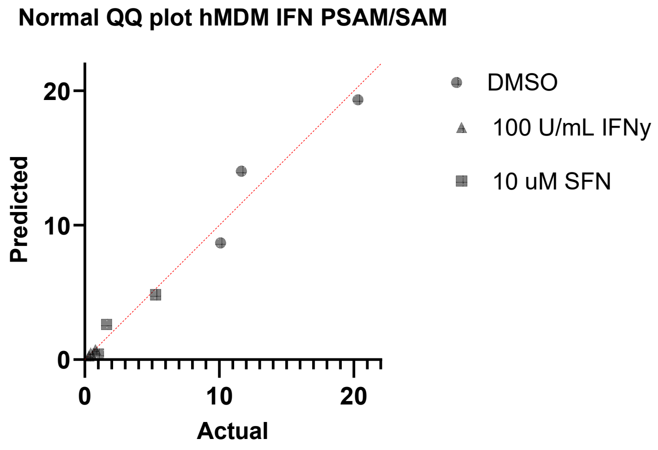
Normal QQ plot hMDM IFN PSAM/SAM
Predicted
Actual
0
10
20
DMSO
100 U/mL IFNy
10 uM SFN

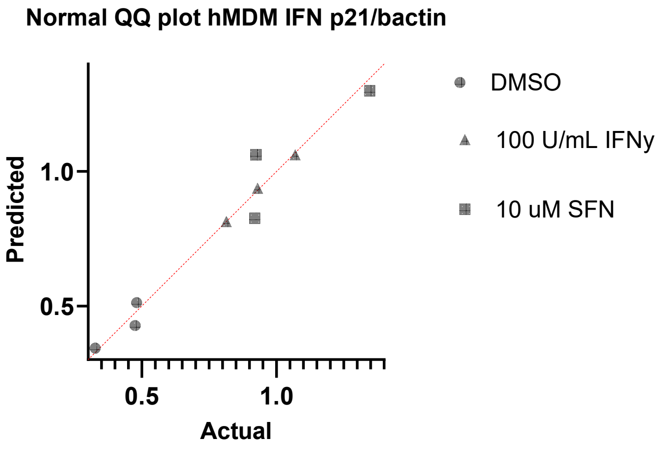
Normal QQ plot hMDM IFN p21/bactin
Predicted
1.0
0.5
0.5
1.0
Actual
DMSO
100 U/mL IFNy
10 uM SFN

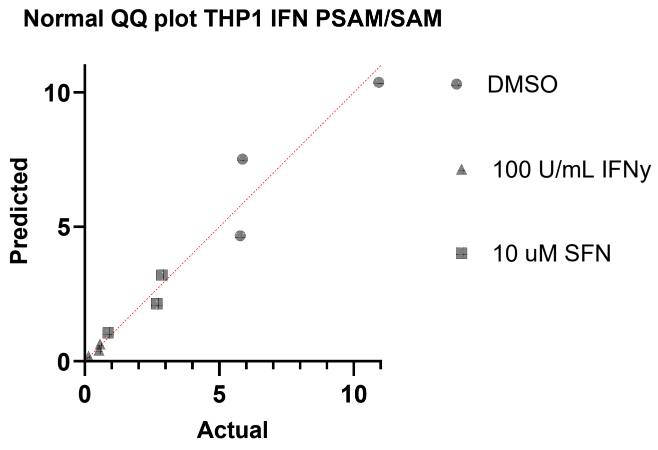
Normal QQ plot THP1 IFN PSAM/SAM
Predicted
Actual
0
5
10
0
5
10
DMSO
100 U/mL IFNy
10 uM SFN

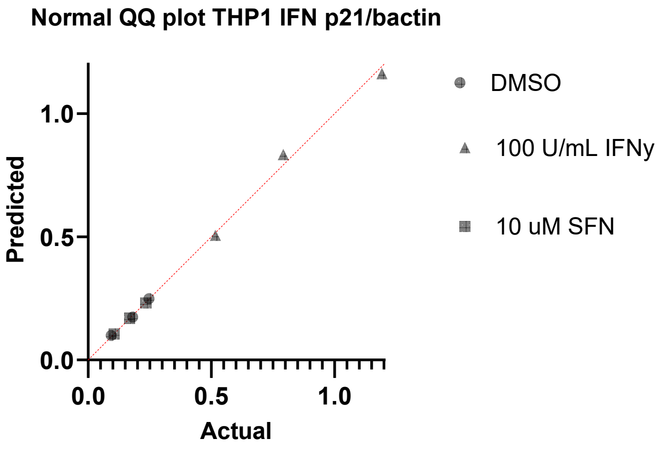

Normal QQ plot THP1 IFN p21/bactin
Predicted
1.0
0.5
0.0
0.0
0.5
1.0
Actual
DMSO
100 U/mL IFNy
10 uM SFN

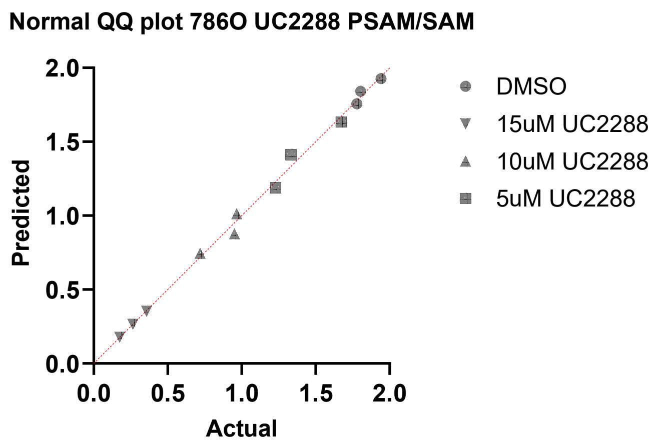
Normal QQ plot 786O UC2288 PSAM/SAM
Predicted
Actual
0.0
0.5
1.0
1.5
2.0
DMSO
15uM UC2288
10uM UC2288
5uM UC2288

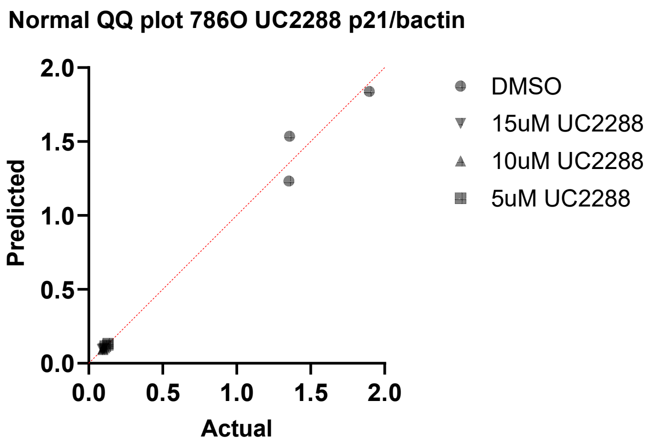
Normal QQ plot 786O UC2288 p21/bactin
2.0
1.5
1.0
0.5
0.0
Predicted
0.0
0.5
1.0
1.5
2.0
Actual
DMSO
15uM UC2288
10uM UC2288
5uM UC2288

Normal QQ plot hMDM UC2288 PSAM/SAM

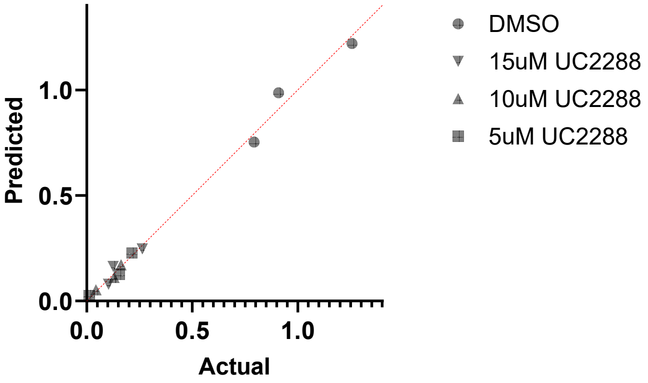

Normal QQ plot hMDM UC2288 p21/bactin

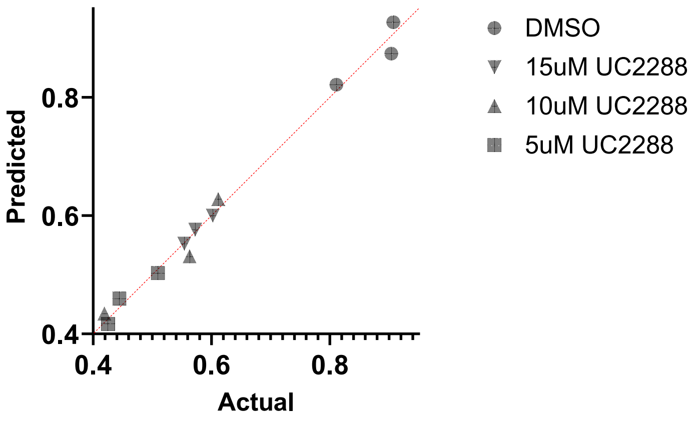

Normal QQ plot THP1 UC2288 PSAM/SAM

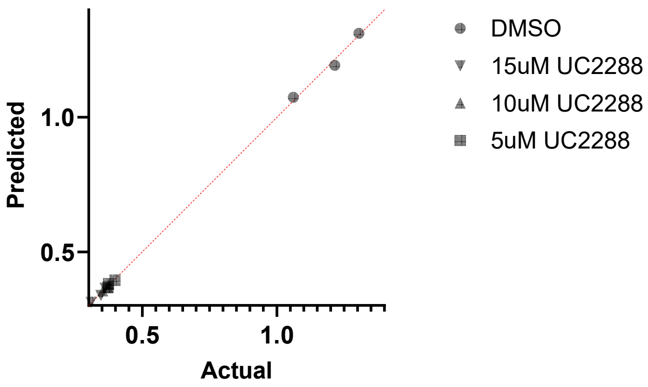

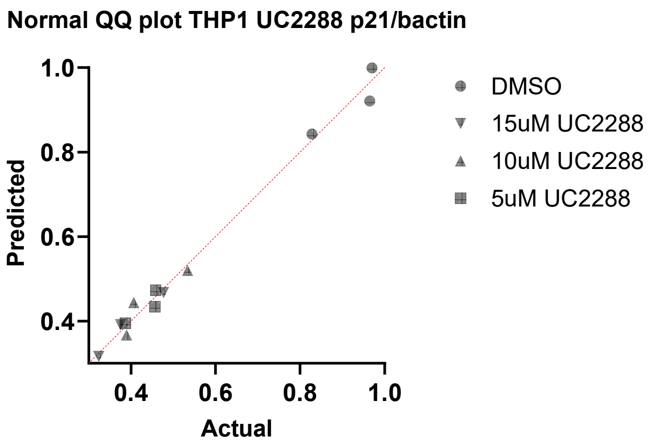
Normal QQ plot THP1 UC2288 p21/bactin
Predicted
Actual
1.0
0.8
0.6
0.4
0.4
0.6
0.8
1.0
DMSO
15uM UC2288
10uM UC2288
5uM UC2288

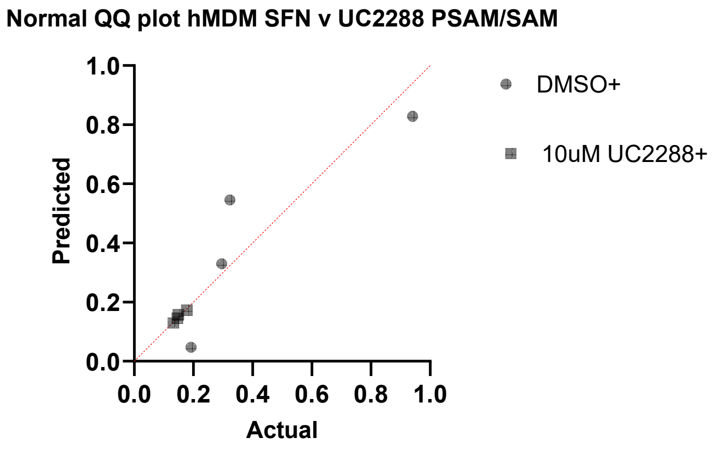
Normal QQ plot hMDM SFN v UC2288 PSAM/SAM
Predicted
Actual
0.0
0.2
0.4
0.6
0.8
1.0
DMSO+
10uM UC2288+

Normal QQ plot hMDM SFN v UC2288 p21/bactin

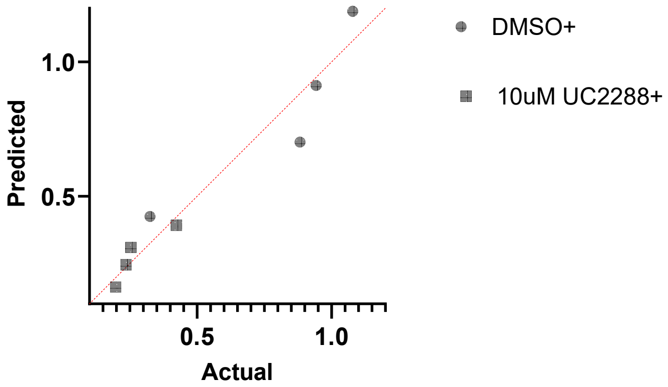

Normal QQ plot THP1 SFN v UC2288 PSAM/SAM

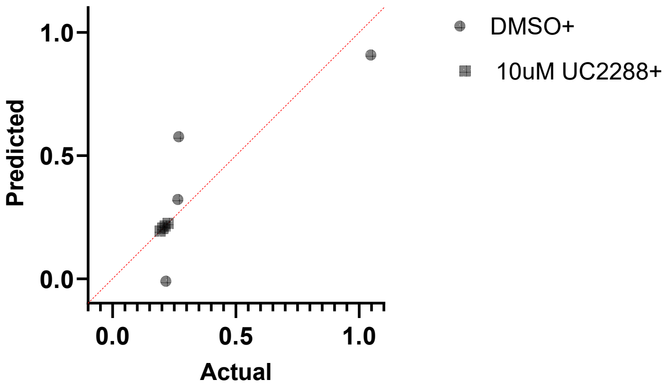

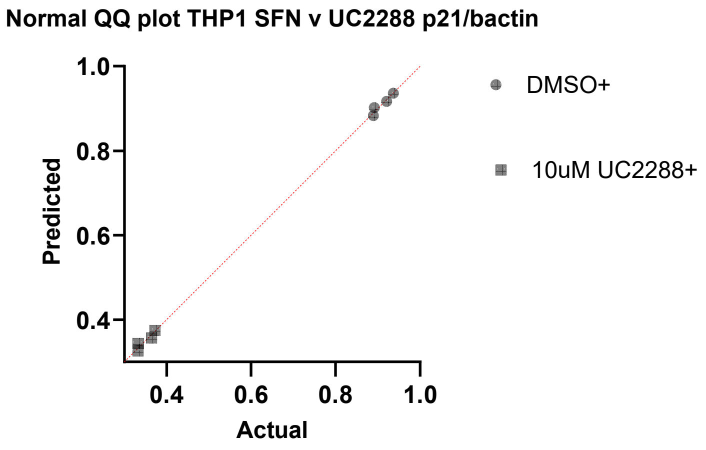
Normal QQ plot THP1 SFN v UC2288 p21/bactin
1.0
0.8
0.6
0.4
Predicted
0.4
0.6
0.8
1.0
Actual
DMSO+
10uM UC2288+

Normal QQ plot hMDM p21 siRNA SFN Donor2 male PSAM/SAM

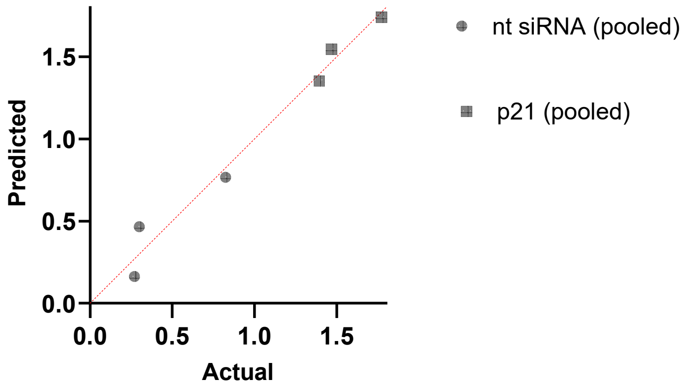

Normal QQ plot hMDM p21 siRNA SFN Donor2 male p21/bactin

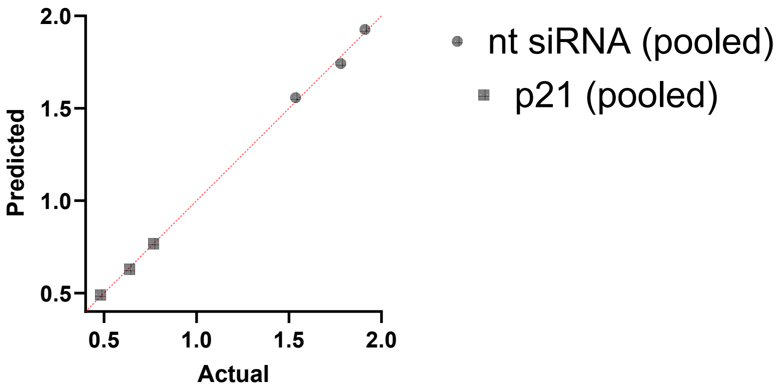

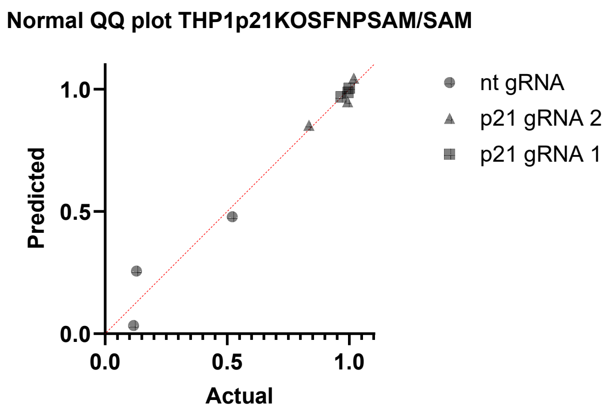
Normal QQ plot THP1p21KOSFNPSAM/SAM
Predicted
Actual
1.0
0.5
0.0
0.0
0.5
1.0
nt gRNA
p21 gRNA 2
p21 gRNA 1

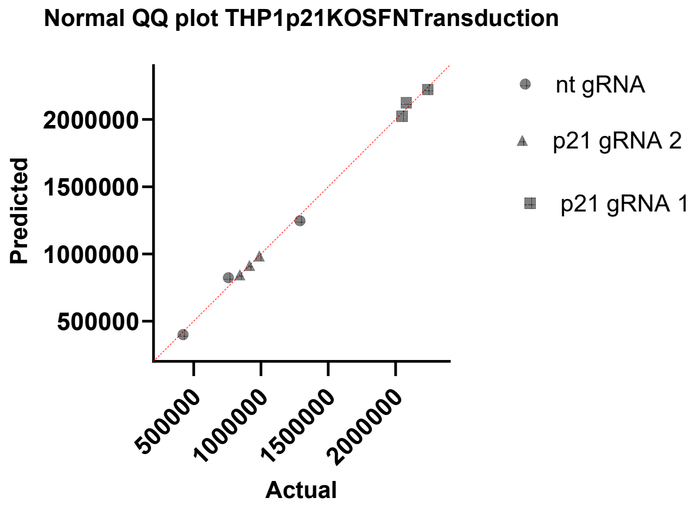
Normal QQ plot THP1p21KOSFNTransduction
Predicted
2000000
1500000
1000000
500000
500000
1000000
1500000
2000000
Actual
nt gRNA
p21 gRNA 2
p21 gRNA 1

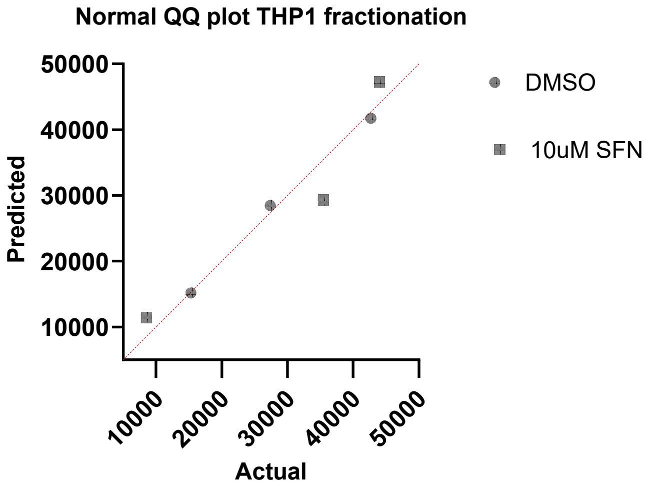

Normal QQ plot THP1 fractionation
50000
40000
30000
20000
10000
Predicted
10000
20000
30000
40000
50000
Actual
DMSO
10uM SFN

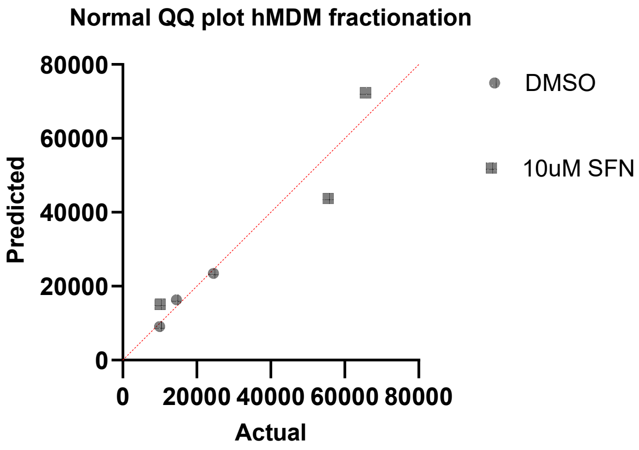
Normal QQ plot hMDM fractionation
80000
60000
40000
20000
0
Predicted
0
20000
40000
60000
80000
Actual
DMSO
10uM SFN

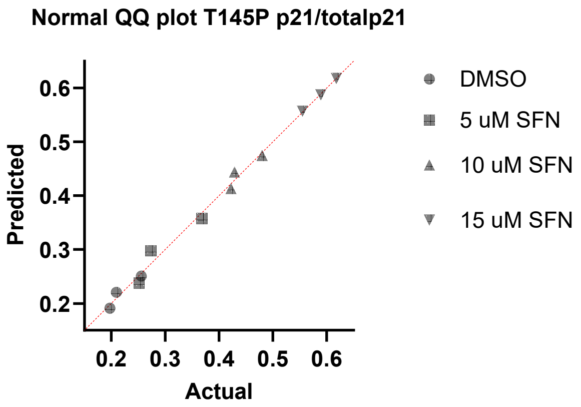

Normal QQ plot T145P p21/totalp21
Predicted
0.6
0.5
0.4
0.3
0.2
0.2
0.3
0.4
0.5
0.6
Actual
DMSO
5 uM SFN
10 uM SFN
15 uM SFN

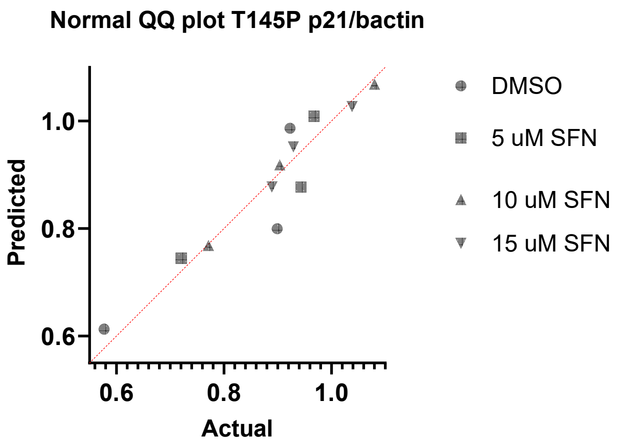
Normal QQ plot T145P p21/bactin
Predicted
Actual
1.0
0.8
0.6
0.6
0.8
1.0
DMSO
5 uM SFN
10 uM SFN
15 uM SFN

Constant	Value
Experiment Date	7/14/2026
Experiment ID	
Notebook ID	
Project	
Experimenter	
Protocol	
