# Supplementary material for: p21 (CDKN1A) Is the Major Driver of Sulforaphane-Mediated Reduction in SAMHD1 T592 Phosphorylation in Macrophages
Source: Biomolecules. 2026 Aug 20;16(8):1213. doi: 10.3390/biom16081213 (PMC13511314; doi:10.3390/biom16081213)
Supplement: Supplementary file 1 [file biomolecules-16-01213-s001.zip › Supplementary File S4 Addiitional Figures and Tables/trypan blue exclusion.pdf]

THP-1 Trypan Blue Exclusion Images

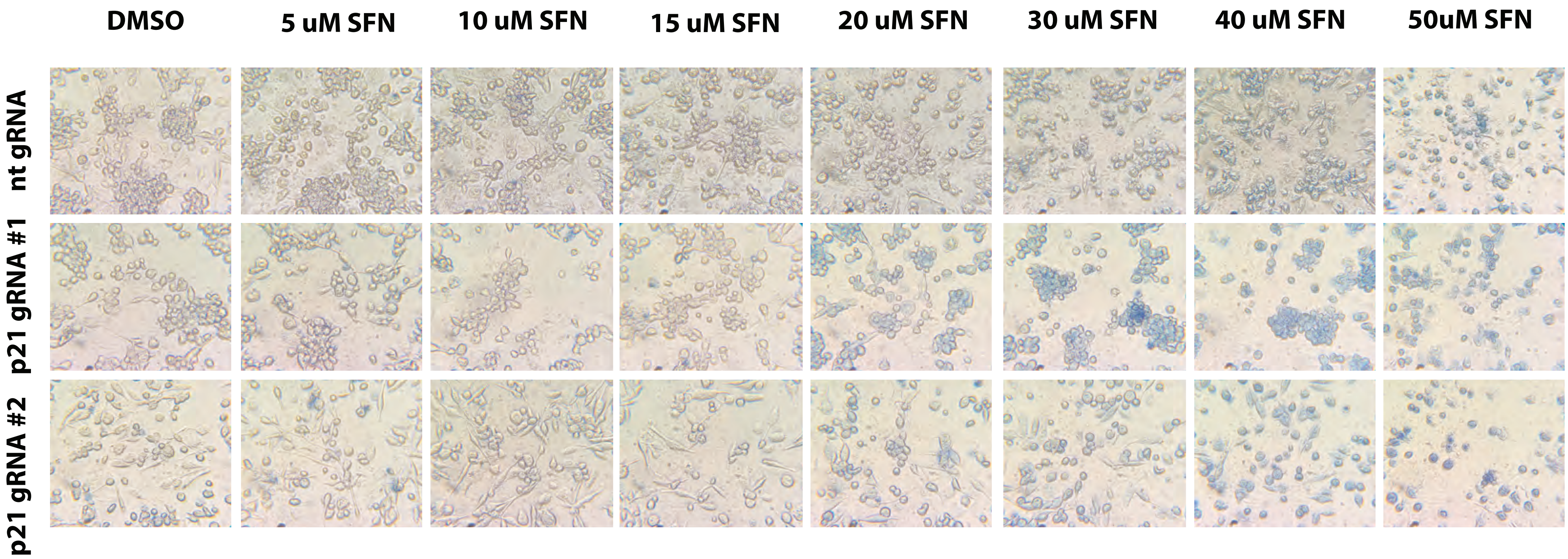

Panel #	Position	Visual Phenotype Observations	Estimated Viability Range	
1	Row 1, Col 1	High density, bright, healthy spherical clusters; virtually no blue dye.	95% – 98%	
2	Row 1, Col 2	Highly dense, clear bright cells; minimal to no staining.	95% – 98%	
3	Row 1, Col 3	High density, bright cell clusters.	93% – 97%	
4	Row 1, Col 4	High density, bright cell clusters.	93% – 97%	
5	Row 1, Col 5	High density, bright cells; minor dark debris starting to appear.	90% – 95%	
6	Row 1, Col 6	High density, mostly viable with rare dark/blue points.	90% – 95%	
7	Row 1, Col 7	Dense cells; subtle increase in dark/blue staining in clusters.	85% – 92%	
8	Row 1, Col 8	Dense cells; scattered small blue/dark non-viable cells.	80% – 88%	
9	Row 2, Col 1	Moderate-high density, bright round cells; healthy.	92% – 96%	
10	Row 2, Col 2	Moderate-high density, bright round cells.	90% – 95%	
11	Row 2, Col 3	Bright cells, clean background.	90% – 95%	
12	Row 2, Col 4	Bright cells; slight onset of small cellular aggregates.	85% – 92%	
13	Row 2, Col 5	Clear appearance of dark blue-stained cell aggregates.	70% – 80%	
14	Row 2, Col 6	Distinct, large dark blue cell clumps; clear cells are heavily reduced.	50% – 65%	
15	Row 2, Col 7	Massive blue-stained non-viable aggregates dominating the field.	30% – 45%	
16	Row 2, Col 8	Severe cell death; dense blue-stained aggregates with few viable cells.	25% – 40%	
17	Row 3, Col 1	Healthy, elongated/spindly adherent cells; completely clear.	95% – 98%	
18	Row 3, Col 2	Elongated, clear adherent phenotype; healthy.	95% – 98%	
19	Row 3, Col 3	Elongated, clear adherent cells.	95% – 98%	
20	Row 3, Col 4	Mostly elongated cells; minor initial cell rounding.	90% – 95%	
21	Row 3, Col 5	Shift toward rounded cell morphology; some dark blue uptake.	80% – 88%	
22	Row 3, Col 6	Marked increase in rounded, distinct dark blue non-viable cells.	65% – 75%	
23	Row 3, Col 7	Widespread scattered blue-stained round dead cells and debris.	50% – 65%	
24	Row 3, Col 8	Predominantly rounded, dark blue-stained non-viable cells.	35% – 50%	
